# Structurally diverse triterpenoid alkaloids from *Buxus myrica* with cytotoxic and apoptosis-inducing activities

**DOI:** 10.1007/s13659-026-00656-6

**Published:** 2026-11-03

**Authors:** Yuwei Wang, Miao Wang, Yang Zhang, Huiyuan Gao

**Affiliations:** School of Traditional Chinese Materia Medica, Key Laboratory of Structure-Based Drug Design & Discovery of Ministry of Education, Shenyang Pharmaceutical University, Shenyang, 110016 China

**Keywords:** *Buxus myrica*, Triterpenoid alkaloids, 9,10-secocycloartane, Conformational analysis, Cytotoxicity, Molecular docking

## Abstract

Sixteen triterpenoid alkaloids, including twelve previously undescribed ones named myribuxines A–L (**1**–**12**), were isolated from the twigs and leaves of *Buxus myrica* H. Lév. To our knowledge, this species has not yet undergone any phytochemical or pharmacological investigation. Their structures and relative configurations were elucidated by comprehensive spectroscopic analysis and DP4^+^ probability calculations, with their absolute configurations tentatively assigned on the basis of the biosynthetic origin of *Buxus* alkaloids. Structurally, **5**, **6**, **7**, and **12** feature a rare C-16 aromatic ester substituent, while **9**, **10**, and **11** possess a rare tetrahydrofuran ring formed by a C-10–O–C-23 ether linkage. All isolated alkaloids were evaluated for cytotoxicity against HepG2, A549, U251, and HCT-116 cell lines. The active alkaloids showed modest cytotoxicity, with half maximal inhibitory concentration (IC_50_) values ranging from 17.5 to 47.3 μM across the four cell lines. Among them, **2** exhibited modest inhibitory activity toward HepG2 cells. Flow cytometry analysis revealed that **2** promoted apoptosis in a dose- and time-dependent manner. Furthermore, network pharmacology screening predicted HSP90α as its potential molecular target. Follow-up molecular docking further revealed the binding model between compound** 2** and HSP90.

**Graphical abstract:**

**Figure:**
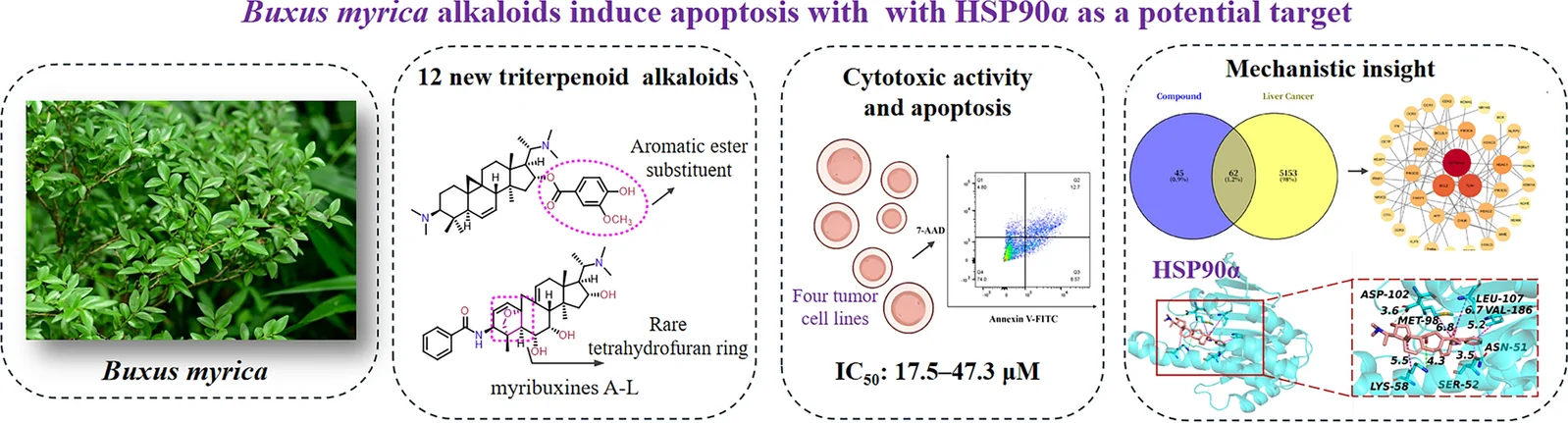

**Supplementary Information:**

The online version contains supplementary material available at https://doi.org/10.1007/s13659-026-00656-6.

## Highlights

- First phytochemical study of Buxus myrica H. Lév.
- Twelve new myribuxines A–L with cycloartane and 9,10-secocycloartane skeletons.
- Cytotoxicity against four human cancer cell lines (IC50: 17.5–47.3 μM).
- Dose- and time-dependent induction of apoptosis by 2 in HepG2 cells.
- HSP90α was computationally predicted as a potential candidate target of 2.

**Supplementary Information:**

The online version contains supplementary material available at https://doi.org/10.1007/s13659-026-00656-6.

## Introduction

The genus *Buxus* (Buxaceae) comprises approximately 100 species globally, which are widely distributed across tropical and subtropical regions [1]. Plants of this genus have long been used in traditional medicine to treat rheumatism, malaria, skin infections, and cardiovascular diseases [2, 3]. Over the past few decades, phytochemical investigations have consistently revealed *Buxus* species to be a rich source of structurally unique triterpenoid alkaloids [4]. These alkaloids are primarily characterized by a degraded cycloartane or pregnane skeleton, typically featuring either an intact 9,19-cyclopropane ring or a 9,10-secocycloartane ring system with varying degrees of unsaturation [2]. Owing to their structurally intricate frameworks, *Buxus* alkaloids exhibit diverse pharmacological potentials, such as acetylcholinesterase inhibitory, cytotoxic, and antibacterial activities, which prompts us to continue seeking more structurally novel *Buxus* alkaloids [5, 6].

**Fig. 1:**
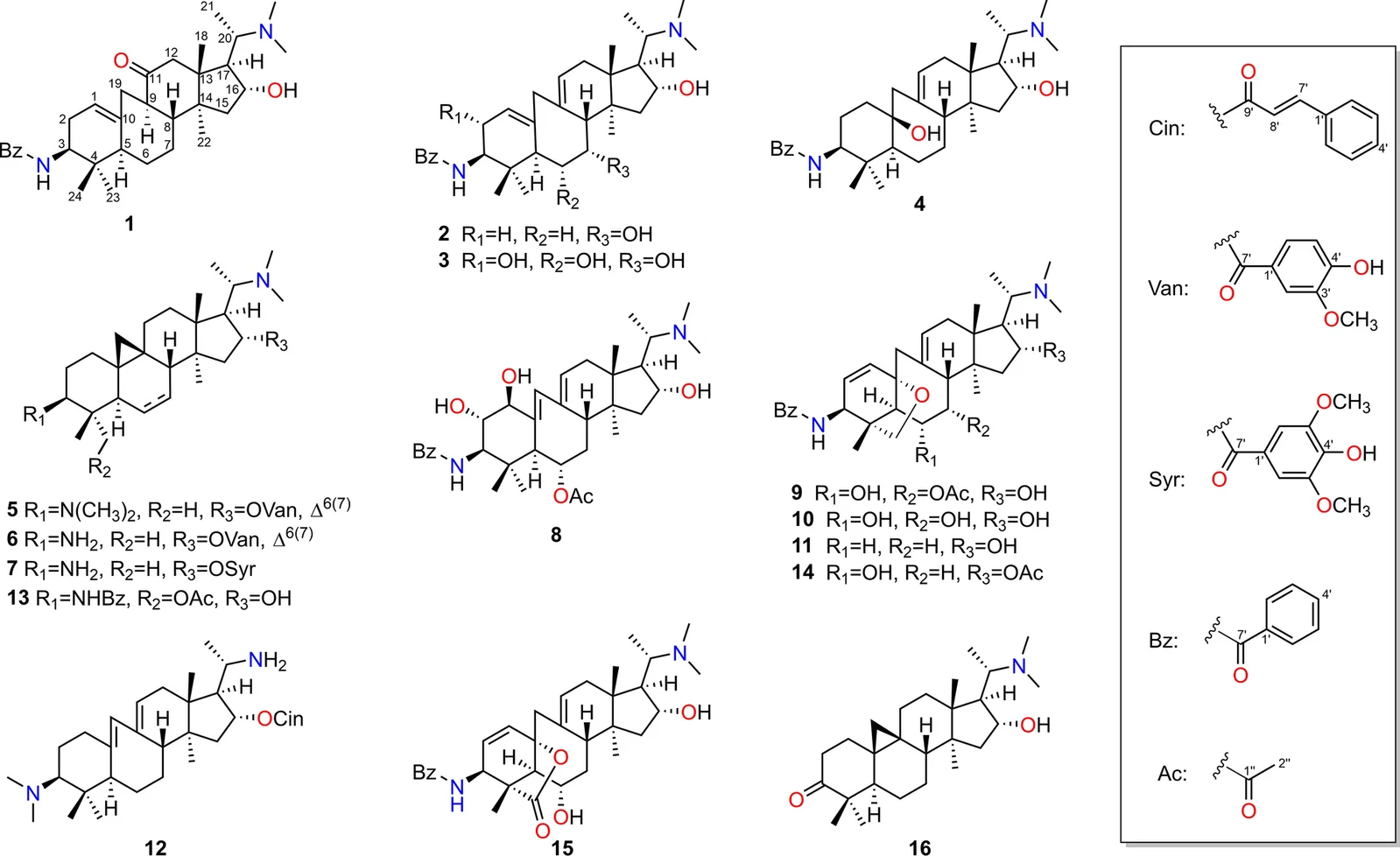
Structures of compounds **1**–**16**

*Buxus myrica* H. Lév. is an endemic species with a narrow distribution, native to southern China and northern Vietnam. Despite the profound chemical diversity generally harbored by the *Buxus* genus, no phytochemical or pharmacological studies on *B. myrica* have been reported to date. Driven by the fascinating chemical architecture and therapeutic potential of *Buxus* alkaloids [7, 8], we conducted the first systematic phytochemical investigation on the total alkaloid fraction derived from the twigs and leaves of *B. myrica*. This endeavor led to the isolation of 16 constituents, comprising 12 previously undescribed 9,19-cycloartane- and 9,10-secocycloartane-type triterpenoid alkaloids, named myribuxines A–L (**1**–**12**), along with four known analogues (**13**–**16**) (Fig. 1). Herein, we detail their isolation, purification, and structural elucidation. Additionally, we report their cytotoxicity against four human cancer cell lines (HepG2, A549, U251, and HCT-116) in vitro. Annexin V/7-AAD double staining was performed to evaluate the apoptosis-inducing ability of **2**. Further network pharmacology and molecular docking were conducted to explore the potential target of **2.**

## Results and discussion

### Structural elucidation

**Fig. 2:**
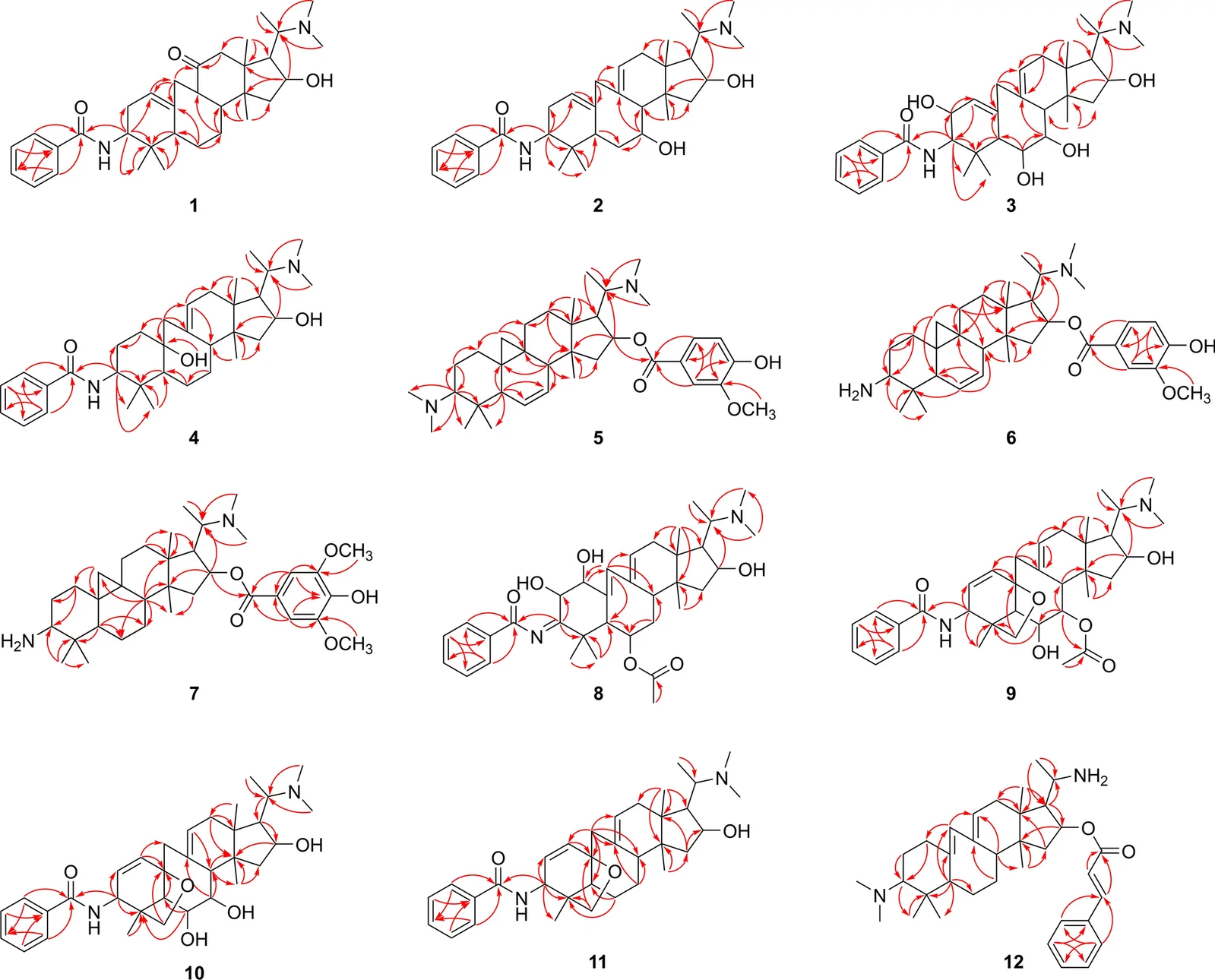
Key HMBC correlations of **1**–**12**

**Fig. 3:**
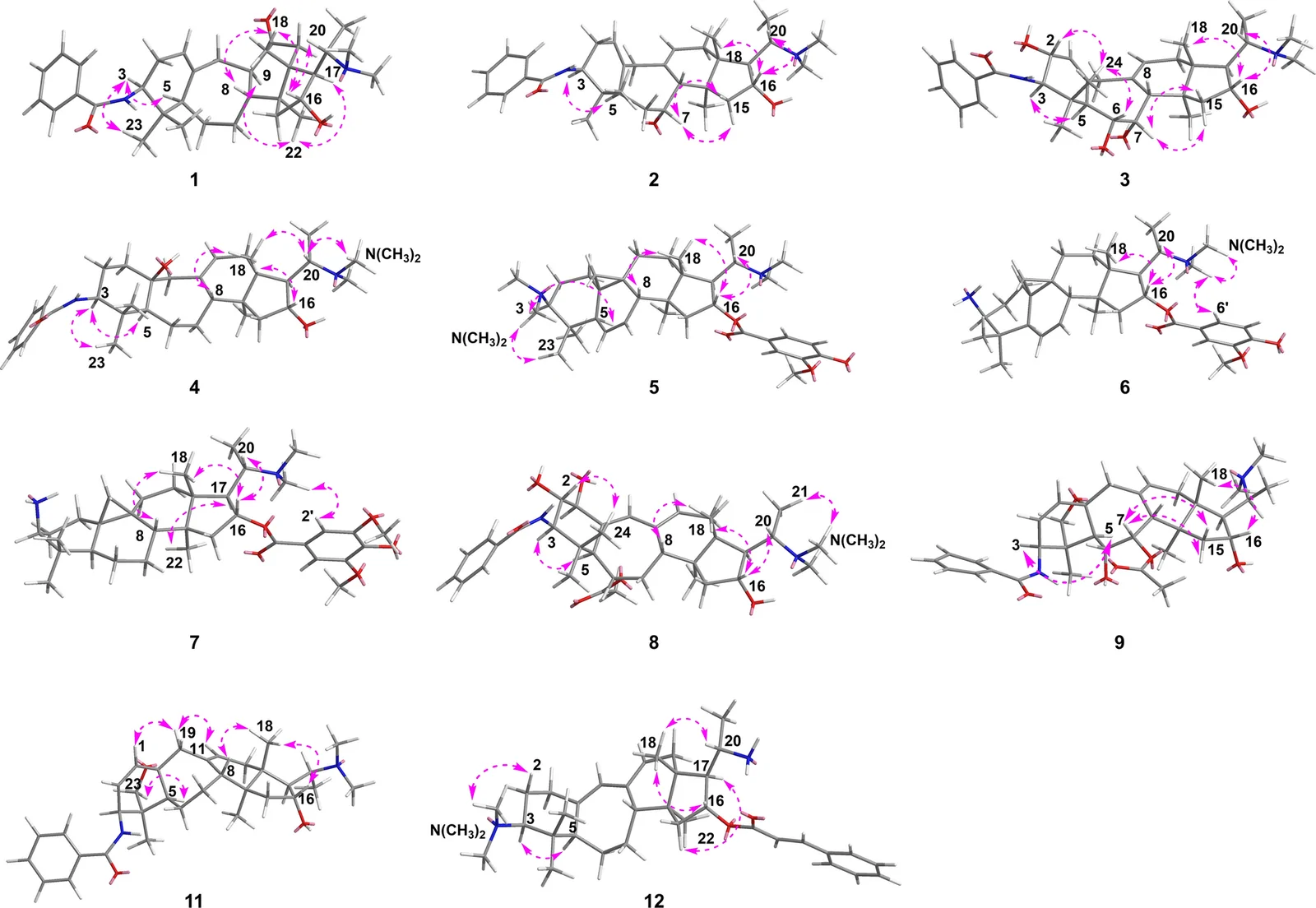
Key ROESY (**1**) and NOESY (**2**–**9**, **11**–**12**) correlations

Myribuxine A (**1**) was obtained as a pale-yellow amorphous powder. It was assigned the molecular formula C_33_H_48_N_2_O_3_ (corresponding to 11 degrees of unsaturation) based on its high-resolution electrospray ionization mass spectrometry (HRESIMS) [M + H]^+^ ion at *m/z* 521.3750 (calcd. for 521.3743) and nuclear magnetic resonance (NMR) data (Tables 1 and 2). The 1D NMR data combined with the HSQC spectrum of **1** revealed a ketone carbonyl (*δ*_C_ 212.8, C-11), a Δ^1(10)^ double bond [*δ*_H_ 5.52 (1H, m, H-1); *δ*_C_ 120.8 (C-1), 140.4 (C-10)], and a monosubstituted benzamido group [*δ*_H_ 7.79 (2H, m, H-2′,6′), 7.53 (1H, m, H-4′), 7.46 (2H, m, H-3′,5′); *δ*_C_ 170.7 (C-7′), 136.3 (C-1′), 132.5 (C-4′), 129.5 (C-3′,5′), 128.4 (C-2′,6′)]. Additional signals included an oxygenated methine [*δ*_H_ 4.36 (1H, td, *J *= 7.1, 1.8 Hz, H-16); *δ*_C_ 76.4 (C-16)], two *N*-methyl groups [*δ*_H_ 2.96, 2.84 (each 3H, s); *δ*_C_ 43.8, 36.4], and five methyl groups [*δ*_H_ 1.34 (3H, s, H_3_−22), 1.31 (3H, d, *J* = 6.6 Hz, H_3_−21), 1.03 (3H, s, H_3_−23), 0.80 (3H, s, H_3_−24), 0.78 (3H, s, H_3_−18)]. These signals indicated that **1** featured a 6/7/6/5 tetracyclic system incorporating a 9,10-secocycloartane skeleton. Comparison with hyrcanone [9] revealed that they had the same core, with the most notable discrepancy being that **1** possessed an oxygenated methine signal (*δ*_H_ 4.36, *δ*_C_ 76.4) at C-16, defining **1** as a hydroxylated derivative of hyrcanone. In the HMBC spectrum (Fig. 2), crosspeaks were observed between H-16 and C-13 (*δ*_C_ 48.6), C-14 (*δ*_C_ 48.5), and C-20 (*δ*_C_ 67.5), unambiguously locating the newly introduced hydroxy group at C-16. Furthermore, HMBC correlations from H-3 (*δ*_H_ 4.15) to C-7', and from H_2_−12 (*δ*_H_ 2.84, 2.36)/H_2_−19 (*δ*_H_ 3.20) to C-11, supported the attachment of a benzamido group at C-3 and the location of the ketone carbonyl group at C-11, respectively. The relative configuration of **1** was established through a detailed ROESY analysis (Fig. 3). Based on the biogenetically *α*-axial H-5 (*δ*_H_ 2.07), correlations of H-5 with H-3 (*δ*_H_ 4.15) and H_3_−23 (*δ*_H_ 1.03), together with those of H_3_−22 (*δ*_H_ 1.34) with H-9 (*δ*_H_ 2.15) and H-17 (*δ*_H_ 2.42), confirmed their co-facial α-orientations, while mutual correlations among H-8 (*δ*_H_ 1.75), H-16 (*δ*_H_ 4.36), H_3_−18 (*δ*_H_ 0.78), and H-20 (*δ*_H_ 3.58) demonstrated their co-facial *β*-orientations, which unambiguously defined the 16*α*-hydroxy configuration. Thus, the structure of **1** was established as depicted in Fig. 1.

**Table 1:** ^13^C NMR data of **1**–**12**

No	1^*a*^	2^*a*^	3^*b*^	4^*c*^	5^*b*^	6^*b*^	7^*a*^	8^*b*^	9^*a*^	10^*a*^	11^*a*^	12^*b*^
1	120.8	121.0	130.9	41.5	30.5	29.0	32.8	81.1	132.9	133.1	133.5	40.1
2	30.4	30.2	68.2	24.8	21.6	25.8	27.1	73.1	130.3	130.0	130.1	24.2
3	54.8	54.2	63.6	56.4	77.0	59.4	61.2	58.8	55.9	56.1	55.3	77.2
4	39.3	38.0	40.0	39.1	40.8	37.7	39.4	42.3	47.5	47.2	47.4	41.9
5	53.2	45.8	54.9	55.8	49.1	46.7	49.1	49.8	54.1	53.3	56.6	52.0
6	26.8	41.8	78.6	27.7	126.8	125.7	21.9	77.9	76.3	77.7	27.6	30.7
7	34.7	70.2	76.7	27.1	130.6	128.8	27.0	33.2	79.0	77.0	27.7	26.3
8	50.6	51.8	47.0	47.6	44.2	42.9	49.2	42.4	47.6	48.6	48.8	50.6
9	51.1	139.0	138.5	138.5	21.9	20.5	20.7	137.5	135.6	134.1	136.5	139.1
10	140.4	141.4	138.7	71.7	29.1	27.8	27.6	134.2	81.3	81.7	83.1	133.2
11	212.8	120.6	119.8	120.5	25.8	24.4	27.0	131.2	127.5	126.4	124.8	131.4
12	51.0	37.3	37.0	36.7	33.2	31.9	33.1	38.3	37.4	37.6	38.0	39.1
13	48.6	45.2	46.3	43.2	48.2	47.0	48.3	45.6	45.4	45.0	45.1	45.4
14	48.5	48.5	48.4	47.0	50.4	49.1	48.5	48.5	48.7	48.7	49.2	48.4
15	45.9	43.8	45.9	42.2	43.8	42.3	46.6	45.2	43.1	43.2	44.0	45.9
16	76.4	79.7	76.7	77.4	80.3	79.0	80.7	76.6	79.3	79.7	79.0	76.8
17	54.4	56.4	54.8	56.1	54.7	53.3	55.2	54.8	56.5	56.6	56.0	48.4
18	18.1	16.5	16.2	16.5	15.9	14.5	19.6	16.6	16.8	16.8	17.0	17.3
19	38.4	47.9	46.3	52.8	18.4	17.3	30.5	131.3	45.2	45.4	45.8	131.9
20	67.5	64.0	68.0	61.4	65.9	64.6	66.1	67.9	63.6	63.7	65.7	61.1
21	11.6	10.3	11.5	9.5	12.0	10.6	11.4	11.4	10.1	10.1	10.7	21.4
22	19.1	20.3	20.0	18.1	18.5	17.0	20.4	18.6	19.5	20.6	18.9	18.1
23	25.0	28.4	28.5	27.7	25.0	24.1	25.6	25.2	78.3	79.0	78.3	23.8
24	14.5	23.1	17.7	16.4	16.2	13.9	14.9	16.9	21.8	21.9	18.9	15.1
3-N(Me)_2_					47.0 40.6							47.2 40.9
20-N(Me)_2_	43.8 36.4	45.8	43.7 36.6	40.0	44.2 36.5	42.7 35.1	44.2 36.6	43.7 36.7	40.1	40.6	40.1	
1'	136.3	136.2	136.6	135.4	122.6	121.0	121.2	136.4	133.0	135.6	135.9	136.4
2'	128.4	128.1	128.4	127.4	114.0	112.2	108.2	128.5	128.4	128.3	128.5	128.8
3'	129.5	129.7	129.4	128.1	149.0	147.6	149.1	129.4	129.7	129.7	129.6	129.9
4'	132.5	132.7	132.4	130.8	153.4	152.0	142.6	132.5	132.9	132.9	132.7	130.8
5'	129.5	129.7	129.4	128.1	116.2	114.8	149.1	129.4	129.7	129.7	129.6	129.9
6'	128.4	128.1	128.4	127.4	125.1	123.6	108.2	128.5	128.4	128.3	128.5	128.8
7'	170.7	169.7	171.8	166.3	167.6	166.4	167.8	171.8	169.9	169.7	170.7	141.5
8'												122.6
9'												167.6
3'-OMe					56.7	55.1	56.9					
5'-OMe							56.9					
Ac-1''								172.5	172.8			
Ac-2''								21.6	21.7			

^*a*^^13^C NMR spectra recorded at 150 MHz in CD_3_OD, ^*b*13^C NMR spectra recorded at 100 MHz in CD_3_OD, ^*c*13^C NMR spectra recorded at 150 MHz in DMSO-*d*_6_

**Table 2:** ^1^H NMR data of **1**–**6**

No	1^*a*^	2^*a*^	3^*b*^	4^*c*^	5^*b*^	6^*b*^
1	5.52 m	5.48 t (3.9)	5.53 s	1.60 dt (13.8, 3.3) 1.49 m	1.74 m	1.77 m 1.63 m
2	2.15 m	2.42 m 2.13 m	4.10 brd (9.2)	1.95 m 1.23 m	2.12 dd (12.4, 3.5) 1.84 m	1.95 m 1.77 m
3	4.15 dd (10.6, 6.7)	4.04 t (5.2)	3.88 d (9.6)	3.78 ddd (12.8, 9.3, 3.5)	3.24 dd (12.6, 3.7)	3.04 dd (11.5, 4.3)
4						
5	2.07 m	2.33 m	2.50 dt (8.7, 2.5)	1.08 dd (12.3, 3.6)	2.06 m	2.08 m
6	1.91 m 1.31 m	1.64 m 2.25 dt (13.7, 5.7)	3.53 dd (8.6, 3.1)	1.95 m 1.54 q (11.5)	5.68 d (10.6)	5.73 d (10.6)
7	1.96 m 1.49 m	4.17 dd (5.9, 2.0)	3.96 d (3.0)	1.45 m 1.29 m	5.55 ddd (9.9, 5.8, 2.9)	5.55 ddd (10.1, 6.1, 3.0)
8	1.75 td (9.6, 5.1)	2.17 m	2.00 m	2.21 d (11.7)	2.70 d (6.0)	2.73 m
9	2.15 m					
10						
11		5.33 m	5.34 m	5.13 m	1.94 m 1.52 m	1.95 m 1.57 m
12	2.84 d (15.7) 2.36 d (15.7)	2.13 m 1.83 m	2.22 m 1.89 dd (16.8, 5.5)	1.95 m 1.73 m	1.84 m 1.57 m	1.95 m 1.57 m
13						
14						
15	2.15 m 1.58 dd (13.8, 1.8)	1.91 dd (13.5, 2.7) 1.47 dd (13.5, 2.7)	2.04 dd (13.4, 9.0) 1.56 m	1.83 dd (13.4, 9.8) 1.23 m	2.34 dd (14.6, 8.1) 1.45 d (14.6)	2.36 dd (14.9, 8.2) 1.49 d (14.6)
16	4.36 td (7.1, 1.8)	4.14 m	4.33 td (7.6, 3.7)	4.00 ddd (9.6, 6.8, 2.3)	5.31 t (7.3)	5.31 t (7.0)
17	2.42 dd (11.1, 7.2)	1.97 dd (10.9, 7.4)	2.25 m	1.73 m	2.67 m	2.73 m
18	0.78 s	0.75 s	0.76 s	0.72 s	1.11 s	1.13 s
19	3.20 m	3.29 m 2.97 d (13.9)	3.49 m 2.86 d (13.7)	2.47 m 2.12 d (15.6)	0.79 d (4.3) 0.03 d (4.3)	0.81 d (4.2) 0.01 d (4.2)
20	3.58 m	2.80 dq (13.2, 6.7)	3.58 m	2.57 dq (12.7, 6.4)	3.70 dd (11.4, 6.5)	3.70 dq (13.2, 6.5)
21	1.31 d (6.6)	0.97 d (6.5)	1.32 d (6.6)	0.83 d (6.4)	1.38 d (6.5)	1.38 d (6.4)
22	1.34 s	1.18 s	1.22 s	0.81 s	0.97 s	1.08 s
23	1.03 s	1.04 s	1.24 s	0.81 s	1.24 s	1.13 s
24	0.80 s	1.00 s	1.06 s	0.94 s	0.98 s	0.88 s
10-OH				3.65 brs		
3-NH				7.90 d (9.3)		
3*-*N(Me)_2_					3.00 brs 2.82 brs	
20*-*N(Me)_2_	2.96 brs 2.84 brs	2.38 brs	2.95 brs 2.81 brs	2.17 brs	2.92 brs 2.82 brs	2.93 brs 2.81 brs
1'						
2'	7.79 m	7.72 m	7.84 m	7.81 m	7.54 d (1.8)	7.53 d (1.9)
3'	7.46 m	7.46 m	7.47 m	7.44 m		
4'	7.53 m	7.55 m	7.52 m	7.49 m		
5'	7.46 m	7.46 m	7.47 m	7.44 m	6.89 d (8.3)	6.86 d (8.2)
6'	7.79 m	7.72 m	7.84 m	7.81 m	7.57 dd (8.3, 1.8)	7.55 dd (8.3, 1.9)
7'						
3'-OMe					3.90 s	3.90 s

^*a*^^1^H NMR spectra recorded at 600 MHz in CD_3_OD, ^*b*1^H NMR spectra recorded at 400 MHz in CD_3_OD, ^*c*1^H NMR spectra recorded at 600 MHz in DMSO-*d*_6_

**Fig. 4:**
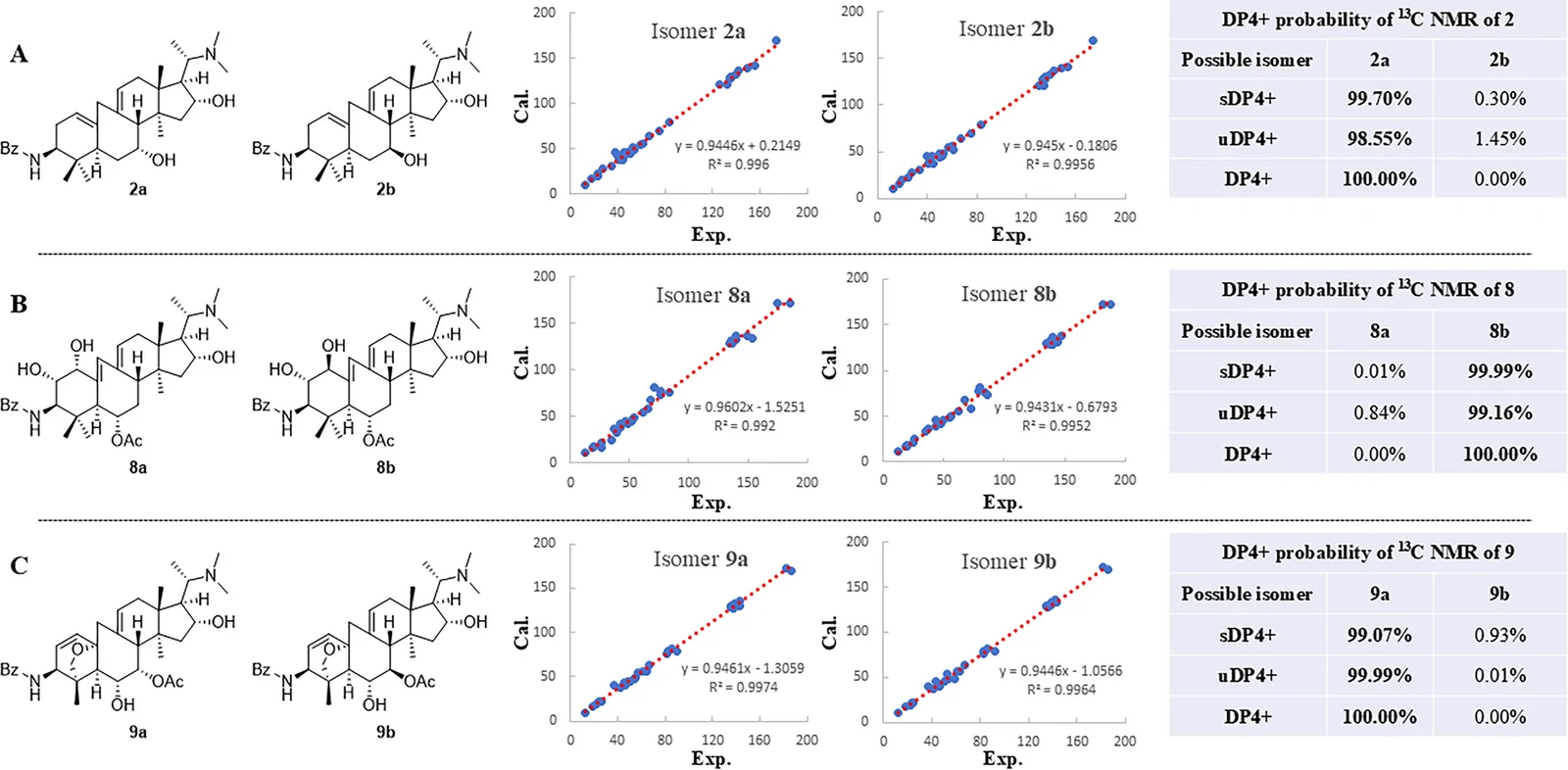
Structures, ^13^C NMR chemical shift correlation plots, and ^13^C DP4^+^ probabilities for the epimers of** 2** (**A**), **8** (**B**), and **9** (**C**)

Myribuxine B (**2**) exhibited a protonated molecular ion peak at *m*/*z* 521.3742 [M + H]^+^ in HRESIMS, corresponding to a molecular formula of C_33_H_48_N_2_O_3_ with 11 indices of hydrogen deficiency. Its NMR data (Tables 1 and 2) shared a high degree of similarity with those of (+)−16*R*-hydroxy-*N*_a_-benzoylbuxadine [10], while an additional oxygenated methine [*δ*_H_ 4.17 (dd, *J* = 5.9, 2.0 Hz), *δ*_C_ 70.2] was readily recognized in **2**. The HMBC correlations (Fig. 2) from H-7 (*δ*_H_ 4.17) to C-6 (*δ*_C_ 41.8), C-8 (*δ*_C_ 51.8), and C-9 (*δ*_C_ 139.0) allowed the assignment of the hydroxy group to C-7. The *α*-orientation of the hydroxy group was deduced from the NOESY correlation (Fig. 3) between H-7 (*δ*_H_ 4.17) and H_2_−15 (*δ*_H_ 1.91, 1.47) [11], and its small vicinal coupling constants with H_2_−6 (*J* = 5.9, 2.0 Hz), which indicated a *β*-pseudo-equatorial orientation for H-7. This was further corroborated by DP4^+^ probability analysis based on NMR chemical shift calculations for the two C-7 epimers, which assigned a 100% probability of all data to 7*α*-OH isomer (Fig. 4 and Additional file 1: Table S1). Thus, the structure of** 2** was defined as depicted.

Myribuxine C (**3**) was obtained as a pale-yellow amorphous powder. Its molecular formula was determined as C_33_H_48_N_2_O_5_ by HRESIMS at *m/z* 553.3670 ([M + H]^+^, calcd. for 553.3641), corresponding to 11 degrees of unsaturation. The ^1^H and ^13^C NMR data (Tables 1 and 2) closely resembled those of **2**, differing only in the presence of two additional hydroxylated methines assignable to C‑2 and C‑6. This was unambiguously confirmed by 2D NMR analysis. In the HMBC spectrum, the correlations from H-2 (*δ*_H_ 4.10) to C-1 (*δ*_C_ 130.9) and C-10 (*δ*_C_ 138.7), and from H-6 (*δ*_H_ 3.53) to C-5 (*δ*_C_ 54.9) and C-7 (*δ*_C_ 76.7) were observed. The *β*‑orientations of H‑2 and H‑6 were established by their NOESY correlations with H_3_‑24, which correspondingly defined the 2*α*,6*α*-dihydroxy configuration. Therefore, the structure of **3** was established.

Compound **4** was obtained as a pale-yellow amorphous powder and exhibited an [M + H]^+^ peak at *m/z* 523.3874 corresponding to the formula C_33_H_50_N_2_O_3_ (calcd. for 523.3900) with 10 degrees of unsaturation. The ^1^H and ^13^C NMR data (Tables 1 and 2) indicated that **4** was an analogue of buxsinine I [12]. Comparison of the NMR data of **4** with buxsinine I revealed prominent chemical shift differences at C‑23 (*δ*_C_ 64.7/*δ*_H_ 3.44, 3.03 for buxsinine I; *δ*_C_ 27.7/*δ*_H_ 0.81 for **4**), suggesting the replacement of the hydroxymethyl group with a methyl group. This was confirmed by HMBC correlations (Fig. 2) from H_3_−23 (*δ*_H_ 0.81) to C-4 (*δ*_C_ 39.1), C-5 (*δ*_C_ 55.8), and C-24 (*δ*_C_ 16.4). In the NOESY spectrum (Fig. 3), key correlations of H-5 (*δ*_H_ 1.08) with both H-3 (*δ*_H_ 3.78) and H_a_−19 (*δ*_H_ 2.47) confirmed their co-facial *α*-orientations, supporting the *β*‑orientation of 10‑OH. Accordingly, **4** was established as depicted and named myribuxine D.

Myribuxine E (**5**), a pale-yellow amorphous powder, possessed a molecular formula of C_36_H_54_N_2_O_4_, as deduced from its HRESIMS ion at *m/z* 579.4174 ([M + H]^+^, calcd. for 579.4162). The ^1^H and ^13^C NMR data (Tables 1 and 2) were highly analogous to those of cyclovirobuxeine A [13], with the most notable discrepancy being that **5** possessed a vanilloyloxy moiety instead of a hydroxy group at C-16. The HMBC correlations (Fig. 2) from H-16 (*δ*_H_ 5.31) to C-14 (*δ*_C_ 50.4), C-20 (*δ*_C_ 65.9), and C-7′ (*δ*_C_ 167.6) supported the location of the vanilloyloxy moiety at C-16. Additionally, the Δ^6(7)^ double bond was confirmed by HMBC correlations from H-6 (*δ*_H_ 5.68) and H-7 (*δ*_H_ 5.55) to C-5 (*δ*_C_ 49.1) and C-8 (*δ*_C_ 44.2). In the NOESY spectrum, key correlations of H-16 (*δ*_H_ 5.31) with H_3_−18 and H-20 supported *β*-orientation of H-16, defining the 16*α*-vanilloyloxy configuration. Therefore, the structure was designated as myribuxine E.

Myribuxine F (**6**) was isolated as a pale-yellow amorphous powder. HRESIMS gave a molecular formula of C_34_H_50_N_2_O_4_ (*m/z* 551.3843 [M + H]^+^, calcd. for 551.3849). The NMR data of **6** were virtually superimposable on those of **5**, suggesting that the dimethylamino group at C‑3 in **5** was replaced by a primary amino group in **6**, as evidenced by the loss of two *N*‑methyl signals and the upfield shift from *δ*_C_ 77.0 to *δ*_C_ 59.4 at C-3. In the NOESY spectrum, key cross-peaks of H-16 (*δ*_H_ 5.31) with H_3_−18 (*δ*_H_ 1.13) and H-20 (*δ*_H_ 3.70), as well as H-3 (*δ*_H_ 3.04) with H-5 (*δ*_H_ 2.08), confirmed that **6** possessed the same relative configuration as **5**. Therefore, the structure was identified as myribuxine F.

Myribuxine G (**7**) was obtained as a pale-yellow amorphous powder, and its molecular formula was deduced as C_35_H_54_N_2_O_5_ on the basis of the HRESIMS data with an [M + H]^+^ ion at *m/z* 583.4105 (calcd. for 583.4111). The 1D NMR data (Tables 1 and 3) were comparable to those of **6**, save for the disappearance of olefinic signals and an increase in the integration of the methoxy singlet from 3H to 6H. These observations indicated saturation of the Δ^6(7)^ double bond and replacement of the vanilloyloxy group at C-16 with a syringoyloxy group. The HMBC correlations (Fig. 2) from H-16 (*δ*_H_ 5.27) to C-7′ (*δ*_C_ 167.8) confirmed the existence of the syringoyloxy moiety at C-16. Key NOESY correlations (Fig. 3) of H-16 with H_3_−18 and H-20 established a *β*-oriented H-16. Thus, the structure of **7** was established.

**Table 3:** ^1^H NMR data of **7**–**12**

No	7^*a*^	8^*b*^	9^*a*^	10^*a*^	11^*a*^	12^*b*^
1	1.66 m 1.42 m	4.19 d (3.6)	5.86 dt (9.5, 1.3)	5.83 d (9.5)	5.88 ddd (9.5, 2.1, 1.0)	2.43 dt (13.3, 3.7) 2.30 m
2	1.85 m 1.76 m	3.62 m	5.96 dd (9.5, 3.9)	5.95 dd (9.5, 3.9)	5.81 dd (9.5, 3.6)	2.08 m 1.84 dd (12.7, 4.4)
3	2.99 m	4.31 d (10.7)	4.58 dd (3.9, 1.8)	4.54 dd (3.9, 1.7)	4.64 dd (3.7, 2.0)	3.29 m
4						
5	1.51 dd (12.3, 4.3)	3.17 d (5.9)	1.93 m	2.13 d (10.8)	1.53 dd (13.0, 4.1)	2.22 m
6	1.71 m 0.89 m	4.90 m	4.08 dd (11.0, 1.7)	3.81 dd (10.8, 1.5)	2.02 m 1.42 m	1.46 m 1.30 m
7	1.39 m 1.20 m	1.79 m 1.63 d (11.2)	5.41 d (1.7)	3.96 d (1.5)	1.65 m 1.60 m	1.57 dd (13.1, 6.8) 1.33 m
8	1.61 dd (12.6, 4.3)	2.22 m	2.20 m	1.99 m	2.04 m	2.10 m
9						
10						
11	2.22 m 1.28 m	5.70 m	5.54 dd (5.5, 2.6)	5.47 dd (5.5, 2.7)	5.47 m	5.63 m
12	1.98 d (11.4) 1.72 m	2.40 d (18.6) 2.04 m	2.10 m 1.86 m	2.09 m 1.85 dd (17.2, 5.3)	2.15 m 1.90 dd (16.6, 5.2)	2.30 m 2.06 m
13						
14						
15	2.22 m 1.71 m	2.22 m 1.49 d (13.4)	2.07 m 1.48 dd (13.7, 2.7)	1.99 m 1.44 dd (13.6, 2.8)	2.08 dd (13.7, 9.6) 1.45 m	2.03 dd (14.0, 8.6) 1.46 d (13.3)
16	5.27 t (7.3)	4.37 m	4.08 m	4.07 ddd (10.1, 7.3, 2.8)	4.20 t (8.6)	4.12 t (7.2)
17	2.81 m	2.22 m	1.93 m	1.94 m	1.98 m	1.92 dd (10.7, 6.4)
18	1.19 s	0.83 s	0.73 s	0.71 s	0.78 s	0.85 s
19	0.66 d (4.3) 0.51 d (4.3)	6.18 m	2.99 m 2.67 d (14.7)	3.01 m 2.57 d (14.4)	2.68 m	6.06 s
20	3.67 dq (12.9, 6.0)	3.59 m	2.71 m	2.69 dq (11.1, 6.7)	3.12 m	4.23 dd (10.7, 6.3)
21	1.39 d (6.6)	1.33 d (6.7)	0.93 d (6.6)	0.93 d (6.6)	1.12 d (6.3)	1.22 d (6.4)
22	1.25 s	0.97 s	0.95 s	1.18 s	0.97 s	0.94 s
23	1.05 s	0.90 s	3.68 d (8.6) 3.59 d (8.6)	3.68 d (8.6) 3.58 d (8.6)	3.67 d (8.4) 3.58 d (8.4)	1.20 s
24	0.92 s	0.81 s	1.32 s	1.35 s	1.16 s	0.91 s
3-N(Me)_2_						2.97 brs 2.78 brs
20-N(Me)_2_	2.94 brs 2.85 brs	2.96 brs 2.81 brs	2.26 brs	2.26 brs	2.55 brs	
1'						
2'	7.31 s	7.83 m	7.84 m	7.85 m	7.76 m	7.55 m
3'		7.46 m	7.49 m	7.49 m	7.47 m	7.37 m
4'		7.51 m	7.55 m	7.56 m	7.54 m	7.38 m
5'		7.46 m	7.49 m	7.49 m	7.47 m	7.37 m
6'	7.31 s	7.83 m	7.84 m	7.85 m	7.76 m	7.55 m
7'						7.52 d (15.9)
8'						6.61 d (15.9)
9'						
3'-OMe	3.89 s					
5'-OMe	3.89 s					
Ac-1''						
Ac-2''		2.04 s	2.11 s			

^*a*^^1^H NMR spectra recorded at 600 MHz in CD_3_OD, ^*b*1^H NMR spectra recorded at 400 MHz in CD_3_OD

Compound **8** showed an [M + H]^+^ ion at *m/z* 595.3742 (calcd. for 595.3747) in the HRESIMS spectrum, corresponding to the molecular formula C_35_H_50_N_2_O_6_. The NMR data (Tables 1 and 3) mirrored those of buxruguline E [14], except for the incorporation of an extra hydroxy group at C-1 manifested by the oxygenated methine at *δ*_C_ 81.1 and *δ*_H_ 4.19, and the replacement of the Δ^1(10)^ double bond with a conjugated Δ^9(11),10(19)^ diene system exhibiting H-19 at *δ*_H_ 6.18 and C-19 at *δ*_C_ 131.3. HMBC correlations (Fig. 2) from H-19 to C-1, C-5, C-8, and C-11, as well as from H-1 to C-19, established the conjugated diene framework and the C-1 location of the hydroxy group. Relative ring A stereochemistry was defined by ^3^*J*_H-H_ coupling constants and NOESY analysis. Key crosspeaks of H-3 (*δ*_H_ 4.31) with H-5 (*δ*_H_ 2.07) and H_3_−23 (*δ*_H_ 1.03) confirmed their co-facial *α*-orientations. The large coupling of H-3 (d, *J* = 10.7 Hz) indicated *β*-axial H-2 (*δ*_H_ 3.62), while the small coupling of H-1 (*δ*_H_ 4.19, d, *J* = 3.6 Hz) defined an equatorial H-1. DP4^+^ probability analysis on the C-1 epimers based on both ^1^H and ^13^C NMR data (Fig. 4 and Additional file 1: Table S4) further established the configuration at C-1, yielding a combined all data probability of > 99.9% for candidate **8b**. Accordingly, **8** was designated as myribuxine H.

Compound **9**, obtained as a pale-yellow amorphous powder, shared the same molecular formula of C_35_H_48_N_2_O_6_ with* O*^10^-obtusifuranamine-A [11], as deduced from HRESIMS at *m/z* 593.3558 ([M + H]^+^, calcd. for 593.3591). The key structural distinction between them was that the substituents at C-6 and C-7 were reversed. HMBC correlations of H-7 (*δ*_H_ 5.41) to C-6, C-8, C-9, and C-1′′ (*δ*_C_ 172.8), and of H-6 (*δ*_H_ 4.08) to C-4, C-5 assigned the acetoxy group to C-7 and the hydroxy group to C-6. HMBC correlations from H_2_−19 to C-1/C-11 and H_2_−23 (*δ*_H_ 3.68, 3.59) to C-4/C-10 confirmed the Δ^1(2)^ and Δ^9(11)^ double bonds and the extra tetrahydrofuran ring. Coupling constants of H-6 (*δ*_H_ 4.08, dd, *J *= 11.0, 1.7 Hz) and H-7 (*δ*_H_ 5.41, d, *J *= 1.7 Hz) established *β*-pseudo-axial H-6 and *β*-pseudo-equatorial H-7. Key NOESY crosspeaks of H-7 with H_2_−15 (*δ*_H_ 2.07/1.48) and H-16 (*δ*_H_ 4.08) with H_3_−18 (*δ*_H_ 0.73)/H-20 (*δ*_H_ 2.71) confirmed the 7*α*-OAc and 16*α*-OH relative configurations. The combined DP4^+^ probability analysis on the C-7 epimers corroborated this assignment (100% for **9a**, Fig. 4 and Additional file 1: Table S7). Accordingly, **9** was designated as myribuxine I.

Myribuxine J (**10**) was obtained as a pale-yellow amorphous powder. Its [M + H]^+^ ion at *m/z* 551.3457 corresponded to a molecular formula of C_33_H_46_N_2_O_5_, which had 12 degrees of unsaturation. The NMR data (Tables 1 and 3) closely resembled those of **9**, except that the acetoxy group signals were absent, and H-7 showed an upfield shift from *δ*_H_ 5.41 to 3.96, reflecting the presence of a hydroxy group rather than an acetoxy group at C-7. The HMBC correlations (Fig. 2) from H-7 (*δ*_H_ 3.96) to C-5 (*δ*_C_ 53.3), C-8 (*δ*_C_ 48.6), and C-9 (*δ*_C_ 134.1) confirmed the above assignment. The coupling constants of H-6 (*J *= 10.8, 1.5 Hz) and H-7 (*J *= 1.5 Hz) matched those of **9**, indicating the 6*α*,7*α*-dihydroxy relative configuration. Thus, the structure of **10** was established.

Myribuxine K (**11**) was purified as a pale-yellow amorphous powder with the molecular formula C_33_H_46_N_2_O_3_, as determined by HRESIMS data at *m/z* 519.3581 ([M + H]^+^, calcd. for 519.3587). The NMR data (Tables 1 and 3) were highly similar to those of *O*^10^-natafuranamine [15], differing by the replacement of a hydroxylated methine with a methylene group at C-6. The HMBC correlations from H_2_−6 (*δ*_H_ 2.02, 1.42) to C-4 (*δ*_C_ 47.4) and C-5 (*δ*_C_ 56.6) were observed. Key NOESY correlations of H-5 with H-3/H_3_−23 (Fig. 3), alongside those among H-8, H-16, H_3_−18, and H-20, defined the overall relative stereochemistry. Accordingly, **11** was identified as myribuxine K.

Compound **12**, based on HRESIMS data at *m/z* 531.3928 ([M + H]^+^, calcd. for 531.3951) was determined to possess the molecular formula C_35_H_50_N_2_O_2_, corresponding to 12 degrees of unsaturation. The NMR data (Tables 1 and 3) indicated that **12** shared the same skeleton as buxaminol E [13], except that the hydroxy group at C‑16 was replaced by a cinnamoyloxy moiety. Characteristic ester resonances included H-7′ (*δ*_H_ 7.52, 1H, d, *J *= 15.9 Hz), H-8′ (*δ*_H_ 6.61, 1H, d, *J *= 15.9 Hz), aromatic protons (*δ*_H_ 7.55, 2H, m; 7.38, 1H, m; 7.37, 2H, m), and an ester carbonyl at *δ*_C_ 167.6 (C-9′). The *trans* geometry of the conjugated double bond was deduced from the large coupling constant between H-7′ and H-8′ (*J *= 15.9 Hz). The 16*α*-cinnamoyloxy configuration was supported by the NOESY correlation between H-16 and H_3_−18. Consequently, **12** was unambiguously identified as myribuxine L.

By comparing their spectroscopic data with those reported in the literature, the known *Buxus* alkaloids were identified as cyclobuxusinine H (**13**) [16], *O*^10^-buxafuranamine (**14**) [17], buxusemine B (**15**) [18], and (−)-cyclomikuranine (**16**) [19], respectively.

### Plausible biosynthetic pathways

**Scheme 1.**
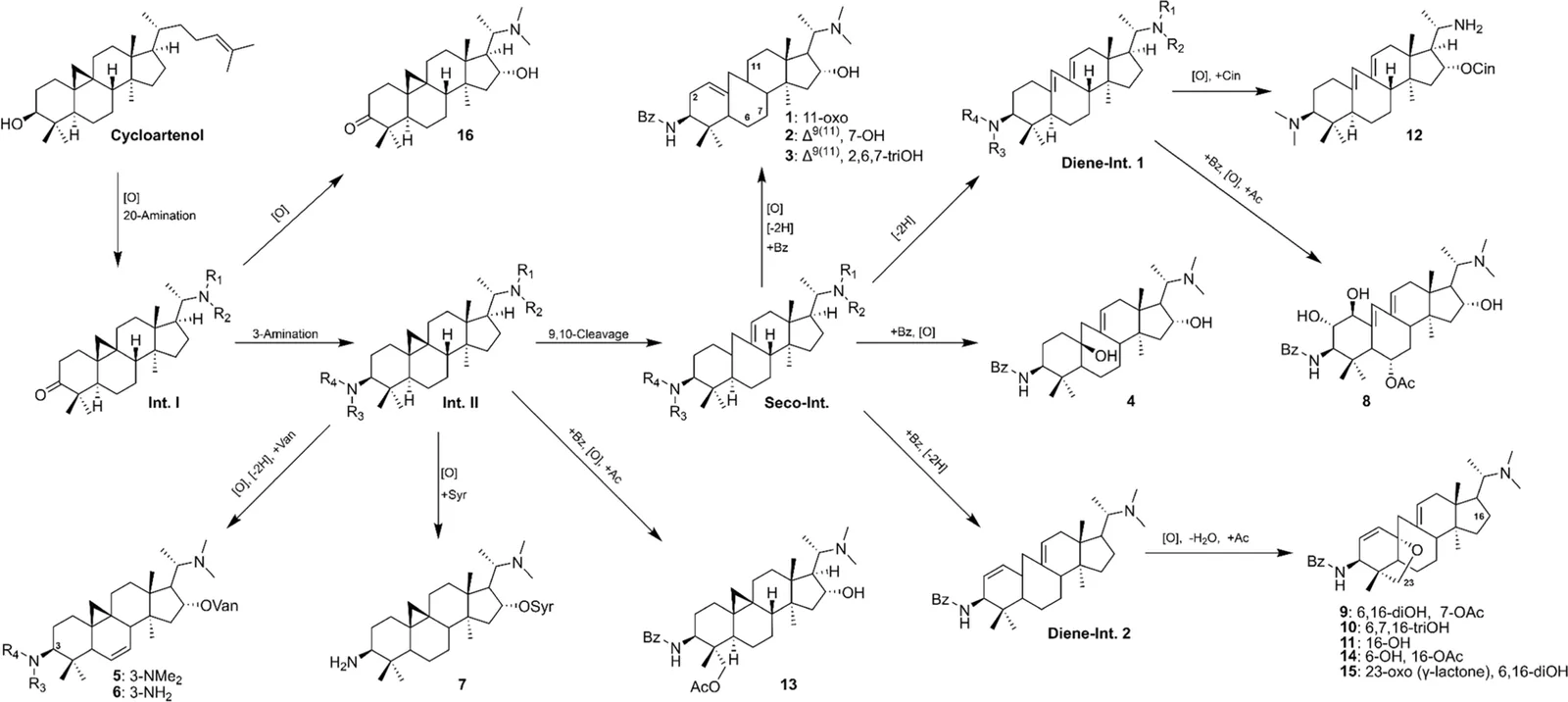
Proposed biosynthetic pathways for compounds **1**–**16**

Biogenetically, alkaloids **1–16** originate from the canonical triterpenoid precursor cycloartenol (Scheme 1). The structural diversification initiates through oxidative side-chain cleavage, C-20 transamination, and C-3 oxidation to generate Int. I. Regioselective C-16 hydroxylation of Int. I directly yields the mononitrogen derivative **16**. Subsequent C-3 transamination of Int. I establishes the universal 3,20-diaminocycloartane core (Int. II), from which the pathway diverges into two major lineages.

In Pathway A, the pristine cyclopropane core is preserved throughout downstream tailoring. Specifically, enzymatic Δ^6(7)^-desaturation, C-16 hydroxylation, and subsequent vanilloylation afford **5** and **6**. Alternatively, direct C-16 syringoylation on the saturated framework generates **7**, whereas C-3 benzoylation coupled with C-23 acetylation produces **13**.

Pathway B encompasses the 9,10-secocycloartane alkaloids, initiated by enzymatic oxidative cleavage of the strained C-9/C-10 bond in Int. II to form Seco-Int. In one cascade, C-3 benzoylation and A-ring Δ^1(10)^-desaturation establish the baseline framework, yielding the 11-oxo alkaloid **1** as well as the Δ^1(10),9(11)^-diene derivatives **2** and **3**. In another, C-3 benzoylation along with C-10 and C-16 hydroxylations furnishes **4**. In a third, Δ^10(19)^-desaturation establishes the Δ^9(11),10(19)^-conjugated diene core (Diene-Int. 1) to yield **8** and **12**. Finally, C-3 benzoylation and Δ^1(2)^-desaturation establish Diene-Int. 2, which undergoes intramolecular dehydrative etherification across C-10 and C-23 to produce the rigid tetrahydrofuran-fused alkaloids **9**–**11** and **14**, while further C-23 oxidation drives intramolecular lactonization to afford the γ-lactone analogue **15**.

### Cytotoxicity

The isolated alkaloids were screened for in vitro cytotoxicity against HepG2, A549, U251, and HCT‑116 using the MTT assay (Table 4). Compounds **9**, **11**, **13**, and **15** were inactive (IC_50 _> 50 μM), while the others exhibited modest inhibitory effects (IC_50 _= 17.5 ± 2.2 to 47.3 ± 1.3 μM). In particular, **2** suppressed the proliferation of HepG2 cells with an IC_50_ value of 24.7 ± 2.0 μM. Although its activity was lower than that of the clinical positive controls (sorafenib: 3.1 ± 0.8 μM; cisplatin: 8.5 ± 0.6 μM), **2** was selected for subsequent apoptosis evaluation in HepG2 cells, primarily considering its sufficient isolation yield together with the cytotoxic profiles of all isolates across four tumor cell lines.

**Table 4:** Cytotoxicities of all compounds against four cell lines (IC_50_, μM)

Compounds	HepG2	A549	U251	HCT-116
**1**	> 50	35.3 ± 1.4	> 50	26.8 ± 1.5
**2**	24.7 ± 2.0	> 50	36.2 ± 2.1	> 50
**3**	22.7 ± 1.3	34.5 ± 1.4	46.3 ± 1.8	33.6 ± 1.3
**4**	26.5 ± 1.2	19.1 ± 1.0	> 50	47.3 ± 1.3
**5**	> 50	38.4 ± 2.3	37.1 ± 2.2	> 50
**6**	28.2 ± 1.6	37.4 ± 1.5	29.3 ± 1.7	> 50
**7**	18.4 ± 1.3	> 50	29.1 ± 1.4	> 50
**8**	39.2 ± 2.4	> 50	28.1 ± 2.3	17.5 ± 2.2
**9**	> 50	> 50	> 50	> 50
**10**	33.5 ± 1.9	42.1 ± 1.8	> 50	24.8 ± 2.0
**11**	> 50	> 50	> 50	> 50
**12**	28.2 ± 2.3	> 50	> 50	> 50
**13**	> 50	> 50	> 50	> 50
**14**	31.6 ± 1.8	42.9 ± 1.9	36.7 ± 2.0	> 50
**15**	> 50	> 50	> 50	> 50
**16**	45.4 ± 2.0	38.8 ± 2.1	> 50	34.2 ± 1.9
**Cisplatin**	8.5 ± 0.6	27.2 ± 0.5	13.9 ± 0.7	7.8 ± 0.4
**Sorafenib**	3.1 ± 0.8	N.D	N.D	N.D
**Temozolomide**	N.D	N.D	13.1 ± 2.3	N.D
**Oxaliplatin**	N.D	N.D	N.D	4.5 ± 1.6

The data were expressed as means ± SEM (n=3). N.D. = not determined

### Compound 2 induced apoptosis in HepG2 cells

**Fig. 5:**
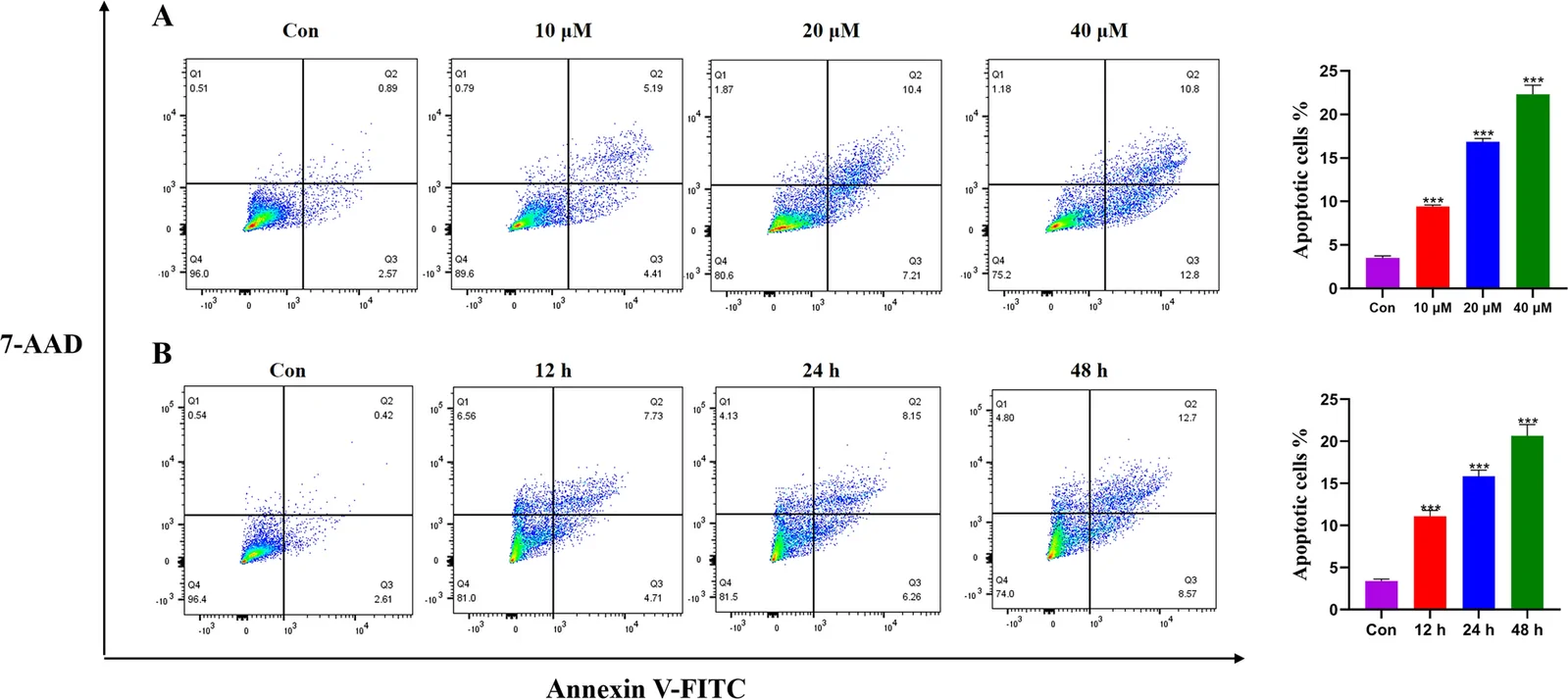
Compound **2** induces apoptosis in HepG2 cells. (**A**) Flow cytometric analysis and quantification of apoptosis after treatment with 0, 10, 20, and 40 μM of **2** for 48 h. (**B**) Flow cytometric analysis and quantification after treatment with 20 μM of **2** for 0, 12, 24, and 48 h. Data are expressed as mean ± SD (n=3). ***P < 0.001 versus control group

As shown in Fig. 5, compound **2** induced apoptosis in HepG2 cells in a dose- and time-dependent manner. The apoptosis rates increased from 3.52 ± 0.24% (control) to 9.43 ± 0.17% (10 μM), 16.86 ± 0.38% (20 μM), and 22.32 ± 1.04% (40 μM) after 48 h. Similarly, when HepG2 cells were treated with 20 μM of **2** at different durations, the apoptosis rates rose from 3.39 ± 0.22% (0 h) to 11.09 ± 0.69% (12 h), 15.85 ± 0.72% (24 h), and 20.65 ± 1.34% (48 h), respectively.

### Computational prediction of anti-liver cancer targets for 2 

**Fig. 6:**
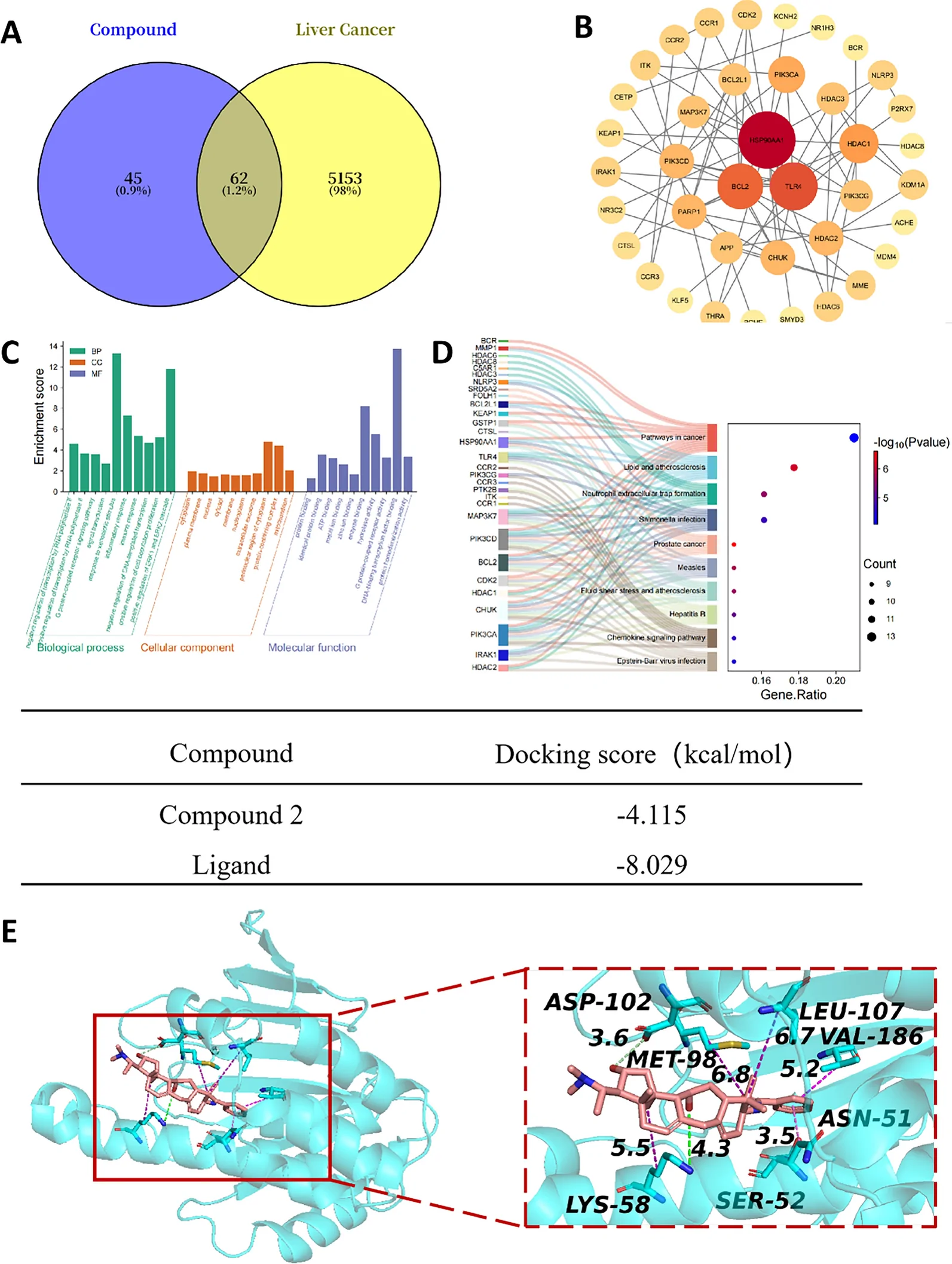
Screening and molecular docking of anti-liver cancer targets for **2**. **A** targets of **2** and liver cancer. **B** PPI network of the overlapping targets. **C** GO functional enrichment analysis of overlapping targets. **D** Sankey pathway enrichment analysis. The embedded table lists the docking scores of **2** and the reference ligand against HSP90α. **E** Binding mode of **2** with HSP90α (PDB: 6GQ6)

To elucidate the molecular mechanism by which **2** induces apoptosis, we adopted an integrated strategy combining network pharmacology and molecular docking to screen its potential anti-liver cancer targets. We cross-referenced putative targets of **2** from the SuperPred and SwissTargetPrediction databases with liver cancer-related targets collected from OMIM, GeneCards and DisGeNET, and finally obtained 62 overlapping targets (Fig. 6A). Topological analysis of the constructed PPI network indicated that HSP90α (heat shock protein 90 alpha family class A member 1), a molecular chaperone, presented the highest node degree. Comprehensive evaluation of multiple topological indices further indicated that this protein acts as a vital hub node (Fig. 6B). Functional enrichment analyses demonstrated that the overlapping targets were predominantly enriched in biological processes such as inflammatory response, ERK signaling cascade and cell proliferation regulation, and were also significantly involved in tumor- and inflammation-associated pathways including Pathways in cancer, Chemokine signaling pathway and Hepatitis B pathway (Fig. 6C, D).

Molecular docking was performed to explore the preliminary binding mode of **2** within HSP90α (PDB ID: 6GQ6, Fig. 6E). Compound **2** displayed a relatively modest docking score of − 4.115 kcal/mol, which is substantially less favorable than that of the co-crystallized synthetic inhibitor. In the docked pose, the 3-benzamido moiety lies 3.5 Å away from the side chain of Ser52. The ligand forms a polar contact with Asp102 at 3.6 Å, and a long-range contact with Lys58 at 4.3 Å.

## Conclusion

To summarize, this work represents the first phytochemical investigation of the total alkaloid fraction derived from *B. myrica*, leading to the isolation of 12 novel myribuxines (**1**–**12**) and four known analogues (**13**–**16**), which expands the known structural diversity of *Buxus* alkaloids. In subsequent cellular evaluations, **2** exhibited modest cytotoxicity and induced apoptosis in HepG2 cells. It should be noted that network pharmacology and molecular docking provide a theoretical working hypothesis implicating HSP90α, which requires future experimental validation. Ultimately, this study provides a solid foundation for the future discovery and evaluation of biologically active alkaloids from *B. myrica*.

## Experimental

### General experimental procedures

Optical rotations and ultraviolet (UV) spectra were measured in methanol (MeOH) using an MCP 200 polarimeter (Anton Paar, Graz, Austria) and a UV-2600i spectrophotometer (Shimadzu, Tokyo, Japan), respectively. HRESIMS data were acquired on either a compact quadrupole time-of-flight (Q-TOF) (Bruker Daltonics, Billerica, MA, USA) or a 6550 iFunnel Q-TOF (Agilent Technologies, Santa Clara, CA, USA). 1D and 2D NMR spectra were recorded on AV-400 or AV-600 spectrometers (Bruker BioSpin, Rheinstetten, Germany). Column chromatography (CC) was performed using silica gel (100–200 and 200–300 mesh, Qingdao Marine Chemical Co., Ltd., Qingdao, China) and reversed-phase C_18_ silica gel (RP-C_18_, 50 μm, YMC Co., Ltd., Japan). Semi-preparative high-performance liquid chromatography (semi-prep-HPLC) was carried out on a Shimadzu LC-6AD liquid chromatography system equipped with an SPD-20A detector, utilizing a YMC RP-C_18_ column (250 mm × 10 mm, 5 µm).

### Plant material

The twigs and leaves of *Buxus myrica* H. Lév. (Buxaceae) were collected from Guizhou Province, China, and authenticated by Professor Jingming Jia. A voucher specimen (No. 20220908) has been deposited in the Key Laboratory of Structure-Based Drug Design and Discovery, Ministry of Education, Shenyang Pharmaceutical University.

### Extraction and isolation

The air-dried and powdered plant material of *B*. *myrica* (26 kg) was extracted three times with 70% aqueous ethanol (solid-to-liquid ratio 1:8, w/v) at 90 °C for 2 h each time. The combined extracts were concentrated under reduced pressure to yield a crude residue. This residue was suspended in water, adjusted to pH 2–3 with 1% aqueous HCl, and partitioned with ethyl acetate (EtOAc, 4 × equal volumes). The combined EtOAc layers were evaporated *in vacuo* to yield a non-alkaloidal fraction (150 g). The remaining acidic aqueous phase was basified to pH 9–10 with aqueous ammonium hydroxide and subsequently partitioned with dichloromethane (CH_2_Cl_2_, 4 × equal volumes). The combined organic layers were concentrated *in vacuo* to afford the total alkaloid fraction (133 g).

The total alkaloid fraction was subjected to silica gel CC, eluting with a step gradient of CH_2_Cl_2_/MeOH (100:0→0:100, *v*/*v*) to afford seven primary fractions (Frs. A–G). Fraction C was subjected to RP-C_18_ CC using a MeOH/H_2_O gradient (0:100→100:0, *v*/*v*) to yield 76 subfractions (Frs. C1–C76). Fr. C14 was purified by semi-prep-HPLC [MeOH/H_2_O, 37:63, *v*/*v*, containing 0.01% trifluoroacetic acid (TFA)] to afford **7** (5.1 mg), **9** (4.2 mg), and **15** (10.3 mg). Fr. C17 was processed under identical conditions to yield **10** (4.2 mg) and **13** (6.4 mg). The combined subfractions C21–C28 were resolved by semi-prep-HPLC (MeOH/H_2_O, 80:20, *v*/*v*, containing 0.01% diethylamine) to yield **1** (7.6 mg) and **2** (35 mg). Fr. C66 was purified by semi-prep-HPLC (MeOH/H_2_O, 45:55, *v*/*v*, containing 0.01% TFA) to give **16** (12.4 mg). Additionally, the combined Frs. C68–C69 were subjected to semi-prep-HPLC (MeOH/H_2_O, 47:53, *v*/*v*, containing 0.01% TFA) to provide **4** (6.4 mg) and **8** (4.8 mg).

Fr. E was subjected to RP-C_18_ CC (MeOH/H_2_O, 0:100→100:0, *v*/*v*) to obtain 66 subfractions (Frs. E1–E66). Fr. E26 was purified by semi-prep-HPLC (MeOH/H_2_O, 43:57, *v*/*v*, containing 0.01% TFA) to yield **3** (4.3 mg). The combined Frs. E33–E38 were initially fractionated by semi-prep-HPLC (MeOH/H_2_O, 50:50, *v*/*v*, containing 0.01% TFA). The resulting peaks were individually purified using a modified mobile phase (MeOH/H_2_O, 45:55,* v*/*v*, containing 0.01% TFA) to afford **5** (3.9 mg), **6** (3.1 mg), **11** (4.5 mg), and **14** (5.7 mg). Finally, semi-prep-HPLC of Fr. E60 (MeOH/H_2_O, 60:40, *v*/*v*, containing 0.01% TFA) gave **12** (3.5 mg).

### Spectroscopic data

Myribuxine A (**1**): pale-yellow, amorphous powder; ${[\alpha ]}_{\mathrm{D}}^{25}$ −26.32 (*c* 0.1, MeOH); UV (MeOH) *λ*_max_ (log *ε*) 201 (3.75), 225 (3.49) nm; ^1^H and ^13^C NMR data see Tables 1 and 2; (+)-HRESIMS *m/z* 521.3750 [M + H]^+^ (calcd. for C_33_H_49_N_2_O_3_, 521.3743).

Myribuxine B (**2**): pale-yellow, amorphous powder; ${[\alpha ]}_{\mathrm{D}}^{25}$ +160.26 (*c* 0.15, MeOH); UV (MeOH) *λ*_max_ (log *ε*) 202 (3.77), 225 (3.37) nm; ^1^H and ^13^C NMR data see Tables 1 and 2; (+)-HRESIMS *m/z* 521.3742 [M + H]^+^ (calcd. for C_33_H_49_N_2_O_3_, 521.3743).

Myribuxine C (**3**): pale-yellow, amorphous powder; ${[\alpha ]}_{\mathrm{D}}^{25}$ −17.15 (*c* 0.1, MeOH); UV (MeOH) *λ*_max_ (log *ε*) 207 (3.81), 225 (3.71) nm; ^1^H and ^13^C NMR data see Tables 1 and 2; (+)-HRESIMS *m/z* 553.3670 [M + H]^+^ (calcd. for C_33_H_49_N_2_O_5_, 553.3641).

Myribuxine D (**4**): pale-yellow, amorphous powder; ${[\alpha ]}_{\mathrm{D}}^{25}$ +46.32 (*c* 0.15, MeOH); UV (MeOH) *λ*_max_ (log *ε*) 203 (3.82), 225 (3.61) nm; ^1^H and ^13^C NMR data see Tables 1 and 2; (+)-HRESIMS *m/z* 523.3874 [M + H]^+^ (calcd. for C_33_H_51_N_2_O_3_, 523.3900).

Myribuxine E (**5**): pale-yellow, amorphous powder; ${[\alpha ]}_{\mathrm{D}}^{25}$ +34.01 (*c* 0.1, MeOH); UV (MeOH) *λ*_max_ (log *ε*) 206 (3.89), 220 (4.00), 262 (3.80), 292 (3.51) nm; ^1^H and ^13^C NMR data see Tables 1 and 2; (+)-HRESIMS *m/z* 579.4174 [M + H]^+^ (calcd. for C_36_H_55_N_2_O_4_, 579.4162).

Myribuxine F (**6**): pale-yellow, amorphous powder; ${[\alpha ]}_{\mathrm{D}}^{25}$ +74.68 (*c* 0.1, MeOH); UV (MeOH) *λ*_max_ (log *ε*) 208 (4.09), 222 (4.10), 265 (3.89), 293 (3.69) nm; ^1^H and ^13^C NMR data see Tables 1 and 2; (+)-HRESIMS *m/z* 551.3843 [M + H]^+^ (calcd. for C_34_H_51_N_2_O_4_, 551.3849).

Myribuxine G (**7**): pale-yellow, amorphous powder; ${[\alpha ]}_{\mathrm{D}}^{25}$ +62.76 (*c* 0.1, MeOH); UV (MeOH) *λ*_max_ (log *ε*) 219 (4.24), 275 (3.89) nm; ^1^H and ^13^C NMR data see Tables 1 and 3; (+)-HRESIMS *m/z* 583.4105 [M + H]^+^ (calcd. for C_35_H_55_N_2_O_5_, 583.4111).

Myribuxine H (**8**): pale-yellow, amorphous powder; ${[\alpha ]}_{\mathrm{D}}^{25}$ −45.77 (*c* 0.1, MeOH); UV (MeOH) *λ*_max_ (log *ε*) 202 (3.74), 246 (3.78) nm; ^1^H and ^13^C NMR data see Tables 1 and 3; (+)-HRESIMS *m/z* 595.3742 [M + H]^+^ (calcd. for C_35_H_51_N_2_O_6_, 595.3747).

Myribuxine I (**9**): pale-yellow, amorphous powder; ${[\alpha ]}_{\mathrm{D}}^{25}$ +80.11 (*c* 0.1, MeOH); UV (MeOH) *λ*_max_ (log *ε*) 203 (3.85), 224 (3.67) nm; ^1^H and ^13^C NMR data see Tables 1 and 3; (+)-HRESIMS *m/z* 593.3558 [M + H]^+^ (calcd. for C_35_H_49_N_2_O_6_, 593.3591).

Myribuxine J (**10**): pale-yellow, amorphous powder; ${[\alpha ]}_{\mathrm{D}}^{25}$ +38.56 (*c* 0.1, MeOH); UV (MeOH) *λ*_max_ (log *ε*) 202 (3.81), 225 (3.44) nm; ^1^H and ^13^C NMR data see Tables 1 and 3; (+)-HRESIMS *m/z* 551.3457 [M + H]^+^ (calcd. for C_33_H_47_N_2_O_5_, 551.3485).

Myribuxine K (**11**): pale-yellow, amorphous powder; ${[\alpha ]}_{\mathrm{D}}^{25}$ +40.05 (*c* 0.1, MeOH); UV (MeOH) *λ*_max_ (log *ε*) 202 (3.87), 224 (3.61) nm; ^1^H and ^13^C NMR data see Tables 1 and 3; (+)-HRESIMS *m/z* 519.3581 [M + H]^+^ (calcd. for C_33_H_47_N_2_O_3_, 519.3587).

Myribuxine L (**12**): pale-yellow, amorphous powder; ${[\alpha ]}_{\mathrm{D}}^{25}$ +38.06 (*c* 0.1, MeOH); UV (MeOH) *λ*_max_ (log *ε*) 203 (3.73), 273 (3.68) nm; ^1^H and ^13^C NMR data see Tables 1 and 3; (+)-HRESIMS *m/z* 531.3928 [M + H]^+^ (calcd. for C_35_H_51_N_2_O_2_, 531.3951).

### Computational NMR

Conformational searches were performed via molecular dynamics simulations using the CREST (version 2.13) program [20] at 400 K for 100 ps, with energies and gradients calculated at the GFN0-xTB level in the gas phase [21]. The conformers accounting for over 97% of the total Boltzmann distribution were selected and further subjected to geometric optimization at the GFN2-xTB level in the gas phase. The resulting low-energy conformers were subsequently optimized, and their vibrational frequencies were calculated at the B3LYP/6-31G(d) level in the gas phase using the Gaussian 09 software package (Gaussian, Inc., Wallingford, CT, USA) to confirm that all optimized structures were true local energy minima without imaginary frequencies and obtain relative Gibbs free energies. The resulting Gibbs free energies were utilized to calculate the Boltzmann population distributions for the subsequent steps. NMR shielding constants for these conformers were computed using the gauge-including atomic orbital method at the mPW1PW91/6-311+G(d,p) level of density functional theory, applying the polarizable continuum model in MeOH. The calculated NMR chemical shifts were Boltzmann-averaged according to their relative Gibbs free energies. The chemical shifts of tetramethylsilane, calculated at the identical theoretical level, were employed as the reference. The stereochemical configurations were ultimately determined using the DP4^+^ probability method [22].

### Cell culture

HepG2, A549, U251, and HCT-116 human cancer cell lines were cultured in their respective complete media supplemented with 10% fetal bovine serum and 1% penicillin–streptomycin, at 37 °C in a humidified 5% CO_2_ atmosphere. HepG2, U251, and HCT-116 cells were grown in high-glucose DMEM, whereas A549 cells were grown in RPMI-1640 medium (all media from Meilunbio, Dalian, China). Upon reaching 80–90% confluence, cells were passaged using 0.25% trypsin–EDTA (Meilunbio). In all experiments, the final concentration of dimethyl sulfoxide (DMSO) in the culture medium was strictly maintained below 0.1% (v/v) to avoid solvent-induced cytotoxicity.

### Cytotoxicity assay

Cytotoxicity was evaluated using the MTT assay. Cells in the logarithmic growth phase were seeded into 96-well plates (1 × 10^4^ cells/well). After overnight attachment, the medium was replaced with fresh medium containing the test compound at 3.125, 6.25, 12.5, 25, and 50 μM. Cisplatin, sorafenib, temozolomide, and oxaliplatin were employed as positive controls. The assay was performed in three independent experiments, with each concentration tested in triplicate technical wells. After 48 h of incubation, 50 μL of MTT solution (2 mg/mL) was added, and the cells were incubated for an additional 4 h. The supernatant was removed, formazan crystals were dissolved in 100 μL DMSO, and absorbance was measured at 490 nm using a microplate reader. Cell viability (%) was calculated using the following formula: Viability (%) = (*A*_treated _− *A*_blank_)/(*A*_control _− *A*_blank_) × 100.

### Flow cytometric analysis of apoptosis

Apoptosis was evaluated using an Annexin V-FITC/7-AAD double-staining kit (Tonbo Biosciences, San Diego, CA, USA) according to the manufacturer’s instructions. Cells were seeded in 6-well plates (2.5 × 10^5^ cells/well), incubated overnight, and then exposed to **2**. For the dose–response assay, cells were treated with **2** at 0 (vehicle control), 10, 20, and 40 μM for 48 h. For the time-course assay, cells were treated with 20 μM of **2** for 0 (untreated control), 12, 24, and 48 h. Each condition was tested in triplicate. Following treatment, cells were harvested, stained with Annexin V-FITC/7-AAD, and immediately analyzed on a BD FACSVerse flow cytometer.

### Network pharmacology

SwissTargetPrediction (http://www.swisstargetprediction.ch/) and SuperPred (https://prediction.charite.de/index.php) were used to predict the potential targets. Relevant liver cancer targets were obtained from GeneCards (https://www.genecards.org/), OMIM (https://omim.org/), and DisGeNET (https://disgenet.com/). The potential targets of **2** in liver cancer were obtained through Venny 2.1 (https://bioinfogp.cnb.csic.es/tools/venny/index.html). Potential therapeutic targets were entered into the STRING (https://string-db.org/) database, and the results were imported into Cytoscape 3.6.0 software to analyze the protein–protein interaction (PPI) network to obtain the core targets. The bioinformatics annotation database DAVID (https://david.ncifcrf.gov/tools.jsp) was used for GO and KEGG pathway enrichment analyses and visualization. Furthermore, the detailed screening thresholds are as follows: SwissTargetPrediction probability ≥ 0.1, GeneCards relevance score ≥ 10.0, and STRING interaction confidence score ≥ 0.70.

### Molecular docking

All molecular docking analyses were implemented in Schrödinger Maestro with the Glide module. The crystal structure of HSP90α (PDB: 6GQ6) was prepared via Protein Preparation Wizard. The 2D structure of **2** was imported into LigPrep to generate physiologically relevant protonated conformations. Molecular docking was performed using the extra-precision (XP) module with a 20 Å grid box centered at the coordinates X = − 20.0, Y = 0.0, and Z = 0.0, and validated by an RMSD of 0.504 Å, confirming the reliability of the docking parameters. The most favorable binding pose was retained, and PyMOL was used to visualize hydrogen bonds and hydrophobic contacts between **2** and key residues, illustrating plausible binding interactions between the compound and HSP90α.

### Statistical analysis

All experimental data are presented as the mean ± SD or mean ± SEM based on three independent biological experiments. Statistical analysis and non-linear regression curve fitting (for IC_50_ determination) were performed using GraphPad Prism 9 software (GraphPad Software, San Diego, CA, USA).

## Supplementary Information

Additional file 1. The online version contains supplementary material available at Supplementary Material. Figures related to the article; HRESIMS, UV, 1D NMR, and 2D NMR spectra of compounds **1**−**12**; calculated NMR data for compounds **2**, **8** and **9** (PDF).

## Data Availability

The data generated and/or analyzed during the current study are available from the corresponding author onreasonable request.
