# Supplementary material for: Structurally diverse triterpenoid alkaloids from Buxus myrica with cytotoxic and apoptosis-inducing activities
Source: Nat Prod Bioprospect. 2026 Nov 3;16(1):100. doi: 10.1007/s13659-026-00656-6 (PMC13728022; doi:10.1007/s13659-026-00656-6)
Supplement: Supplementary file 1 [file 13659_2026_656_MOESM1_ESM.pdf]

Structurally Diverse Triterpenoid Alkaloids from *Buxus* myrica with Cytotoxic and Apoptosis-Inducing Activities

Myribuxine A (1) **1**

Figure S1.1 UV spectrum of compound **1** 1

Figure S1.2 HR-ESI-MS spectrum of compound **1** 1

Figure S1.3 ^{1}H NMR spectrum of compound **1** (600 MHz, MeOD) 1

Figure S1.4 ^{13}C NMR spectrum of compound **1** (150 MHz, MeOD) 2

Figure S1.5 HSQC spectrum of compound **1** 2

Figure S1.6 HMBC spectrum of compound **1** 3

Figure S1.7 ROESY spectrum of compound **1** 3

Myribuxine B (2) **4**

Figure S2.1 UV spectrum of compound **2** 4

Figure S2.2 HR-ESI-MS spectrum of compound **2** 4

Figure S2.3 ^{1}H NMR spectrum of compound **2** (600 MHz, MeOD) 4

Figure S2.4 ^{13}C NMR spectrum of compound **2** (150 MHz, MeOD) 5

Figure S2.5 HSQC spectrum of compound **2** 5

Figure S2.6 HMBC spectrum of compound **2** 6

Figure S2.7 NOESY spectrum of compound **2** 6

Table S1 Experimental/calculated NMR shifts and statistical parameters for **2** 6

Table S2 Relative energies and Boltzmann populations for **2a** 8

Cartesian coordinates of **2a-1** 9

Cartesian coordinates of **2a-2** 11

Cartesian coordinates of **2a-3** 13

Cartesian coordinates of **2a-4** 15

Cartesian coordinates of **2a-5** 17

Cartesian coordinates of **2a-6** 19

Table S3 Relative energies and Boltzmann populations for **2b** 21

Cartesian coordinates of **2b-1** 22

Cartesian coordinates of **2b-2** 24

Cartesian coordinates of **2b-3** 26

Cartesian coordinates of **2b-4** 28

Cartesian coordinates of **2b-5** 30

Cartesian coordinates of **2b-6** 32

Myribuxine C (3) **35**

Figure S3.1 UV spectrum of compound **3** 35

Figure S3.2 HR-ESI-MS spectrum of compound **3** 35

Figure S3.3 ^{1}H NMR spectrum of compound **3** (400 MHz, MeOD) 35

Figure S3.4 ^{13}C NMR spectrum of compound **3** (100 MHz, MeOD) 36

Figure S3.5 HSQC spectrum of compound **3** 36
Figure S3.6 HMBC spectrum of compound **3** 37
Figure S3.7 NOESY spectrum of compound **3** 37
Myribuxine D (4) **38**
Figure S4.1 UV spectrum of compound **4** 38
Figure S4.2 HR-ESI-MS spectrum of compound **4** 38
Figure S4.3 ^{1}H NMR spectrum of compound **4** (600 MHz, DMSO-d_6) 38
Figure S4.4 ^{13}C NMR spectrum of compound **4** (150 MHz, DMSO-d_6) 39
Figure S4.5 HSQC spectrum of compound **4** 39
Figure S4.6 HMBC spectrum of compound **4** 40
Figure S4.7 NOESY spectrum of compound **4** 40
Myribuxine E (5) **41**
Figure S5.1 UV spectrum of compound **5** 41
Figure S5.2 HR-ESI-MS spectrum of compound **5** 41
Figure S5.3 ^{1}H NMR spectrum of compound **5** (400 MHz, MeOD) 41
Figure S5.4 ^{13}C NMR spectrum of compound **5** (100 MHz, MeOD) 42
Figure S5.5 HSQC spectrum of compound **5** 42
Figure S5.6 HMBC spectrum of compound **5** 43
Figure S5.7 NOESY spectrum of compound **5** 43
Myribuxine F (6) **43**
Figure S6.1 UV spectrum of compound **6** 44
Figure S6.2 HR-ESI-MS spectrum of compound **6** 44
Figure S6.3 ^{1}H NMR spectrum of compound **6** (400 MHz, MeOD) 44
Figure S6.4 ^{13}C NMR spectrum of compound **6** (100 MHz, MeOD) 45
Figure S6.5 HSQC spectrum of compound **6** 45
Figure S6.6 HMBC spectrum of compound **6** 46
Figure S6.7 NOESY spectrum of compound **6** 46
Myribuxine G (7) **46**
Figure S7.1 UV spectrum of compound **7** 47
Figure S7.2 HR-ESI-MS spectrum of compound **7** 47
Figure S7.3 ^{1}H NMR spectrum of compound **7** (600 MHz, MeOD) 47
Figure S7.4 ^{13}C NMR spectrum of compound **7** (150 MHz, MeOD) 48
Figure S7.5 HSQC spectrum of compound **7** 48
Figure S7.6 HMBC spectrum of compound **7** 49
Figure S7.7 NOESY spectrum of compound **7** 49
Myribuxine H (8) **50**
Figure S8.1 UV spectrum of compound **8** 50
Figure S8.2 HR-ESI-MS spectrum of compound **8** 50
Figure S8.3 ^{1}H NMR spectrum of compound **8** (400 MHz, MeOD) 50

Figure S8.4 ^{13}C NMR spectrum of compound **8** (100 MHz, MeOD) 51
Figure S8.5 HSQC spectrum of compound **8** 51
Figure S8.6 HMBC spectrum of compound **8** 52
Figure S8.7 NOESY spectrum of compound **8** 52
Table S4 Experimental/calculated NMR shifts and statistical parameters for **8** 52
Table S5 Relative energies and Boltzmann populations for **8a** 54
Cartesian coordinates of **8a-1** 55
Cartesian coordinates of **8a-2** 57
Table S6 Relative energies and Boltzmann populations for **8b** 59
Cartesian coordinates of **8b-1** 60
Cartesian coordinates of **8b-2** 62
Cartesian coordinates of **8b-3** 64
Cartesian coordinates of **8b-4** 67
Cartesian coordinates of **8b-5** 69
Myribuxine I (9) 72
Figure S9.1 UV spectrum of compound **9** 72
Figure S9.2 HR-ESI-MS spectrum of compound **9** 72
Figure S9.3 ^{1}H NMR spectrum of compound **9** (600 MHz, MeOD) 72
Figure S9.4 ^{13}C NMR spectrum of compound **9** (150 MHz, MeOD) 73
Figure S9.5 HSQC spectrum of compound **9** 73
Figure S9.6 HMBC spectrum of compound **9** 74
Figure S9.7 NOESY spectrum of compound **9** 74
Table S7 Experimental/calculated NMR shifts and statistical parameters for **9** 74
Table S8 Relative energies and Boltzmann populations for **9a** 76
Cartesian coordinates of **9a-1** 77
Cartesian coordinates of **9a-2** 79
Cartesian coordinates of **9a-3** 81
Table S9 Relative energies and Boltzmann populations for **9b** 83
Cartesian coordinates of **9b-1** 84
Cartesian coordinates of **9b-2** 86
Cartesian coordinates of **9b-3** 88
Cartesian coordinates of **9b-4** 91
Myribuxine J (10) 93
Figure S10.1 UV spectrum of compound **10** 93
Figure S10.2 HR-ESI-MS spectrum of compound **10** 93
Figure S10.3 ^{1}H NMR spectrum of compound **10** (600 MHz, MeOD) 94
Figure S10.4 ^{13}C NMR spectrum of compound **10** (150 MHz, MeOD) 94
Figure S10.5 HSQC spectrum of compound **10** 95
Figure S10.6 HMBC spectrum of compound **10** 95
Myribuxine K (11) 95

Figure S11.1 UV spectrum of compound **11** 96
Figure S11.2 HR-ESI-MS spectrum of compound **11** 96
Figure S11.3 ^{1}H NMR spectrum of compound **11** (600 MHz, MeOD) 96
Figure S11.4 ^{13}C NMR spectrum of compound **11** (150 MHz, MeOD) 97
Figure S11.5 HSQC spectrum of compound **11** 97
Figure S11.6 HMBC spectrum of compound **11** 98
Figure S11.7 NOESY spectrum of compound **11** 98
Myribuxine L (12) 98
Figure S12.1 UV spectrum of compound **12** 99
Figure S12.2 HR-ESI-MS spectrum of compound **12** 99
Figure S12.3 ^{1}H NMR spectrum of compound **12** (400 MHz, MeOD) 99
Figure S12.4 ^{13}C NMR spectrum of compound **12** (100 MHz, MeOD) 100
Figure S12.5 HSQC spectrum of compound **12** 100
Figure S12.6 HMBC spectrum of compound **12** 101
Figure S12.7 NOESY spectrum of compound **12** 101

Myribuxine A (**1**)

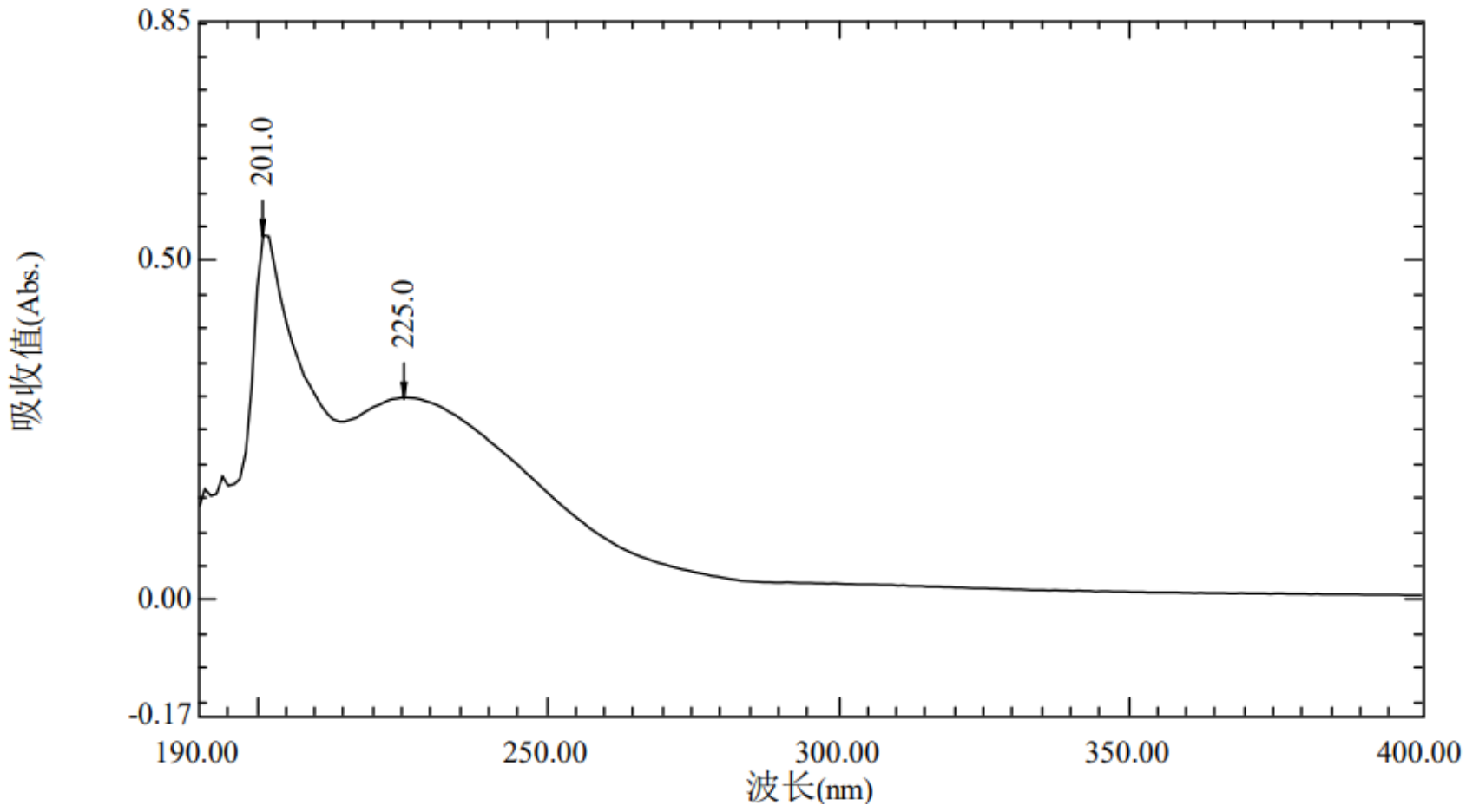

Figure S1.1 UV spectrum of compound **1**

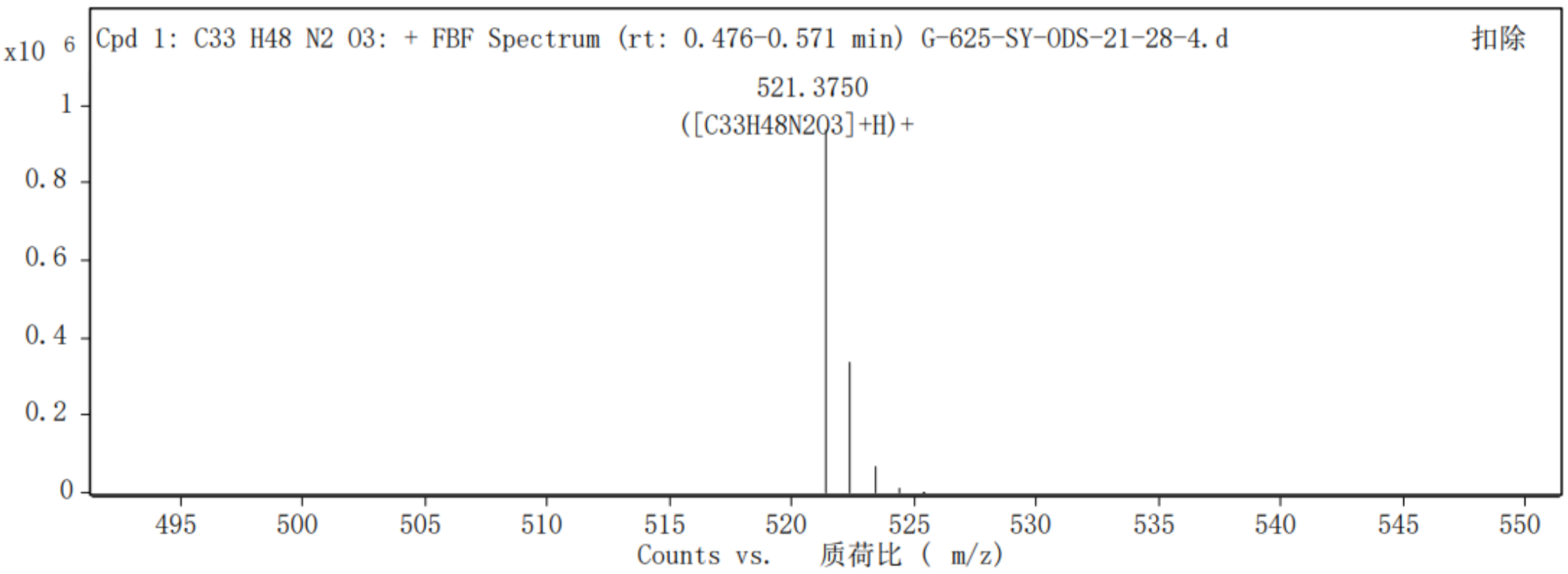

Figure S1.2 HR-ESI-MS spectrum of compound **1**

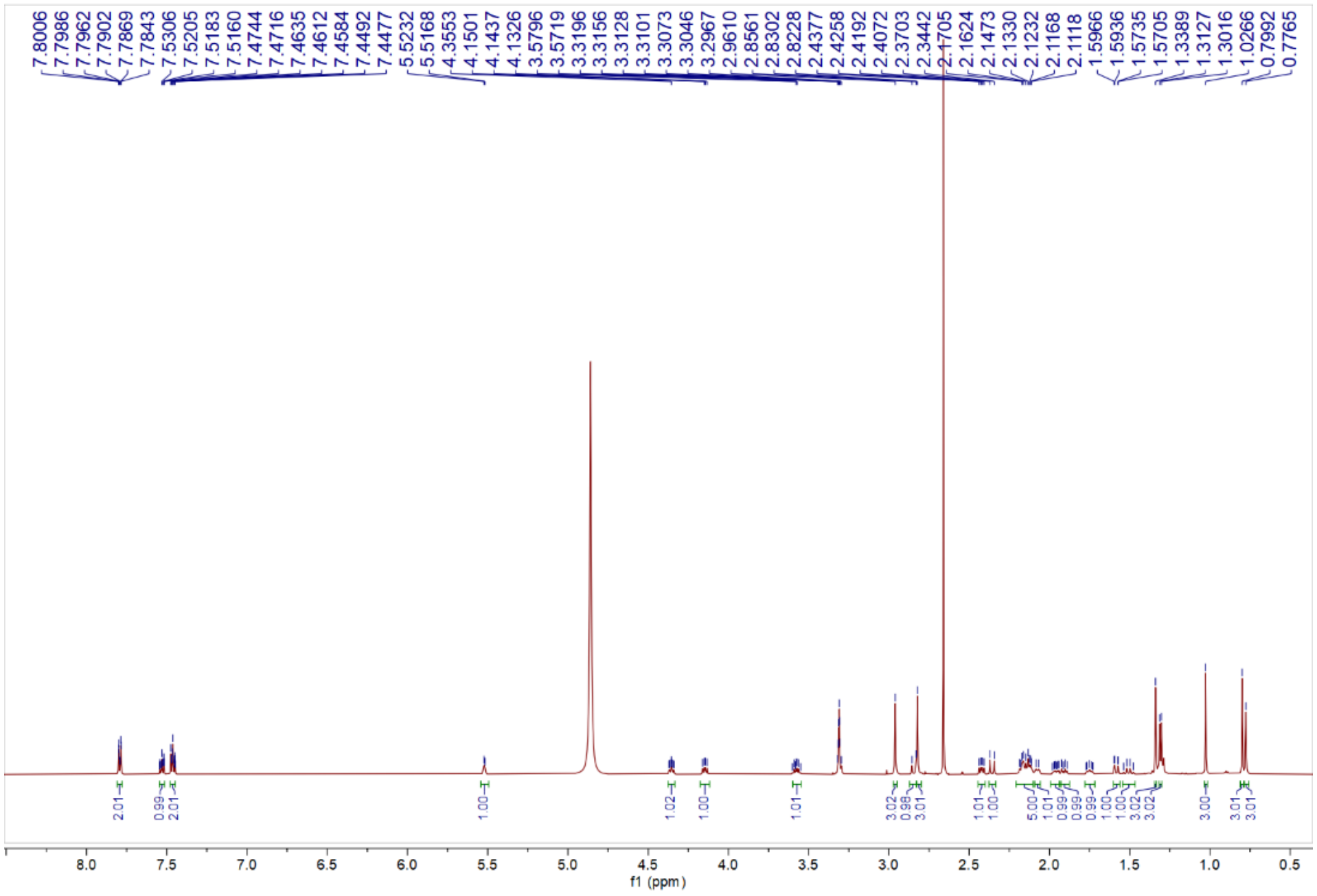

Figure S1.3 ^{1}H NMR spectrum of compound **1** (600 MHz, MeOD)

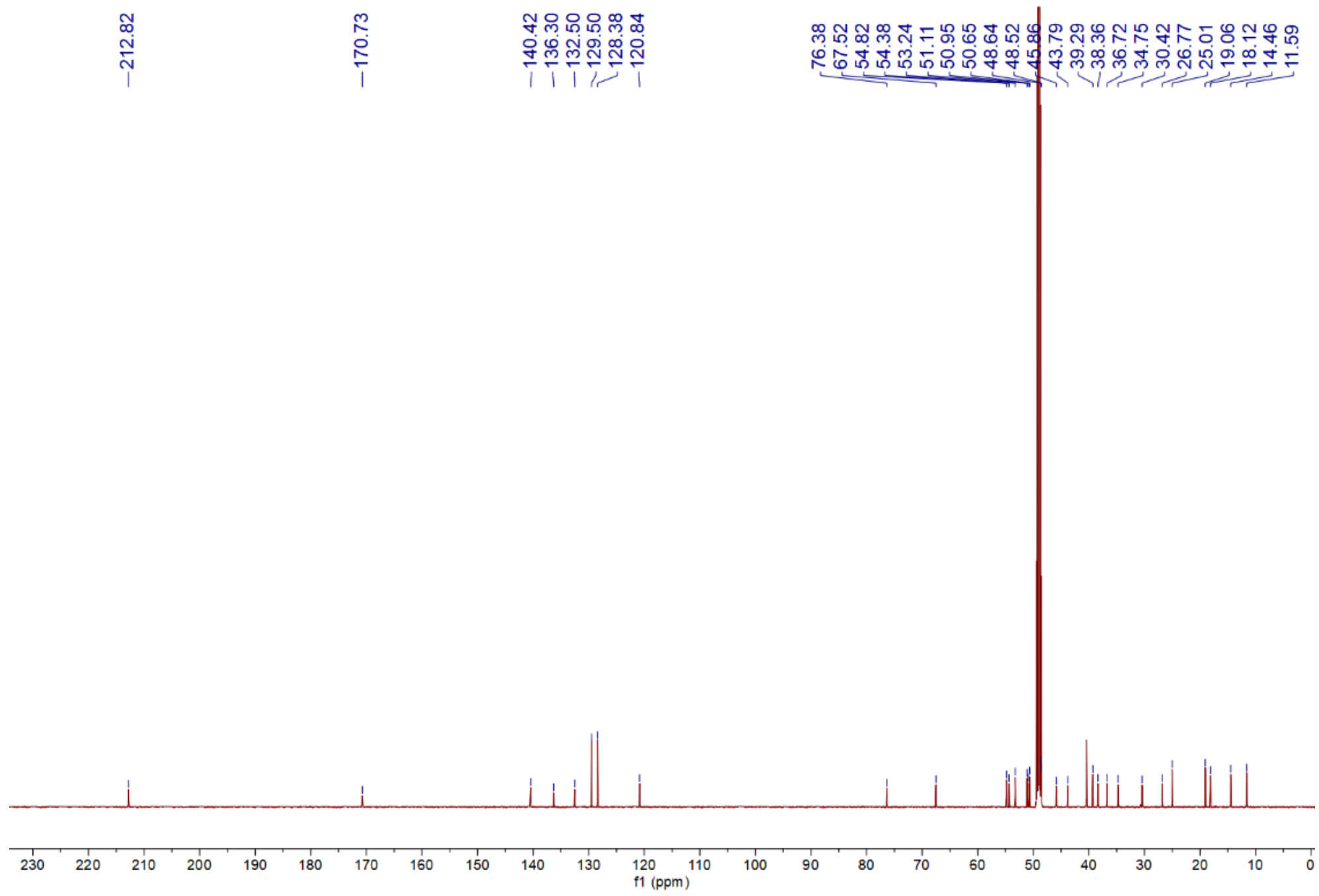

Figure S1.4 ^{13}C NMR spectrum of compound **1** (150 MHz, MeOD)

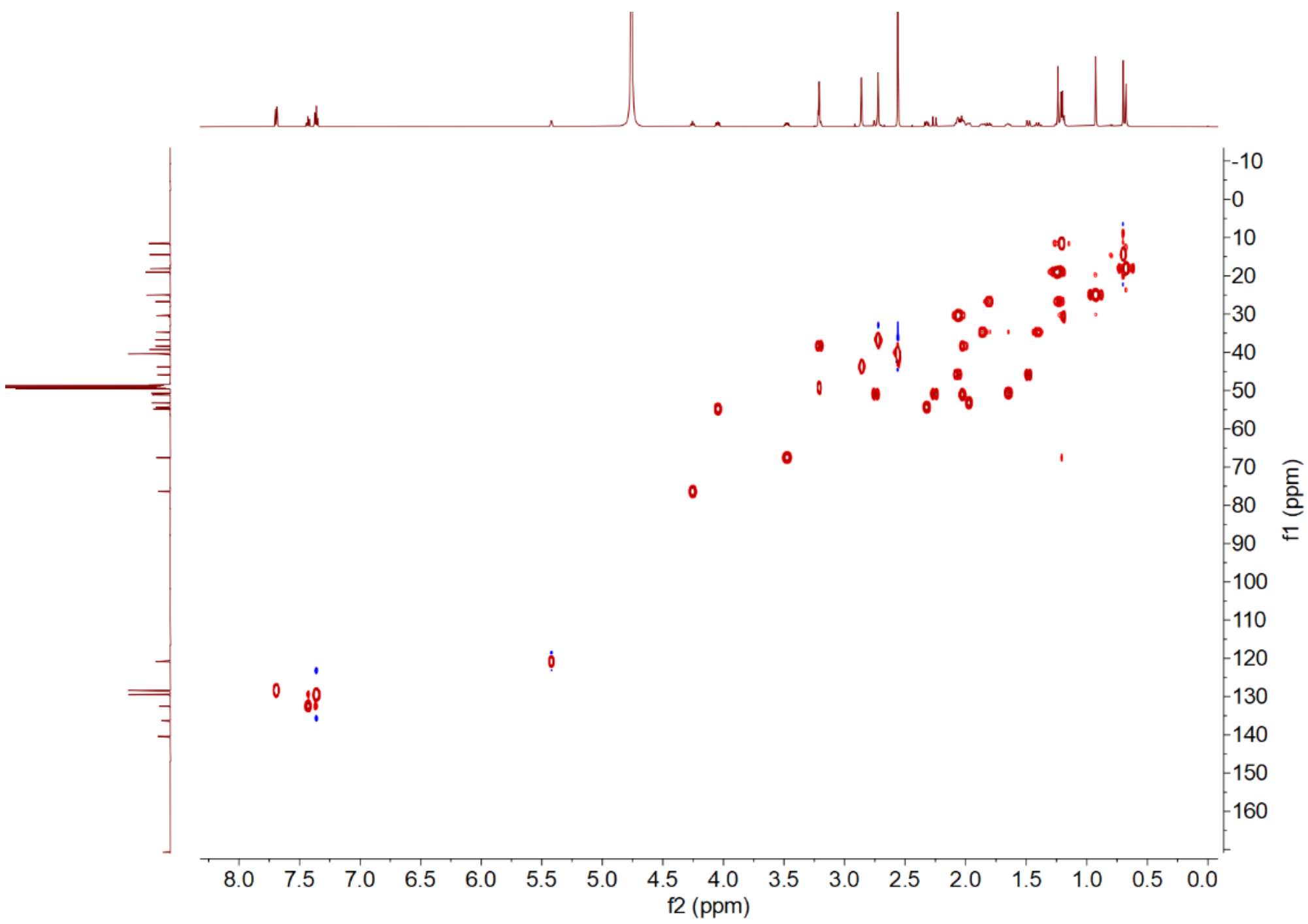

Figure S1.5 HSQC spectrum of compound **1**

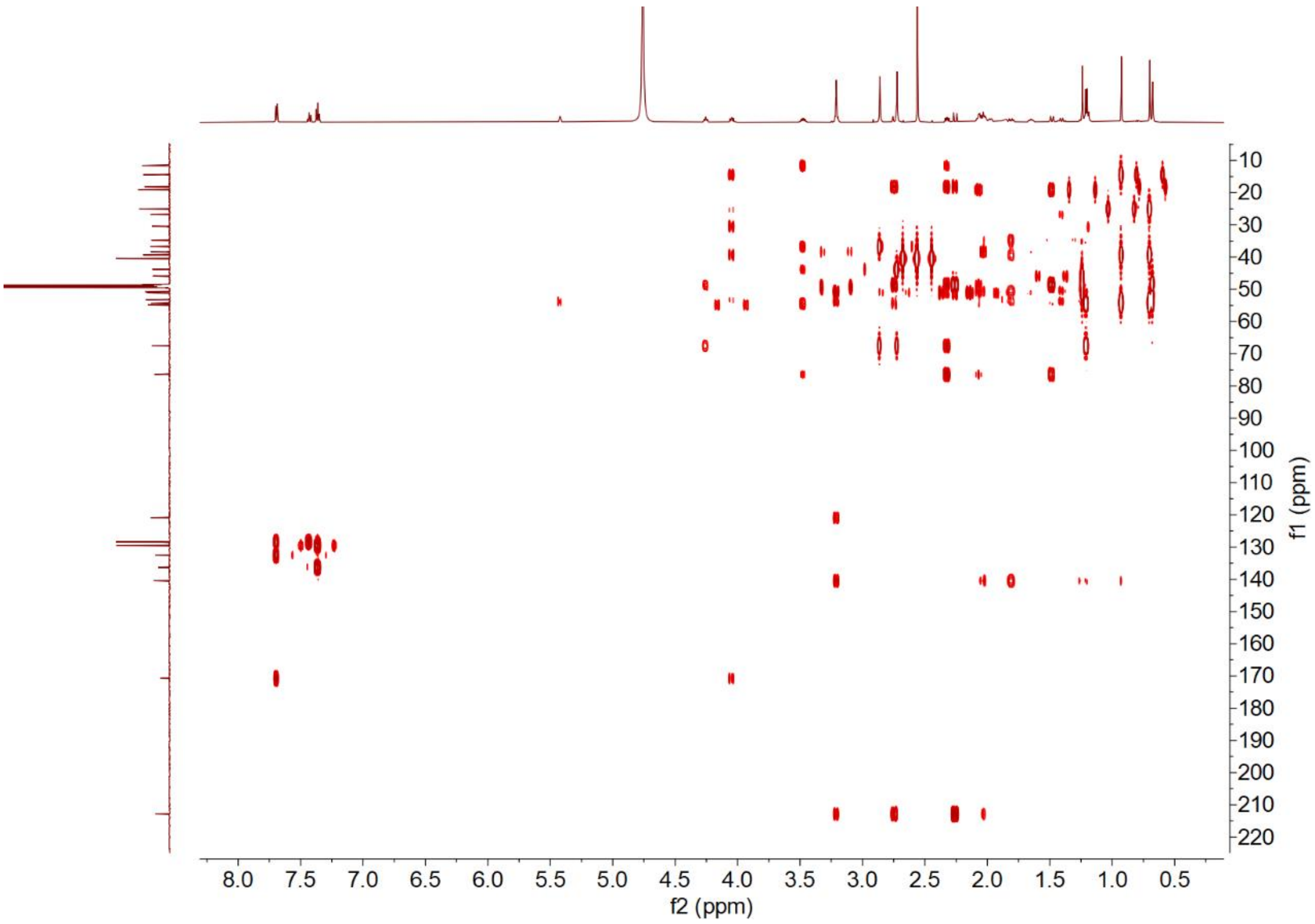

Figure S1.6 HMBC spectrum of compound **1**

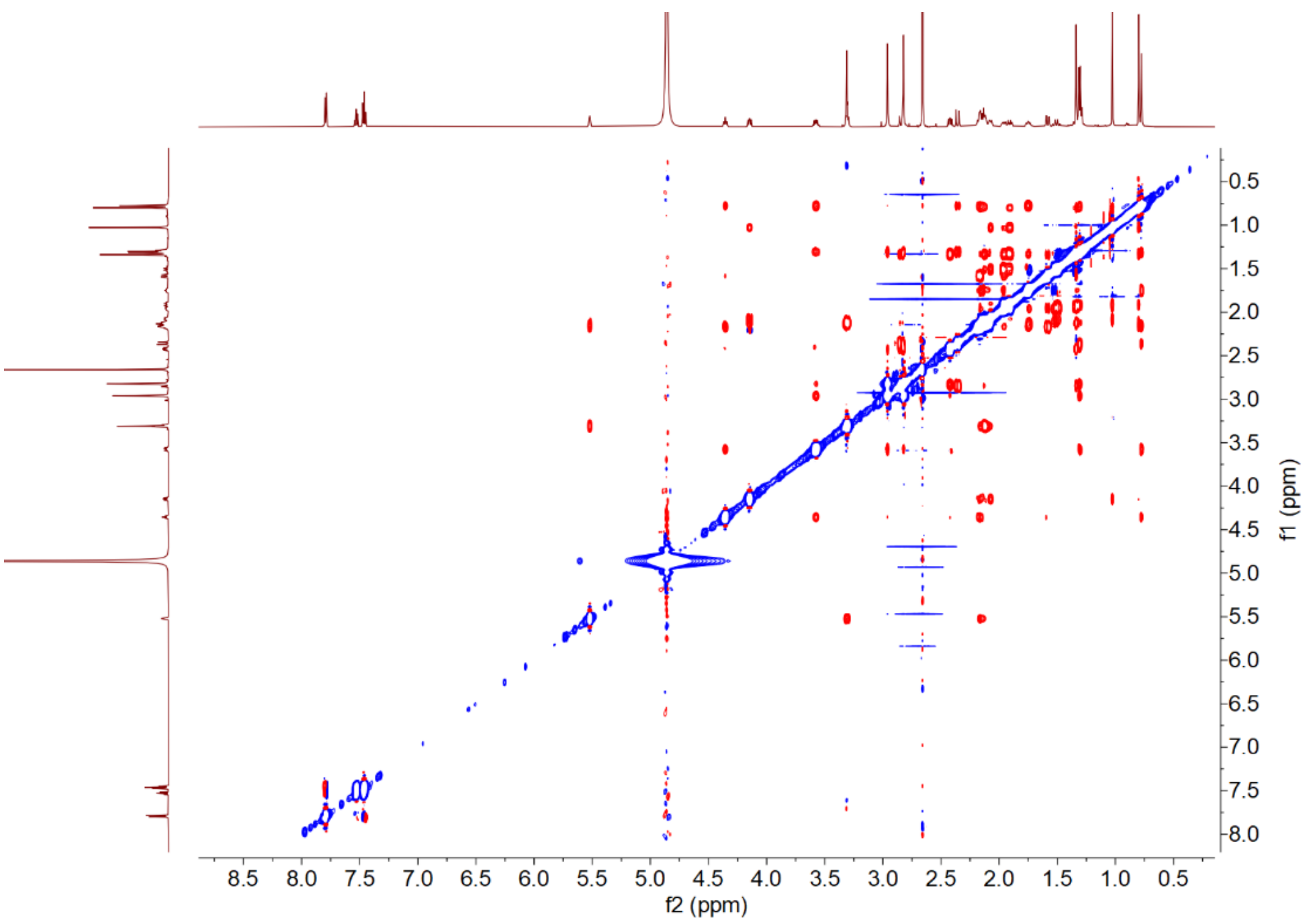

Figure S1.7 ROESY spectrum of compound **1**

Myribuxine B (**2**)

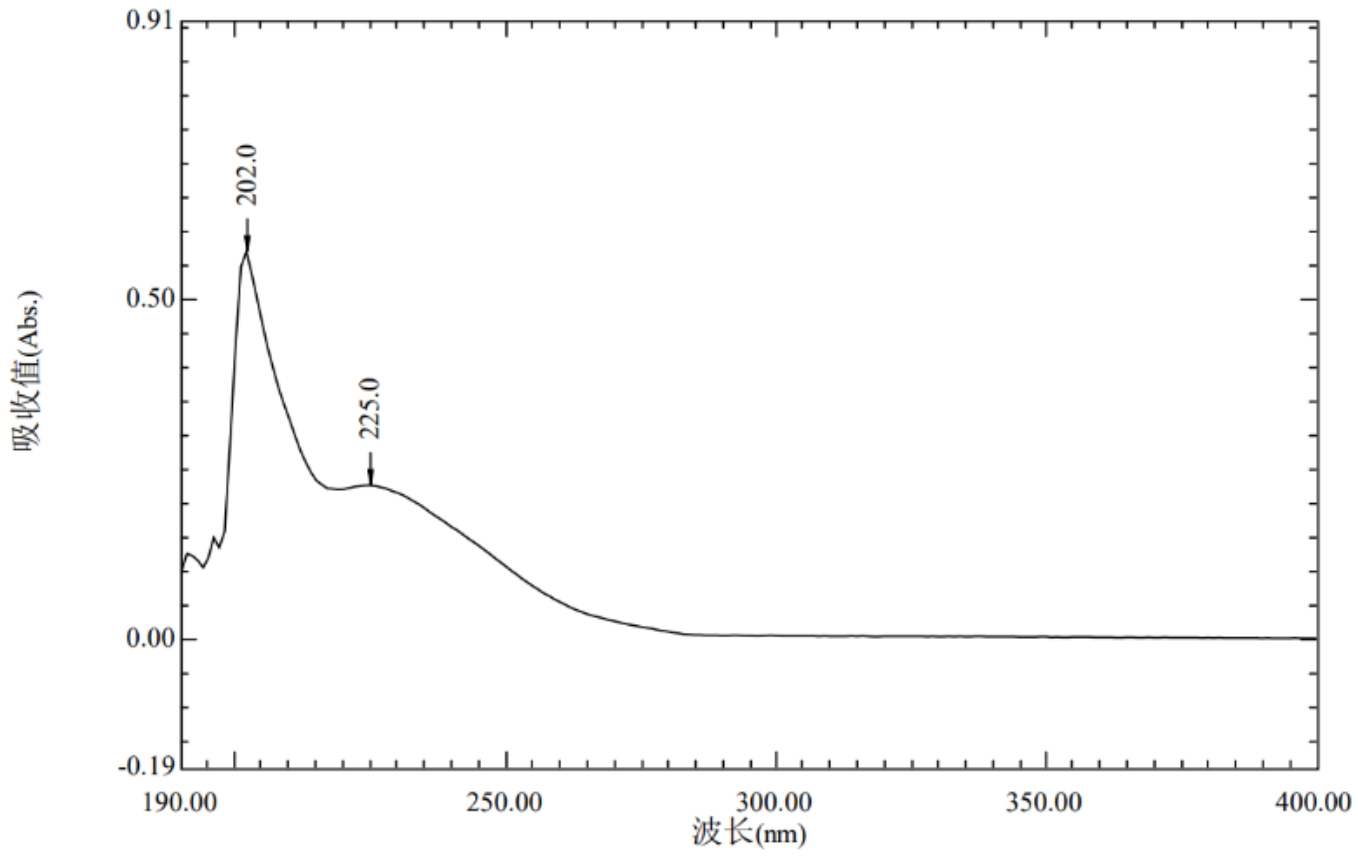

Figure S2.1 UV spectrum of compound **2**

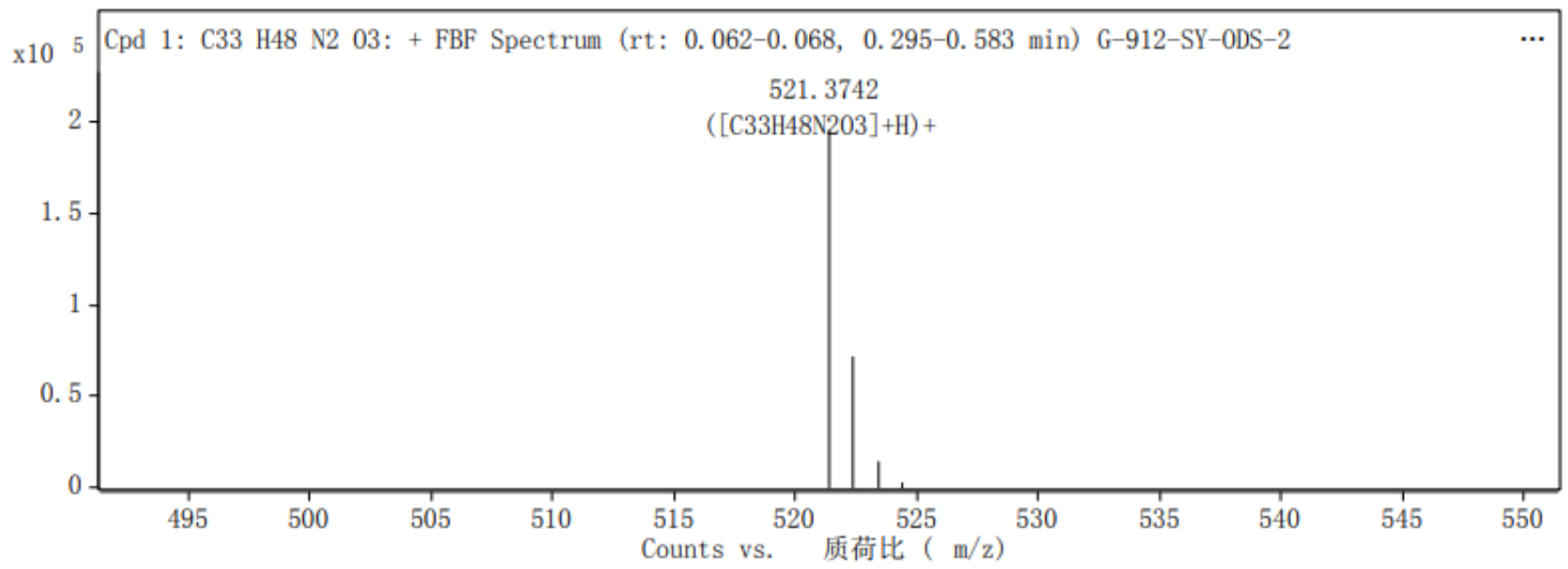

Figure S2.2 HR-ESI-MS spectrum of compound **2**

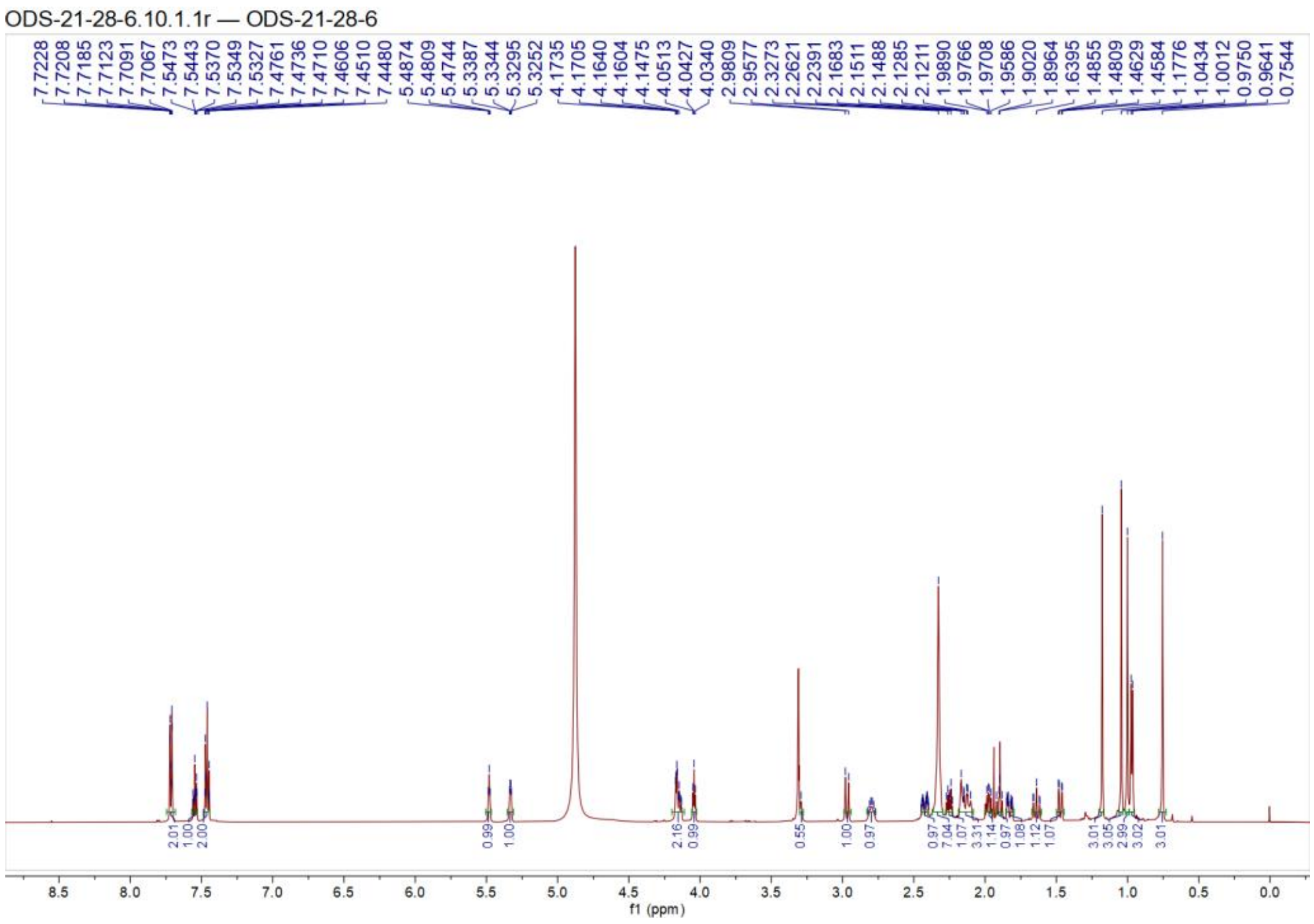

Figure S2.3 ^{1}H NMR spectrum of compound **2** (600 MHz, MeOD)

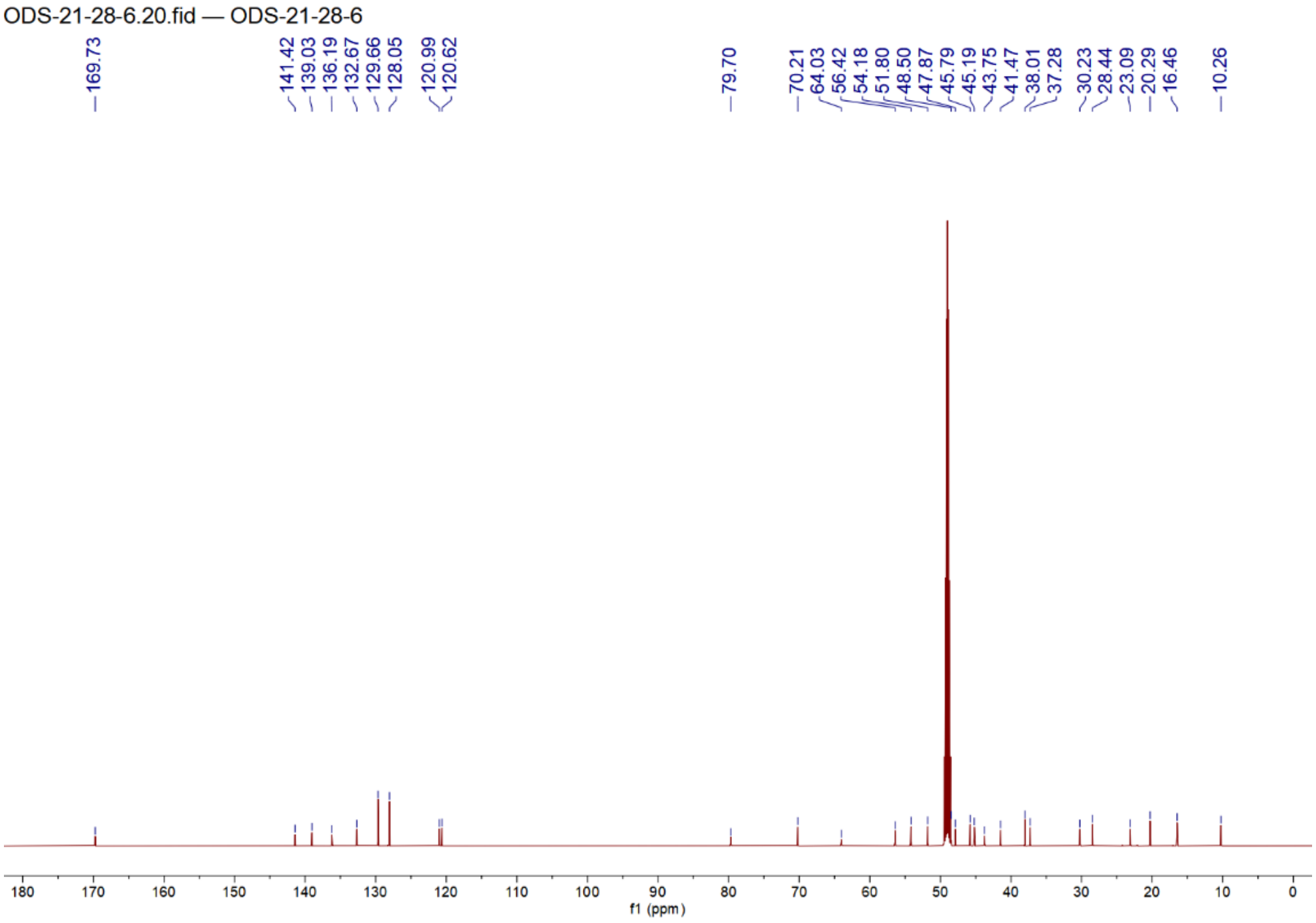

Figure S2.4 ^{13}C NMR spectrum of compound **2** (150 MHz, MeOD)

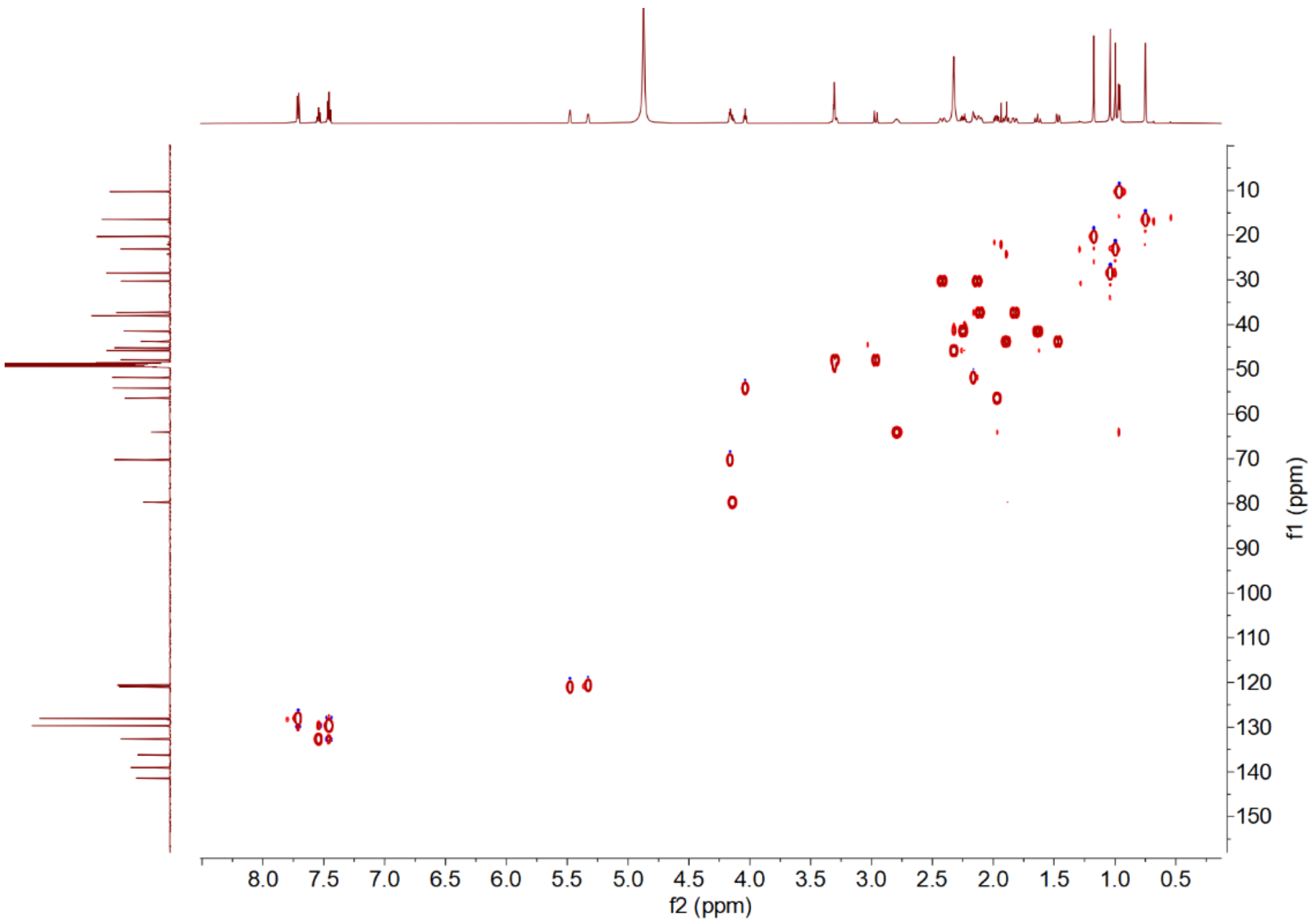

Figure S2.5 HSQC spectrum of compound **2**

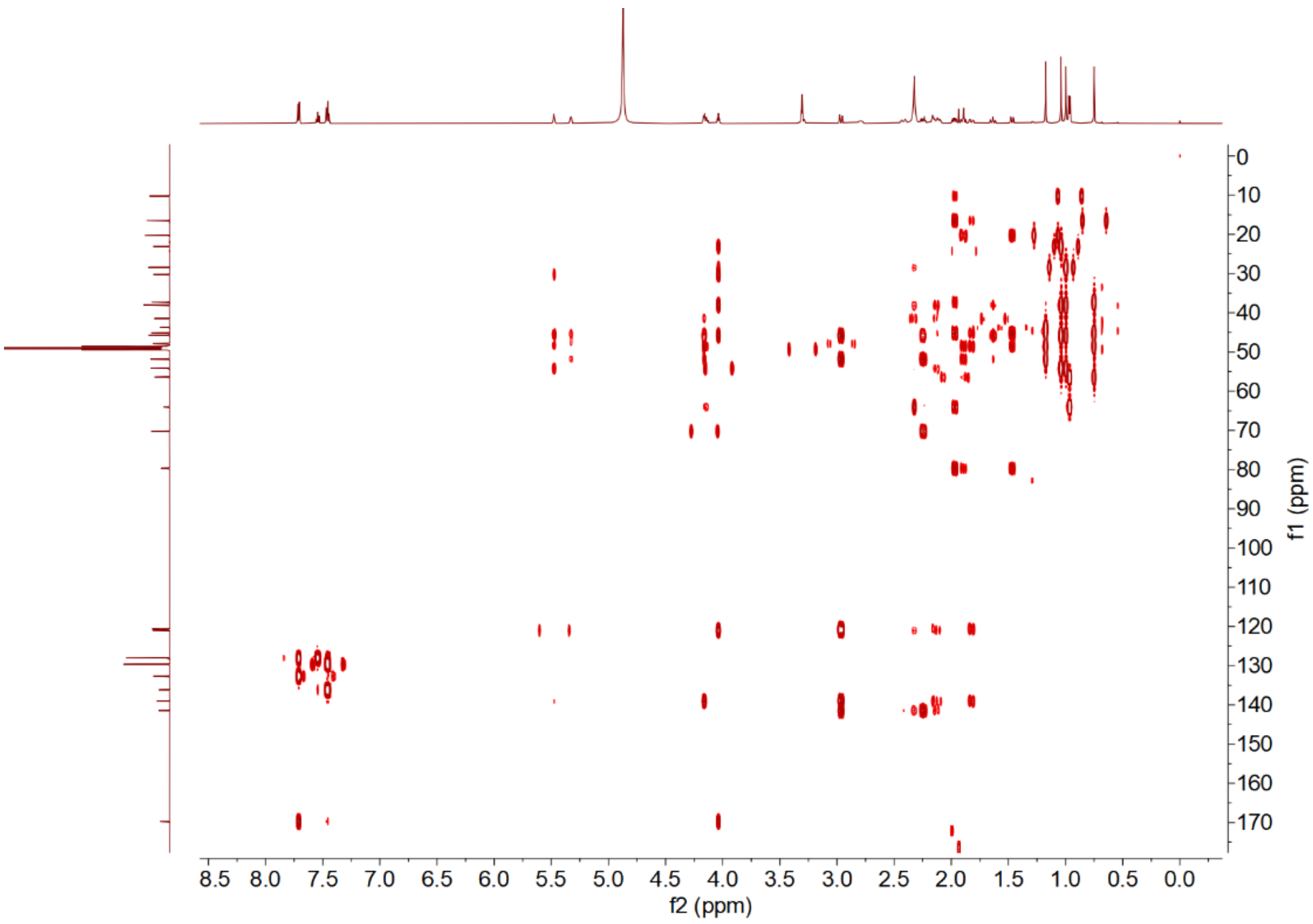

Figure S2.6 HMBC spectrum of compound **2**

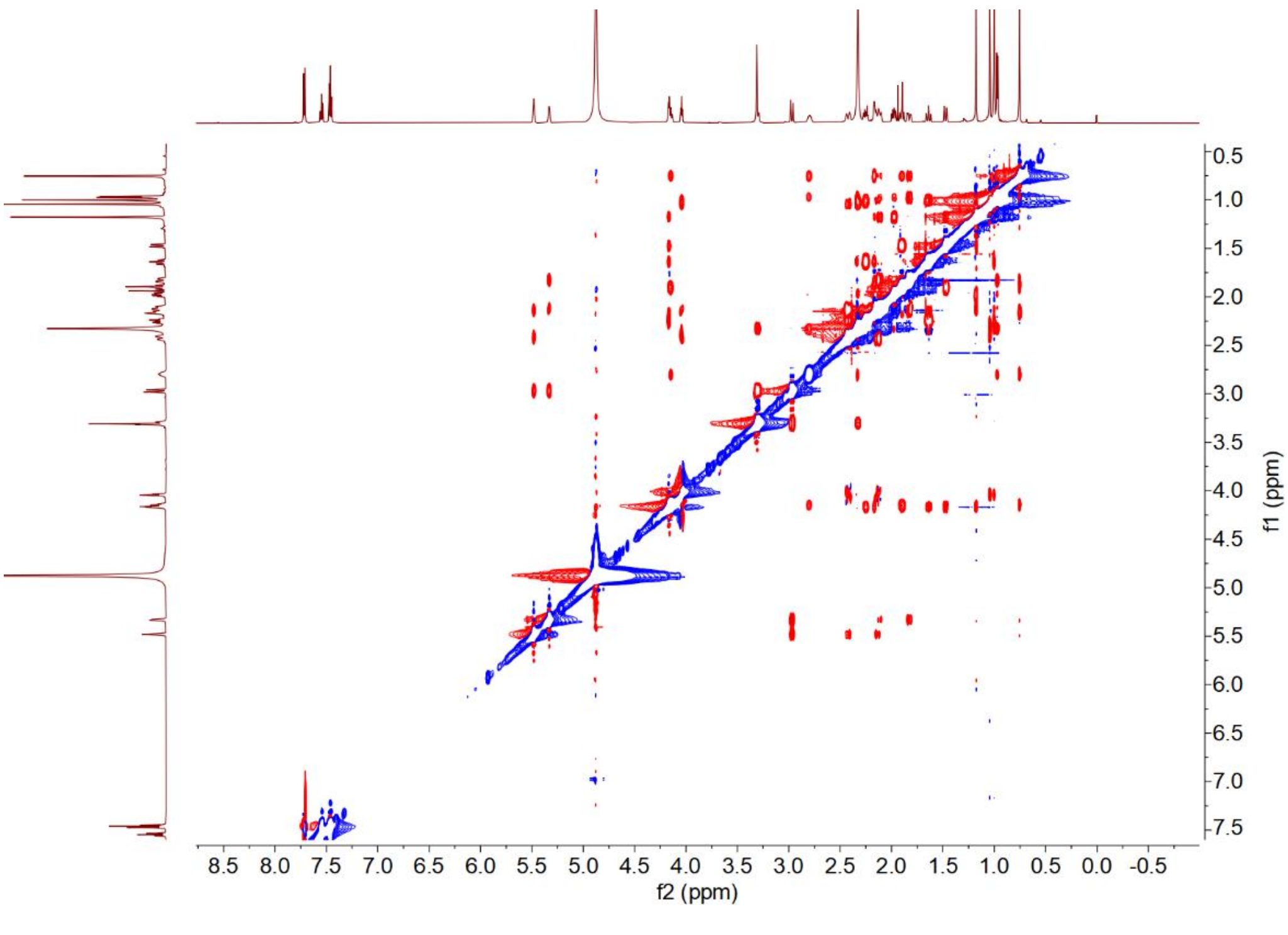

Figure S2.7 NOESY spectrum of compound **2**

Table S1 Experimental/calculated NMR shifts and statistical parameters for **2**

Position / Parameter	Experimental	Isomer 2a	Isomer 2b
C	121	125.5140019	130.2716225
C	30.2	34.71749533	34.13962412

C	54.2	58.94499019	57.47327815
C	38	43.90436858	44.4452989
C	45.8	47.97135794	49.1553774
C	41.8	40.62680323	41.48664734
C	70.2	74.79657067	75.39892512
C	51.8	53.04278936	61.21163978
C	139	149.8553428	148.8850841
C	141.4	156.0	154.0
C	120.6	132.2	134.4
C	37.3	40.91	41.24
C	45.2	50.92	52.35
C	48.5	53.43	53.25
C	43.8	46.31	49.96
C	79.7	83.59	83.30
C	56.4	61.29	60.39
C	16.5	18.11	17.39
C	47.9	51.17	49.55
C	64	66.78	67.39
C	10.3	12.15	12.10
C	20.3	22.90	19.67
C	23.1	23.32	24.18
C	28.4	27.07	27.59
C	136.2	142.44	142.04
C	128.1	135.3692828	134.9796667
C	128.1	134.1696622	133.6417614
C	129.7	135.8068752	136.0875715
C	129.7	135.6851899	135.8138854
C	132.7	139.6523618	139.4011357
C	169.7	173.5181495	173.8062713
C	45.8	45.25006354	43.36217493
C	45.8	38.39388111	40.08755413
**^{13}C MAE (unscaled)**		**4.713**	**5.061**
**^{13}C CMAE**		**2.144**	**2.333**
**^{13}C R^2**		**0.996**	**0.9956**
**^{13}C sDP4$^+$ Probability**		**99.70%**	**0.30%**
**^{13}C uDP4$^+$ Probability**		**98.55%**	**1.45%**
**^{13}C DP4$^+$ Probability**		**100%**	**0%**
H	5.48	5.838110403	6.132036319
H	2.42	2.989297926	3.060968655
H	2.13	2.143662481	2.302246824
H	4.04	4.274691745	4.366237601
H	2.33	2.462219014	2.634964837
H	1.64	1.526565696	2.137260885

H	2.25	2.614362511	2.3552579
H	4.17	4.584023297	3.611212005
H	2.17	2.577312072	2.031303298
H	5.33	5.989985878	6.029763905
H	2.13	2.316531132	2.428834318
H	1.83	2.115660595	2.172455349
H	1.91	2.086986496	2.330023548
H	1.47	1.468689316	1.693847626
H	4.14	4.357081574	4.449242049
H	1.97	1.832742085	1.876380734
H	0.75	0.859443806	0.725875441
H	3.29	4.272816432	3.10889675
H	2.97	2.986007466	3.076252163
H	2.8	2.733245551	2.704926656
H	0.97	0.933616345	0.959889884
H	1.18	1.402477685	1.23737644
H	1	1.062625382	1.126409439
H	1.04	1.14217318	1.302730102
H	7.72	8.339183692	8.419232897
H	7.72	8.19334711	8.209952879
H	7.46	7.969911886	8.022896571
H	7.46	7.933052499	7.963570536
H	7.55	8.040450735	8.035117426
H	2.38	2.469879826	2.369062901
H	2.38	2.195325699	2.293065498
**^{1}H MAE (unscaled)**		**0.281**	**0.306**
**^{1}H sDP4$^+$ Probability**		**99.90%**	**0.10%**
**^{1}H uDP4$^+$ Probability**		**99.93%**	**0.07%**
**^{1}H DP4$^+$ Probability**		**100%**	**0%**
**sDP4$^+$ Probability (All Data)**		**100%**	**0%**
**uDP4$^+$ Probability (All Data)**		**100%**	**0%**
**DP4$^+$ Probability (All Data)**		**100%**	**0%**

Table S2 Relative energies and Boltzmann populations for **2a**

Conformer	Energy (Hartree)	Δ*G* (kcal/mol)	Population (%)
2a-1	-1620.846023	0.466	12.43
2a-2	-1620.846711	0.035	25.77
2a-3	-1620.846766	0.000	27.31

Conformer	Energy (Hartree)	ΔG (kcal/mol)	Population (%)
2a-4	-1620.846021	0.467	12.41
2a-5	-1620.845911	0.537	11.04
2a-6	-1620.845911	0.537	11.04

Cartesian coordinates of **2a-1**

Center Number	Atomic Number	Atomic Type	Coordinates (Angstroms) X	Y	Z
1	6	0	4.558771	-1.417208	1.535514
2	6	0	4.487464	-0.874579	0.101600
3	6	0	3.673093	-1.828870	-0.827884
4	6	0	2.205513	-1.910478	-0.282876
5	6	0	2.145686	-2.084373	1.235386
6	6	0	3.202475	-1.845490	2.027668
7	7	0	3.929689	0.480382	0.126608
8	6	0	4.585235	1.555814	-0.413352
9	6	0	3.702173	-1.252914	-2.257685
10	6	0	4.359235	-3.211866	-0.837118
11	6	0	3.948606	2.901398	-0.184590
12	8	0	5.631232	1.444921	-1.049526
13	6	0	4.770985	4.028865	-0.311149
14	6	0	4.253955	5.306512	-0.111152
15	6	0	2.902706	5.474255	0.202607
16	6	0	2.071436	4.358073	0.310161
17	6	0	2.591115	3.077545	0.118600
18	6	0	1.346611	-2.959221	-1.036576
19	6	0	-0.212642	-2.946425	-0.854705
20	6	0	-0.759365	-1.737742	-0.067820
21	6	0	-0.396505	-1.758494	1.418349
22	6	0	-2.269680	-1.421931	-0.246492
23	6	0	-2.580944	-0.075990	0.536989
24	6	0	-2.419080	-0.347967	2.044763
25	6	0	-1.148700	-1.119569	2.328931
26	6	0	-2.723315	-1.077936	-1.686564
27	6	0	-3.886609	-0.066688	-1.529402
28	6	0	-4.003697	0.251845	-0.005782
29	6	0	-4.641337	1.640188	0.220601
30	6	0	-4.717448	2.080141	1.693379
31	7	0	-5.964846	1.671467	-0.477713

32	6	0	-7.059961	1.036040	0.258292
33	6	0	-6.347162	3.013199	-0.910412
34	6	0	0.831945	-2.542425	1.846471
35	8	0	-5.089202	-0.594547	-2.069128
36	6	0	-1.604060	1.069188	0.161947
37	6	0	-3.145317	-2.603017	0.245119
38	1	0	-4.690830	-0.486035	0.421625
39	1	0	1.767937	-0.926102	-0.509633
40	8	0	-0.661947	-4.161965	-0.237117
41	1	0	-0.233729	-0.889297	-0.528105
42	1	0	5.264444	-2.259330	1.580984
43	1	0	4.980825	-0.645288	2.192682
44	1	0	5.493755	-0.765896	-0.311580
45	1	0	3.092355	-1.979007	3.103830
46	1	0	3.197650	0.655343	0.801742
47	1	0	4.736167	-1.093483	-2.582605
48	1	0	3.190213	-0.286892	-2.311437
49	1	0	3.230920	-1.928026	-2.979692
50	1	0	5.432893	-3.106509	-1.036244
51	1	0	4.239467	-3.741118	0.113618
52	1	0	3.946593	-3.849764	-1.625874
53	1	0	5.813377	3.876176	-0.570526
54	1	0	4.903116	6.173128	-0.203426
55	1	0	2.497464	6.471165	0.354596
56	1	0	1.015696	4.482891	0.535344
57	1	0	1.921493	2.222921	0.171824
58	1	0	1.554435	-2.848542	-2.105747
59	1	0	1.690384	-3.963390	-0.759899
60	1	0	-0.653766	-2.887416	-1.860812
61	1	0	-2.397570	0.591540	2.613492
62	1	0	-3.283034	-0.905516	2.436646
63	1	0	-0.842555	-1.160988	3.374806
64	1	0	-1.907947	-0.636719	-2.273122
65	1	0	-3.076339	-1.956138	-2.235323
66	1	0	-3.633806	0.866436	-2.060576
67	1	0	-4.032765	2.378906	-0.315399
68	1	0	-5.174055	1.312253	2.327141
69	1	0	-3.719606	2.284503	2.091441
70	1	0	-5.304533	2.999766	1.800342
71	1	0	-7.365861	1.590944	1.161743
72	1	0	-7.933667	0.963297	-0.399002
73	1	0	-6.781763	0.021068	0.554244
74	1	0	-6.545281	3.710602	-0.075792
75	1	0	-5.556906	3.441889	-1.535085

76	1	0	−7.260237	2.952361	−1.513116
77	1	0	0.662393	−3.592671	1.573658
78	1	0	0.918233	−2.509163	2.937965
79	1	0	−5.738404	0.112669	−1.879619
80	1	0	−1.948473	2.013630	0.597617
81	1	0	−0.602932	0.888087	0.564264
82	1	0	−1.515405	1.222334	−0.918293
83	1	0	−2.970252	−2.849494	1.295253
84	1	0	−4.209952	−2.404419	0.098221
85	1	0	−2.897787	−3.495343	−0.332115
86	1	0	−0.394028	−4.894853	−0.813056

Cartesian coordinates of **2a-2**

Center Number	Atomic Number	Atomic Type	Coordinates (Angstroms) X	Y	Z
1	6	0	4.558655	−1.419235	1.517546
2	6	0	4.484322	−0.880337	0.082638
3	6	0	3.663203	−1.834135	−0.840872
4	6	0	2.195937	−1.910116	−0.292648
5	6	0	2.140242	−2.072793	1.227782
6	6	0	3.200411	−1.833588	2.015831
7	7	0	3.930699	0.476748	0.108447
8	6	0	4.594434	1.553333	−0.418143
9	6	0	3.691118	−1.261858	−2.272314
10	6	0	4.344294	−3.219667	−0.848539
11	6	0	3.962889	2.899935	−0.178894
12	8	0	5.643380	1.444636	−1.049710
13	6	0	4.791818	4.024449	−0.287051
14	6	0	4.280541	5.302363	−0.074116
15	6	0	2.928511	5.473616	0.234267
16	6	0	2.090729	4.360659	0.323202
17	6	0	2.604684	3.079796	0.118711
18	6	0	1.337790	−2.958476	−1.047179
19	6	0	−0.208708	−2.964257	−0.841189
20	6	0	−0.756770	−1.735657	−0.068006
21	6	0	−0.411984	−1.770227	1.422327
22	6	0	−2.263705	−1.410506	−0.269229
23	6	0	−2.602653	−0.096480	0.553458
24	6	0	−2.477900	−0.425876	2.052675
25	6	0	−1.196276	−1.178505	2.338580
26	6	0	−2.689582	−1.017416	−1.705486

27	6	0	−3.866019	−0.023203	−1.540893
28	6	0	−4.011676	0.253720	−0.011740
29	6	0	−4.647774	1.638957	0.238872
30	6	0	−4.757416	2.037358	1.721244
31	7	0	−5.953307	1.694468	−0.490588
32	6	0	−7.071183	1.056506	0.208364
33	6	0	−6.313245	3.048071	−0.906366
34	6	0	0.832111	−2.530209	1.851796
35	8	0	−5.053391	−0.553569	−2.110625
36	6	0	−1.619273	1.061981	0.241915
37	6	0	−3.144002	−2.611182	0.168070
38	1	0	−4.716650	−0.488336	0.378603
39	1	0	1.763394	−0.923454	−0.521353
40	8	0	−0.510644	−4.227531	−0.230423
41	1	0	−0.222935	−0.888195	−0.519882
42	1	0	5.256454	−2.267867	1.562022
43	1	0	4.990400	−0.649251	2.170703
44	1	0	5.489331	−0.775361	−0.334446
45	1	0	3.092478	−1.959259	3.093226
46	1	0	3.197724	0.650559	0.782903
47	1	0	3.182393	−0.294058	−2.327595
48	1	0	4.725235	−1.106805	−2.599328
49	1	0	3.216679	−1.937493	−2.991394
50	1	0	5.418161	−3.118291	−1.049174
51	1	0	4.223393	−3.747049	0.103005
52	1	0	3.927375	−3.857522	−1.634616
53	1	0	5.834834	3.869131	−0.542338
54	1	0	4.934902	6.166501	−0.151847
55	1	0	2.527846	6.470749	0.396654
56	1	0	1.034435	4.488611	0.544258
57	1	0	1.930852	2.227695	0.157815
58	1	0	1.526374	−2.828769	−2.116839
59	1	0	1.677188	−3.968212	−0.796031
60	1	0	−0.671749	−2.942619	−1.839457
61	1	0	−2.493209	0.489478	2.659258
62	1	0	−3.341434	−1.016004	2.396879
63	1	0	−0.903075	−1.236273	3.387251
64	1	0	−1.862288	−0.547633	−2.250213
65	1	0	−3.018703	−1.873774	−2.301590
66	1	0	−3.616731	0.925179	−2.044877
67	1	0	−4.022649	2.388780	−0.261263
68	1	0	−5.229836	1.253224	2.322799
69	1	0	−3.768829	2.228638	2.147801
70	1	0	−5.345379	2.954830	1.840383

71	1	0	-7.930203	1.004241	-0.469772
72	1	0	-6.809743	0.033504	0.492416
73	1	0	-7.391964	1.598044	1.114559
74	1	0	-5.506189	3.479570	-1.507063
75	1	0	-7.214377	3.007628	-1.528252
76	1	0	-6.520935	3.731914	-0.063053
77	1	0	0.675532	-3.585151	1.591740
78	1	0	0.923726	-2.480045	2.942288
79	1	0	-5.711890	0.146157	-1.926686
80	1	0	-1.969821	1.985541	0.715348
81	1	0	-0.623194	0.862350	0.646407
82	1	0	-1.519563	1.263079	-0.829376
83	1	0	-2.868986	-2.995802	1.154884
84	1	0	-4.208692	-2.369310	0.178171
85	1	0	-3.050375	-3.420348	-0.567485
86	1	0	-1.466961	-4.277442	-0.092092

Cartesian coordinates of **2a-3**

Center Number	Atomic Number	Atomic Type	Coordinates (Angstroms) X	Y	Z
1	6	0	4.532348	-1.357397	1.620026
2	6	0	4.504171	-0.780482	0.197544
3	6	0	3.745541	-1.728266	-0.786291
4	6	0	2.261151	-1.857110	-0.297722
5	6	0	2.156260	-2.099633	1.210353
6	6	0	3.175662	-1.849453	2.046937
7	7	0	3.926486	0.564276	0.240959
8	6	0	4.508431	1.652791	-0.345624
9	6	0	3.810955	-1.117451	-2.200892
10	6	0	4.462471	-3.095400	-0.803974
11	6	0	3.839017	2.980341	-0.096777
12	8	0	5.508577	1.569114	-1.056544
13	6	0	4.093578	4.004249	-1.019028
14	6	0	3.511379	5.259637	-0.861569
15	6	0	2.677652	5.513229	0.230522
16	6	0	2.434569	4.506011	1.165783
17	6	0	3.011316	3.245915	1.003241
18	6	0	1.447882	-2.879767	-1.130557
19	6	0	-0.105879	-2.905803	-0.985811
20	6	0	-0.695616	-1.750361	-0.132875
21	6	0	-0.408183	-1.917024	1.359335

22	6	0	−2.196125	−1.420791	−0.370427
23	6	0	−2.597879	−0.208055	0.571081
24	6	0	−2.536343	−0.697294	2.029816
25	6	0	−1.246008	−1.443024	2.295960
26	6	0	−2.556605	−0.876817	−1.774555
27	6	0	−3.761632	0.073237	−1.562430
28	6	0	−3.985022	0.183199	−0.021709
29	6	0	−4.653789	1.527035	0.343402
30	6	0	−4.857844	1.753318	1.851576
31	7	0	−5.914716	1.657177	−0.452078
32	6	0	−7.061760	0.930049	0.096342
33	6	0	−6.269897	3.047504	−0.725669
34	6	0	0.854507	−2.661827	1.759176
35	8	0	−4.909041	−0.413897	−2.243390
36	6	0	−1.622659	0.989705	0.430873
37	6	0	−3.080660	−2.669475	−0.113459
38	1	0	−4.695583	−0.603789	0.253206
39	1	0	1.818951	−0.868247	−0.496634
40	8	0	−0.418308	−4.216618	−0.491988
41	1	0	−0.153026	−0.861894	−0.484672
42	1	0	5.263760	−2.176828	1.674911
43	1	0	4.899845	−0.588312	2.312315
44	1	0	5.522696	−0.643348	−0.174854
45	1	0	3.038886	−2.036342	3.112174
46	1	0	3.083791	0.687147	0.783801
47	1	0	3.408613	−1.799487	−2.956846
48	1	0	4.848026	−0.895359	−2.472703
49	1	0	3.252441	−0.177644	−2.259954
50	1	0	5.538433	−2.962803	−0.972621
51	1	0	4.328686	−3.646048	0.132434
52	1	0	4.083212	−3.725900	−1.614724
53	1	0	4.756306	3.790106	−1.850913
54	1	0	3.710342	6.042785	−1.588272
55	1	0	2.226503	6.493856	0.357010
56	1	0	1.802893	4.702408	2.027986
57	1	0	2.836636	2.487336	1.762274
58	1	0	1.677780	−2.706100	−2.185449
59	1	0	1.787927	−3.895548	−0.906269
60	1	0	−0.532374	−2.802730	−1.995219
61	1	0	−2.607641	0.144590	2.731140
62	1	0	−3.397810	−1.342639	2.261261
63	1	0	−0.989573	−1.592662	3.345153
64	1	0	−1.713148	−0.333269	−2.216076
65	1	0	−2.834302	−1.665169	−2.480665

66	1	0	-3.512023	1.073861	-1.951501
67	1	0	-4.009494	2.332223	-0.030188
68	1	0	-5.351407	0.900593	2.330378
69	1	0	-3.900508	1.906702	2.357377
70	1	0	-5.469245	2.644215	2.036364
71	1	0	-7.443418	1.350831	1.042509
72	1	0	-7.879592	0.956850	-0.632428
73	1	0	-6.802595	-0.118681	0.265413
74	1	0	-6.533970	3.625949	0.178739
75	1	0	-5.436399	3.552677	-1.224185
76	1	0	-7.133860	3.074383	-1.398995
77	1	0	0.750181	-3.696735	1.407563
78	1	0	0.918179	-2.700275	2.852190
79	1	0	-5.586521	0.259099	-2.032688
80	1	0	-2.007221	1.850111	0.989129
81	1	0	-0.639298	0.760830	0.848899
82	1	0	-1.485659	1.311410	-0.606243
83	1	0	-2.861984	-3.150519	0.844943
84	1	0	-4.147492	-2.439394	-0.145683
85	1	0	-2.929847	-3.398176	-0.919995
86	1	0	-1.378242	-4.283777	-0.391775

Cartesian coordinates of **2a-4**

Center Number	Atomic Number	Atomic Type	Coordinates (Angstroms) X	Y	Z
1	6	0	4.558404	-1.417298	1.535632
2	6	0	4.487254	-0.874789	0.101671
3	6	0	3.672884	-1.829071	-0.827830
4	6	0	2.205281	-1.910617	-0.282889
5	6	0	2.145371	-2.084566	1.235376
6	6	0	3.202114	-1.845630	2.027712
7	7	0	3.929572	0.480247	0.126501
8	6	0	4.585421	1.555599	-0.413294
9	6	0	3.702016	-1.253150	-2.257642
10	6	0	4.358954	-3.212097	-0.837067
11	6	0	3.948989	2.901296	-0.184625
12	8	0	5.631501	1.444504	-1.049289
13	6	0	2.591414	3.077665	0.118098
14	6	0	2.071907	4.358267	0.309626
15	6	0	2.903425	5.474301	0.202495
16	6	0	4.254750	5.306348	-0.110818

17	6	0	4.771619	4.028629	-0.310788
18	6	0	1.346389	-2.959368	-1.036553
19	6	0	-0.212863	-2.946393	-0.854800
20	6	0	-0.759501	-1.737743	-0.067789
21	6	0	-0.396811	-1.758741	1.418384
22	6	0	-2.269784	-1.421761	-0.246619
23	6	0	-2.581026	-0.075927	0.537074
24	6	0	-2.419355	-0.348177	2.044820
25	6	0	-1.149012	-1.119868	2.329001
26	6	0	-2.723139	-1.077414	-1.686685
27	6	0	-3.886462	-0.066215	-1.529498
28	6	0	-4.003683	0.252095	-0.005848
29	6	0	-4.641236	1.640433	0.220680
30	6	0	-4.717380	2.080105	1.693546
31	7	0	-5.964684	1.671857	-0.477705
32	6	0	-6.347125	3.013734	-0.909850
33	6	0	-7.059760	1.036006	0.258000
34	6	0	0.831637	-2.542711	1.846419
35	8	0	-5.089027	-0.594052	-2.069360
36	6	0	-1.604018	1.069251	0.162340
37	6	0	-3.145546	-2.602865	0.244662
38	1	0	-4.690941	-0.485813	0.421322
39	1	0	1.767768	-0.926230	-0.509688
40	8	0	-0.662354	-4.161912	-0.237336
41	1	0	-0.233717	-0.889284	-0.527880
42	1	0	5.264131	-2.259373	1.581262
43	1	0	4.980367	-0.645292	2.192772
44	1	0	5.493605	-0.766198	-0.311387
45	1	0	3.091981	-1.979196	3.103863
46	1	0	3.197745	0.655408	0.801816
47	1	0	4.736021	-1.093706	-2.582526
48	1	0	3.230788	-1.928288	-2.979631
49	1	0	3.190063	-0.287121	-2.311429
50	1	0	5.432598	-3.106793	-1.036286
51	1	0	4.239236	-3.741315	0.113695
52	1	0	3.946227	-3.850012	-1.625772
53	1	0	1.921636	2.223147	0.170968
54	1	0	1.016107	4.483270	0.534435
55	1	0	2.498309	6.471266	0.354462
56	1	0	4.904100	6.172858	-0.202755
57	1	0	5.814069	3.875777	-0.569830
58	1	0	1.554284	-2.848816	-2.105718
59	1	0	1.690130	-3.963515	-0.759734
60	1	0	-0.653919	-2.887170	-1.860928

61	1	0	-2.397949	0.591190	2.613764
62	1	0	-3.283383	-0.905832	2.436386
63	1	0	-0.842851	-1.161325	3.374865
64	1	0	-1.907643	-0.636099	-2.272980
65	1	0	-3.076123	-1.955468	-2.235709
66	1	0	-3.633703	0.866983	-2.060546
67	1	0	-4.032577	2.379197	-0.315161
68	1	0	-3.719525	2.284198	2.091717
69	1	0	-5.304311	2.999809	1.800660
70	1	0	-5.174163	1.312162	2.327119
71	1	0	-6.545385	3.710747	-0.074937
72	1	0	-5.556869	3.442784	-1.534272
73	1	0	-7.260147	2.953039	-1.512643
74	1	0	-6.781449	0.020944	0.553538
75	1	0	-7.365765	1.590494	1.161671
76	1	0	-7.933422	0.963432	-0.399366
77	1	0	0.662063	-3.592921	1.573478
78	1	0	0.917947	-2.509585	2.937913
79	1	0	-5.738291	0.113095	-1.879822
80	1	0	-1.948372	2.013611	0.598241
81	1	0	-0.602910	0.888003	0.564637
82	1	0	-1.515340	1.222653	-0.917865
83	1	0	-2.970166	-2.849944	1.294601
84	1	0	-4.210195	-2.403960	0.098235
85	1	0	-2.898464	-3.494965	-0.333120
86	1	0	-0.393548	-4.894861	-0.812783

Cartesian coordinates of **2a-5**

Center Number	Atomic Number	Atomic Type	Coordinates (Angstroms) X	Y	Z
1	6	0	4.581063	-1.414079	1.541171
2	6	0	4.507488	-0.845936	0.116800
3	6	0	3.694999	-1.787972	-0.828219
4	6	0	2.227677	-1.887175	-0.284157
5	6	0	2.167226	-2.075956	1.232235
6	6	0	3.224253	-1.847585	2.027310
7	7	0	3.951849	0.506975	0.175022
8	6	0	4.537240	1.586281	-0.425521
9	6	0	3.717086	-1.191331	-2.249835
10	6	0	4.388364	-3.167273	-0.859301
11	6	0	3.914586	2.928235	-0.137042

12	8	0	5.505378	1.482688	-1.177144
13	6	0	3.129711	3.200257	0.992476
14	6	0	2.597370	4.474216	1.193071
15	6	0	2.842892	5.489323	0.267030
16	6	0	3.634139	5.229008	-0.854670
17	6	0	4.171726	3.959259	-1.050624
18	6	0	1.378979	-2.937384	-1.048070
19	6	0	-0.180469	-2.940598	-0.867926
20	6	0	-0.739390	-1.739030	-0.079125
21	6	0	-0.377611	-1.758918	1.407434
22	6	0	-2.252441	-1.436709	-0.257900
23	6	0	-2.574591	-0.092386	0.524179
24	6	0	-2.409586	-0.361158	2.032241
25	6	0	-1.134477	-1.124507	2.317264
26	6	0	-2.709710	-1.098118	-1.698076
27	6	0	-3.881926	-0.097212	-1.540925
28	6	0	-4.000858	0.221541	-0.017533
29	6	0	-4.650714	1.604371	0.207755
30	6	0	-4.729464	2.045481	1.679954
31	7	0	-5.975183	1.622793	-0.489529
32	6	0	-7.064047	0.979417	0.248727
33	6	0	-6.369631	2.960304	-0.924344
34	6	0	0.852581	-2.539100	1.837545
35	8	0	-5.080331	-0.636237	-2.079306
36	6	0	-1.608731	1.060938	0.146850
37	6	0	-3.117806	-2.624945	0.234966
38	1	0	-4.681061	-0.522197	0.410876
39	1	0	1.779025	-0.905997	-0.503508
40	8	0	-0.618619	-4.162239	-0.254181
41	1	0	-0.221724	-0.884682	-0.537365
42	1	0	5.285570	-2.257843	1.572143
43	1	0	5.003728	-0.652688	2.209817
44	1	0	5.512140	-0.727737	-0.297374
45	1	0	3.113240	-1.993109	3.101884
46	1	0	3.138169	0.644952	0.757343
47	1	0	3.194035	-0.230643	-2.290745
48	1	0	4.748006	-1.013176	-2.572985
49	1	0	3.252044	-1.862370	-2.979720
50	1	0	5.461353	-3.053337	-1.057216
51	1	0	4.271432	-3.711710	0.083150
52	1	0	3.978599	-3.794777	-1.657744
53	1	0	2.954947	2.435113	1.744756
54	1	0	1.998472	4.674854	2.077368
55	1	0	2.426505	6.480933	0.423434

56	1	0	3.834853	6.018056	-1.574455
57	1	0	4.801406	3.739115	-1.906268
58	1	0	1.586881	-2.815238	-2.115941
59	1	0	1.731884	-3.940574	-0.779685
60	1	0	-0.620777	-2.883528	-1.874455
61	1	0	-2.393585	0.579478	2.599253
62	1	0	-3.269452	-0.923731	2.426125
63	1	0	-0.829788	-1.165401	3.363668
64	1	0	-1.898640	-0.650078	-2.285349
65	1	0	-3.055328	-1.979914	-2.245843
66	1	0	-3.637918	0.837589	-2.073121
67	1	0	-4.049105	2.347775	-0.329469
68	1	0	-5.179795	1.274920	2.315016
69	1	0	-3.732826	2.258063	2.076677
70	1	0	-5.323494	2.960755	1.786109
71	1	0	-7.937554	0.897145	-0.407733
72	1	0	-6.776468	-0.032208	0.547077
73	1	0	-7.374216	1.533836	1.151050
74	1	0	-7.282376	2.890314	-1.526615
75	1	0	-6.573710	3.657350	-0.090807
76	1	0	-5.583441	3.395039	-1.549945
77	1	0	0.935174	-2.508680	2.929446
78	1	0	0.687640	-3.589154	1.561236
79	1	0	-5.735587	0.065092	-1.888583
80	1	0	-1.959959	2.002762	0.582604
81	1	0	-0.604317	0.891798	0.544818
82	1	0	-1.524254	1.215123	-0.933588
83	1	0	-2.941860	-2.867954	1.285783
84	1	0	-4.184045	-2.436114	0.086785
85	1	0	-2.861853	-3.516124	-0.340451
86	1	0	-0.345615	-4.890718	-0.833300

Cartesian coordinates of **2a-6**

Center Number	Atomic Number	Atomic Type	Coordinates (Angstroms) X	Y	Z
1	6	0	4.581280	-1.413271	1.541260
2	6	0	4.507593	-0.845287	0.116835
3	6	0	3.695433	-1.787670	-0.828095
4	6	0	2.228101	-1.887037	-0.284140
5	6	0	2.167618	-2.075743	1.232267
6	6	0	3.224552	-1.847094	2.027383

7	7	0	3.951450	0.507441	0.174886
8	6	0	4.536583	1.586939	−0.425567
9	6	0	3.717527	−1.191329	−2.249837
10	6	0	4.389067	−3.166838	−0.858782
11	6	0	3.913510	2.928689	−0.137112
12	8	0	5.504824	1.483651	−1.177103
13	6	0	3.128586	3.200491	0.992429
14	6	0	2.595840	4.474281	1.193004
15	6	0	2.840998	5.489440	0.266921
16	6	0	3.632294	5.229348	−0.854797
17	6	0	4.170295	3.959770	−1.050724
18	6	0	1.379589	−2.937513	−1.047889
19	6	0	−0.179932	−2.940603	−0.868116
20	6	0	−0.738997	−1.739199	−0.079196
21	6	0	−0.377220	−1.759106	1.407380
22	6	0	−2.252064	−1.436964	−0.257914
23	6	0	−2.574274	−0.092652	0.524168
24	6	0	−2.409229	−0.361424	2.032227
25	6	0	−1.134122	−1.124768	2.317226
26	6	0	−2.709406	−1.098422	−1.698071
27	6	0	−3.881702	−0.097621	−1.540877
28	6	0	−4.000596	0.221155	−0.017490
29	6	0	−4.650549	1.603961	0.207793
30	6	0	−4.729212	2.045132	1.679979
31	7	0	−5.975070	1.622295	−0.489404
32	6	0	−7.063876	0.978869	0.248901
33	6	0	−6.369584	2.959763	−0.924308
34	6	0	0.853039	−2.539203	1.837476
35	8	0	−5.080106	−0.636769	−2.079181
36	6	0	−1.608483	1.060717	0.146834
37	6	0	−3.117424	−2.625240	0.234961
38	1	0	−4.680719	−0.522612	0.410985
39	1	0	1.779347	−0.905939	−0.503604
40	8	0	−0.618219	−4.162420	−0.254798
41	1	0	−0.221426	−0.884779	−0.537403
42	1	0	5.285994	−2.256858	1.572297
43	1	0	5.003734	−0.651725	2.209862
44	1	0	5.512220	−0.726784	−0.297318
45	1	0	3.113537	−1.992659	3.101950
46	1	0	3.137934	0.645220	0.757491
47	1	0	4.748452	−1.013124	−2.572970
48	1	0	3.252653	−1.862594	−2.979630
49	1	0	3.194327	−0.230744	−2.290981
50	1	0	5.462014	−3.052767	−1.056859

51	1	0	4.272354	-3.710985	0.083864
52	1	0	3.979300	-3.794669	-1.656967
53	1	0	2.954086	2.435315	1.744738
54	1	0	1.996909	4.674754	2.077314
55	1	0	2.424301	6.480923	0.423301
56	1	0	3.832733	6.018440	-1.574609
57	1	0	4.800024	3.739809	-1.906379
58	1	0	1.587795	-2.815911	-2.115772
59	1	0	1.732339	-3.940586	-0.778910
60	1	0	-0.619995	-2.883274	-1.874736
61	1	0	-2.393182	0.579227	2.599220
62	1	0	-3.269095	-0.923951	2.426178
63	1	0	-0.829472	-1.165757	3.363643
64	1	0	-1.898397	-0.650248	-2.285336
65	1	0	-3.054881	-1.980253	-2.245869
66	1	0	-3.637826	0.837183	-2.073115
67	1	0	-4.049017	2.347365	-0.329505
68	1	0	-5.179385	1.274540	2.315106
69	1	0	-3.732572	2.257884	2.076609
70	1	0	-5.323360	2.960329	1.786143
71	1	0	-7.937444	0.896674	-0.407492
72	1	0	-6.776279	-0.032796	0.547111
73	1	0	-7.373972	1.533194	1.151302
74	1	0	-6.573581	3.656896	-0.090826
75	1	0	-5.583465	3.394439	-1.550043
76	1	0	-7.282388	2.889705	-1.526486
77	1	0	0.688299	-3.589261	1.561063
78	1	0	0.935559	-2.508899	2.929391
79	1	0	-5.735337	0.064642	-1.888686
80	1	0	-1.959745	2.002529	0.582583
81	1	0	-0.604061	0.891634	0.544802
82	1	0	-1.524005	1.214931	-0.933602
83	1	0	-2.941830	-2.867982	1.285901
84	1	0	-4.183632	-2.436566	0.086383
85	1	0	-2.861110	-3.516501	-0.340154
86	1	0	-0.345794	-4.890682	-0.834461

Table S3 Relative energies and Boltzmann populations for **2b**

**Conformer**	**Energy (Hartree)**	**Δ*G* (kcal/mol)**	**Population (%)**
2b-1	-1620.847536	0.948	6.16
2b-2	-1620.847715	0.836	7.45

Conformer	Energy (Hartree)	Δ*G* (kcal/mol)	Population (%)
2b-3	-1620.848836	0.132	24.41
2b-4	-1620.849045	0.001	30.46
2b-5	-1620.849047	0.000	30.52
2b-6	-1620.845819	2.026	1.00

Cartesian coordinates of **2b-1**

Center Number	Atomic Number	Atomic Type	Coordinates (Angstroms) X	Y	Z
1	6	0	-3.150049	0.492943	2.327727
2	6	0	-3.906345	1.196750	1.188647
3	6	0	-2.921446	1.807794	0.142048
4	6	0	-2.077345	0.643319	-0.478749
5	6	0	-1.616393	-0.387388	0.540341
6	6	0	-2.101658	-0.445417	1.789184
7	7	0	-4.843224	0.250374	0.579173
8	6	0	-6.181640	0.514547	0.452412
9	6	0	-3.738703	2.517768	-0.955366
10	6	0	-2.056688	2.865991	0.858776
11	6	0	-7.028082	-0.606840	-0.089696
12	8	0	-6.675559	1.598000	0.757699
13	6	0	-8.403662	-0.557087	0.172540
14	6	0	-9.248756	-1.560101	-0.296166
15	6	0	-8.731079	-2.618389	-1.047429
16	6	0	-7.365151	-2.665326	-1.331159
17	6	0	-6.517217	-1.665284	-0.854346
18	6	0	-0.900874	1.094810	-1.386814
19	6	0	0.465927	1.352329	-0.707649
20	6	0	1.421504	0.127584	-0.681427
21	6	0	0.865380	-1.023448	0.168530
22	6	0	2.878462	0.478773	-0.231938
23	6	0	3.723029	-0.856065	-0.086803
24	6	0	3.147812	-1.659261	1.089710
25	6	0	1.649654	-1.776267	0.958429
26	6	0	3.744805	1.294820	-1.228563
27	6	0	5.207831	0.864105	-0.978821
28	6	0	5.166397	-0.290431	0.065773
29	6	0	6.399232	-1.211124	-0.079862

30	6	0	6.414149	−2.429747	0.859588
31	7	0	7.631770	−0.368380	0.027174
32	6	0	8.031219	−0.038027	1.396401
33	6	0	8.760153	−0.914698	−0.721145
34	6	0	−0.604563	−1.410949	0.050101
35	8	0	5.989280	1.967007	−0.535365
36	6	0	3.645168	−1.734010	−1.362513
37	6	0	2.816124	1.278299	1.096433
38	1	0	5.232691	0.173333	1.055989
39	1	0	−2.772015	0.114367	−1.152630
40	8	0	1.153253	2.375660	−1.437233
41	1	0	1.464579	−0.218343	−1.727485
42	1	0	−2.696003	1.239471	2.994790
43	1	0	−3.869780	−0.055701	2.949230
44	1	0	−4.527841	2.001780	1.589829
45	1	0	−1.732701	−1.218018	2.464001
46	1	0	−4.531845	−0.707736	0.498312
47	1	0	−3.088055	3.008920	−1.687889
48	1	0	−4.384725	3.286641	−0.517855
49	1	0	−4.381701	1.814812	−1.493406
50	1	0	−1.443267	3.425563	0.143496
51	1	0	−2.698876	3.596839	1.365821
52	1	0	−1.386289	2.430675	1.604740
53	1	0	−8.786582	0.281729	0.744332
54	1	0	−10.312730	−1.515649	−0.079645
55	1	0	−9.390645	−3.398778	−1.417600
56	1	0	−6.959800	−3.475668	−1.931098
57	1	0	−5.462490	−1.698970	−1.114951
58	1	0	−1.191977	2.021943	−1.893146
59	1	0	−0.757586	0.369438	−2.197195
60	1	0	0.299428	1.683817	0.324108
61	1	0	3.570920	−2.671386	1.127183
62	1	0	3.416013	−1.193316	2.049680
63	1	0	1.184792	−2.585612	1.521965
64	1	0	3.450150	1.094966	−2.263632
65	1	0	3.643533	2.369879	−1.079631
66	1	0	5.644130	0.473260	−1.913429
67	1	0	6.413190	−1.586001	−1.110452
68	1	0	6.237057	−2.145159	1.902498
69	1	0	5.641459	−3.150616	0.577626
70	1	0	7.376675	−2.952727	0.811740
71	1	0	7.192127	0.401767	1.942115
72	1	0	8.405006	−0.903469	1.970476
73	1	0	8.832758	0.708431	1.361599

74	1	0	8.474278	-1.065657	-1.767110
75	1	0	9.590639	-0.200111	-0.696817
76	1	0	9.136857	-1.875541	-0.323723
77	1	0	-0.750386	-2.341177	0.609080
78	1	0	-0.826998	-1.650571	-1.001696
79	1	0	6.871799	1.566498	-0.405461
80	1	0	4.312540	-2.597680	-1.264954
81	1	0	2.640081	-2.131176	-1.522990
82	1	0	3.942050	-1.192308	-2.265688
83	1	0	3.810968	1.494574	1.492333
84	1	0	2.348738	2.247910	0.903315
85	1	0	2.234519	0.769696	1.871840
86	1	0	0.606533	3.175910	-1.402024

Cartesian coordinates of **2b-2**

Center Number	Atomic Number	Atomic Type	Coordinates (Angstroms) X	Y	Z
1	6	0	-3.138365	0.357295	2.395752
2	6	0	-3.905275	1.117802	1.300328
3	6	0	-2.929011	1.783619	0.277637
4	6	0	-2.084827	0.655733	-0.406443
5	6	0	-1.613015	-0.423448	0.554980
6	6	0	-2.089577	-0.547130	1.802336
7	7	0	-4.849608	0.200985	0.661197
8	6	0	-6.170968	0.495120	0.470023
9	6	0	-3.753440	2.546620	-0.778336
10	6	0	-2.062667	2.806895	1.042276
11	6	0	-7.022428	-0.611370	-0.097137
12	8	0	-6.647253	1.600371	0.723392
13	6	0	-6.699738	-1.972616	-0.002573
14	6	0	-7.545568	-2.938895	-0.547848
15	6	0	-8.721563	-2.555101	-1.194488
16	6	0	-9.055959	-1.201349	-1.281659
17	6	0	-8.215478	-0.237433	-0.730044
18	6	0	-0.916847	1.158651	-1.298674
19	6	0	0.455072	1.384297	-0.617686
20	6	0	1.413558	0.162810	-0.666526
21	6	0	0.868248	-1.031672	0.128991
22	6	0	2.874849	0.493935	-0.215252
23	6	0	3.722394	-0.844906	-0.146951
24	6	0	3.160813	-1.707482	0.993358

25	6	0	1.661806	−1.821790	0.871564
26	6	0	3.729684	1.361770	−1.177701
27	6	0	5.195898	0.919582	−0.967967
28	6	0	5.166563	−0.285079	0.018668
29	6	0	6.398743	−1.196536	−0.182844
30	6	0	6.423240	−2.457206	0.698761
31	7	0	7.631807	−0.358298	−0.048744
32	6	0	8.042448	−0.088823	1.330500
33	6	0	8.754269	−0.870074	−0.829786
34	6	0	−0.601861	−1.417224	0.005482
35	8	0	5.983554	1.999171	−0.480442
36	6	0	3.632141	−1.656911	−1.465122
37	6	0	2.825415	1.223806	1.153402
38	1	0	5.242234	0.129904	1.029690
39	1	0	−2.781033	0.159479	−1.103211
40	8	0	1.132883	2.448450	−1.295613
41	1	0	1.446842	−0.128893	−1.729177
42	1	0	−2.683378	1.067076	3.101214
43	1	0	−3.853463	−0.227845	2.988340
44	1	0	−4.523588	1.901186	1.746245
45	1	0	−1.712674	−1.351884	2.433849
46	1	0	−4.500778	−0.707166	0.390269
47	1	0	−3.106126	3.093609	−1.472883
48	1	0	−4.417755	3.272342	−0.297891
49	1	0	−4.379993	1.868434	−1.365607
50	1	0	−1.454809	3.403282	0.352288
51	1	0	−2.703938	3.509307	1.588834
52	1	0	−1.386701	2.336993	1.761762
53	1	0	−5.807150	−2.295729	0.527408
54	1	0	−7.289351	−3.991300	−0.460174
55	1	0	−9.378364	−3.308422	−1.621314
56	1	0	−9.974305	−0.898181	−1.777266
57	1	0	−8.463003	0.818020	−0.772854
58	1	0	−1.214859	2.109442	−1.754461
59	1	0	−0.778063	0.475840	−2.145900
60	1	0	0.298124	1.656834	0.432743
61	1	0	3.585853	−2.719249	0.976337
62	1	0	3.437659	−1.288905	1.972548
63	1	0	1.204609	−2.660338	1.397431
64	1	0	3.423150	1.216841	−2.218403
65	1	0	3.629683	2.427361	−0.970922
66	1	0	5.620182	0.575351	−1.926132
67	1	0	6.403132	−1.522583	−1.229996
68	1	0	6.250240	−2.221205	1.754377

69	1	0	5.652081	-3.167609	0.387405
70	1	0	7.387575	-2.973270	0.622082
71	1	0	8.845257	0.657028	1.322086
72	1	0	7.208400	0.328268	1.901153
73	1	0	8.418628	-0.979155	1.863425
74	1	0	9.584394	-0.156313	-0.780911
75	1	0	9.134976	-1.846972	-0.478057
76	1	0	8.459733	-0.975089	-1.878951
77	1	0	-0.739487	-2.374410	0.519263
78	1	0	-0.833494	-1.606015	-1.054604
79	1	0	6.867377	1.592523	-0.381799
80	1	0	4.305596	-2.520332	-1.422346
81	1	0	2.627220	-2.051614	-1.633104
82	1	0	3.913631	-1.067105	-2.342727
83	1	0	3.823920	1.423087	1.548905
84	1	0	2.353223	2.200712	1.017470
85	1	0	2.254631	0.673125	1.907870
86	1	0	0.583755	3.243451	-1.212303

Cartesian coordinates of **2b-3**

Center Number	Atomic Number	Atomic Type	Coordinates (Angstroms) X	Y	Z
1	6	0	-3.157977	0.507974	2.320598
2	6	0	-3.907850	1.206488	1.173604
3	6	0	-2.918543	1.805295	0.125022
4	6	0	-2.076595	0.632589	-0.482655
5	6	0	-1.618354	-0.389935	0.546987
6	6	0	-2.108312	-0.435889	1.794461
7	7	0	-4.848189	0.258638	0.570136
8	6	0	-6.186528	0.521820	0.446042
9	6	0	-3.728912	2.505077	-0.984064
10	6	0	-2.051083	2.870199	0.829311
11	6	0	-7.033367	-0.601135	-0.093445
12	8	0	-6.682043	1.604440	0.751635
13	6	0	-8.408096	-0.553257	0.173401
14	6	0	-9.253569	-1.557382	-0.292279
15	6	0	-8.737223	-2.615115	-1.045246
16	6	0	-7.372234	-2.660204	-1.333682
17	6	0	-6.523968	-1.658961	-0.859850
18	6	0	-0.893977	1.081161	-1.384264

19	6	0	0.458165	1.346270	−0.680960
20	6	0	1.420237	0.119188	−0.661192
21	6	0	0.867360	−1.034850	0.186760
22	6	0	2.876641	0.470137	−0.219238
23	6	0	3.725315	−0.861754	−0.078903
24	6	0	3.155235	−1.664203	1.101041
25	6	0	1.655931	−1.783679	0.976364
26	6	0	3.733380	1.288439	−1.222476
27	6	0	5.200240	0.858637	−0.987508
28	6	0	5.167626	−0.290422	0.063618
29	6	0	6.403477	−1.206651	−0.084631
30	6	0	6.426917	−2.420593	0.860447
31	7	0	7.632735	−0.357835	0.012169
32	6	0	8.038386	−0.021132	1.378459
33	6	0	8.759558	−0.903031	−0.739964
34	6	0	−0.604013	−1.419732	0.072008
35	8	0	5.984862	1.963885	−0.561983
36	6	0	3.643388	−1.743360	−1.352091
37	6	0	2.818852	1.272262	1.107910
38	1	0	5.237642	0.179108	1.050600
39	1	0	−2.767995	0.098387	−1.155969
40	8	0	1.080264	2.488431	−1.280892
41	1	0	1.460307	−0.234419	−1.707807
42	1	0	−2.704772	1.257805	2.984241
43	1	0	−3.881168	−0.035508	2.942702
44	1	0	−4.526784	2.016798	1.567733
45	1	0	−1.742240	−1.202086	2.478335
46	1	0	−4.536907	−0.699308	0.487811
47	1	0	−3.072201	2.995055	−1.711521
48	1	0	−4.380758	3.274507	−0.556270
49	1	0	−4.365489	1.797120	−1.523753
50	1	0	−1.405628	3.391885	0.115633
51	1	0	−2.694407	3.624463	1.299689
52	1	0	−1.410541	2.445345	1.607675
53	1	0	−8.789973	0.285303	0.746277
54	1	0	−10.316863	−1.514222	−0.072081
55	1	0	−9.397090	−3.396384	−1.413076
56	1	0	−6.967919	−3.470066	−1.935035
57	1	0	−5.470072	−1.691031	−1.124031
58	1	0	−1.168571	2.012487	−1.887307
59	1	0	−0.746270	0.342235	−2.183833
60	1	0	0.272269	1.660589	0.346692
61	1	0	3.579998	−2.675606	1.137718
62	1	0	3.426697	−1.196703	2.059147

63	1	0	1.194651	-2.590498	1.546330
64	1	0	3.441834	1.076251	-2.259337
65	1	0	3.633553	2.364170	-1.072008
66	1	0	5.623529	0.460625	-1.925100
67	1	0	6.413771	-1.586378	-1.113549
68	1	0	6.253868	-2.131802	1.902821
69	1	0	5.655353	-3.145381	0.585590
70	1	0	7.390866	-2.940574	0.810271
71	1	0	7.200689	0.417197	1.927465
72	1	0	8.418565	-0.883316	1.952919
73	1	0	8.836663	0.728308	1.336622
74	1	0	8.468997	-1.059214	-1.783879
75	1	0	9.587397	-0.185317	-0.722529
76	1	0	9.141424	-1.860779	-0.340482
77	1	0	-0.751207	-2.343372	0.641535
78	1	0	-0.824582	-1.672238	-0.977342
79	1	0	6.867407	1.562831	-0.430629
80	1	0	4.312878	-2.605272	-1.254575
81	1	0	2.638454	-2.143289	-1.506394
82	1	0	3.936575	-1.205709	-2.259216
83	1	0	3.814641	1.488762	1.501282
84	1	0	2.347868	2.239799	0.916653
85	1	0	2.241547	0.760290	1.884000
86	1	0	1.346866	2.243976	-2.181753

Cartesian coordinates of **2b-4**

Center Number	Atomic Number	Atomic Type	Coordinates (Angstroms) X	Y	Z
1	6	0	3.143552	-0.367902	2.393434
2	6	0	3.905033	-1.124046	1.290777
3	6	0	2.925425	-1.776370	0.263720
4	6	0	2.085004	-0.638685	-0.408569
5	6	0	1.614100	0.432002	0.564075
6	6	0	2.094037	0.542786	1.811289
7	7	0	4.855043	-0.206779	0.659211
8	6	0	6.175359	-0.503735	0.468200
9	6	0	3.744143	-2.529989	-0.803583
10	6	0	2.054680	-2.806712	1.014635
11	6	0	7.028446	0.601113	-0.100759
12	8	0	6.651289	-1.608728	0.723091
13	6	0	6.709781	1.963141	-0.004443

14	6	0	7.556867	2.927579	−0.551177
15	6	0	8.730148	2.541117	−1.201133
16	6	0	9.060582	1.186491	−1.290044
17	6	0	8.218914	0.224487	−0.736839
18	6	0	0.912207	−1.137497	−1.296800
19	6	0	−0.445474	−1.371773	−0.592573
20	6	0	−1.411760	−0.149509	−0.646593
21	6	0	−0.871366	1.047807	0.147922
22	6	0	−2.871997	−0.483252	−0.203316
23	6	0	−3.725536	0.851077	−0.139258
24	6	0	−3.170630	1.713377	1.004999
25	6	0	−1.670601	1.832471	0.890399
26	6	0	−3.715527	−1.354464	−1.172966
27	6	0	−5.186009	−0.915524	−0.979152
28	6	0	−5.167837	0.282694	0.015556
29	6	0	−6.404235	1.188035	−0.187782
30	6	0	−6.439612	2.442678	0.701685
31	7	0	−7.632799	0.341291	−0.065687
32	6	0	−8.049826	0.062014	1.310185
33	6	0	−8.754130	0.851990	−0.849777
34	6	0	0.599904	1.432406	0.029542
35	8	0	−5.976045	−1.999799	−0.511884
36	6	0	−3.631930	1.667760	−1.454585
37	6	0	−2.826410	−1.216147	1.164030
38	1	0	−5.246980	−0.139451	1.023081
39	1	0	2.779185	−0.136786	−1.103516
40	8	0	−1.057257	−2.547055	−1.136261
41	1	0	−1.442306	0.151065	−1.709801
42	1	0	2.688521	−1.081103	3.095161
43	1	0	3.861651	0.211958	2.987651
44	1	0	4.519065	−1.913980	1.730670
45	1	0	1.718766	1.340845	2.452460
46	1	0	4.507857	0.699989	0.381827
47	1	0	3.091630	−3.073985	−1.495159
48	1	0	4.412312	−3.258058	−0.332144
49	1	0	4.366247	−1.847084	−1.390708
50	1	0	1.415964	−3.365243	0.323049
51	1	0	2.696471	−3.534332	1.526965
52	1	0	1.407186	−2.345106	1.765767
53	1	0	5.819398	2.287849	0.528287
54	1	0	7.303860	3.980663	−0.461878
55	1	0	9.387999	3.292954	−1.629015
56	1	0	9.976883	0.881136	−1.788138
57	1	0	8.463481	−0.831625	−0.780636

58	1	0	1.194828	-2.091341	-1.750688
59	1	0	0.769155	-0.439160	-2.132820
60	1	0	-0.269209	-1.629008	0.452590
61	1	0	-3.598625	2.723834	0.987310
62	1	0	-3.450487	1.292565	1.982191
63	1	0	-1.218421	2.668983	1.423692
64	1	0	-3.411406	-1.197003	-2.215949
65	1	0	-3.616423	-2.420579	-0.964507
66	1	0	-5.596480	-0.563468	-1.940496
67	1	0	-6.404808	1.520622	-1.232946
68	1	0	-6.271311	2.200708	1.756647
69	1	0	-5.670165	3.158700	0.399153
70	1	0	-7.405839	2.954737	0.622931
71	1	0	-8.848364	-0.688104	1.292992
72	1	0	-7.216915	-0.353614	1.883545
73	1	0	-8.433755	0.947539	1.845197
74	1	0	-9.580694	0.133738	-0.809205
75	1	0	-9.141179	1.824722	-0.494045
76	1	0	-8.454805	0.964715	-1.896796
77	1	0	0.737549	2.383164	0.555211
78	1	0	0.830208	1.635262	-1.028413
79	1	0	-6.860499	-1.593683	-0.412439
80	1	0	-4.309306	2.527980	-1.411806
81	1	0	-2.627936	2.067661	-1.615513
82	1	0	-3.907750	1.081652	-2.336801
83	1	0	-3.825636	-1.416190	1.557342
84	1	0	-2.350206	-2.190681	1.029992
85	1	0	-2.260094	-0.661793	1.918837
86	1	0	-1.320044	-2.351113	-2.049963

Cartesian coordinates of **2b-5**

Center Number	Atomic Number	Atomic Type	Coordinates (Angstroms) X	Y	Z
1	6	0	-3.143777	0.367762	2.393148
2	6	0	-3.905087	1.124072	1.290488
3	6	0	-2.925379	1.776420	0.263542
4	6	0	-2.084932	0.638760	-0.408754
5	6	0	-1.614135	-0.432024	0.563861
6	6	0	-2.094233	-0.542891	1.811011
7	7	0	-4.855114	0.206932	0.658768
8	6	0	-6.175505	0.503776	0.468066

9	6	0	−3.744023	2.530103	−0.803769
10	6	0	−2.054620	2.806730	1.014476
11	6	0	−7.028606	−0.601143	−0.100737
12	8	0	−6.651481	1.608717	0.723075
13	6	0	−6.709681	−1.963128	−0.004793
14	6	0	−7.556815	−2.927614	−0.551370
15	6	0	−8.730419	−2.541241	−1.200796
16	6	0	−9.061114	−1.186651	−1.289333
17	6	0	−8.219389	−0.224609	−0.736296
18	6	0	−0.912023	1.137606	−1.296782
19	6	0	0.445528	1.371857	−0.592351
20	6	0	1.411865	0.149617	−0.646499
21	6	0	0.871416	−1.047778	0.147884
22	6	0	2.872076	0.483290	−0.203170
23	6	0	3.725604	−0.851063	−0.139232
24	6	0	3.170673	−1.713456	1.004949
25	6	0	1.670638	−1.832492	0.890315
26	6	0	3.715597	1.354523	−1.172792
27	6	0	5.186077	0.915609	−0.978967
28	6	0	5.167931	−0.282705	0.015637
29	6	0	6.404332	−1.188074	−0.187785
30	6	0	6.439723	−2.442726	0.701674
31	7	0	7.632956	−0.341412	−0.065750
32	6	0	8.754167	−0.852128	−0.849975
33	6	0	8.050125	−0.062279	1.310098
34	6	0	−0.599854	−1.432385	0.029453
35	8	0	5.976016	1.999898	−0.511542
36	6	0	3.631949	−1.667610	−1.454628
37	6	0	2.826534	1.216115	1.164227
38	1	0	5.247104	0.139325	1.023206
39	1	0	−2.779059	0.136977	−1.103847
40	8	0	1.057330	2.547255	−1.135752
41	1	0	1.442411	−0.150816	−1.709743
42	1	0	−2.688791	1.080833	3.095035
43	1	0	−3.861970	−0.212142	2.987212
44	1	0	−4.519112	1.914015	1.730383
45	1	0	−1.719040	−1.340982	2.452191
46	1	0	−4.508010	−0.699949	0.381636
47	1	0	−3.091483	3.074072	−1.495347
48	1	0	−4.412123	3.258237	−0.332327
49	1	0	−4.366184	1.847243	−1.390883
50	1	0	−1.416122	3.365453	0.322845
51	1	0	−2.696397	3.534179	1.527067
52	1	0	−1.406907	2.345067	1.765386

53	1	0	-5.819041	-2.287828	0.527519
54	1	0	-7.303568	-3.980663	-0.462367
55	1	0	-9.388310	-3.293105	-1.628566
56	1	0	-9.977671	-0.881360	-1.786996
57	1	0	-8.464138	0.831474	-0.779820
58	1	0	-1.194606	2.091466	-1.750678
59	1	0	-0.768845	0.439293	-2.132799
60	1	0	0.269130	1.628898	0.452838
61	1	0	3.598618	-2.723937	0.987159
62	1	0	3.450513	-1.292780	1.982200
63	1	0	1.218449	-2.669039	1.423558
64	1	0	3.411474	1.197005	-2.215765
65	1	0	3.616424	2.420639	-0.964390
66	1	0	5.596632	0.563680	-1.940316
67	1	0	6.404843	-1.520647	-1.232949
68	1	0	6.271446	-2.200764	1.756641
69	1	0	5.670281	-3.158765	0.399162
70	1	0	7.405955	-2.954778	0.622908
71	1	0	9.141265	-1.824865	-0.494286
72	1	0	8.454710	-0.964873	-1.896954
73	1	0	9.580750	-0.133883	-0.809539
74	1	0	7.217329	0.353457	1.883551
75	1	0	8.433957	-0.947893	1.845036
76	1	0	8.848793	0.687707	1.292900
77	1	0	-0.737490	-2.383116	0.555172
78	1	0	-0.830104	-1.635326	-1.028507
79	1	0	6.860546	1.593911	-0.412339
80	1	0	4.309143	-2.527975	-1.411856
81	1	0	2.627902	-2.067322	-1.615701
82	1	0	3.907963	-1.081494	-2.336775
83	1	0	3.825773	1.416062	1.557540
84	1	0	2.350396	2.190686	1.030239
85	1	0	2.260181	0.661748	1.918995
86	1	0	1.319315	2.351820	-2.049799

Cartesian coordinates of **2b-6**

Center Number	Atomic Number	Atomic Type	Coordinates (Angstroms) X	Y	Z
1	6	0	4.560948	-1.386500	1.678927
2	6	0	4.515671	-0.917559	0.216943
3	6	0	3.706661	-1.910724	-0.676887

4	6	0	2.232758	−1.949791	−0.147790
5	6	0	2.152406	−2.048950	1.370983
6	6	0	3.194743	−1.781907	2.172056
7	7	0	3.971149	0.441775	0.163979
8	6	0	4.651213	1.485302	−0.408162
9	6	0	3.757172	−1.409614	−2.133285
10	6	0	4.382313	−3.297093	−0.608754
11	6	0	4.020790	2.844971	−0.259751
12	8	0	5.713050	1.335606	−1.008898
13	6	0	4.855399	3.959111	−0.419947
14	6	0	4.344810	5.248553	−0.291653
15	6	0	2.988387	5.440826	−0.016771
16	6	0	2.145333	4.337197	0.123402
17	6	0	2.658264	3.045324	0.003396
18	6	0	1.361724	−3.019571	−0.853464
19	6	0	−0.196399	−2.982897	−0.673386
20	6	0	−0.738929	−1.689992	−0.020336
21	6	0	−0.395003	−1.683259	1.478788
22	6	0	−2.258429	−1.403544	−0.209984
23	6	0	−2.610982	−0.068237	0.578501
24	6	0	−2.453213	−0.325048	2.086197
25	6	0	−1.171272	−1.063478	2.381907
26	6	0	−2.737467	−1.061381	−1.645252
27	6	0	−3.931123	−0.093317	−1.476564
28	6	0	−4.044625	0.221669	0.045442
29	6	0	−4.716852	1.593482	0.277699
30	6	0	−4.796616	2.037122	1.749263
31	7	0	−6.044723	1.590639	−0.412192
32	6	0	−6.463343	2.920590	−0.846534
33	6	0	−7.118926	0.928822	0.330381
34	6	0	0.819293	−2.464510	1.962804
35	8	0	−5.122514	−0.666947	−1.999410
36	6	0	−1.663129	1.099670	0.198814
37	6	0	−3.080781	−2.623364	0.277181
38	1	0	−4.707435	−0.536302	0.477864
39	1	0	1.807608	−0.974277	−0.426567
40	8	0	−0.812238	−3.123796	−1.957844
41	1	0	−0.209756	−0.861519	−0.509846
42	1	0	5.259689	−2.229569	1.779497
43	1	0	4.977784	−0.584126	2.301921
44	1	0	5.529380	−0.838027	−0.184404
45	1	0	3.065537	−1.863416	3.251409
46	1	0	3.223104	0.655718	0.809510
47	1	0	3.278378	−2.113860	−2.821655

48	1	0	4.796283	−1.285668	−2.457650
49	1	0	3.261648	−0.439802	−2.241207
50	1	0	3.974796	−3.970005	−1.370420
51	1	0	5.458803	−3.207815	−0.800379
52	1	0	4.249767	−3.779620	0.365037
53	1	0	5.902100	3.786666	−0.647659
54	1	0	5.003156	6.105061	−0.409597
55	1	0	2.588305	6.446748	0.079182
56	1	0	1.085800	4.480228	0.317726
57	1	0	1.979875	2.199492	0.080299
58	1	0	1.723374	−4.019490	−0.582419
59	1	0	1.520296	−2.905706	−1.930201
60	1	0	−0.502224	−3.835159	−0.049297
61	1	0	−2.460706	0.616258	2.651738
62	1	0	−3.304090	−0.905059	2.475478
63	1	0	−0.880139	−1.107849	3.431991
64	1	0	−1.938122	−0.598231	−2.232610
65	1	0	−3.051946	−1.948398	−2.195805
66	1	0	−3.723200	0.848070	−2.012749
67	1	0	−4.130028	2.346043	−0.263214
68	1	0	−5.229655	1.260610	2.389150
69	1	0	−3.802827	2.271272	2.141085
70	1	0	−5.407793	2.941129	1.855166
71	1	0	−6.676142	3.615686	−0.013286
72	1	0	−5.687229	3.367670	−1.476035
73	1	0	−7.377126	2.834520	−1.445217
74	1	0	−6.813149	−0.078385	0.625527
75	1	0	−7.434061	1.476571	1.235244
76	1	0	−7.994254	0.832975	−0.321850
77	1	0	0.661406	−3.532178	1.740942
78	1	0	0.873940	−2.395444	3.054340
79	1	0	−5.791875	0.021875	−1.815584
80	1	0	−2.023329	2.035418	0.640800
81	1	0	−0.653819	0.938590	0.588907
82	1	0	−1.589023	1.255182	−0.881751
83	1	0	−4.155277	−2.466739	0.153274
84	1	0	−2.821764	−3.486241	−0.343580
85	1	0	−2.882827	−2.884416	1.321065
86	1	0	−0.509382	−3.965699	−2.332448

Myribuxine C (**3**)

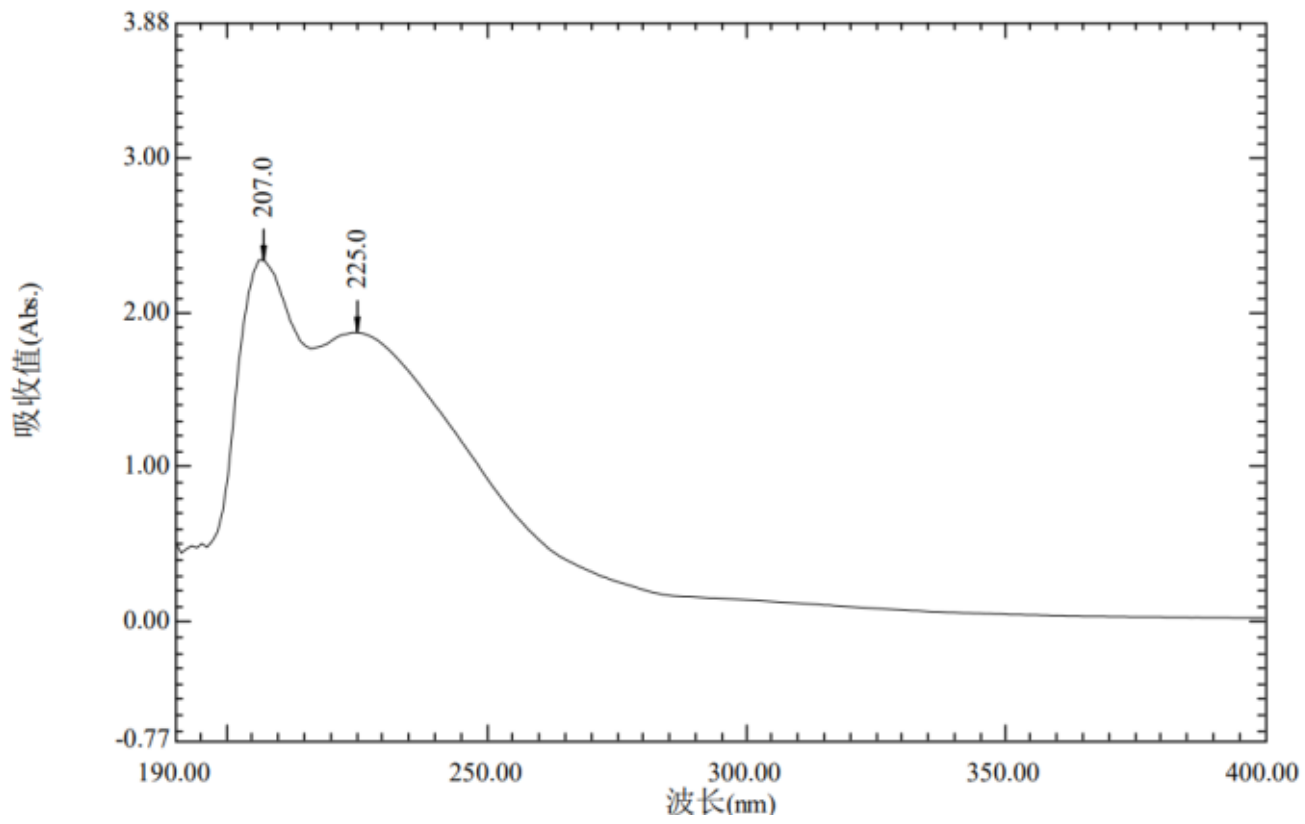

Figure S3.1 UV spectrum of compound **3**

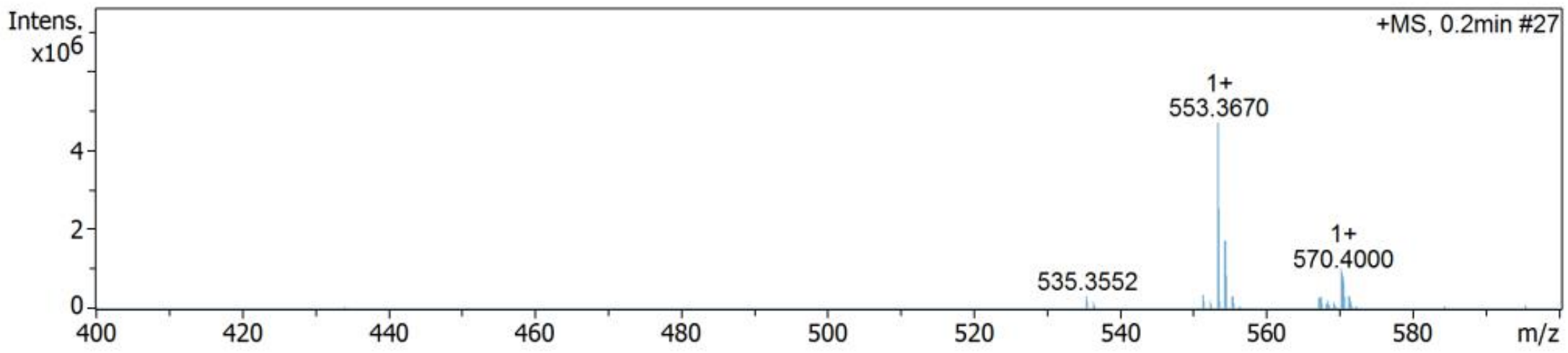

Figure S3.2 HR-ESI-MS spectrum of compound **3**

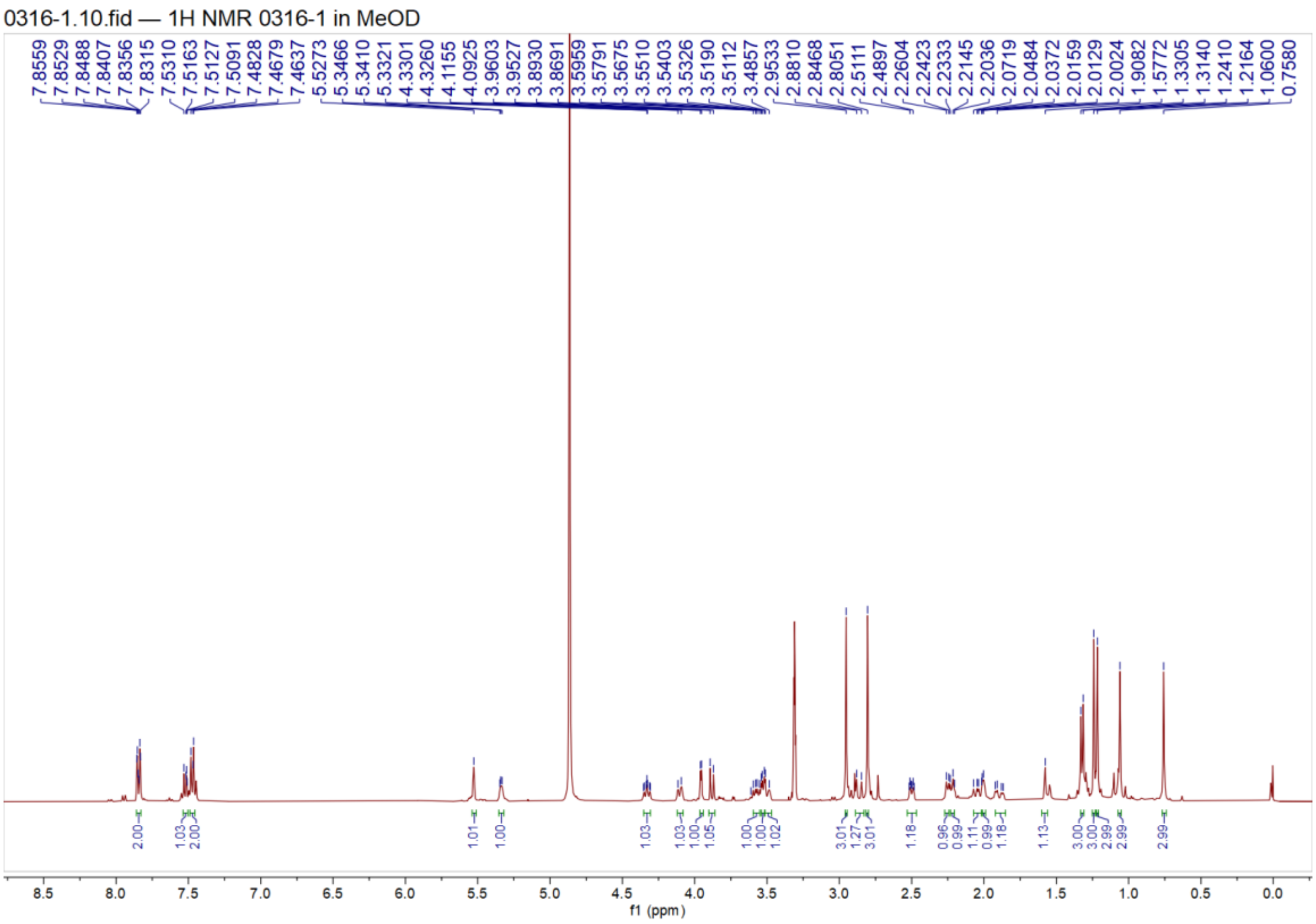

Figure S3.3 ^{1}H NMR spectrum of compound **3** (400 MHz, MeOD)

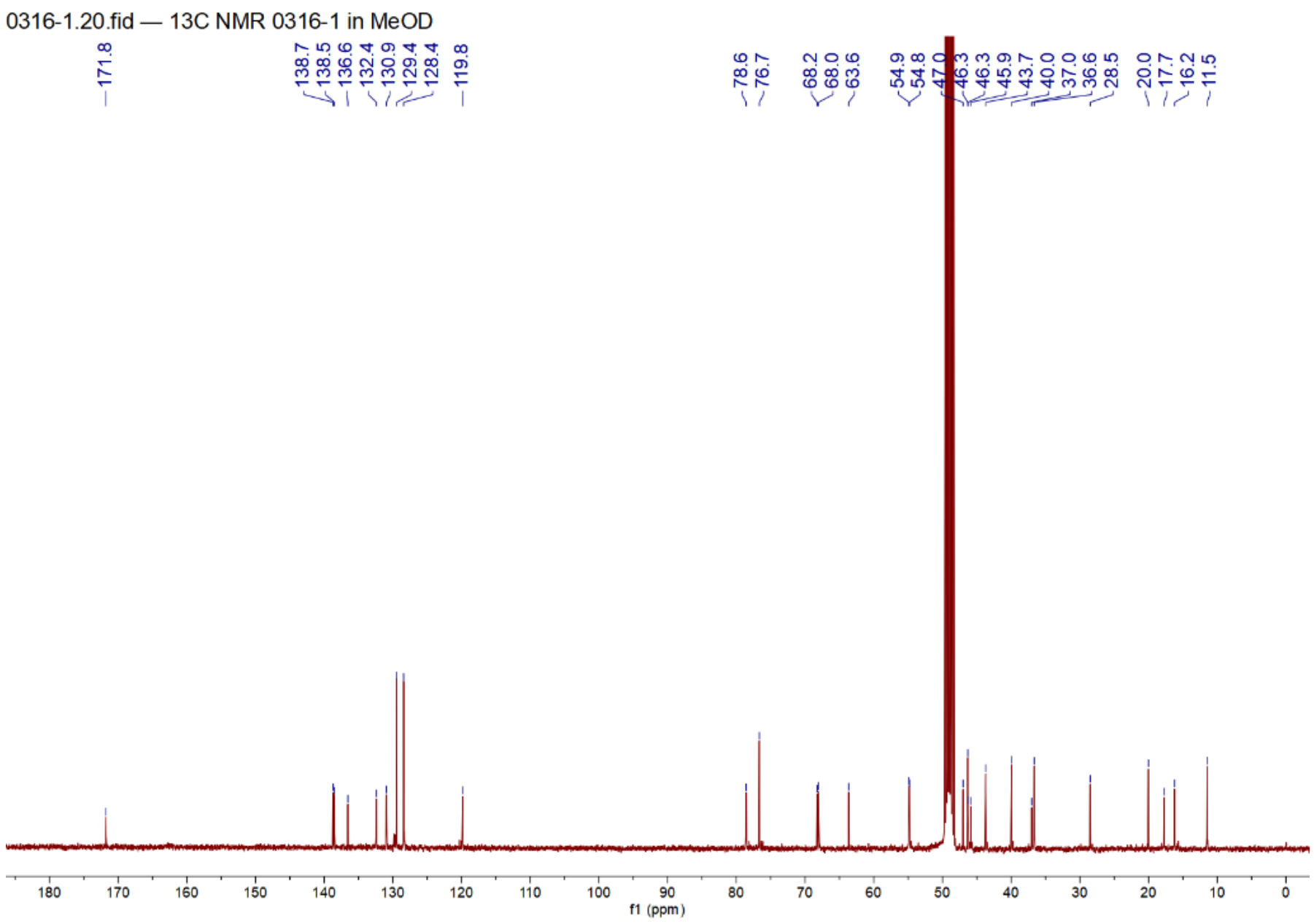

Figure S3.4 ^{13}C NMR spectrum of compound **3** (100 MHz, MeOD)

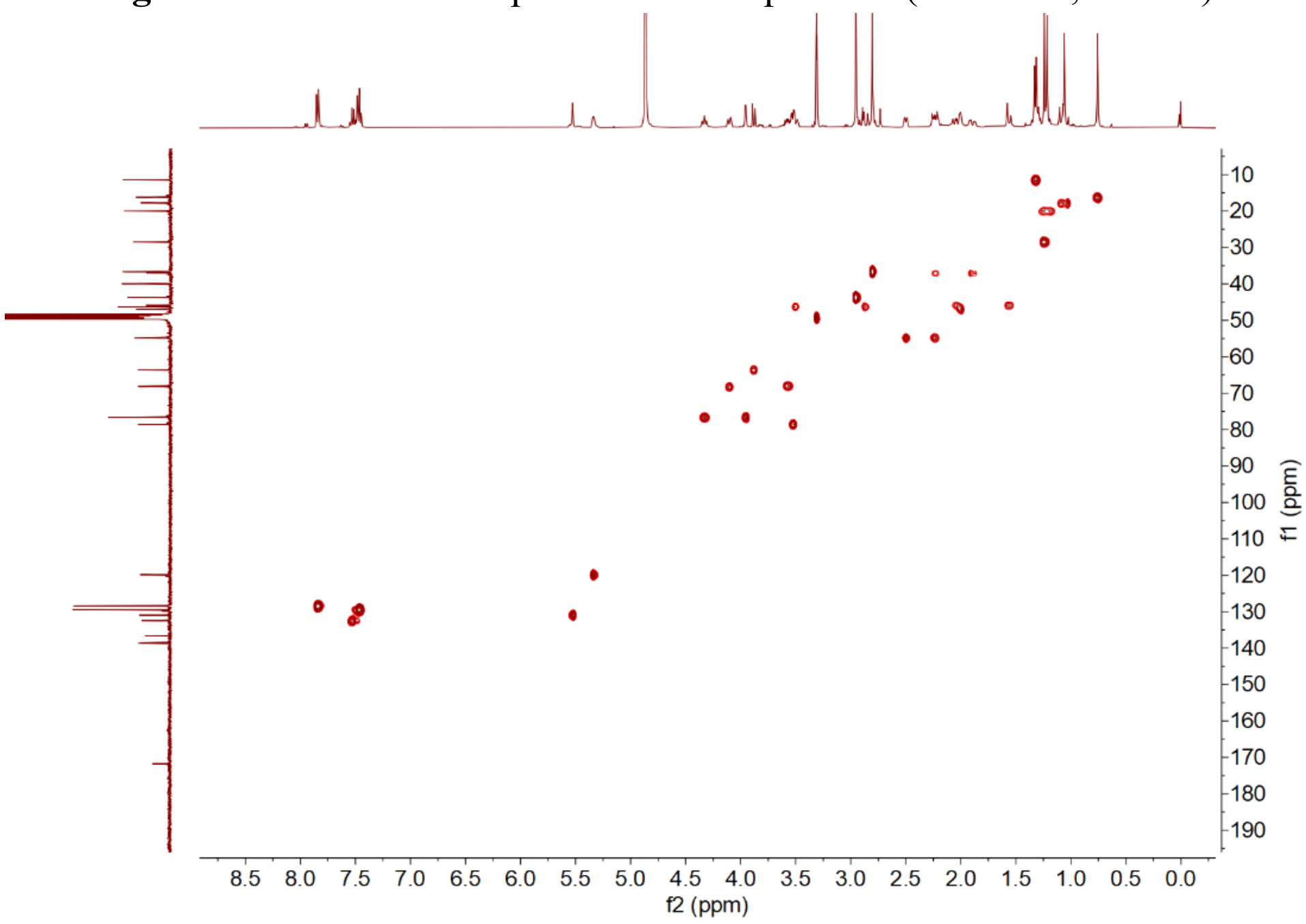

Figure S3.5 HSQC spectrum of compound **3**

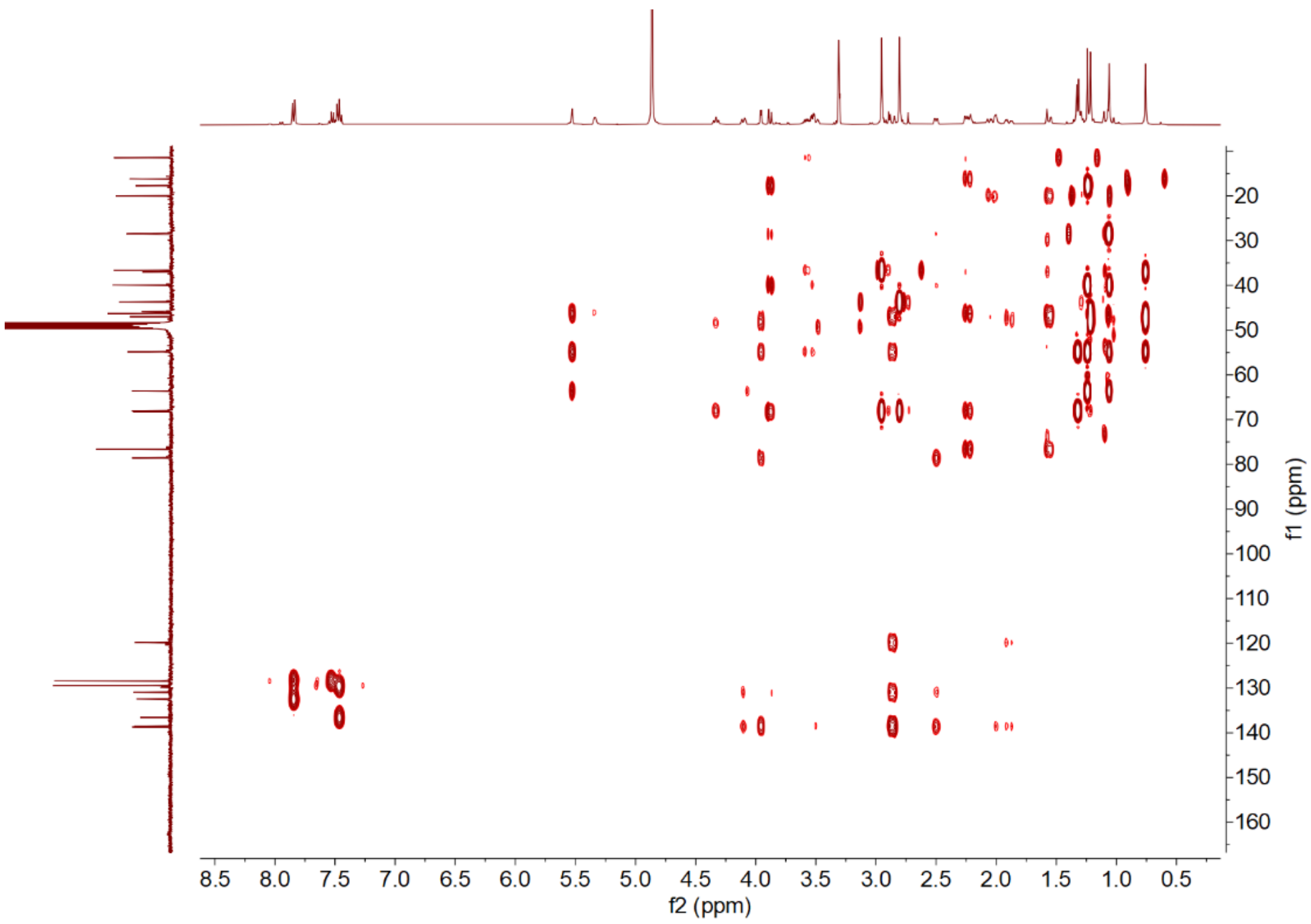

Figure S3.6 HMBC spectrum of compound **3**

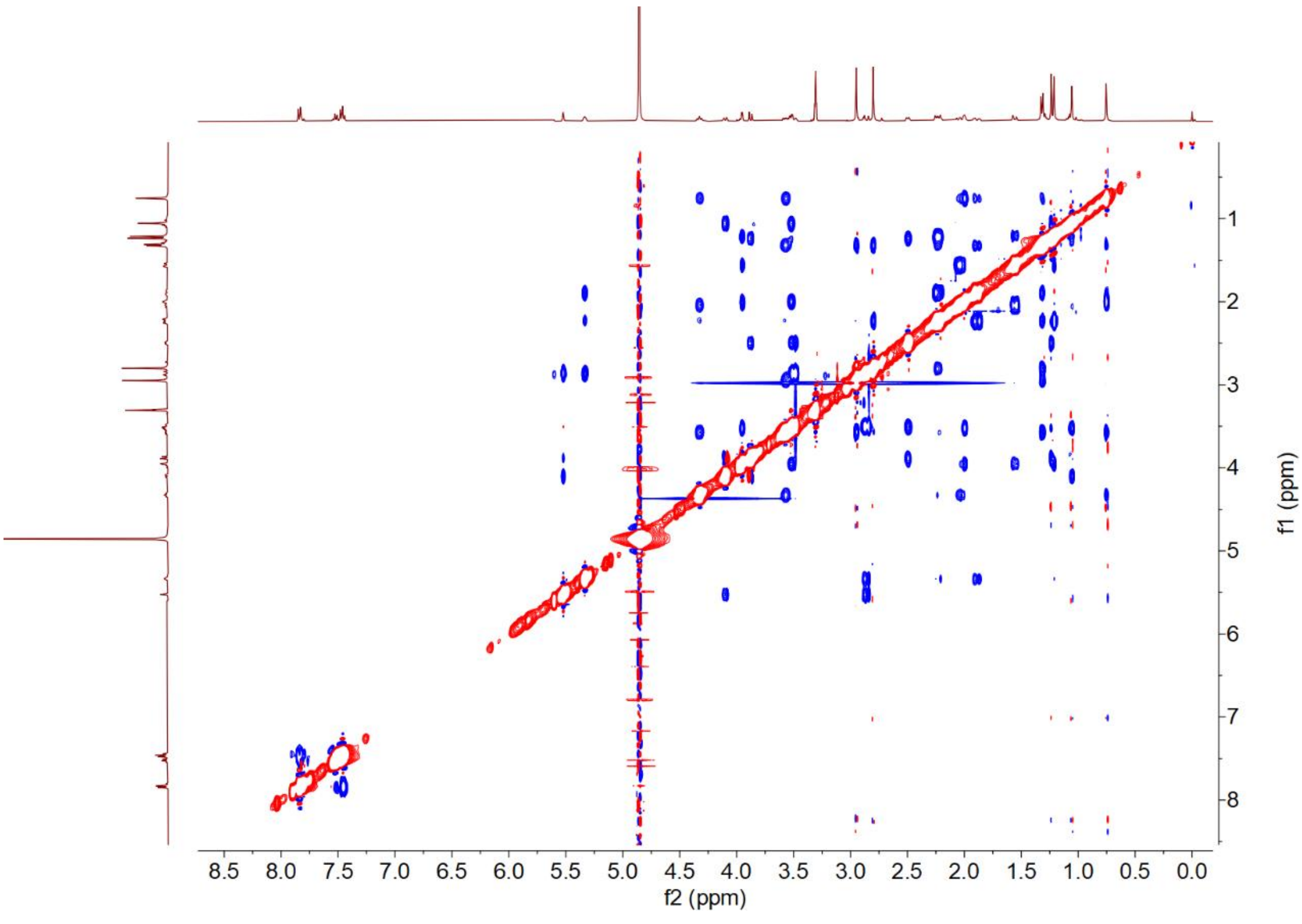

Figure S3.7 NOESY spectrum of compound **3**

Myribuxine D (**4**)

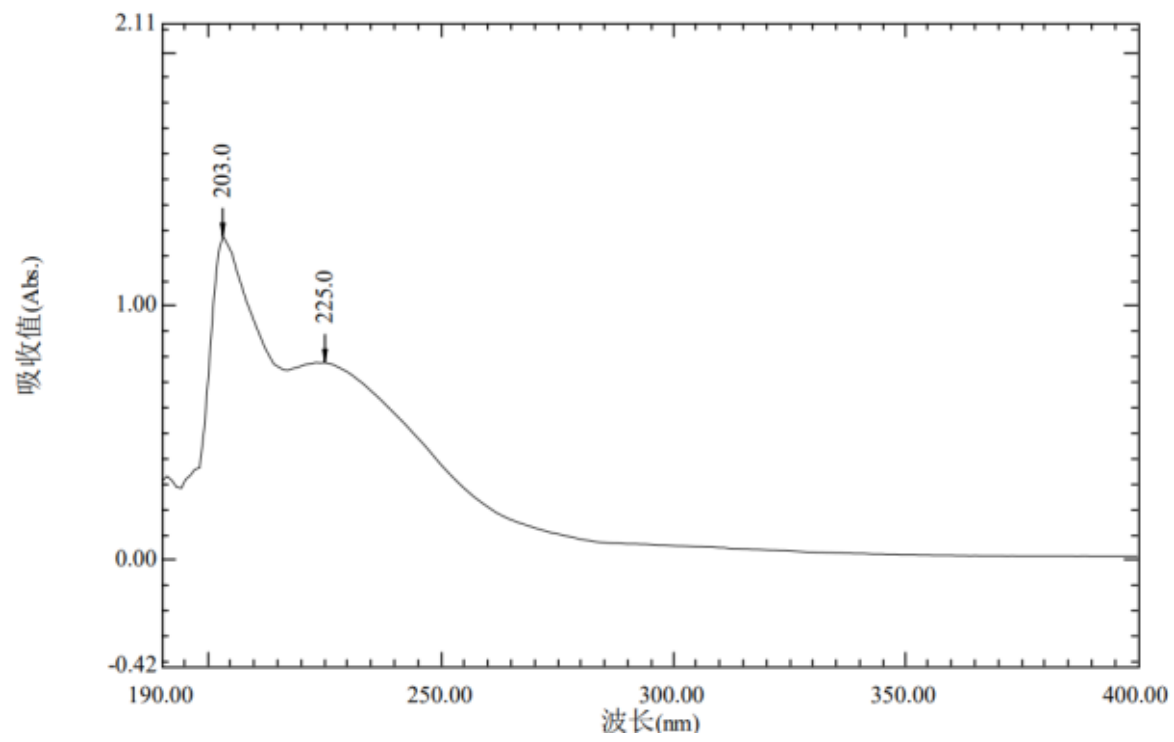

Figure S4.1 UV spectrum of compound **4**

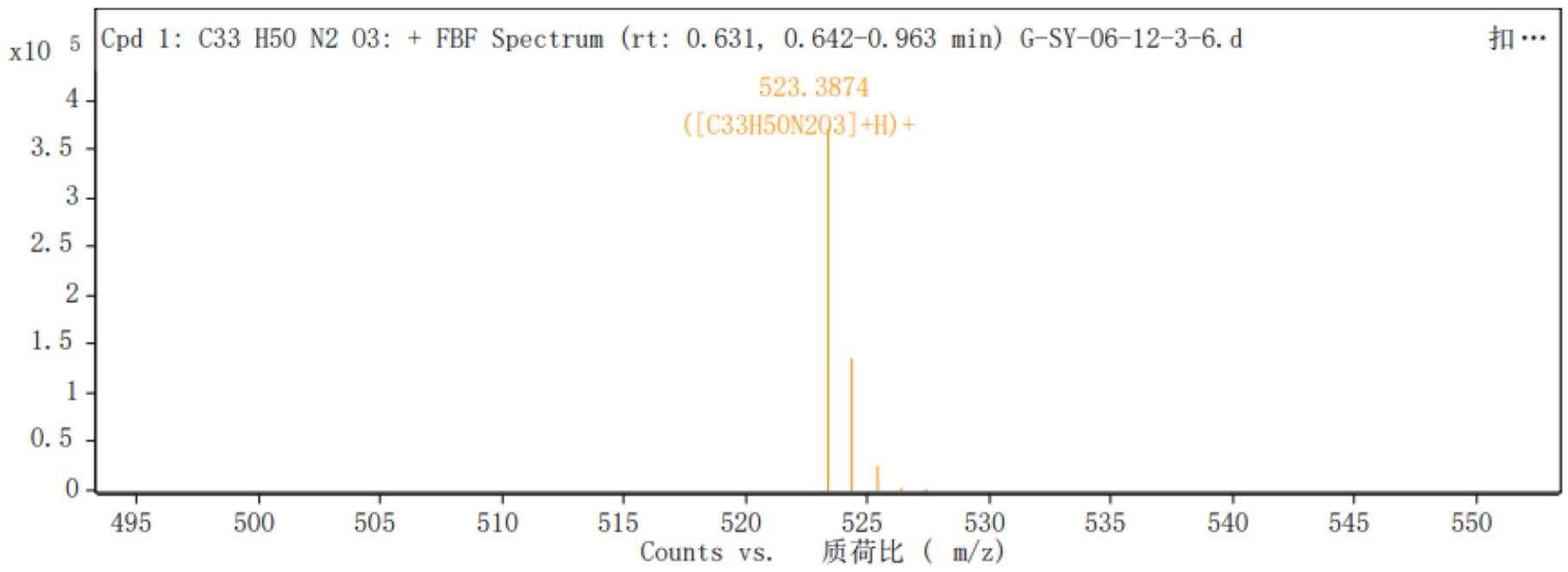

Figure S4.2 HR-ESI-MS spectrum of compound **4**

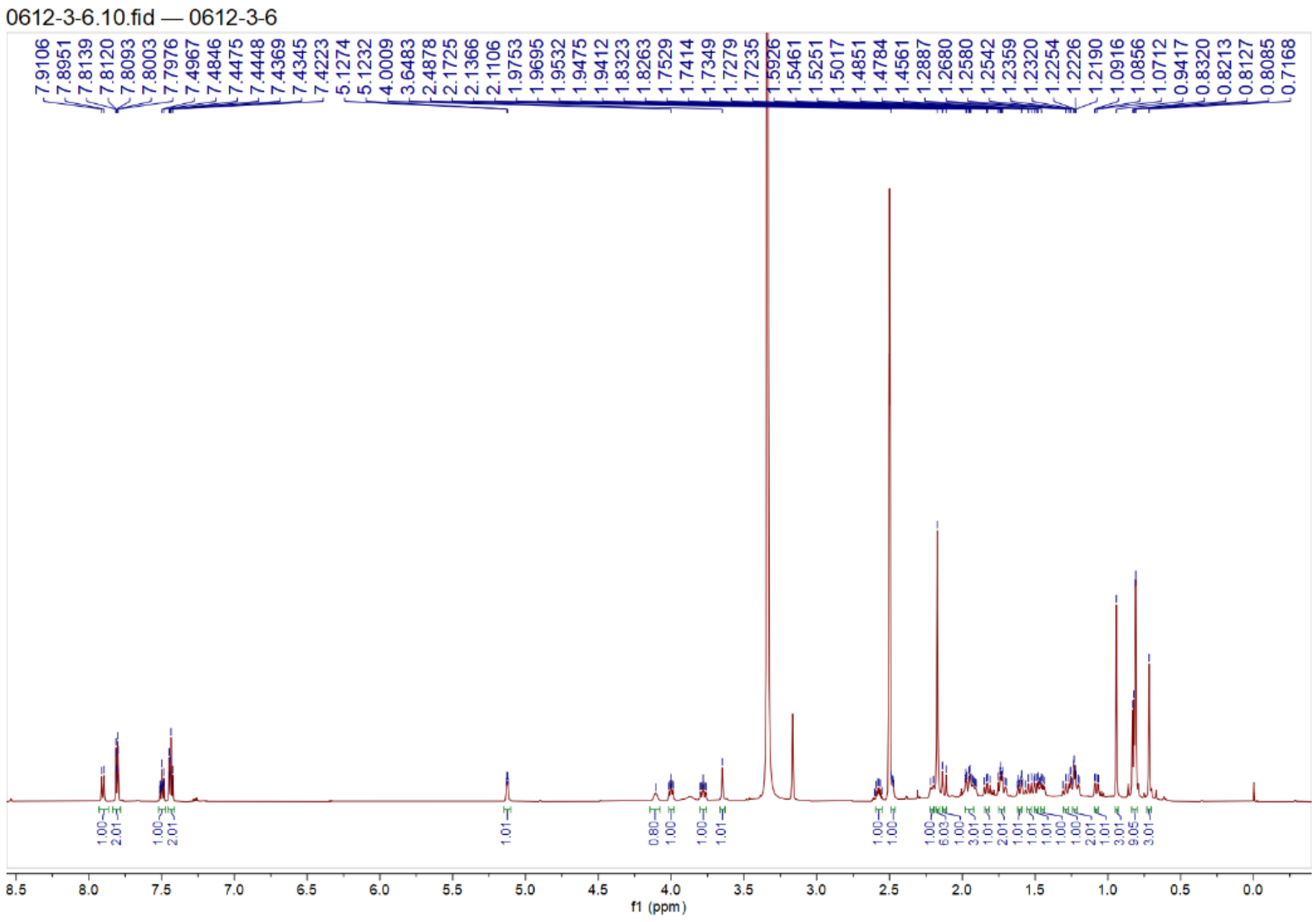

Figure S4.3 ^{1}H NMR spectrum of compound **4** (600 MHz, DMSO-d_6)

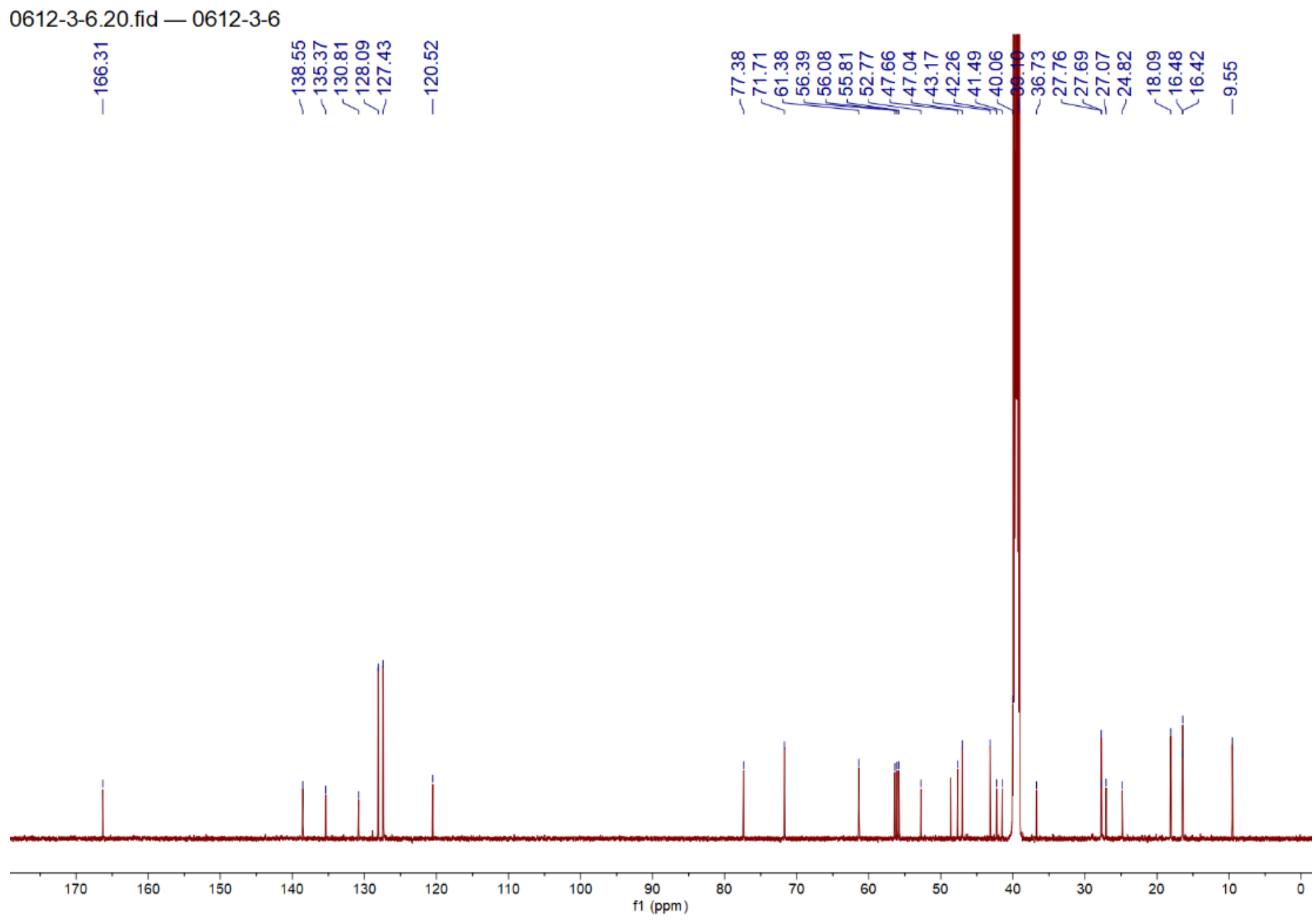

Figure S4.4 ^{13}C NMR spectrum of compound **4** (150 MHz, DMSO-d_6)

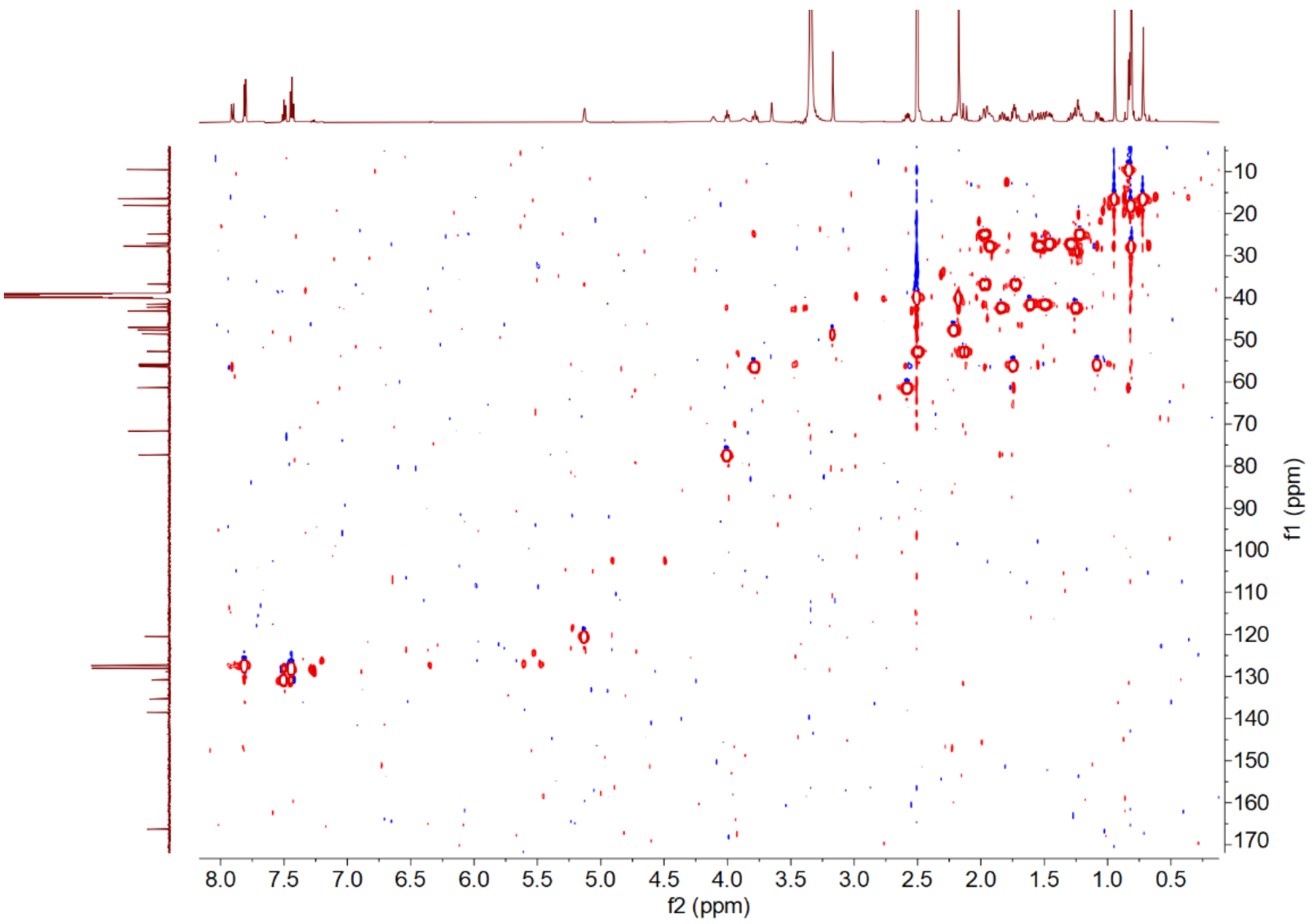

Figure S4.5 HSQC spectrum of compound **4**

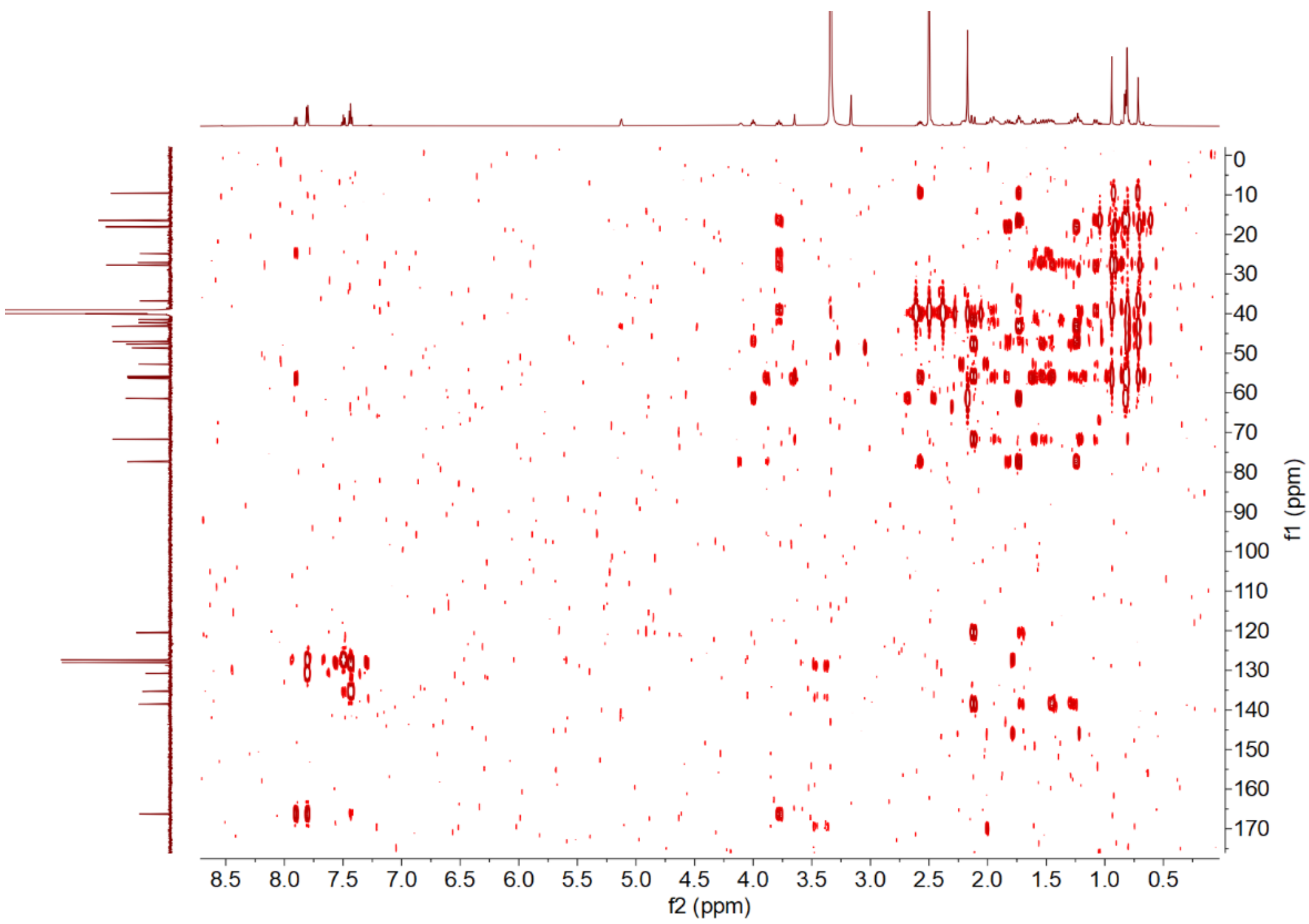

Figure S4.6 HMBC spectrum of compound **4**

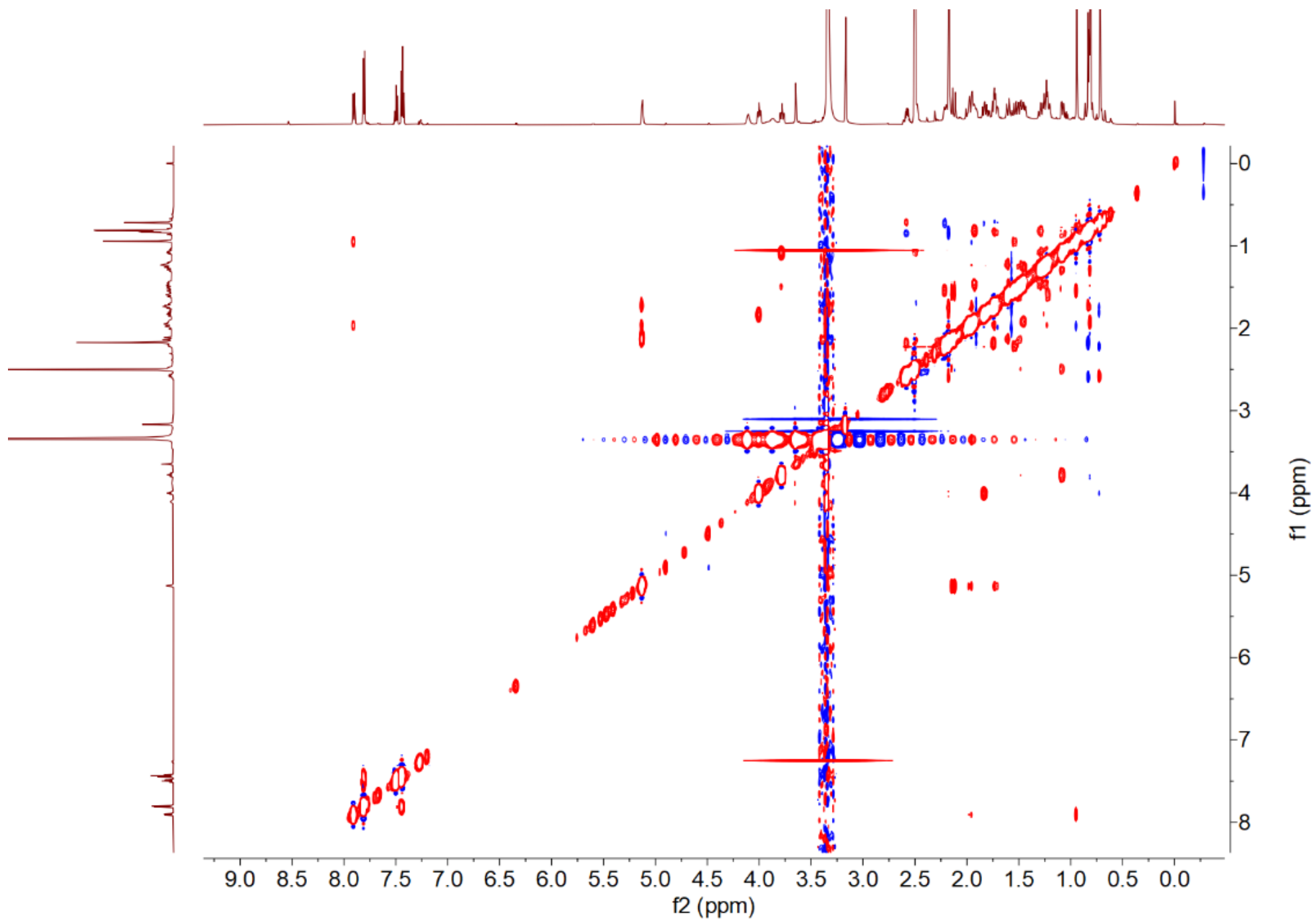

Figure S4.7 NOESY spectrum of compound **4**

Myribuxine E (**5**)

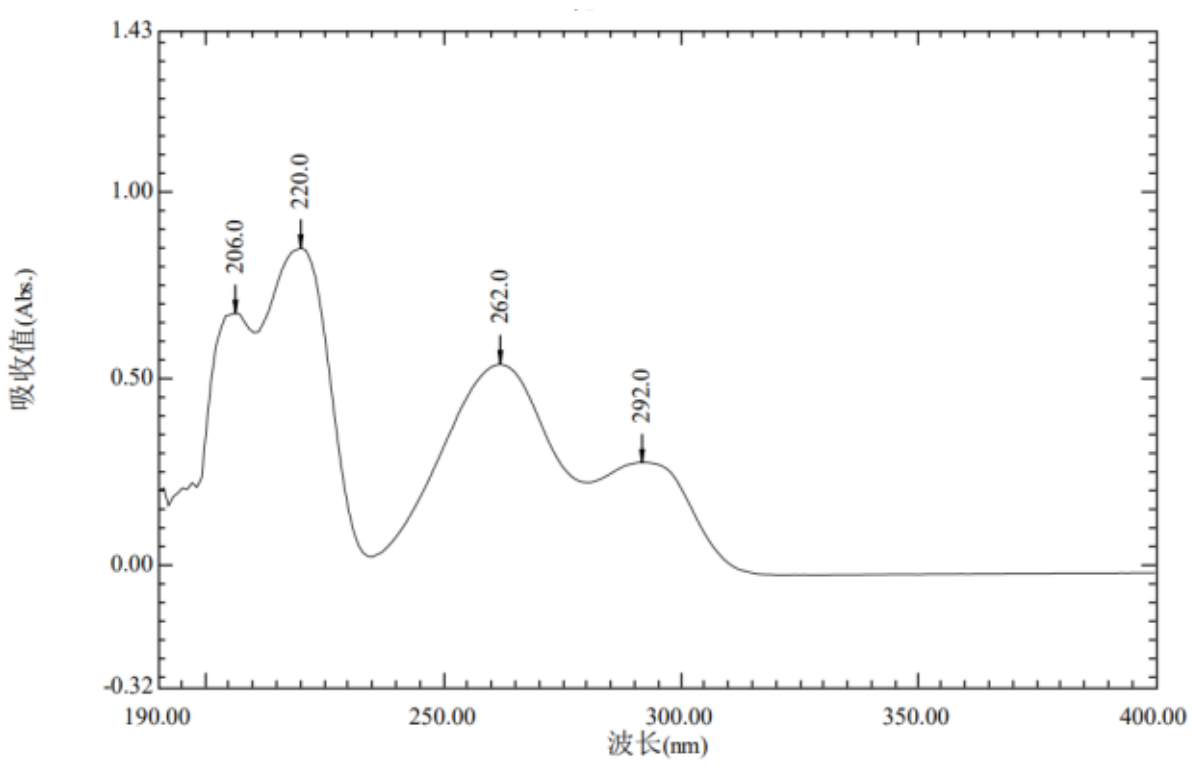

Figure S5.1 UV spectrum of compound **5**

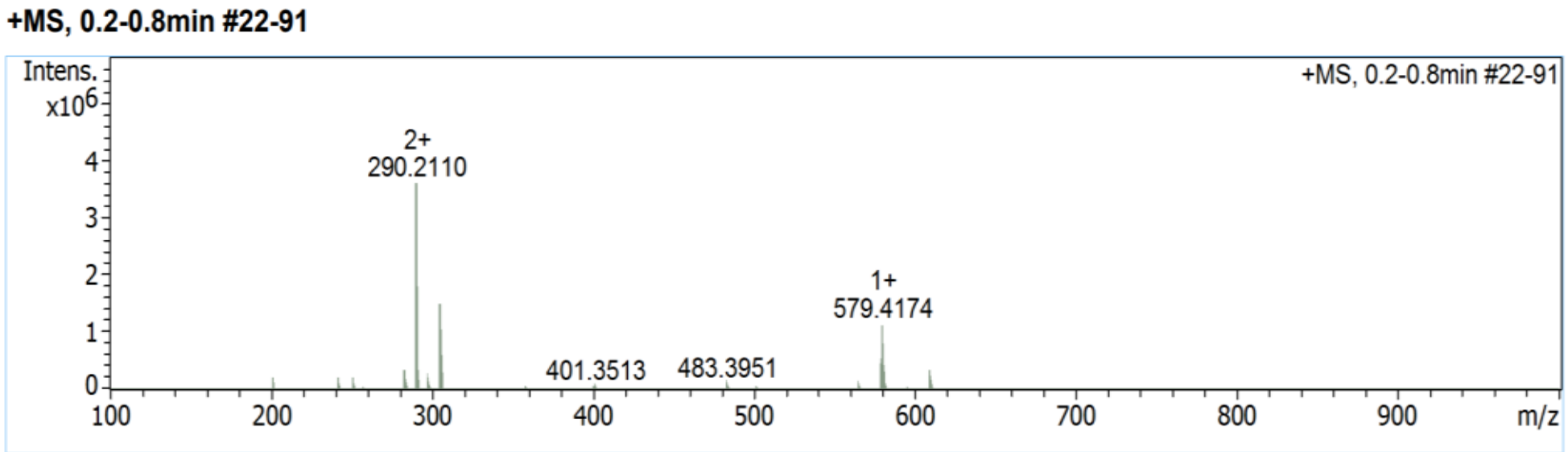

Figure S5.2 HR-ESI-MS spectrum of compound **5**

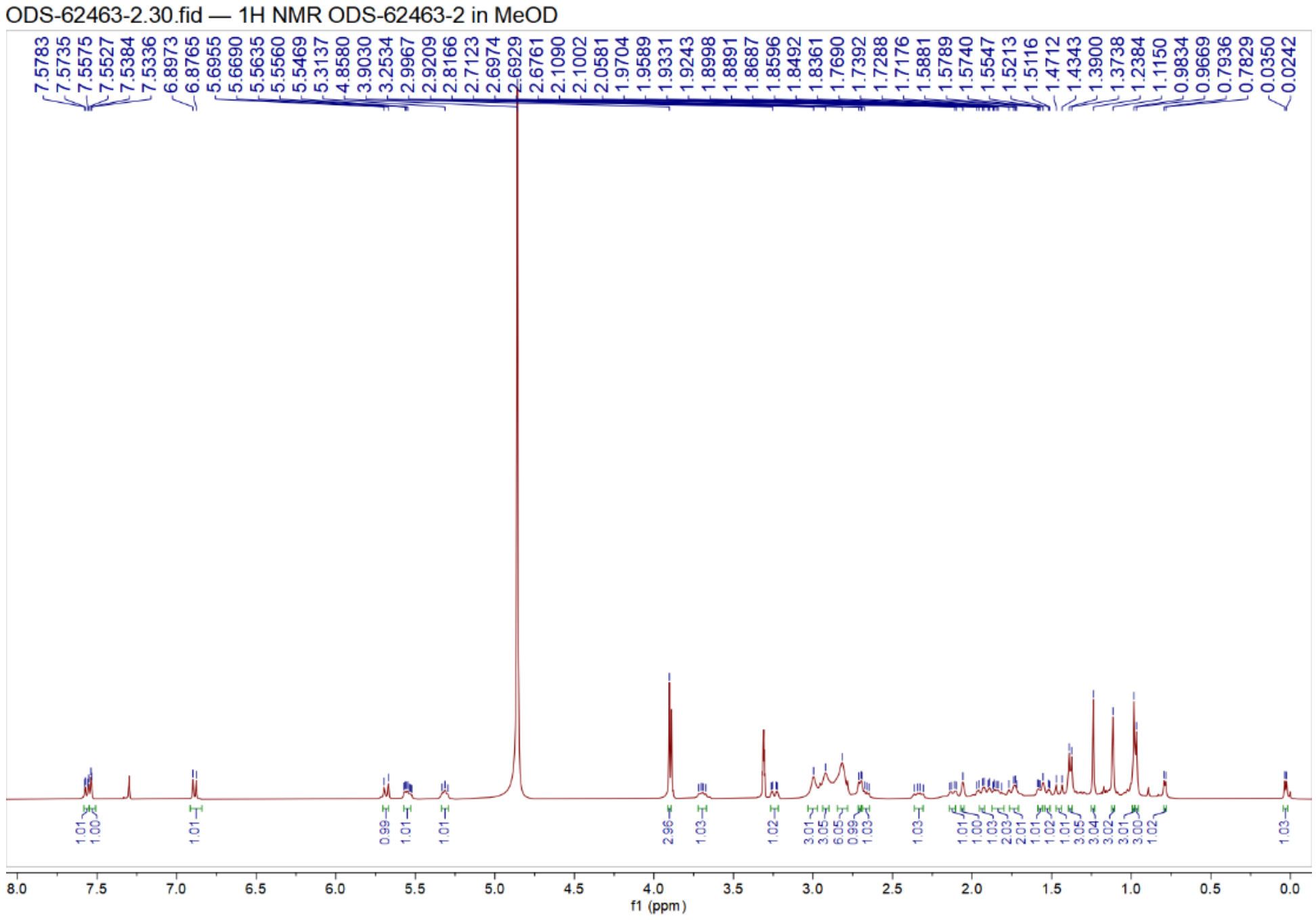

Figure S5.3 ^{1}H NMR spectrum of compound **5** (400 MHz, MeOD)

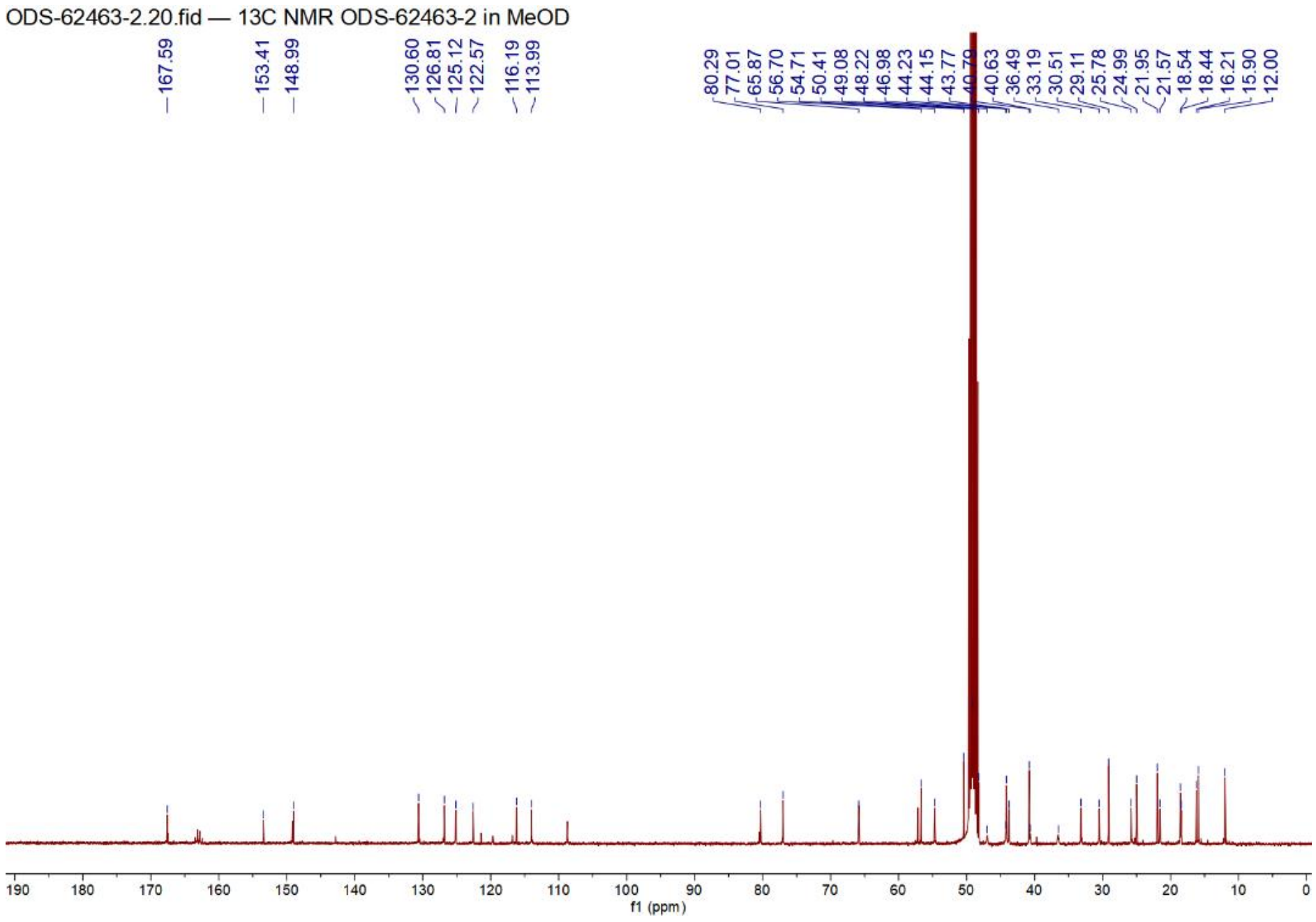

Figure S5.4 ^{13}C NMR spectrum of compound **5** (100 MHz, MeOD)

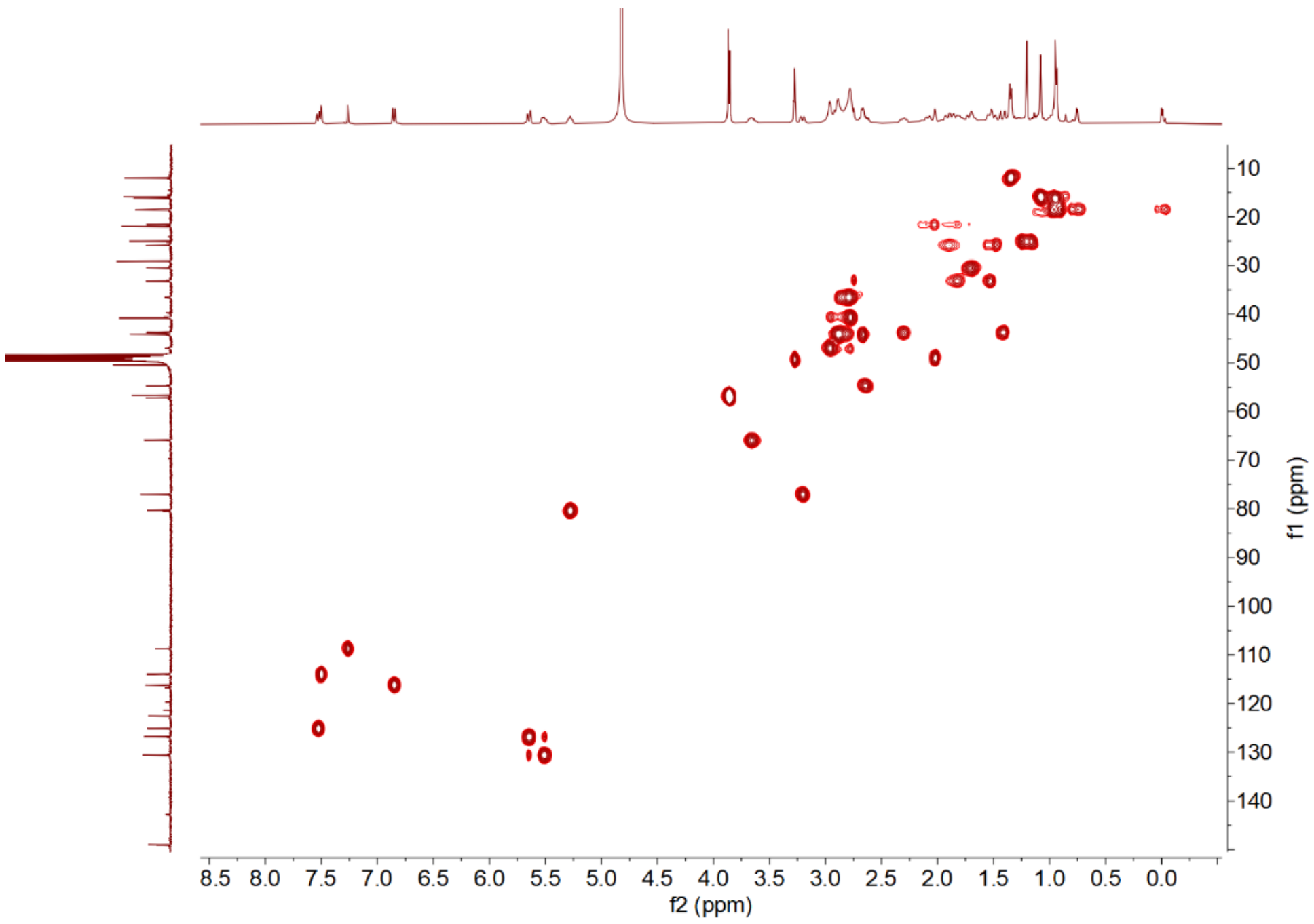

Figure S5.5 HSQC spectrum of compound **5**

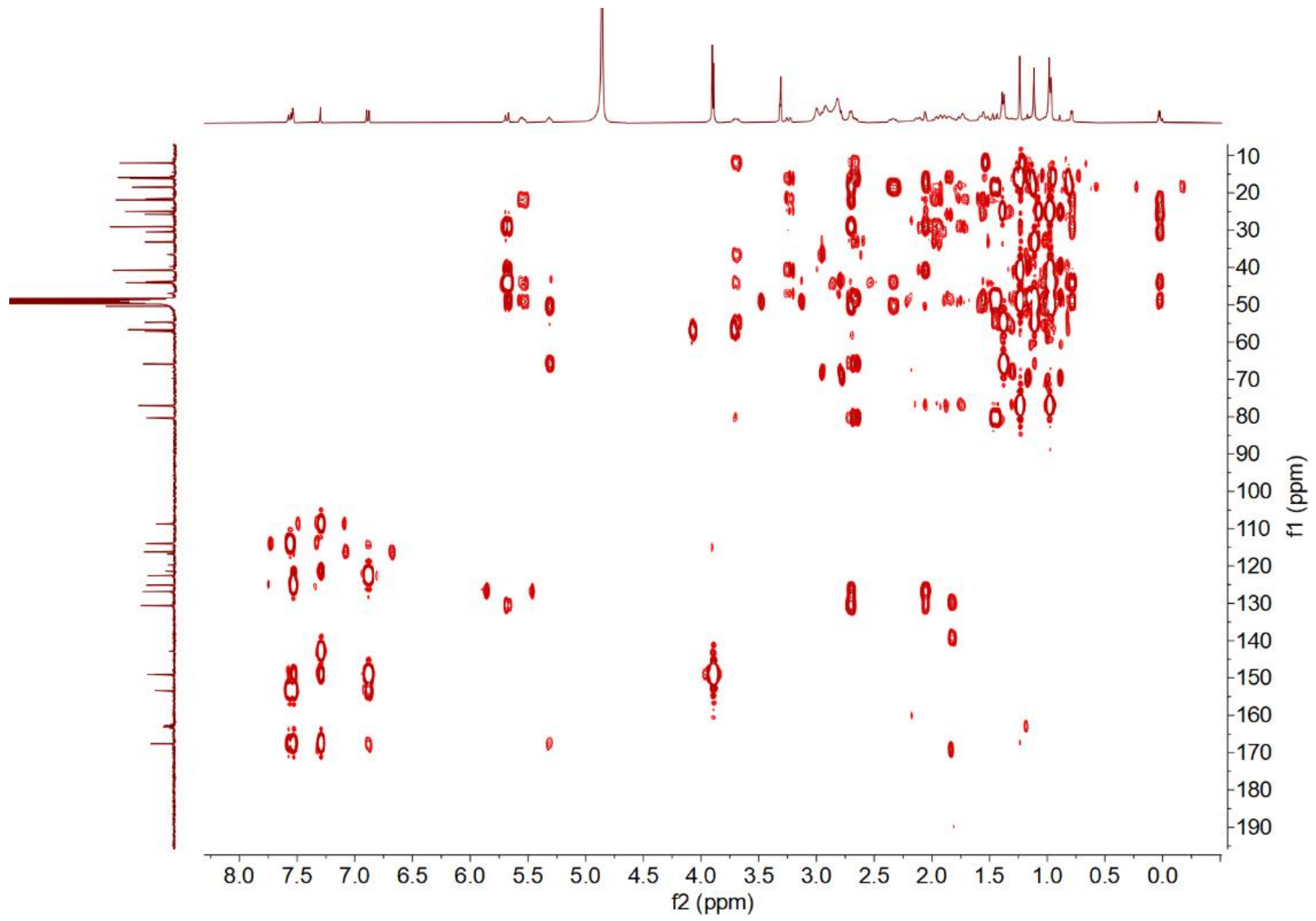

Figure S5.6 HMBC spectrum of compound **5**

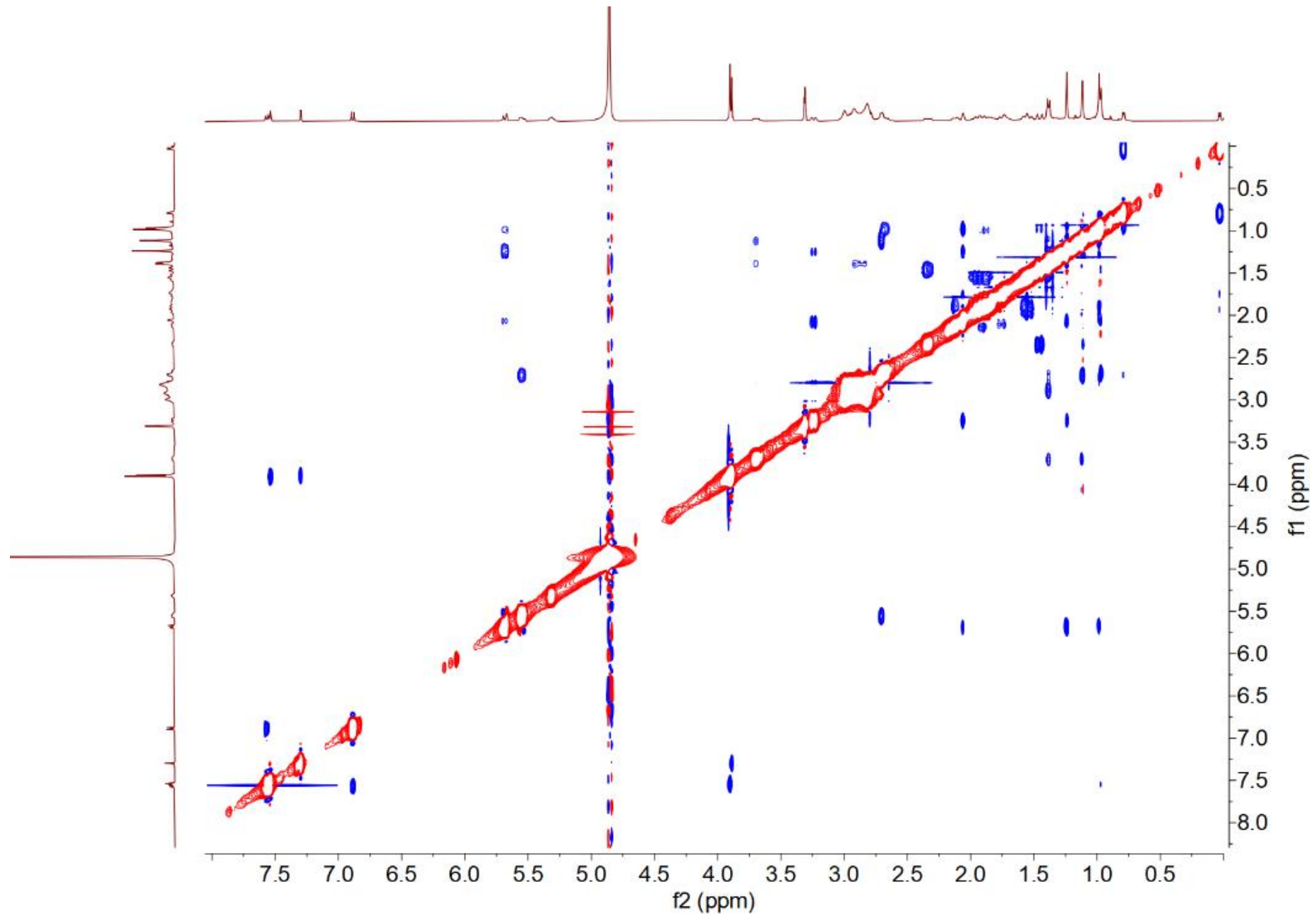

Figure S5.7 NOESY spectrum of compound **5**

Myribuxine F (**6**)

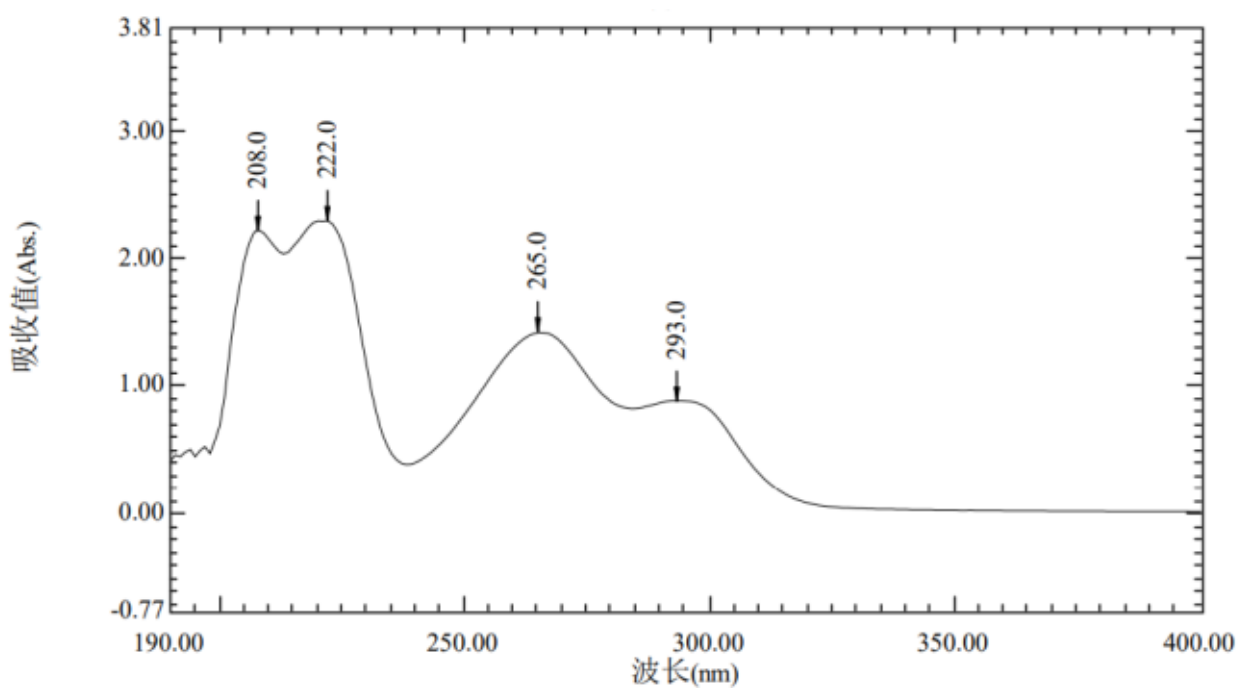

Figure S6.1 UV spectrum of compound **6**

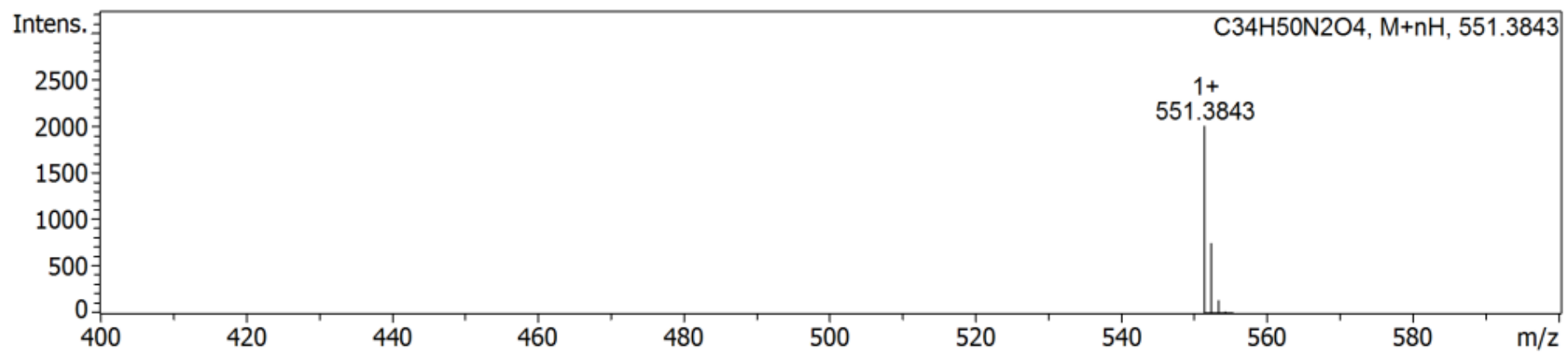

Figure S6.2 HR-ESI-MS spectrum of compound **6**

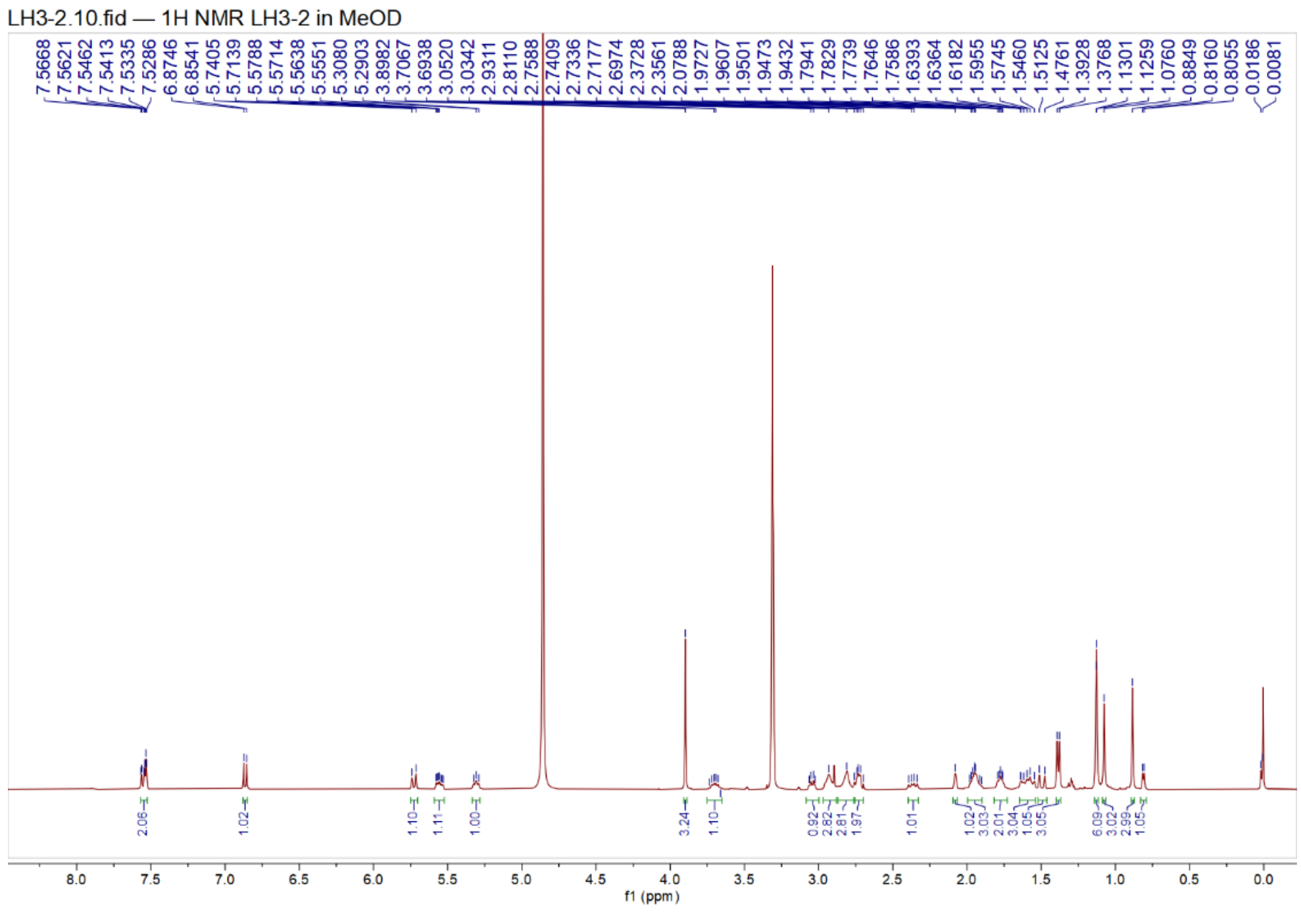

Figure S6.3 ^{1}H NMR spectrum of compound **6** (400 MHz, MeOD)

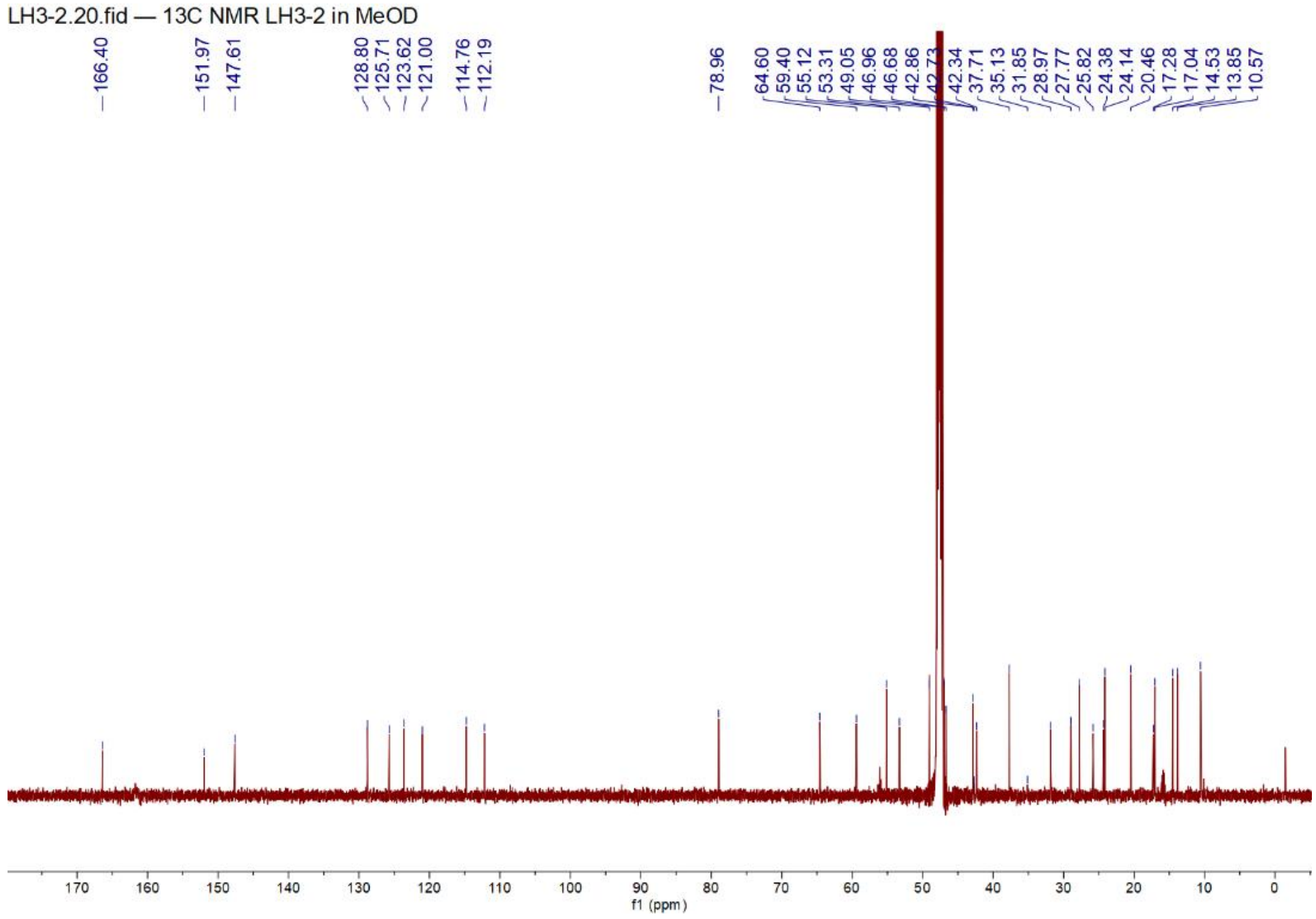

Figure S6.4 ^{13}C NMR spectrum of compound **6** (100 MHz, MeOD)

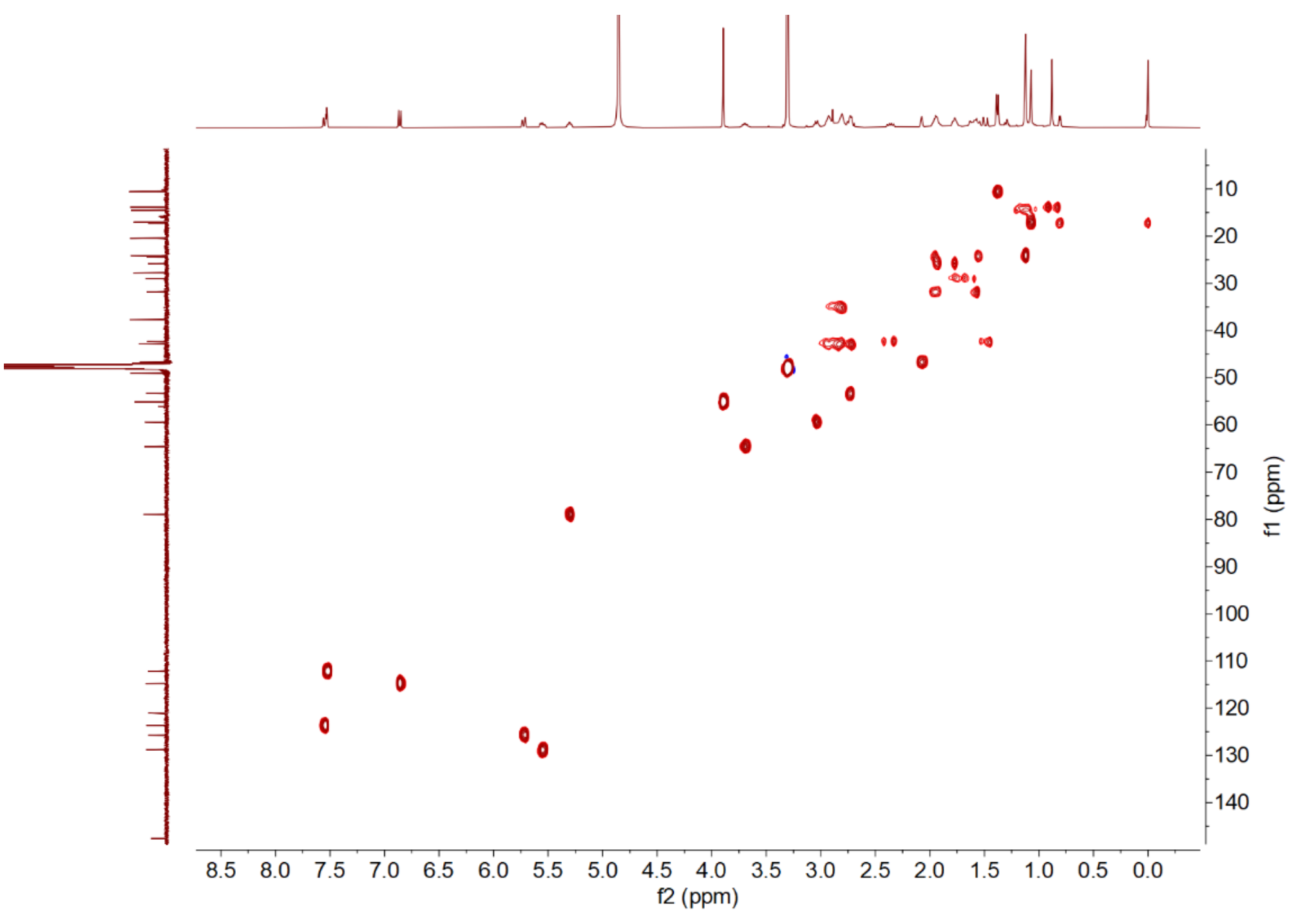

Figure S6.5 HSQC spectrum of compound **6**

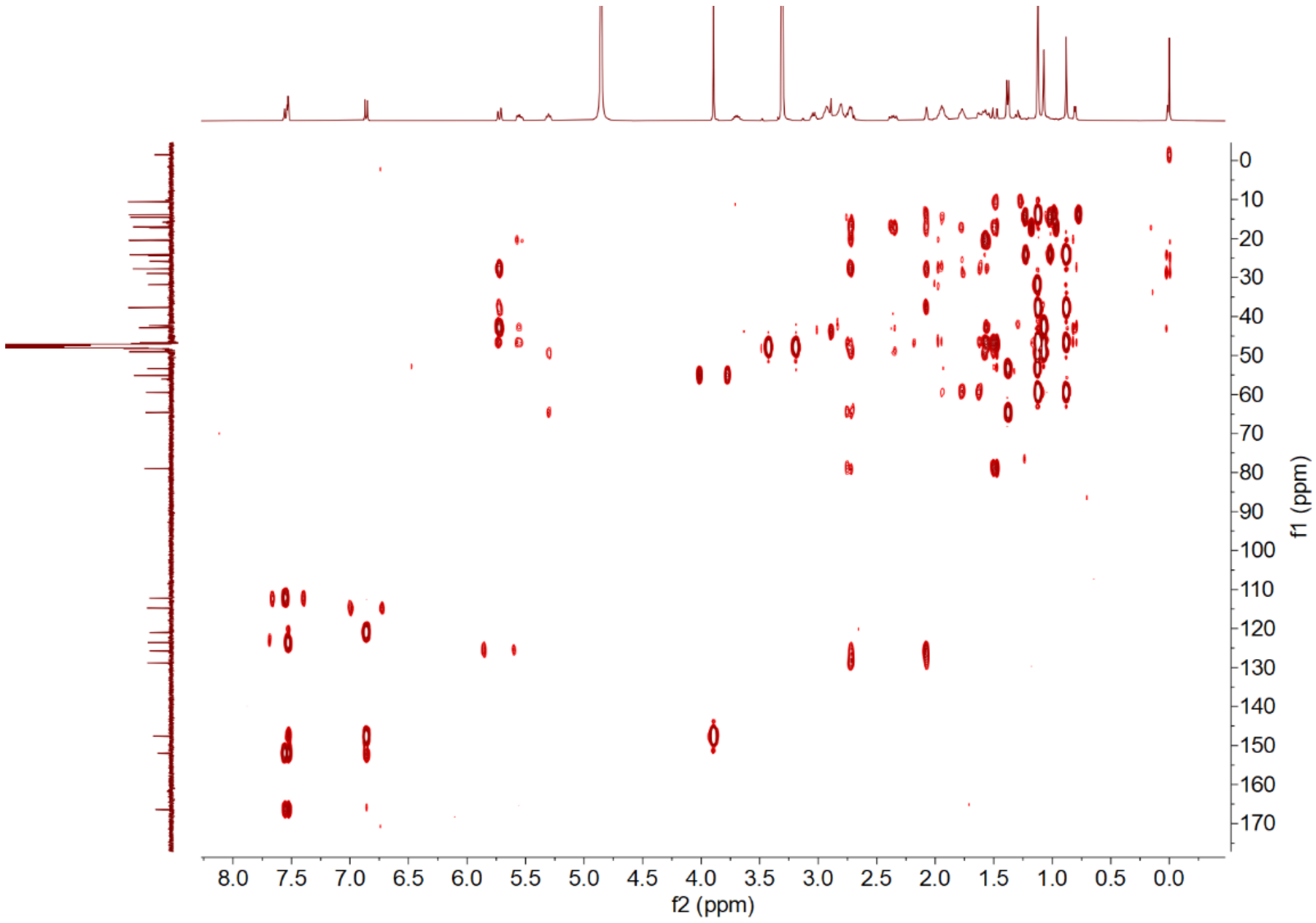

Figure S6.6 HMBC spectrum of compound **6**

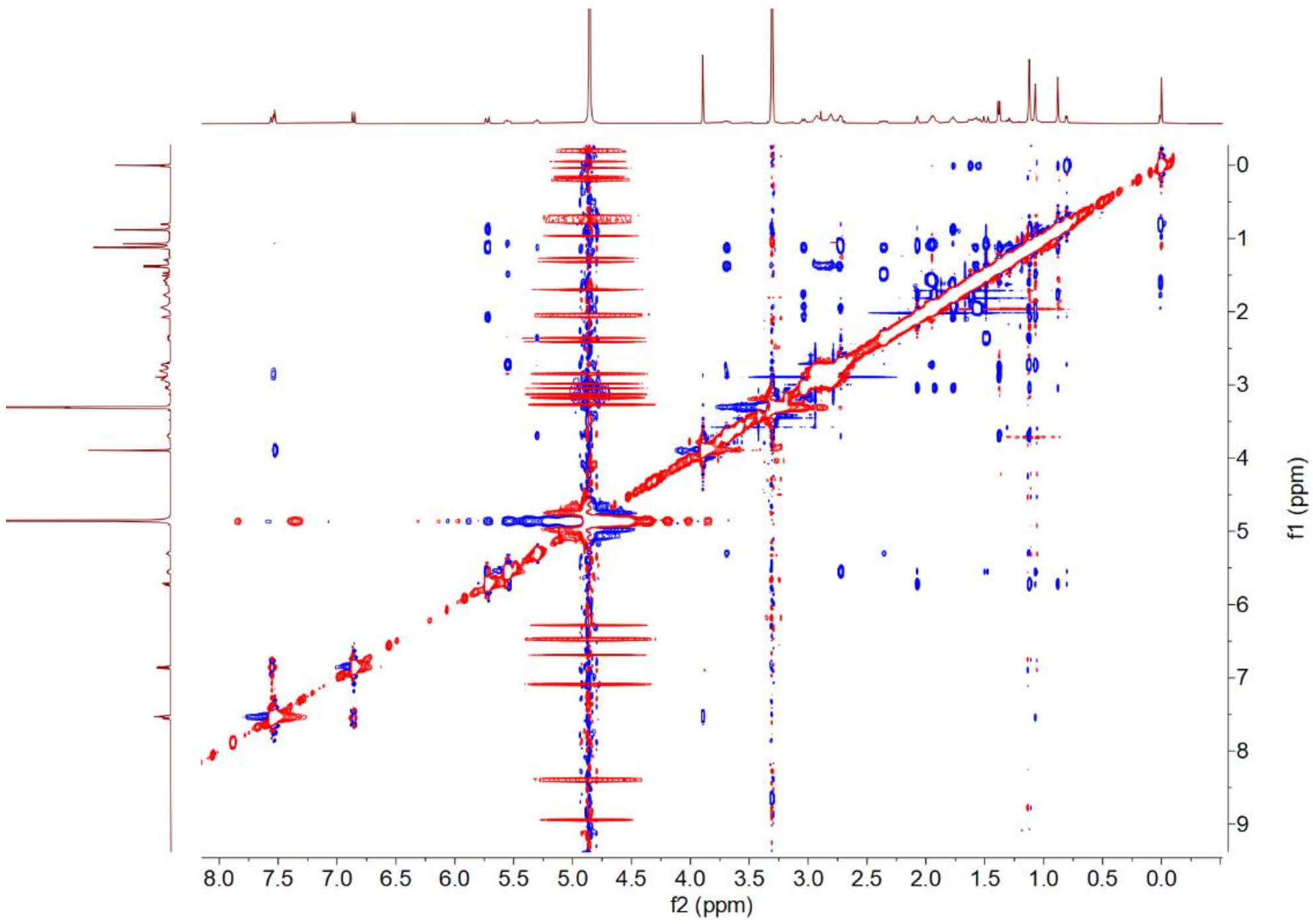

Figure S6.7 NOESY spectrum of compound **6**

Myribuxine G (**7**)

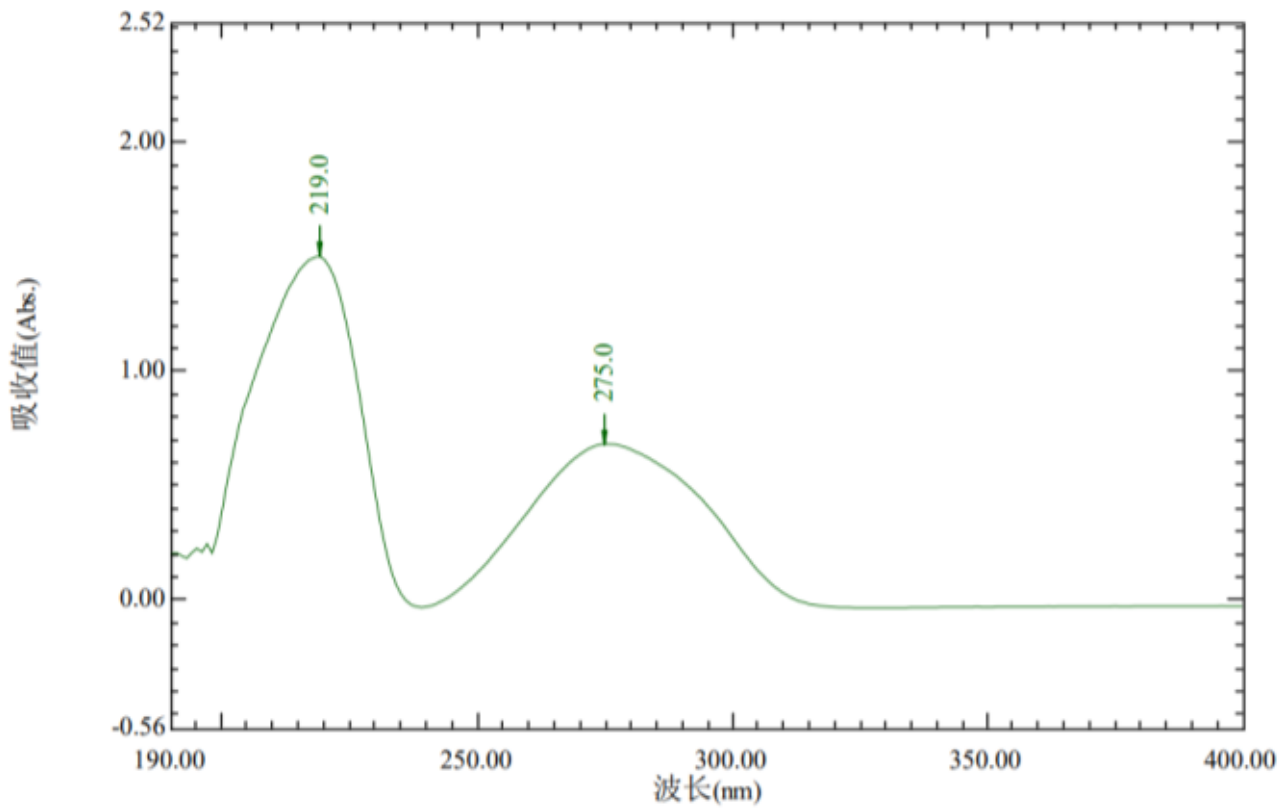

Figure S7.1 UV spectrum of compound **7**

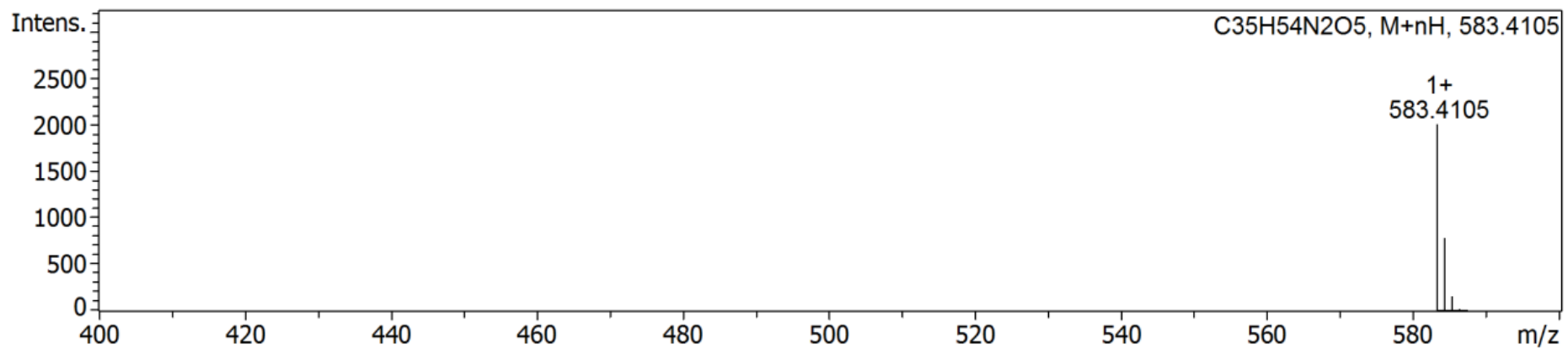

Figure S7.2 HR-ESI-MS spectrum of compound **7**

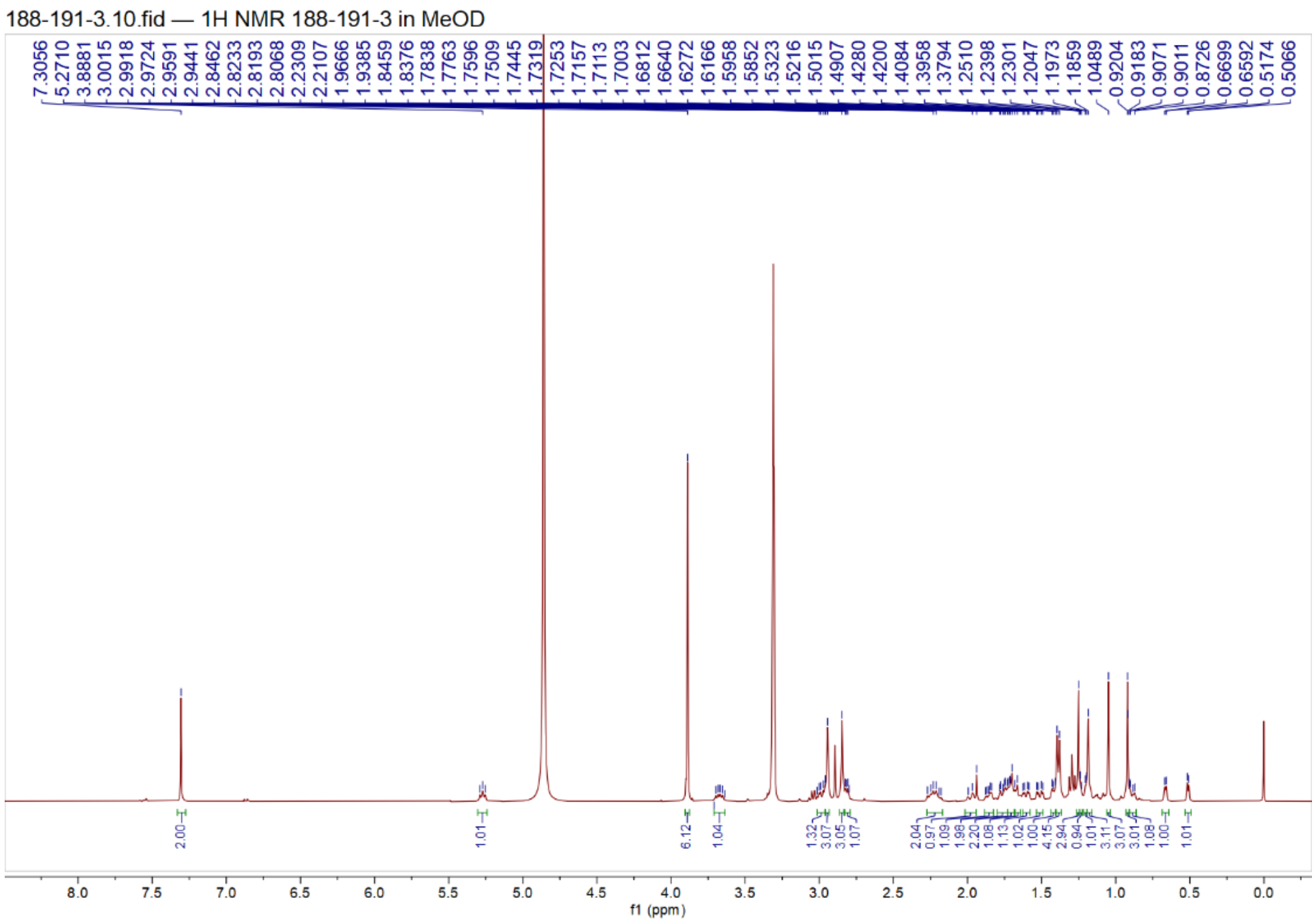

Figure S7.3 ^{1}H NMR spectrum of compound **7** (600 MHz, MeOD)

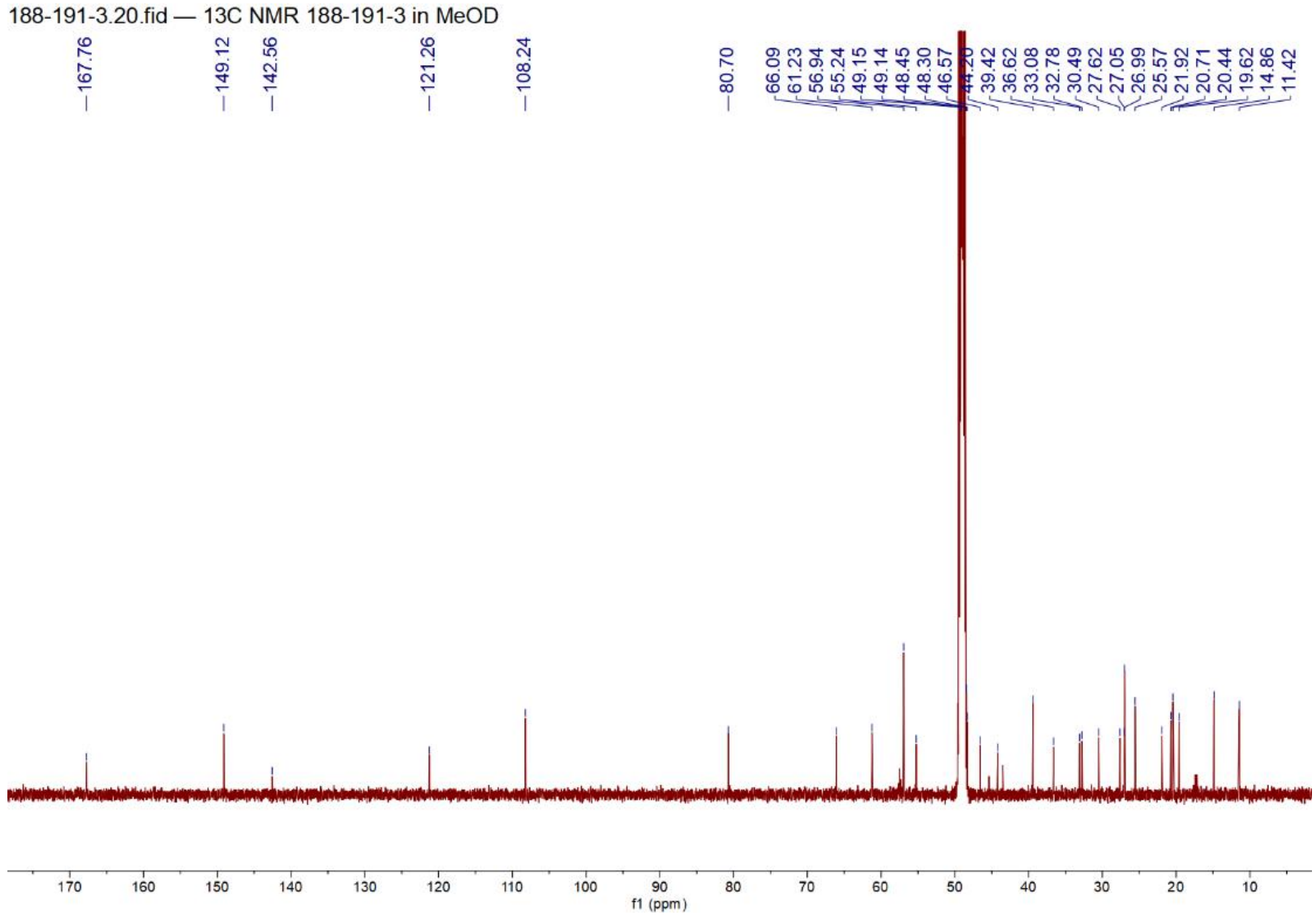

Figure S7.4 ^{13}C NMR spectrum of compound **7** (150 MHz, MeOD)

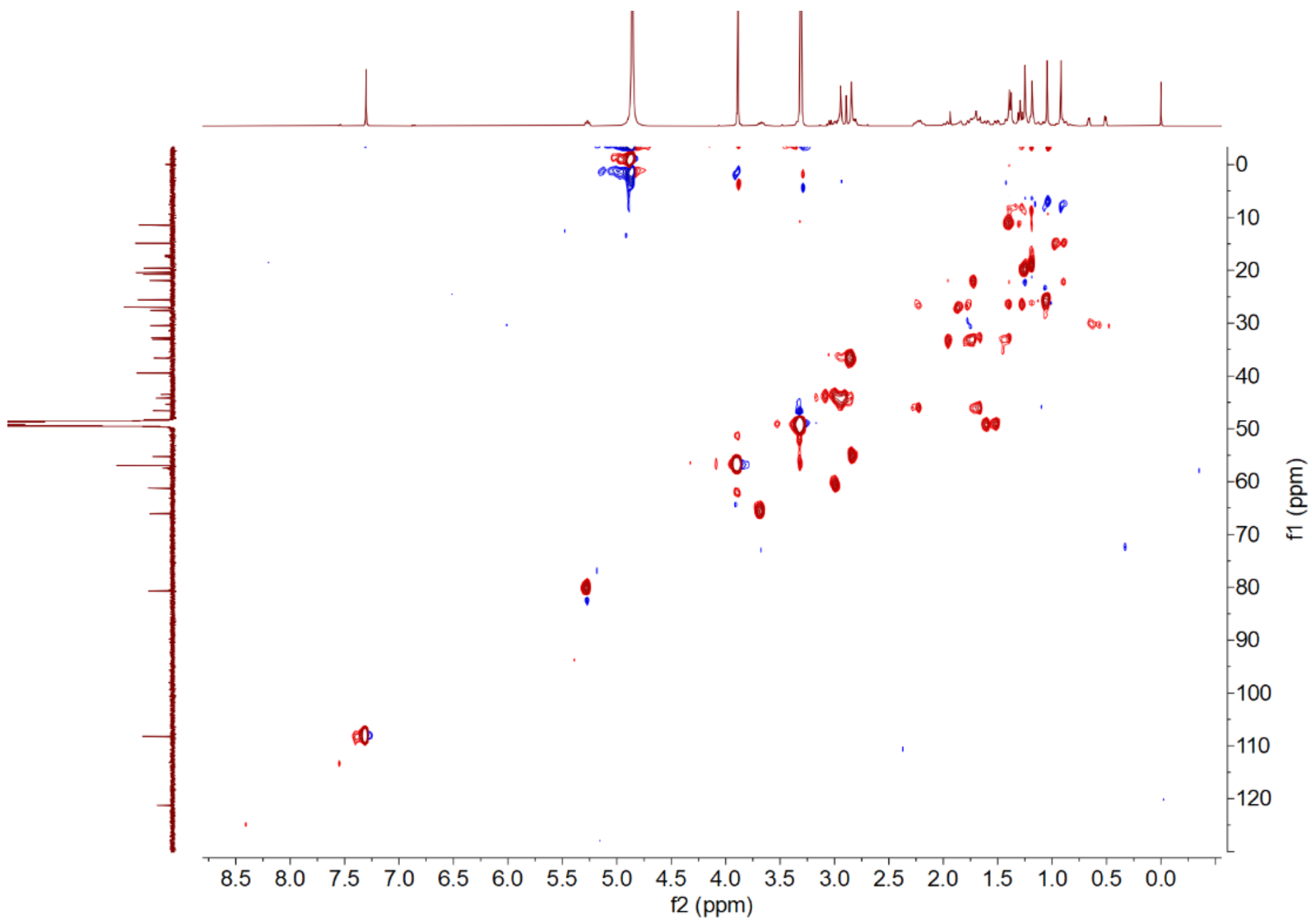

Figure S7.5 HSQC spectrum of compound **7**

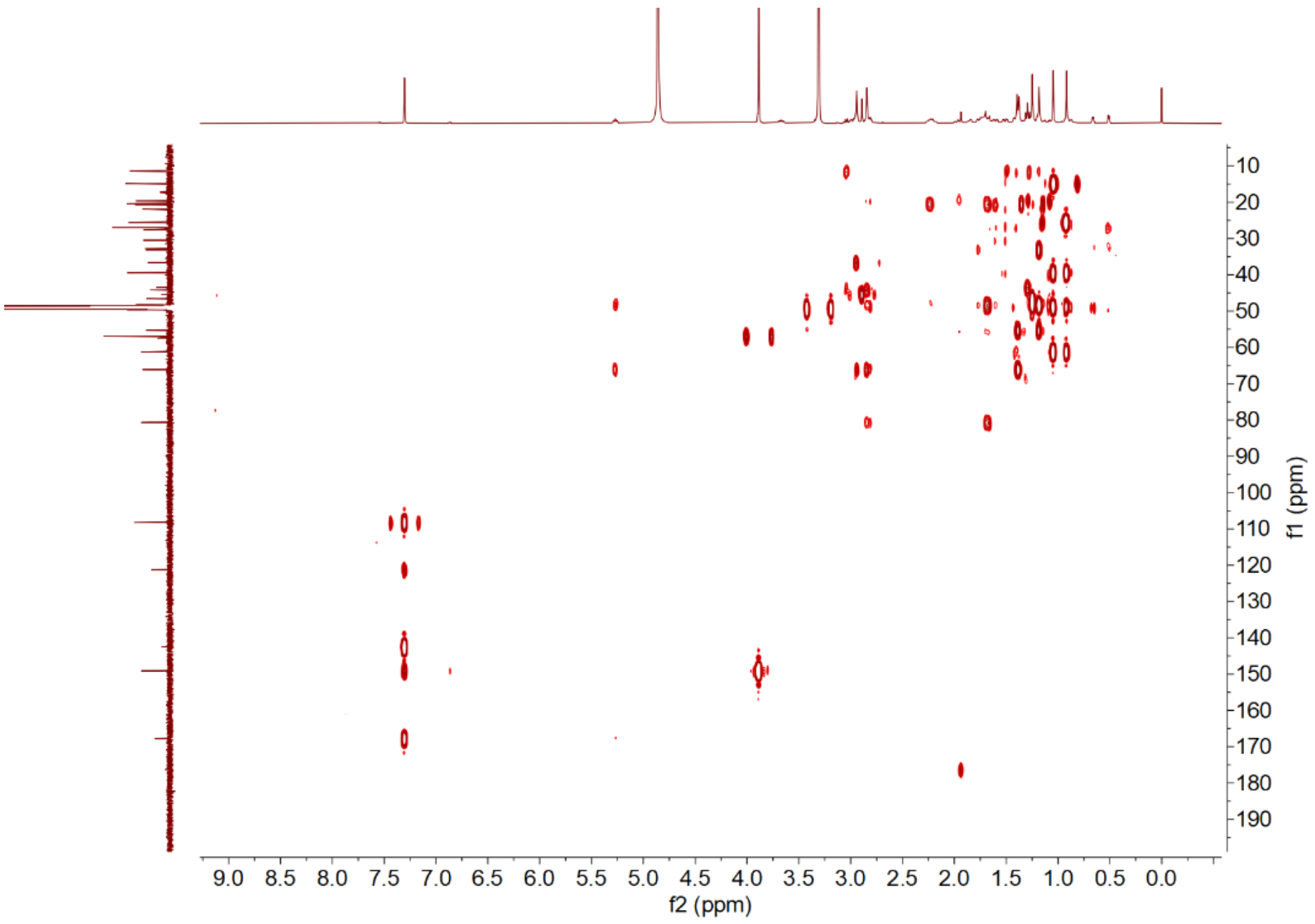

Figure S7.6 HMBC spectrum of compound **7**

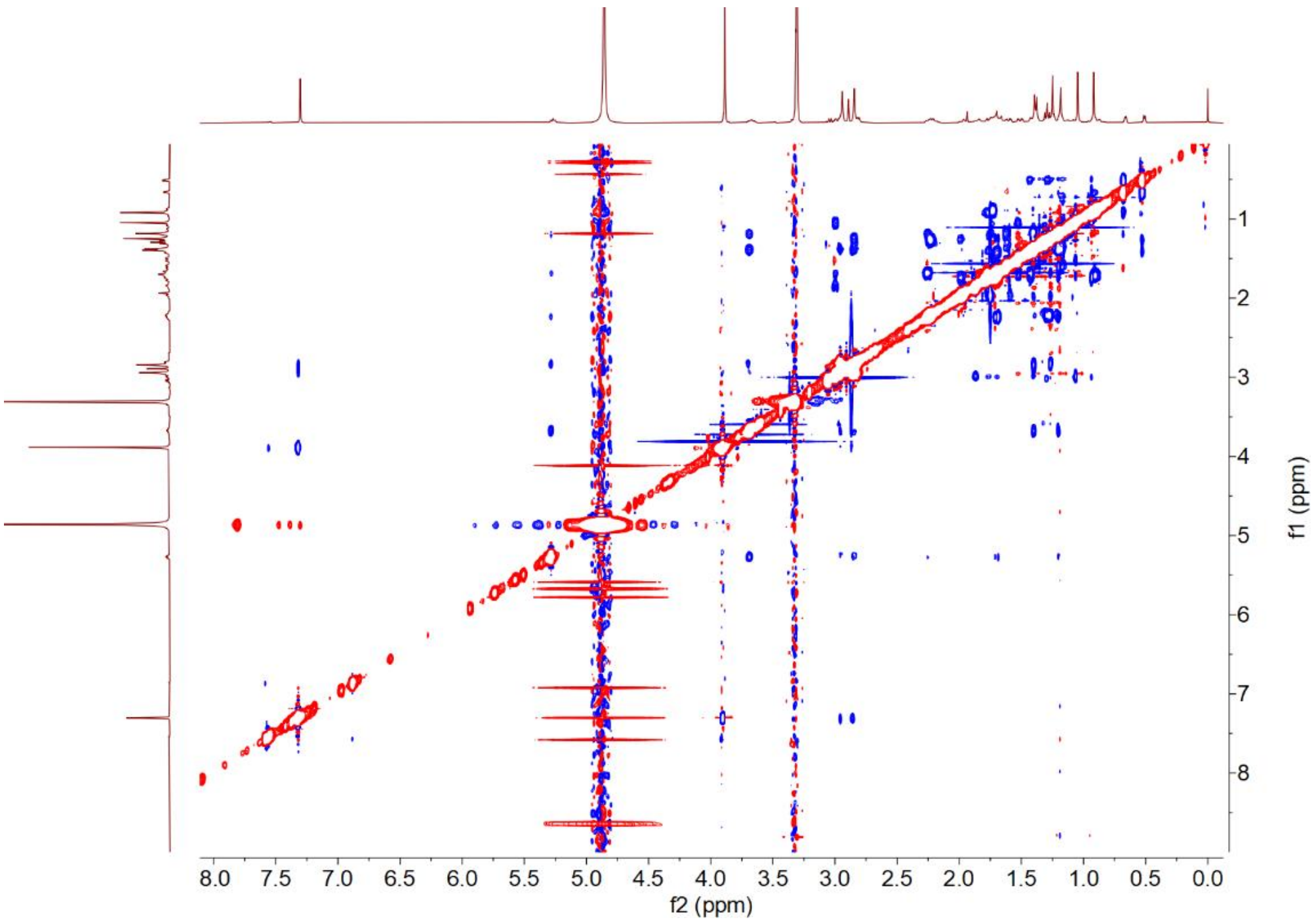

Figure S7.7 NOESY spectrum of compound **7**

Myribuxine H (**8**)

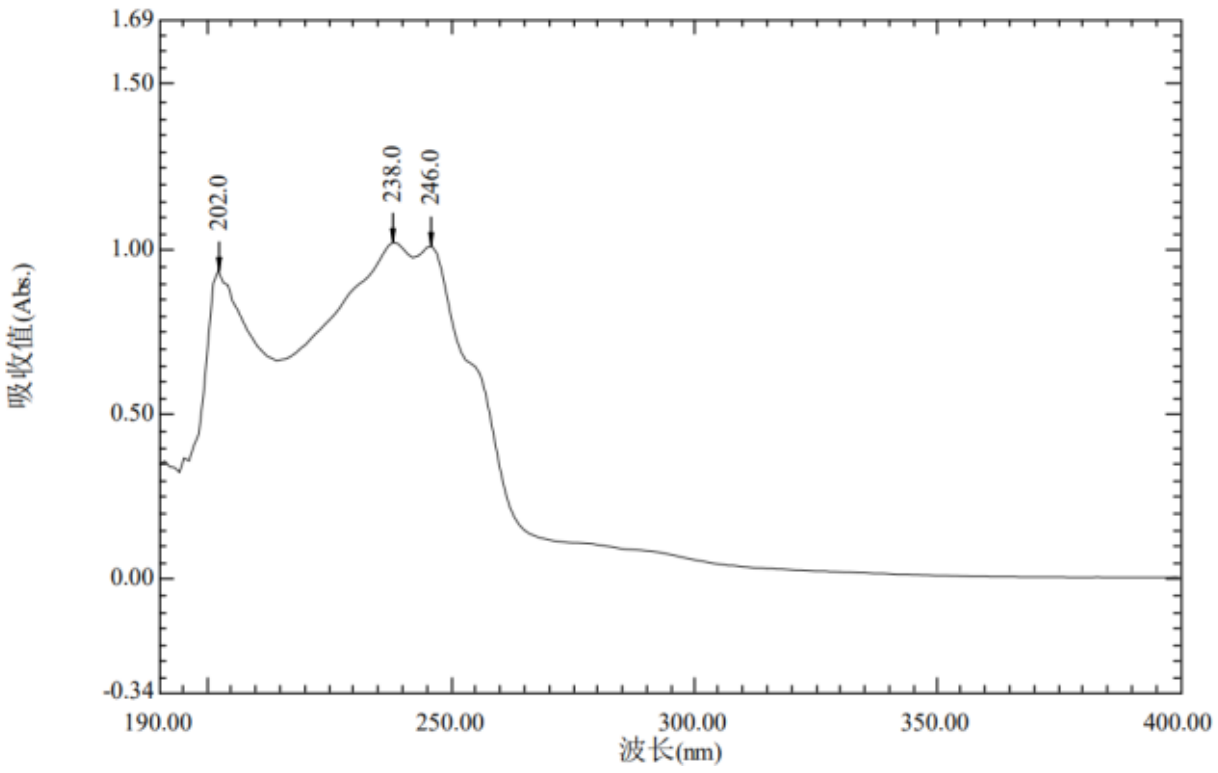

Figure S8.1 UV spectrum of compound **8**

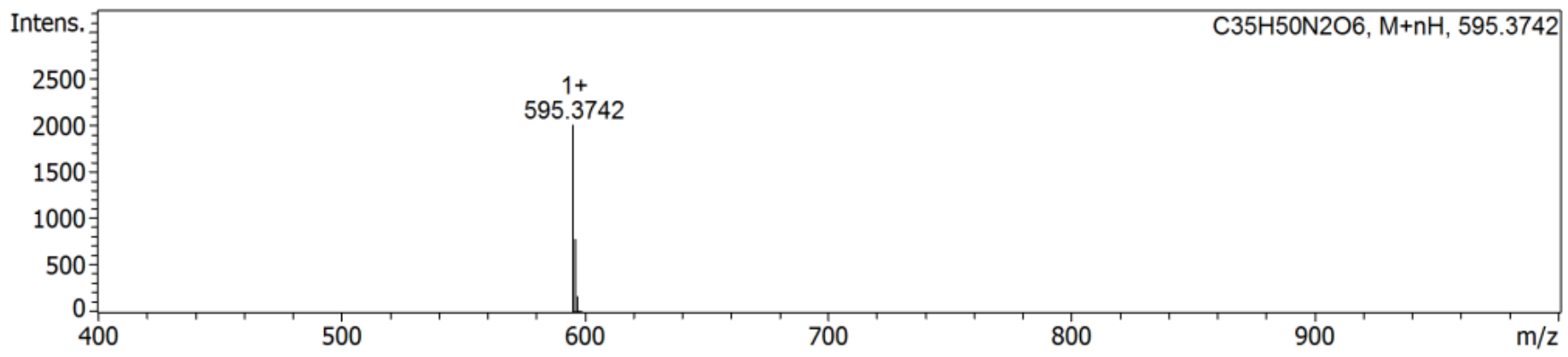

Figure S8.2 HR-ESI-MS spectrum of compound **8**

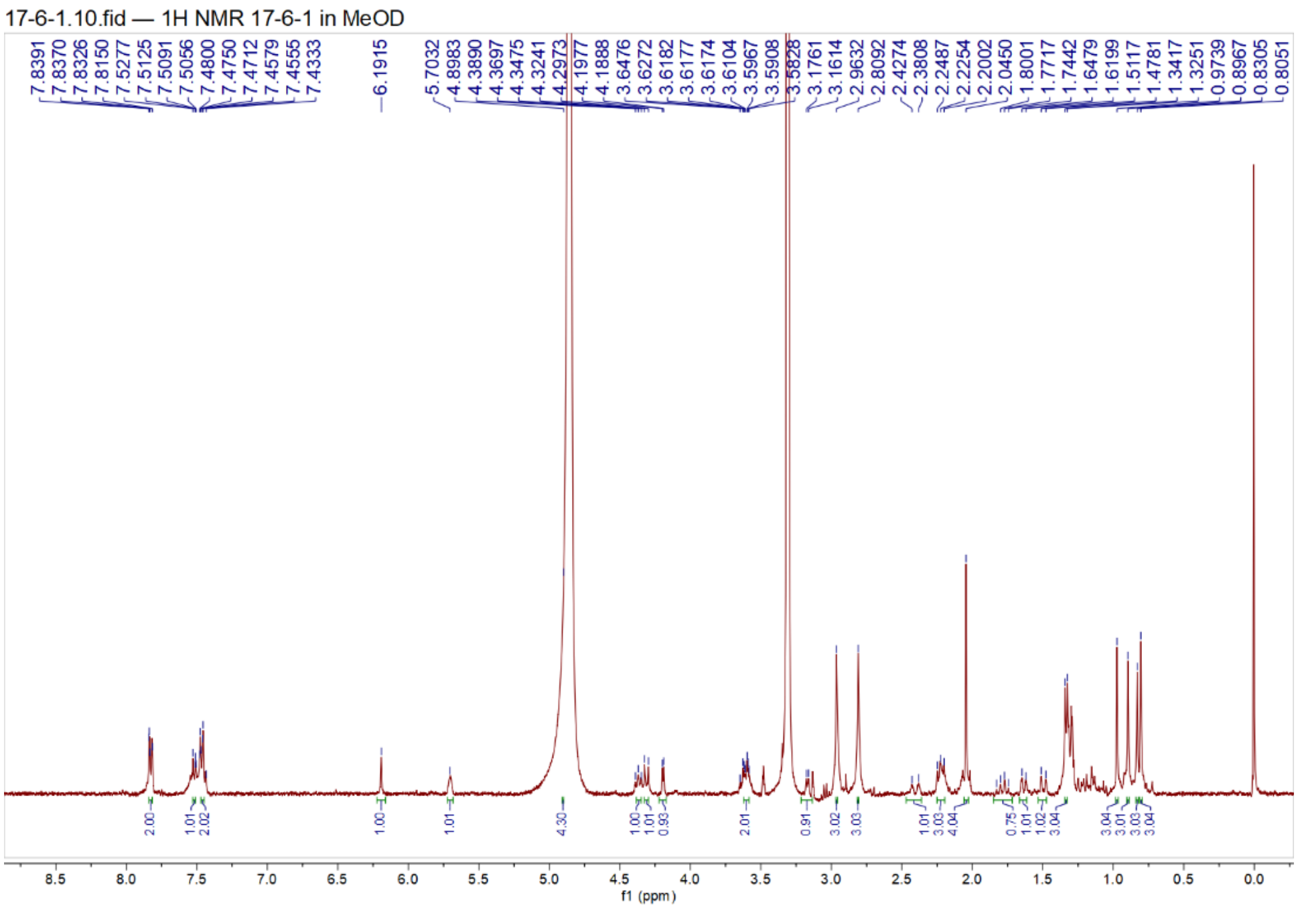

Figure S8.3 ^{1}H NMR spectrum of compound **8** (400 MHz, MeOD)

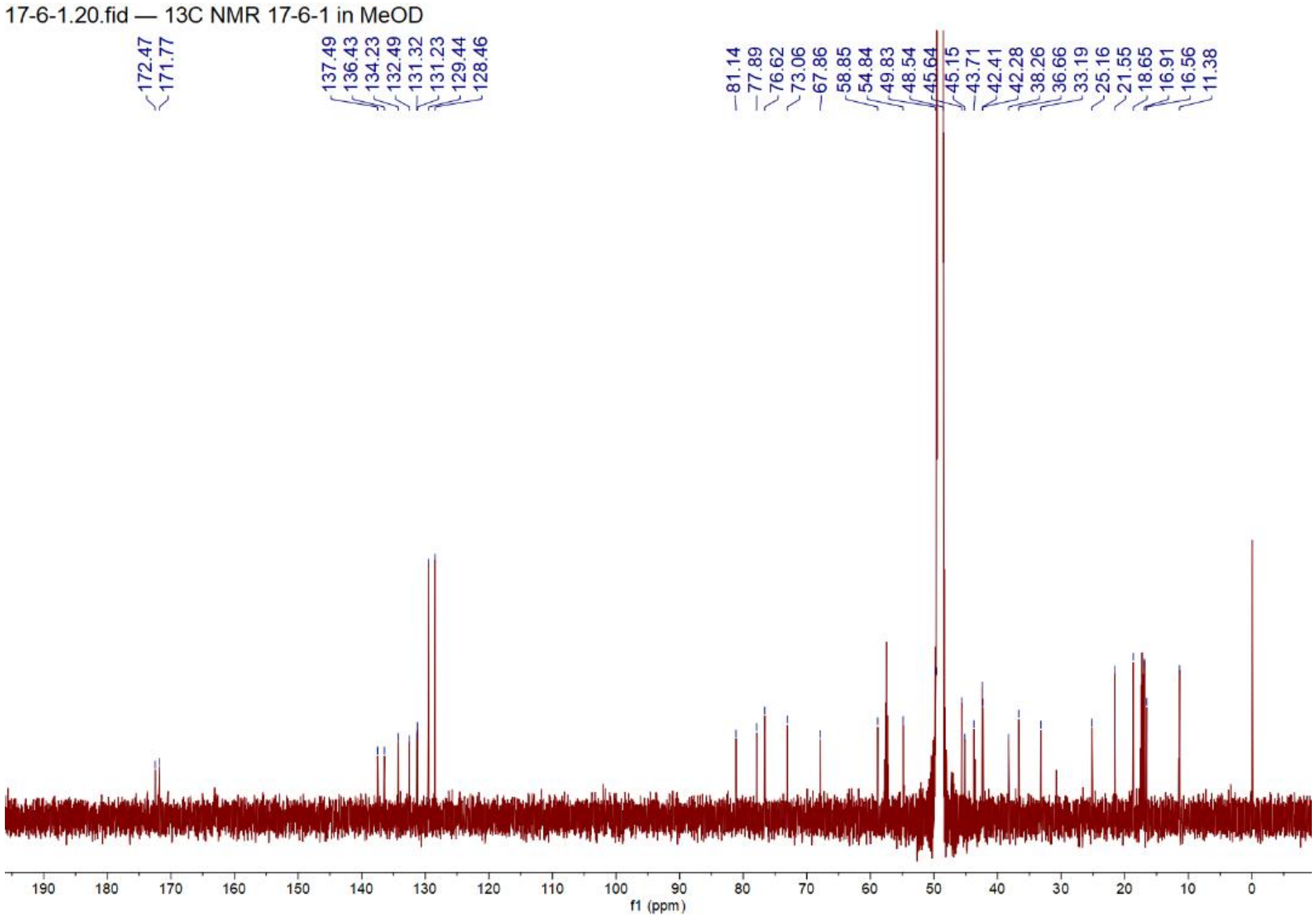

Figure S8.4 ^{13}C NMR spectrum of compound **8** (100 MHz, MeOD)

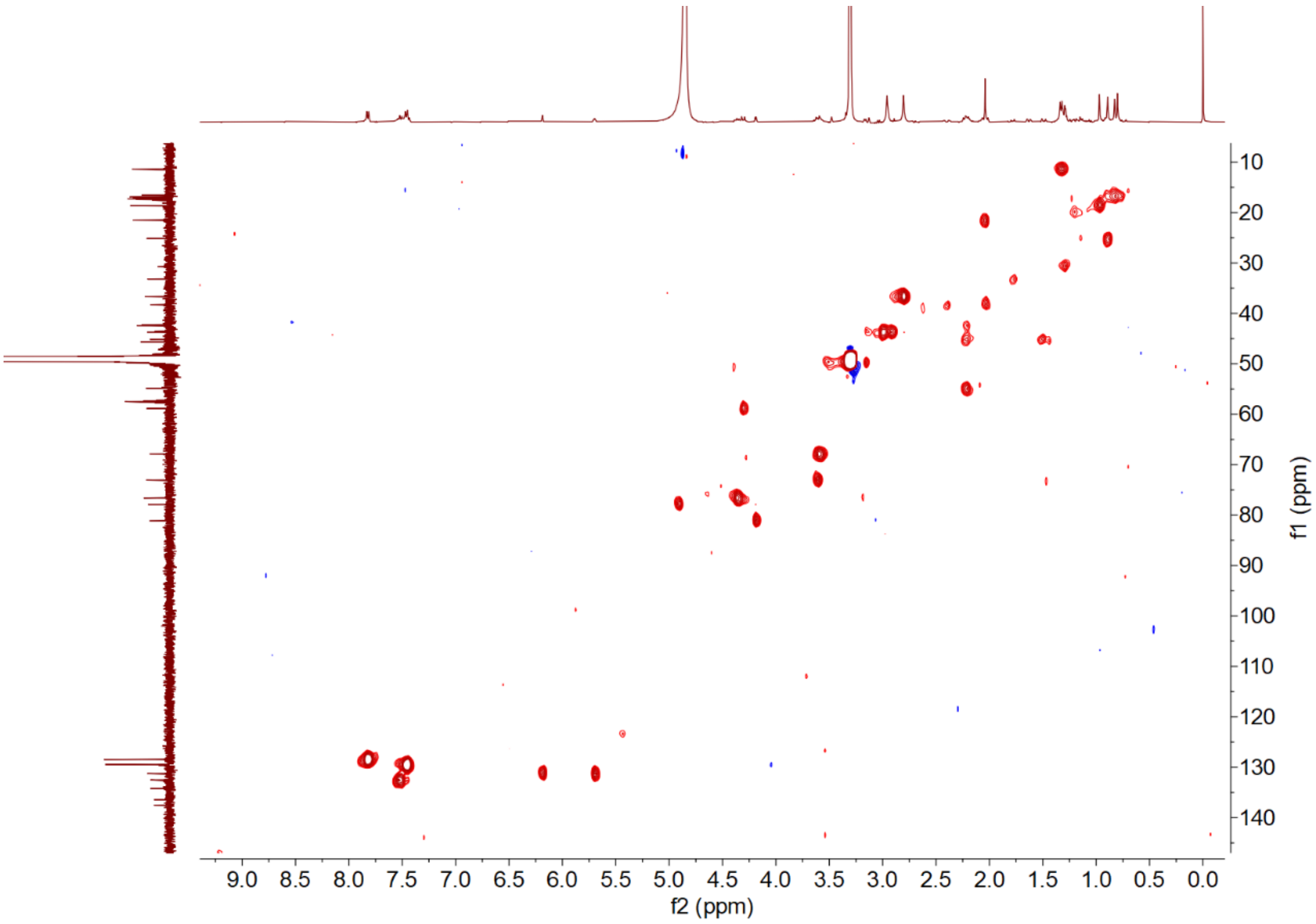

Figure S8.5 HSQC spectrum of compound **8**

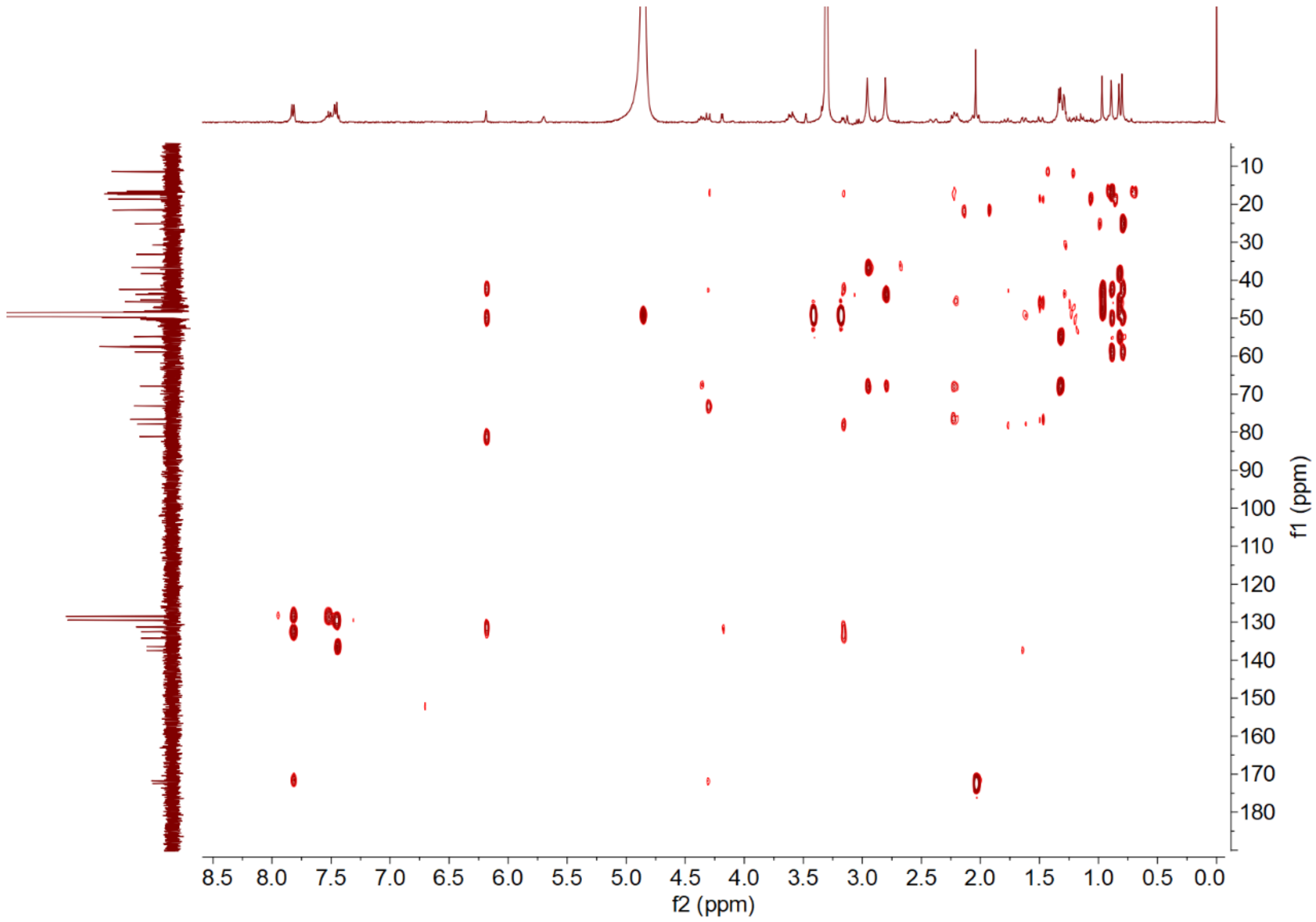

Figure S8.6 HMBC spectrum of compound **8**

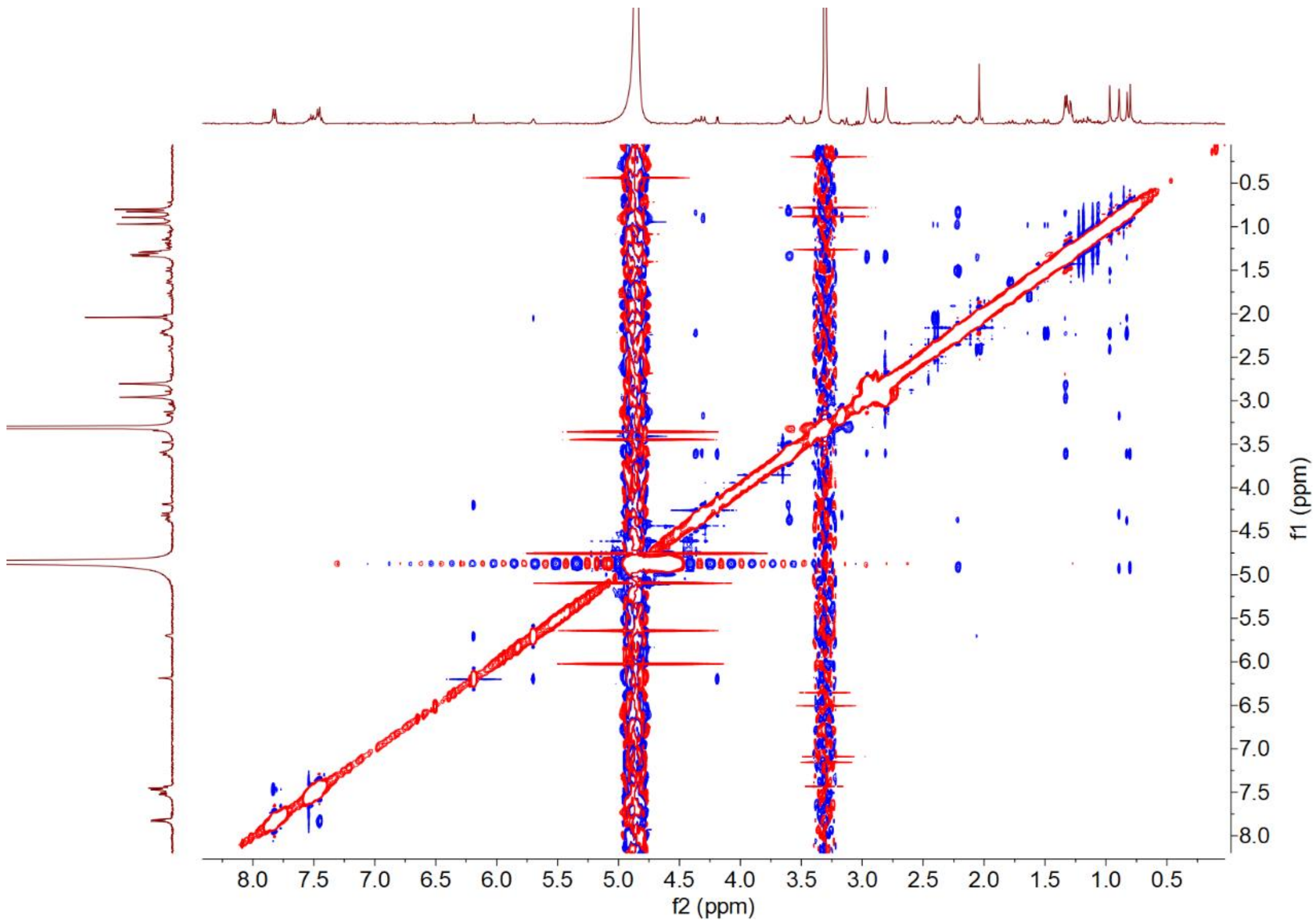

Figure S8.7 NOESY spectrum of compound **8**

Table S4 Experimental/calculated NMR shifts and statistical parameters for **8**

Position / Parameter	Experimental	Isomer 8a	Isomer 8b

C	81.1	71.19319	79.99836
C	73.1	75.89836	86.37014
C	58.8	65.77913	72.27295
C	42.3	42.21408	47.17376
C	49.8	54.06707	56.14462
C	77.9	76.19516	78.29401
C	33.2	38.9479	34.95345
C	42.4	48.20842	45.4511
C	137.5	149.0637	147.7905
C	134.2	153.2	138.7
C	131.2	135.7	144.1
C	38.3	41.75	43.67
C	45.6	52.16	49.63
C	48.5	53.27	55.11
C	45.2	45.99	43.82
C	76.6	83.57	83.59
C	54.8	60.81	61.89
C	16.6	18.94	18.85
C	131.3	137.91	136.80
C	67.9	67.53	67.49
C	11.4	12.01	11.86
C	18.6	20.44	19.69
C	16.9	26.19	19.18
C	25.2	35.17	25.85
C	136.4	139.40	140.23
C	128.5	136.8628	139.606
C	128.5	134.7426	137.6428
C	129.4	135.4187	135.8937
C	129.4	135.4043	135.3552
C	132.5	139.8342	140.9128
C	171.8	174.2794	187.9489
C	43.7	46.09095	46.47498
C	36.7	37.22251	37.63502
C	172.5	184.8772	181.6369
C	21.6	25.93694	24.7831566027
**^{13}C MAE (unscaled)**		**5.459**	**5.520**
**^{13}C CMAE**		**3.046**	**2.603**
**^{13}C R^2**		**0.9920**	**0.9952**
**^{13}C sDP4$^+$ Probability**		**0.01%**	**99.99%**
**^{13}C uDP4$^+$ Probability**		**0.84%**	**99.16%**
**^{13}C DP4$^+$ Probability**		**0.00%**	**100.00%**
H	4.31	4.185815	3.533539
H	3.62	4.56466	3.658124

H	4.19	4.561536	3.856805
H	3.17	3.164416	2.504775
H	4.9	5.79179	5.342438
H	1.79	2.630791	2.373104
H	1.63	1.727209	2.083935
H	2.22	2.657827	3.001815
H	5.7	5.964301	6.157551
H	2.4	2.607054	2.577166
H	2.04	2.256612	2.193879
H	2.22	2.204768	2.012942
H	1.49	1.543365	1.416165
H	4.37	4.543788	4.446599
H	2.22	2.132357	1.869942
H	0.83	0.874807	0.811879
H	6.18	7.237449	7.093021
H	3.59	2.934772	2.598228
H	1.33	1.056899	1.016579
H	0.97	1.677275	1.237338
H	0.81	1.181715	1.494885
H	0.9	1.304449	1.137586
H	7.83	8.887935	8.568041
H	7.83	8.87325	8.371373
H	7.46	8.06958	8.123636
H	7.46	8.024057	8.051533
H	7.51	8.071461	8.134823
H	2.96	2.496893	2.552378
H	2.81	2.334218	2.21051
H	2.04	2.297529	2.047573
**^{1}H MAE (unscaled)**		**0.442**	**0.439**
**^{1}H sDP4^{+} Probability**		**99.97%**	**0.03%**
**^{1}H uDP4^{+} Probability**		**10.36%**	**89.64%**
**^{1}H DP4^{+} Probability**		**99.73%**	**0.27%**
**sDP4^{+} Probability (All Data)**		**21.00%**	**79.00%**
**uDP4^{+} Probability (All Data)**		**0.10%**	**99.90%**
**DP4^{+} Probability (All Data)**		**0.03%**	**99.97%**

Table S5 Relative energies and Boltzmann populations for **8a**

Conformer	Energy (Hartree)	Δ*G* (kcal/mol)	Population (%)
8a-1	−1923.890788	0.00	99.63

Conformer	Energy (Hartree)	ΔG (kcal/mol)	Population (%)
8a-2	−1923.885465	3.34	0.35
8a-3	−1923.878764	7.55	0.00
8a-4	−1923.876940	8.69	0.00
8a-5	−1923.876939	8.69	0.00
8a-6	−1923.877286	8.47	0.00
8a-7	−1923.882418	5.25	0.01
8a-8	−1923.880810	6.26	0.00

Cartesian coordinates of **8a-1**

Center Number	Atomic Number	Atomic Type	Coordinates (Angstroms) X	Y	Z
1	6	0	3.435113	2.582388	0.029953
2	6	0	3.467172	1.391742	1.020291
3	6	0	2.074918	0.998760	1.597317
4	6	0	0.962108	0.836170	0.493701
5	6	0	0.989251	2.010417	-0.488130
6	6	0	2.340629	2.447411	-1.034078
7	6	0	0.836643	-0.448392	-0.353334
8	6	0	-0.301176	-0.368671	-1.402485
9	6	0	-1.632551	0.421969	-1.180876
10	6	0	-1.470594	1.874213	-0.696792
11	6	0	-2.795713	-0.260665	-0.416693
12	6	0	-4.091012	0.646309	-0.581849
13	6	0	-3.859341	1.972675	0.169054
14	6	0	-2.489852	2.543601	-0.128880
15	6	0	2.230682	-0.220244	2.532319
16	6	0	1.579211	2.155263	2.508996
17	7	0	4.183796	0.275792	0.383144
18	6	0	-0.157657	2.513584	-0.965230
19	8	0	3.209464	3.838233	0.685320
20	6	0	-3.271975	-1.618920	-0.983522
21	6	0	-4.785126	-1.697344	-0.659606
22	6	0	-5.187496	-0.320880	-0.043241
23	6	0	-4.388250	0.999263	-2.062313
24	6	0	-2.435194	-0.486250	1.072896

25	6	0	−6.688080	−0.032046	−0.272366
26	6	0	−7.163303	1.342366	0.230186
27	7	0	−7.478203	−1.174456	0.286504
28	6	0	−7.686622	−1.123484	1.735368
29	6	0	−8.747156	−1.378890	−0.408210
30	8	0	−5.058579	−2.783546	0.214410
31	6	0	5.554742	0.320457	0.342120
32	6	0	6.288985	−0.861238	−0.228425
33	8	0	6.167691	1.309369	0.753074
34	6	0	5.676209	−1.906228	−0.934367
35	6	0	6.444789	−2.955572	−1.439472
36	6	0	7.826969	−2.973222	−1.247514
37	6	0	8.443710	−1.931764	−0.549520
38	6	0	7.679860	−0.881598	−0.047992
39	1	0	0.016346	0.902257	1.040323
40	8	0	0.578929	−1.618428	0.486852
41	8	0	2.278235	3.628712	−1.812489
42	1	0	−5.044050	−0.402222	1.039566
43	1	0	−1.986878	0.502831	−2.219272
44	6	0	1.482758	−2.613098	0.503893
45	6	0	0.984853	−3.793120	1.300304
46	8	0	2.577918	−2.554582	−0.028323
47	1	0	4.402556	2.619614	−0.479658
48	1	0	4.094369	1.710699	1.862040
49	1	0	2.679030	1.652865	−1.716618
50	1	0	1.761426	−0.659289	−0.892860
51	1	0	−0.560117	−1.398160	−1.675181
52	1	0	0.157798	0.063408	−2.297392
53	1	0	−4.617569	2.716873	−0.108432
54	1	0	−3.973115	1.836593	1.255342
55	1	0	−2.326879	3.581612	0.161337
56	1	0	1.256705	−0.533286	2.922562
57	1	0	2.857038	0.062313	3.387080
58	1	0	2.707706	−1.082294	2.064613
59	1	0	2.340511	2.411992	3.253945
60	1	0	1.341739	3.059727	1.949137
61	1	0	0.680208	1.831790	3.047252
62	1	0	3.709234	−0.604195	0.218789
63	1	0	−0.109611	3.355090	−1.653673
64	1	0	4.048653	4.110876	1.086848
65	1	0	−2.753485	−2.468125	−0.528132
66	1	0	−3.107748	−1.682676	−2.065915
67	1	0	−5.352483	−1.839202	−1.594356
68	1	0	−3.662830	1.713549	−2.461451

69	1	0	-4.400475	0.123879	-2.719549
70	1	0	-5.370005	1.478812	-2.141428
71	1	0	-1.529185	-1.091977	1.137149
72	1	0	-2.253509	0.446649	1.613936
73	1	0	-3.217171	-1.042521	1.596099
74	1	0	-6.874470	-0.073176	-1.352553
75	1	0	-8.254284	1.429129	0.166596
76	1	0	-6.739314	2.147981	-0.375796
77	1	0	-6.871368	1.522654	1.270432
78	1	0	-8.128012	-2.071372	2.063099
79	1	0	-6.731441	-1.005325	2.254118
80	1	0	-8.360699	-0.311445	2.057503
81	1	0	-8.567114	-1.511897	-1.479821
82	1	0	-9.465145	-0.548211	-0.278744
83	1	0	-9.224121	-2.289494	-0.028945
84	1	0	-6.023787	-2.704781	0.355623
85	1	0	4.605446	-1.918642	-1.101771
86	1	0	5.958947	-3.759563	-1.985642
87	1	0	8.421466	-3.793013	-1.642545
88	1	0	9.520066	-1.937821	-0.399299
89	1	0	8.137798	-0.057151	0.487779
90	1	0	2.370641	4.351227	-1.164889
91	1	0	-0.035316	-4.056080	1.006979
92	1	0	1.655724	-4.640516	1.155139
93	1	0	0.963370	-3.527180	2.363350

Cartesian coordinates of **8a-2**

Center Number	Atomic Number	Atomic Type	Coordinates (Angstroms) X	Y	Z
1	6	0	3.274852	2.204707	-0.671978
2	6	0	3.494696	1.358418	0.618101
3	6	0	2.188120	1.043450	1.431894
4	6	0	0.914848	1.015500	0.515025
5	6	0	0.782669	2.330087	-0.269199
6	6	0	2.028290	3.097906	-0.676807
7	6	0	0.730247	-0.163690	-0.473330
8	6	0	-0.445603	0.002040	-1.460950
9	6	0	-1.803575	0.667946	-1.079965
10	6	0	-1.687593	2.044051	-0.409062
11	6	0	-2.897020	-0.168239	-0.365356
12	6	0	-4.242841	0.674531	-0.356080

13	6	0	-4.032524	1.895396	0.565696
14	6	0	-2.710202	2.577031	0.285218
15	6	0	2.354536	-0.220572	2.303180
16	6	0	1.971964	2.197571	2.451359
17	7	0	4.287725	0.161393	0.285688
18	6	0	-0.425436	2.791132	-0.626302
19	8	0	4.371216	3.078056	-0.905897
20	6	0	-3.344974	-1.453763	-1.086644
21	6	0	-4.778907	-1.742379	-0.560719
22	6	0	-5.293931	-0.409166	0.084077
23	6	0	-4.608957	1.226768	-1.757865
24	6	0	-2.452422	-0.534090	1.072936
25	6	0	-6.792389	-0.137246	-0.223970
26	6	0	-7.343618	1.068319	0.566491
27	7	0	-7.594480	-1.366255	-0.068131
28	6	0	-7.767918	-1.845604	1.296184
29	6	0	-8.858992	-1.325965	-0.785061
30	8	0	-4.823321	-2.857777	0.334056
31	6	0	5.633405	0.247753	0.108240
32	6	0	6.404200	-1.013672	-0.152339
33	8	0	6.227087	1.338647	0.172964
34	6	0	5.853519	-2.302572	-0.089090
35	6	0	6.651635	-3.417803	-0.343361
36	6	0	8.003054	-3.262211	-0.656315
37	6	0	8.557873	-1.981841	-0.716713
38	6	0	7.763490	-0.866237	-0.466668
39	1	0	0.059423	0.963183	1.197723
40	8	0	0.509437	-1.420941	0.250693
41	8	0	2.233376	4.244054	0.149322
42	1	0	-5.213277	-0.522830	1.172509
43	1	0	-2.216212	0.864548	-2.080145
44	6	0	1.402740	-2.414041	0.126191
45	6	0	0.913547	-3.678391	0.788166
46	8	0	2.484740	-2.301729	-0.427049
47	1	0	3.169313	1.495251	-1.510470
48	1	0	4.141819	1.950974	1.270830
49	1	0	1.895229	3.443256	-1.714401
50	1	0	1.632364	-0.312314	-1.070334
51	1	0	-0.654705	-0.987961	-1.882681
52	1	0	-0.044800	0.592896	-2.290557
53	1	0	-4.839452	2.628663	0.438107
54	1	0	-4.075509	1.602658	1.626035
55	1	0	-2.583377	3.574190	0.706909
56	1	0	1.425694	-0.422618	2.848190

57	1	0	3.143739	−0.049547	3.043640
58	1	0	2.632588	−1.118523	1.755499
59	1	0	2.803406	2.217994	3.165604
60	1	0	1.909524	3.176064	1.973660
61	1	0	1.050460	2.024892	3.020578
62	1	0	3.800814	−0.684812	0.008498
63	1	0	−0.486131	3.752838	−1.136918
64	1	0	5.179934	2.630925	−0.566686
65	1	0	−2.691735	−2.312797	−0.896529
66	1	0	−3.359315	−1.305288	−2.173433
67	1	0	−5.447274	−2.042124	−1.370120
68	1	0	−4.671217	0.449047	−2.525888
69	1	0	−5.583072	1.723540	−1.718483
70	1	0	−3.895696	1.983269	−2.094812
71	1	0	−1.627499	−1.247978	1.035856
72	1	0	−2.108742	0.339500	1.631873
73	1	0	−3.258176	−0.984360	1.660467
74	1	0	−6.873586	0.098496	−1.291705
75	1	0	−7.153045	0.966006	1.641157
76	1	0	−8.425976	1.167104	0.426973
77	1	0	−6.888416	2.007786	0.239027
78	1	0	−8.263698	−2.822271	1.264030
79	1	0	−6.794736	−1.995715	1.767583
80	1	0	−8.381337	−1.181013	1.933578
81	1	0	−8.687658	−1.042721	−1.829946
82	1	0	−9.606620	−0.626268	−0.362239
83	1	0	−9.308574	−2.325848	−0.777125
84	1	0	−4.160778	−2.704396	1.025349
85	1	0	4.807301	−2.458299	0.150771
86	1	0	6.214368	−4.411312	−0.293832
87	1	0	8.620829	−4.134786	−0.851936
88	1	0	9.609209	−1.853152	−0.959414
89	1	0	8.174389	0.136513	−0.506101
90	1	0	3.172948	4.467543	−0.005095
91	1	0	0.808613	−3.509413	1.865513
92	1	0	−0.071856	−3.956039	0.401747
93	1	0	1.626814	−4.483945	0.611616

Table S6 Relative energies and Boltzmann populations for **8b**

Conformer	Energy (Hartree)	Δ*G* (kcal/mol)	Population (%)
8b-1	−1923.888806	1.36	8.32

Conformer	Energy (Hartree)	Δ*G* (kcal/mol)	Population (%)
8b-2	−1923.890978	0.00	82.98
8b-3	−1923.888811	1.36	8.36
8b-4	−1923.885022	3.74	0.15
8b-5	−1923.885185	3.64	0.18
8b-6	−1923.882159	5.53	0.01
8b-7	−1923.880719	6.44	0.00

Cartesian coordinates of **8b-1**

Center Number	Atomic Number	Atomic Type	Coordinates (Angstroms) X	Y	Z
1	6	0	-3.349368	-1.616625	1.437336
2	6	0	-3.714465	-1.085342	0.041383
3	6	0	-2.444840	-0.708971	-0.783699
4	6	0	-1.734683	0.373208	0.074371
5	6	0	-1.285180	-0.224366	1.372003
6	6	0	-2.472899	-0.613631	2.217981
7	6	0	-0.648944	1.240882	-0.580598
8	6	0	0.561583	0.548208	-1.184428
9	6	0	1.547959	-0.175388	-0.239916
10	6	0	1.272133	-0.178968	1.253623
11	6	0	2.983098	0.348458	-0.465593
12	6	0	3.988447	-0.479560	0.387409
13	6	0	3.750481	-0.112269	1.848765
14	6	0	2.282964	-0.165708	2.138466
15	6	0	-2.905124	-0.131917	-2.123041
16	6	0	-1.581841	-1.952801	-1.019276
17	7	0	-4.556118	-2.043915	-0.627822
18	6	0	-0.047098	-0.404921	1.828253
19	8	0	-4.492514	-1.939238	2.196079
20	6	0	3.517945	0.165871	-1.894663
21	6	0	5.035209	-0.049518	-1.754556
22	6	0	5.339133	-0.074711	-0.235350
23	6	0	3.775161	-1.992547	0.244991
24	6	0	3.027680	1.842003	-0.103785
25	6	0	6.600611	-0.910188	0.040296
26	6	0	6.952944	-1.005472	1.528142

27	7	0	7.682605	-0.355559	-0.787508
28	6	0	8.351583	0.805129	-0.229874
29	6	0	8.647894	-1.347980	-1.210880
30	8	0	5.729714	0.972522	-2.423287
31	6	0	-5.768551	-1.844339	-1.199025
32	6	0	-6.381147	-0.492520	-0.992829
33	8	0	-6.321092	-2.724241	-1.826519
34	6	0	-6.430671	0.425186	-2.033980
35	6	0	-7.025126	1.660093	-1.841000
36	6	0	-7.584326	1.979723	-0.615290
37	6	0	-7.560840	1.056736	0.417199
38	6	0	-6.962145	-0.176763	0.230506
39	1	0	-2.522261	1.100321	0.322922
40	8	0	-0.158597	2.173123	0.394077
41	8	0	-2.100424	-1.174835	3.449426
42	1	0	5.562863	0.949189	0.071898
43	1	0	1.524800	-1.229074	-0.541033
44	6	0	-0.936472	3.191820	0.744953
45	8	0	-2.030464	3.402845	0.284876
46	6	0	-0.261789	4.034995	1.795208
47	1	0	-2.802643	-2.561470	1.351148
48	1	0	-4.312456	-0.173703	0.165261
49	1	0	-3.085742	0.292789	2.401638
50	1	0	-1.138539	1.826878	-1.372199
51	1	0	0.198706	-0.150767	-1.933715
52	1	0	1.108429	1.316202	-1.728327
53	1	0	4.252988	-0.814447	2.517902
54	1	0	4.148537	0.879632	2.077850
55	1	0	2.039349	-0.220043	3.192099
56	1	0	-2.055062	0.108905	-2.755185
57	1	0	-3.520933	-0.859099	-2.647598
58	1	0	-3.490260	0.772328	-1.970503
59	1	0	-0.895971	-1.799315	-1.846376
60	1	0	-1.002181	-2.198953	-0.133817
61	1	0	-2.204356	-2.804706	-1.281499
62	1	0	-4.197620	-2.984874	-0.720355
63	1	0	-0.003869	-0.795214	2.838344
64	1	0	-5.063239	-1.161737	2.245220
65	1	0	3.355340	1.044693	-2.515509
66	1	0	3.050999	-0.687129	-2.385669
67	1	0	5.322779	-1.018630	-2.192038
68	1	0	2.854720	-2.299329	0.733966
69	1	0	3.745033	-2.298229	-0.796324
70	1	0	4.587088	-2.526371	0.731811

71	1	0	2.488870	2.426773	-0.844113
72	1	0	2.558524	2.025773	0.857423
73	1	0	4.044391	2.219347	-0.090830
74	1	0	6.433519	-1.923072	-0.339968
75	1	0	6.257336	-1.661210	2.042119
76	1	0	6.922653	-0.028858	2.003715
77	1	0	7.951212	-1.422687	1.648689
78	1	0	8.957313	1.264478	-1.012545
79	1	0	7.617876	1.534938	0.103575
80	1	0	9.014475	0.561619	0.612050
81	1	0	9.344451	-0.883684	-1.908985
82	1	0	8.134071	-2.157950	-1.726722
83	1	0	9.230594	-1.773698	-0.380123
84	1	0	6.668360	0.815901	-2.244021
85	1	0	-6.005644	0.167514	-2.992445
86	1	0	-7.054409	2.373561	-2.651054
87	1	0	-8.045867	2.944397	-0.466396
88	1	0	-8.011291	1.297574	1.369058
89	1	0	-6.960700	-0.909931	1.025247
90	1	0	-2.848303	-1.706648	3.755470
91	1	0	0.754179	4.264817	1.485717
92	1	0	-0.827363	4.947606	1.955503
93	1	0	-0.215053	3.463659	2.720214

Cartesian coordinates of **8b-2**

Center Number	Atomic Number	Atomic Type	Coordinates (Angstroms) X	Y	Z
1	6	0	3.274852	2.204707	-0.671978
2	6	0	3.494696	1.358418	0.618101
3	6	0	2.188120	1.043450	1.431894
4	6	0	0.914848	1.015500	0.515025
5	6	0	0.782669	2.330087	-0.269199
6	6	0	2.028290	3.097906	-0.676807
7	6	0	0.730247	-0.163690	-0.473330
8	6	0	-0.445603	0.002040	-1.460950
9	6	0	-1.803575	0.667946	-1.079965
10	6	0	-1.687593	2.044051	-0.409062
11	6	0	-2.897020	-0.168239	-0.365356
12	6	0	-4.242841	0.674531	-0.356080
13	6	0	-4.032524	1.895396	0.565696
14	6	0	-2.710202	2.577031	0.285218

15	6	0	2.354536	−0.220572	2.303180
16	6	0	1.971964	2.197571	2.451359
17	7	0	4.287725	0.161393	0.285688
18	6	0	−0.425436	2.791132	−0.626302
19	8	0	4.371216	3.078056	−0.905897
20	6	0	−3.344974	−1.453763	−1.086644
21	6	0	−4.778907	−1.742379	−0.560719
22	6	0	−5.293931	−0.409166	0.084077
23	6	0	−4.608957	1.226768	−1.757865
24	6	0	−2.452422	−0.534090	1.072936
25	6	0	−6.792389	−0.137246	−0.223970
26	6	0	−7.343618	1.068319	0.566491
27	7	0	−7.594480	−1.366255	−0.068131
28	6	0	−7.767918	−1.845604	1.296184
29	6	0	−8.858992	−1.325965	−0.785061
30	8	0	−4.823321	−2.857777	0.334056
31	6	0	5.633405	0.247753	0.108240
32	6	0	6.404200	−1.013672	−0.152339
33	8	0	6.227087	1.338647	0.172964
34	6	0	5.853519	−2.302572	−0.089090
35	6	0	6.651635	−3.417803	−0.343361
36	6	0	8.003054	−3.262211	−0.656315
37	6	0	8.557873	−1.981841	−0.716713
38	6	0	7.763490	−0.866237	−0.466668
39	1	0	0.059423	0.963183	1.197723
40	8	0	0.509437	−1.420941	0.250693
41	8	0	2.233376	4.244054	0.149322
42	1	0	−5.213277	−0.522830	1.172509
43	1	0	−2.216212	0.864548	−2.080145
44	6	0	1.402740	−2.414041	0.126191
45	6	0	0.913547	−3.678391	0.788166
46	8	0	2.484740	−2.301729	−0.427049
47	1	0	3.169313	1.495251	−1.510470
48	1	0	4.141819	1.950974	1.270830
49	1	0	1.895229	3.443256	−1.714401
50	1	0	1.632364	−0.312314	−1.070334
51	1	0	−0.654705	−0.987961	−1.882681
52	1	0	−0.044800	0.592896	−2.290557
53	1	0	−4.839452	2.628663	0.438107
54	1	0	−4.075509	1.602658	1.626035
55	1	0	−2.583377	3.574190	0.706909
56	1	0	1.425694	−0.422618	2.848190
57	1	0	3.143739	−0.049547	3.043640
58	1	0	2.632588	−1.118523	1.755499

59	1	0	2.803406	2.217994	3.165604
60	1	0	1.909524	3.176064	1.973660
61	1	0	1.050460	2.024892	3.020578
62	1	0	3.800814	-0.684812	0.008498
63	1	0	-0.486131	3.752838	-1.136918
64	1	0	5.179934	2.630925	-0.566686
65	1	0	-2.691735	-2.312797	-0.896529
66	1	0	-3.359315	-1.305288	-2.173433
67	1	0	-5.447274	-2.042124	-1.370120
68	1	0	-4.671217	0.449047	-2.525888
69	1	0	-5.583072	1.723540	-1.718483
70	1	0	-3.895696	1.983269	-2.094812
71	1	0	-1.627499	-1.247978	1.035856
72	1	0	-2.108742	0.339500	1.631873
73	1	0	-3.258176	-0.984360	1.660467
74	1	0	-6.873586	0.098496	-1.291705
75	1	0	-7.153045	0.966006	1.641157
76	1	0	-8.425976	1.167104	0.426973
77	1	0	-6.888416	2.007786	0.239027
78	1	0	-8.263698	-2.822271	1.264030
79	1	0	-6.794736	-1.995715	1.767583
80	1	0	-8.381337	-1.181013	1.933578
81	1	0	-8.687658	-1.042721	-1.829946
82	1	0	-9.606620	-0.626268	-0.362239
83	1	0	-9.308574	-2.325848	-0.777125
84	1	0	-4.160778	-2.704396	1.025349
85	1	0	4.807301	-2.458299	0.150771
86	1	0	6.214368	-4.411312	-0.293832
87	1	0	8.620829	-4.134786	-0.851936
88	1	0	9.609209	-1.853152	-0.959414
89	1	0	8.174389	0.136513	-0.506101
90	1	0	3.172948	4.467543	-0.005095
91	1	0	0.808613	-3.509413	1.865513
92	1	0	-0.071856	-3.956039	0.401747
93	1	0	1.626814	-4.483945	0.611616

Cartesian coordinates of **8b-3**

Center Number	Atomic Number	Atomic Type	Coordinates (Angstroms) X	Y	Z
1	6	0	-3.406805	-2.154230	0.710609

2	6	0	-3.799859	-1.067549	-0.306242
3	6	0	-2.560513	-0.452474	-1.014948
4	6	0	-1.734923	0.163623	0.147843
5	6	0	-1.274861	-0.909173	1.086943
6	6	0	-2.443713	-1.600794	1.775608
7	6	0	-0.625262	1.164775	-0.212548
8	6	0	0.536752	0.658079	-1.055620
9	6	0	1.580901	-0.264992	-0.389506
10	6	0	1.288908	-0.784944	1.004421
11	6	0	2.978618	0.390169	-0.383537
12	6	0	4.027833	-0.614647	0.177760
13	6	0	3.747903	-0.786028	1.667601
14	6	0	2.283682	-1.018546	1.874923
15	6	0	-3.054870	0.632647	-1.973826
16	6	0	-1.793275	-1.531090	-1.791916
17	7	0	-4.770135	-1.628372	-1.216926
18	6	0	-0.029960	-1.238691	1.428837
19	8	0	-4.546397	-2.654303	1.365408
20	6	0	3.541852	0.727265	-1.773225
21	6	0	5.069023	0.574528	-1.656044
22	6	0	5.353157	0.063148	-0.221068
23	6	0	3.926805	-1.999349	-0.476604
24	6	0	2.921467	1.681825	0.445115
25	6	0	6.670909	-0.728994	-0.189110
26	6	0	7.006308	-1.293901	1.194898
27	7	0	7.719877	0.142943	-0.739434
28	6	0	8.280987	1.098177	0.197903
29	6	0	8.772030	-0.583133	-1.418880
30	8	0	5.696294	1.803301	-1.921491
31	6	0	-5.941496	-1.078619	-1.642963
32	6	0	-6.450709	0.104047	-0.888932
33	8	0	-6.538532	-1.564209	-2.583098
34	6	0	-6.721161	1.272395	-1.592302
35	6	0	-7.220599	2.380927	-0.933306
36	6	0	-7.471103	2.324136	0.427555
37	6	0	-7.226900	1.154295	1.127307
38	6	0	-6.716879	0.045023	0.475781
39	1	0	-2.454328	0.774526	0.713712
40	8	0	-0.096094	1.708741	1.003182
41	8	0	-2.063550	-2.595890	2.684933
42	1	0	5.492059	0.936789	0.419184
43	1	0	1.637365	-1.152564	-1.031299
44	6	0	-0.799876	2.652545	1.622132
45	8	0	-1.854579	3.080539	1.226728

46	6	0	−0.090062	3.111491	2.868891
47	1	0	−2.897525	−2.980736	0.180732
48	1	0	−4.306922	−0.265967	0.238760
49	1	0	−2.992310	−0.864532	2.377021
50	1	0	−1.105892	1.993102	−0.752335
51	1	0	0.110053	0.156437	−1.918903
52	1	0	1.057996	1.534240	−1.438892
53	1	0	4.287309	−1.645865	2.071861
54	1	0	4.079140	0.087146	2.235838
55	1	0	2.031146	−1.433505	2.842871
56	1	0	−2.228480	1.068145	−2.528688
57	1	0	−3.753343	0.207802	−2.691625
58	1	0	−3.562590	1.424819	−1.427960
59	1	0	−2.471672	−2.280296	−2.194668
60	1	0	−1.270958	−1.100101	−2.641446
61	1	0	−1.068115	−2.030231	−1.153668
62	1	0	−4.457967	−2.403259	−1.789986
63	1	0	0.029684	−1.976598	2.223444
64	1	0	−5.206148	−2.876949	0.695560
65	1	0	3.325844	1.751606	−2.070879
66	1	0	3.143184	0.060317	−2.536926
67	1	0	5.432278	−0.172686	−2.379317
68	1	0	3.025678	−2.513215	−0.153189
69	1	0	3.930663	−1.934366	−1.560332
70	1	0	4.770233	−2.613057	−0.170873
71	1	0	2.277543	2.407522	−0.043622
72	1	0	2.510739	1.500624	1.432575
73	1	0	3.900994	2.139494	0.533807
74	1	0	6.589119	−1.564329	−0.892076
75	1	0	6.892750	−0.537714	1.966947
76	1	0	8.031963	−1.658419	1.210128
77	1	0	6.355917	−2.129792	1.432093
78	1	0	7.484251	1.625779	0.716278
79	1	0	8.942208	0.636725	0.944316
80	1	0	8.865396	1.829715	−0.362203
81	1	0	8.337104	−1.217934	−2.189571
82	1	0	9.375407	−1.210530	−0.745658
83	1	0	9.437883	0.133542	−1.899672
84	1	0	6.641372	1.656933	−1.769470
85	1	0	−6.533211	1.303133	−2.655143
86	1	0	−7.417980	3.288911	−1.483278
87	1	0	−7.862408	3.189109	0.941940
88	1	0	−7.432747	1.103229	2.186117
89	1	0	−6.537664	−0.865668	1.028923

90	1	0	-1.872603	-3.408549	2.203151
91	1	0	-0.701435	3.839581	3.392373
92	1	0	0.103782	2.252593	3.506388
93	1	0	0.864039	3.555977	2.595583

Cartesian coordinates of **8b-4**

Center Number	Atomic Number	Atomic Type	Coordinates (Angstroms) X	Y	Z
1	6	0	-3.154725	-1.254635	1.759880
2	6	0	-3.569393	-1.010049	0.297231
3	6	0	-2.349130	-0.666549	-0.635194
4	6	0	-1.662784	0.570305	0.070105
5	6	0	-1.199544	0.235768	1.470180
6	6	0	-2.384568	-0.073567	2.361947
7	6	0	-0.660289	1.394504	-0.756831
8	6	0	0.675430	0.797834	-1.181930
9	6	0	1.625236	0.116889	-0.148728
10	6	0	1.377113	0.327953	1.352980
11	6	0	3.111354	0.432411	-0.491097
12	6	0	4.081825	-0.325314	0.496191
13	6	0	3.886584	0.310783	1.885130
14	6	0	2.417551	0.401739	2.218962
15	6	0	-2.889441	-0.266668	-2.021399
16	6	0	-1.407070	-1.877546	-0.787760
17	7	0	-4.374008	-2.157873	-0.142632
18	6	0	0.047436	0.199260	1.970555
19	8	0	-4.297231	-1.461854	2.592277
20	6	0	3.604013	-0.080144	-1.859126
21	6	0	5.150481	-0.131390	-1.739880
22	6	0	5.476561	-0.140542	-0.206669
23	6	0	3.738225	-1.831880	0.613140
24	6	0	3.331348	1.974464	-0.410406
25	6	0	6.610334	-1.134324	0.166201
26	6	0	7.062444	-0.977955	1.633602
27	7	0	7.719133	-1.064228	-0.806248
28	6	0	8.510629	0.158848	-0.783972
29	6	0	8.564047	-2.247677	-0.802252
30	8	0	5.790311	0.941516	-2.438387
31	6	0	-5.518632	-2.150355	-0.933947
32	6	0	-6.493338	-1.014340	-0.820088
33	8	0	-5.742467	-3.098020	-1.679598

34	6	0	−7.243292	−0.709723	−1.965991
35	6	0	−8.208427	0.293005	−1.932215
36	6	0	−8.455010	0.986327	−0.744257
37	6	0	−7.732715	0.670496	0.407177
38	6	0	−6.752301	−0.322337	0.373085
39	1	0	−2.496493	1.278563	0.195124
40	8	0	−0.332322	2.600460	−0.000055
41	8	0	−1.998325	−0.375446	3.689377
42	1	0	5.845656	0.861174	0.046807
43	1	0	1.485628	−0.962354	−0.300577
44	6	0	−1.185563	3.645938	−0.089155
45	8	0	−2.206334	3.638584	−0.745410
46	6	0	−0.690815	4.808178	0.739952
47	1	0	−2.498253	−2.136660	1.818651
48	1	0	−4.231991	−0.141705	0.283739
49	1	0	−3.070279	0.794696	2.360816
50	1	0	−1.175115	1.731030	−1.659730
51	1	0	0.473673	0.076331	−1.981071
52	1	0	1.195879	1.623424	−1.673264
53	1	0	4.378400	−0.280697	2.668010
54	1	0	4.358798	1.303357	1.934487
55	1	0	2.181115	0.471605	3.280141
56	1	0	−3.487818	−1.074568	−2.455240
57	1	0	−3.516708	0.630561	−1.964569
58	1	0	−2.073522	−0.062828	−2.723017
59	1	0	−1.945557	−2.748993	−1.179277
60	1	0	−0.611195	−1.667808	−1.507678
61	1	0	−0.933091	−2.161498	0.156257
62	1	0	−3.842087	−3.000493	−0.342089
63	1	0	0.093536	0.000801	3.038714
64	1	0	−4.787426	−2.205555	2.201395
65	1	0	3.309465	0.551186	−2.704445
66	1	0	3.204189	−1.081421	−2.060424
67	1	0	5.560893	−1.024455	−2.213926
68	1	0	2.745348	−1.988976	1.040871
69	1	0	3.781657	−2.356180	−0.347018
70	1	0	4.445955	−2.327998	1.284825
71	1	0	2.769236	2.418827	0.415240
72	1	0	4.381230	2.245528	−0.267627
73	1	0	2.999528	2.467740	−1.329708
74	1	0	6.214613	−2.149565	0.045167
75	1	0	7.335724	0.058891	1.861455
76	1	0	7.933955	−1.607513	1.844881
77	1	0	6.273886	−1.272256	2.332395

78	1	0	9.102635	0.296474	0.140632
79	1	0	9.212722	0.136406	-1.624990
80	1	0	7.863835	1.026200	-0.929330
81	1	0	9.242200	-2.208233	-1.662713
82	1	0	7.946632	-3.147823	-0.901636
83	1	0	9.189824	-2.363707	0.104541
84	1	0	5.397888	1.769356	-2.120065
85	1	0	-7.059113	-1.276584	-2.872697
86	1	0	-8.774207	0.529370	-2.829218
87	1	0	-9.212298	1.765218	-0.715279
88	1	0	-7.932933	1.194561	1.337674
89	1	0	-6.204903	-0.563380	1.278976
90	1	0	-2.790593	-0.747218	4.112230
91	1	0	-0.633402	4.514377	1.793326
92	1	0	0.318177	5.094891	0.426691
93	1	0	-1.371665	5.652420	0.626535

Cartesian coordinates of **8b-5**

Center Number	Atomic Number	Atomic Type	Coordinates (Angstroms) X	Y	Z
1	6	0	-3.286537	-1.360746	1.057906
2	6	0	-3.570715	-0.829215	-0.364678
3	6	0	-2.273653	-0.339777	-1.108257
4	6	0	-1.607528	0.701003	-0.125574
5	6	0	-1.256768	0.074400	1.203345
6	6	0	-2.516630	-0.348048	1.927144
7	6	0	-0.526805	1.631923	-0.702912
8	6	0	0.823471	1.080799	-1.143448
9	6	0	1.683187	0.174585	-0.209040
10	6	0	1.323045	0.061693	1.281347
11	6	0	3.197334	0.510600	-0.360754
12	6	0	4.066798	-0.498910	0.487141
13	6	0	3.781187	-0.195677	1.968985
14	6	0	2.294350	-0.112402	2.211446
15	6	0	-2.698492	0.355253	-2.416264
16	6	0	-1.353745	-1.532232	-1.440137
17	7	0	-4.319219	-1.812480	-1.151724
18	6	0	-0.052802	-0.125266	1.766474
19	8	0	-4.480421	-1.763628	1.735044
20	6	0	3.790512	0.314001	-1.770979
21	6	0	5.318189	0.166209	-1.541902

22	6	0	5.515933	−0.210831	−0.045251
23	6	0	3.683478	−1.976970	0.223428
24	6	0	3.452567	1.979463	0.081817
25	6	0	6.599919	−1.303459	0.160746
26	6	0	6.931592	−1.522251	1.651574
27	7	0	7.787009	−1.031472	−0.676943
28	6	0	8.601946	0.107741	−0.271111
29	6	0	8.604719	−2.210918	−0.914993
30	8	0	6.022065	1.396356	−1.769695
31	6	0	−5.668525	−2.101670	−1.023353
32	6	0	−6.617399	−0.988209	−0.670584
33	8	0	−6.088025	−3.230513	−1.245380
34	6	0	−7.664214	−1.283243	0.214888
35	6	0	−8.607352	−0.309372	0.539068
36	6	0	−8.532442	0.959597	−0.041652
37	6	0	−7.510335	1.250933	−0.946788
38	6	0	−6.552377	0.284053	−1.256219
39	1	0	−2.428805	1.403821	0.084666
40	8	0	−0.234136	2.656523	0.296129
41	8	0	−2.261031	−0.913252	3.196605
42	1	0	5.877149	0.695222	0.453574
43	1	0	1.541497	−0.842995	−0.599505
44	6	0	−1.053954	3.729521	0.347270
45	8	0	−2.024114	3.881953	−0.366994
46	6	0	−0.594549	4.700033	1.410181
47	1	0	−2.685152	−2.274759	1.006646
48	1	0	−4.217650	0.047873	−0.257843
49	1	0	−3.167540	0.543577	2.041959
50	1	0	−0.964204	2.154662	−1.556788
51	1	0	0.661499	0.539500	−2.082285
52	1	0	1.398325	1.965693	−1.425827
53	1	0	4.191361	−0.976206	2.622979
54	1	0	4.277669	0.735085	2.281680
55	1	0	1.978148	−0.266630	3.242500
56	1	0	−3.339134	−0.305958	−3.007803
57	1	0	−3.250193	1.281643	−2.214582
58	1	0	−1.830538	0.612899	−3.032576
59	1	0	−0.487649	−1.207837	−2.022452
60	1	0	−0.977700	−2.031530	−0.542492
61	1	0	−1.875516	−2.272478	−2.057494
62	1	0	−3.817461	−2.677010	−1.332476
63	1	0	−0.096319	−0.538558	2.771483
64	1	0	−5.116769	−1.028677	1.695946
65	1	0	3.580623	1.148487	−2.449531

66	1	0	3.387943	−0.592616	−2.240734
67	1	0	5.745977	−0.611778	−2.184774
68	1	0	4.334694	−2.642385	0.799243
69	1	0	2.661903	−2.194537	0.543495
70	1	0	3.778573	−2.262324	−0.829450
71	1	0	3.035691	2.678132	−0.649724
72	1	0	2.985391	2.202486	1.044156
73	1	0	4.516429	2.212202	0.139136
74	1	0	6.205222	−2.246199	−0.236157
75	1	0	7.778358	−2.207830	1.768256
76	1	0	6.086520	−1.956876	2.193768
77	1	0	7.192773	−0.580298	2.147325
78	1	0	9.130617	−0.037548	0.689706
79	1	0	9.363571	0.285046	−1.039608
80	1	0	7.986552	1.007097	−0.209817
81	1	0	7.980135	−3.029072	−1.291781
82	1	0	9.148399	−2.581870	−0.023975
83	1	0	9.355642	−1.980311	−1.680047
84	1	0	6.113437	1.509827	−2.728025
85	1	0	−7.726885	−2.283051	0.633094
86	1	0	−9.407974	−0.543325	1.235534
87	1	0	−9.273017	1.715793	0.204457
88	1	0	−7.458775	2.229962	−1.415229
89	1	0	−5.769587	0.509225	−1.974405
90	1	0	−3.069571	−1.406264	3.422280
91	1	0	−1.253363	5.569019	1.419728
92	1	0	−0.606333	4.211398	2.390032
93	1	0	0.436361	5.012760	1.215561

Myribuxine I (**9**)

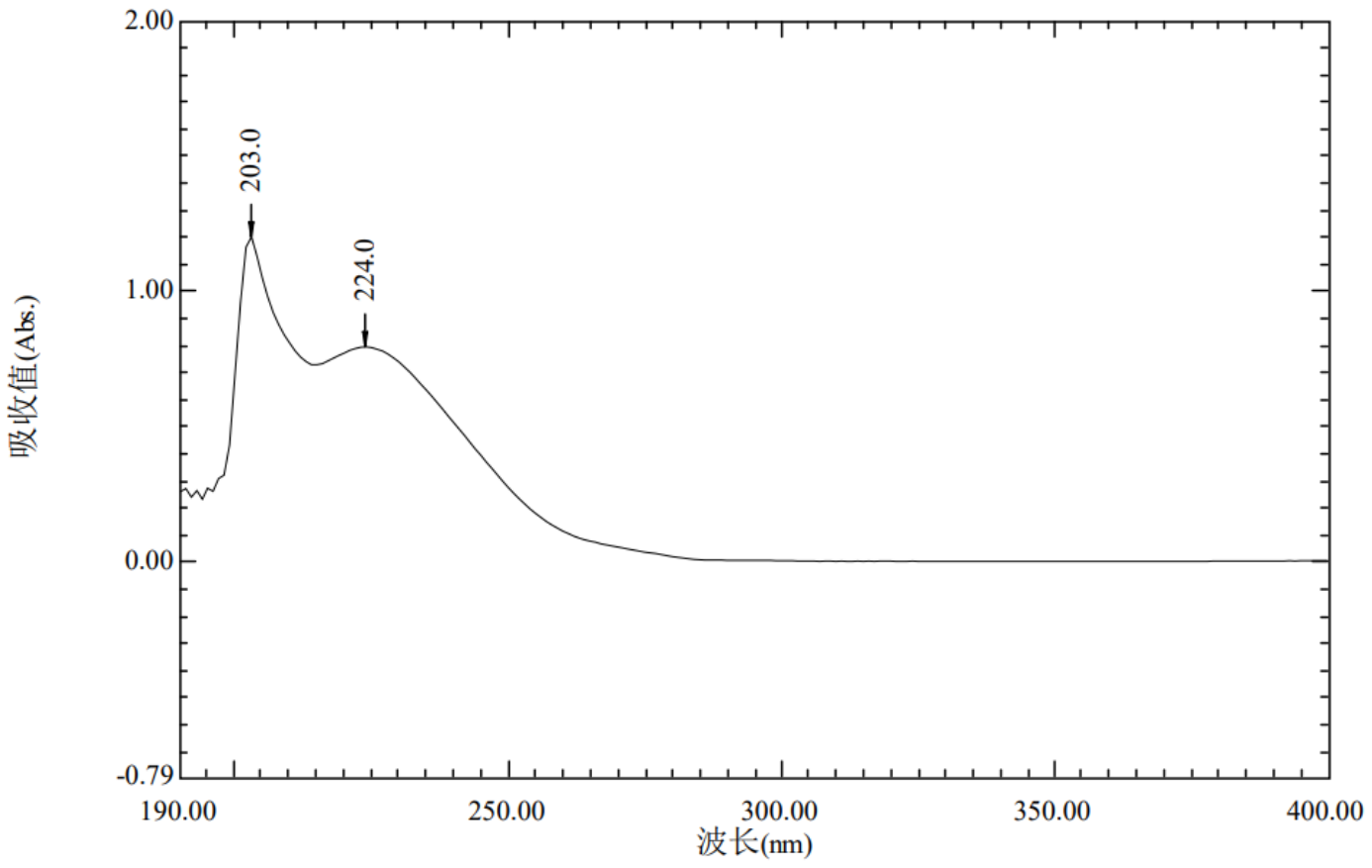

Figure S9.1 UV spectrum of compound **9**

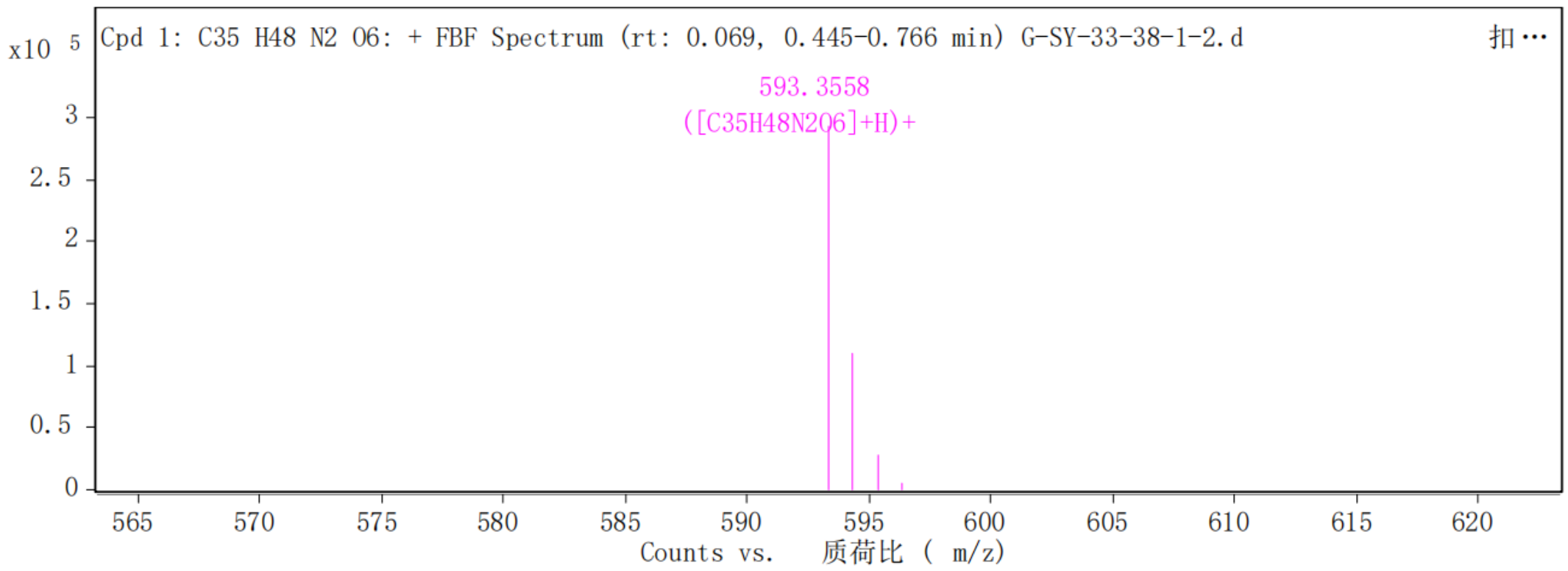

Figure S9.2 HR-ESI-MS spectrum of compound **9**

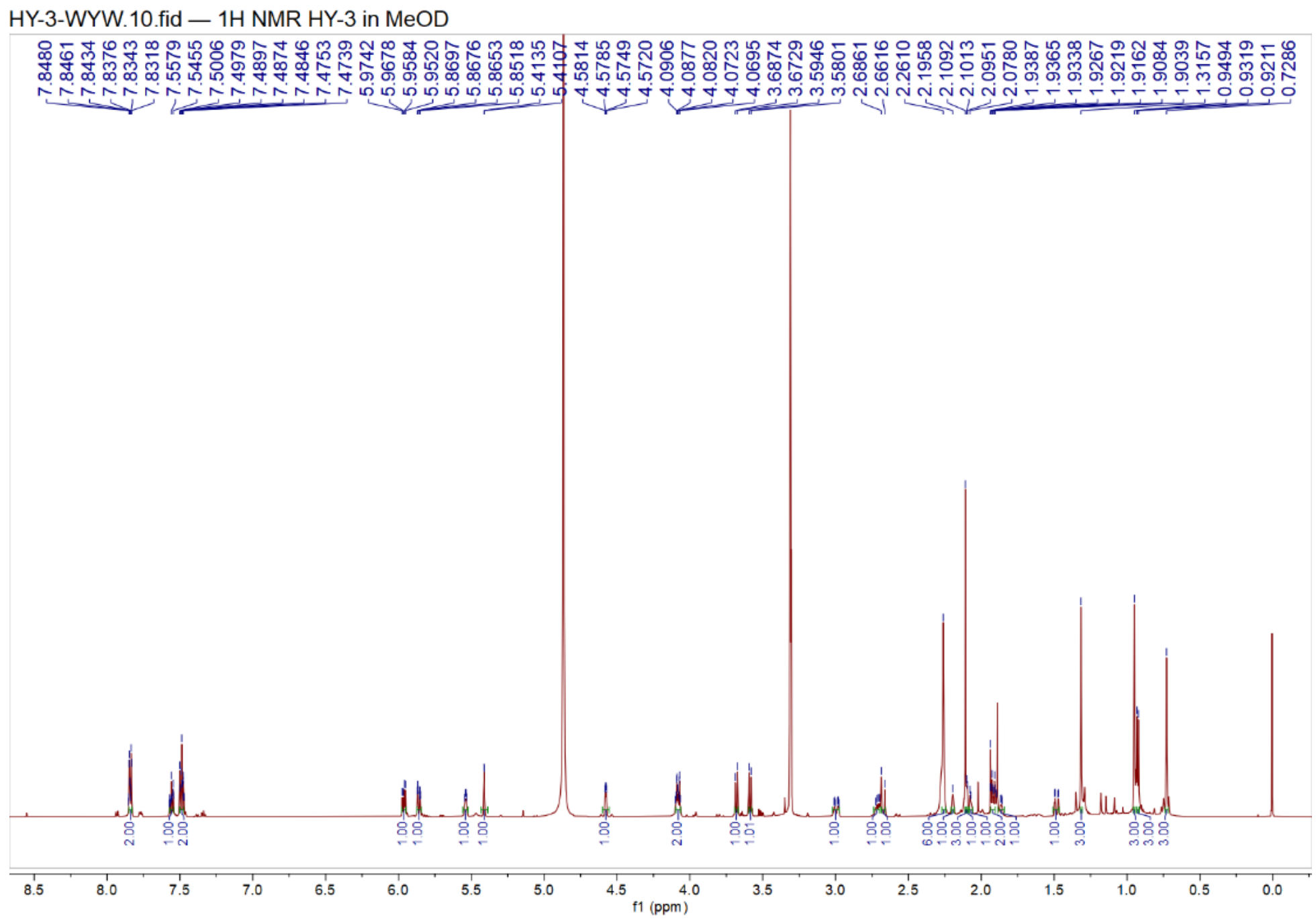

Figure S9.3 ^{1}H NMR spectrum of compound **9** (600 MHz, MeOD)

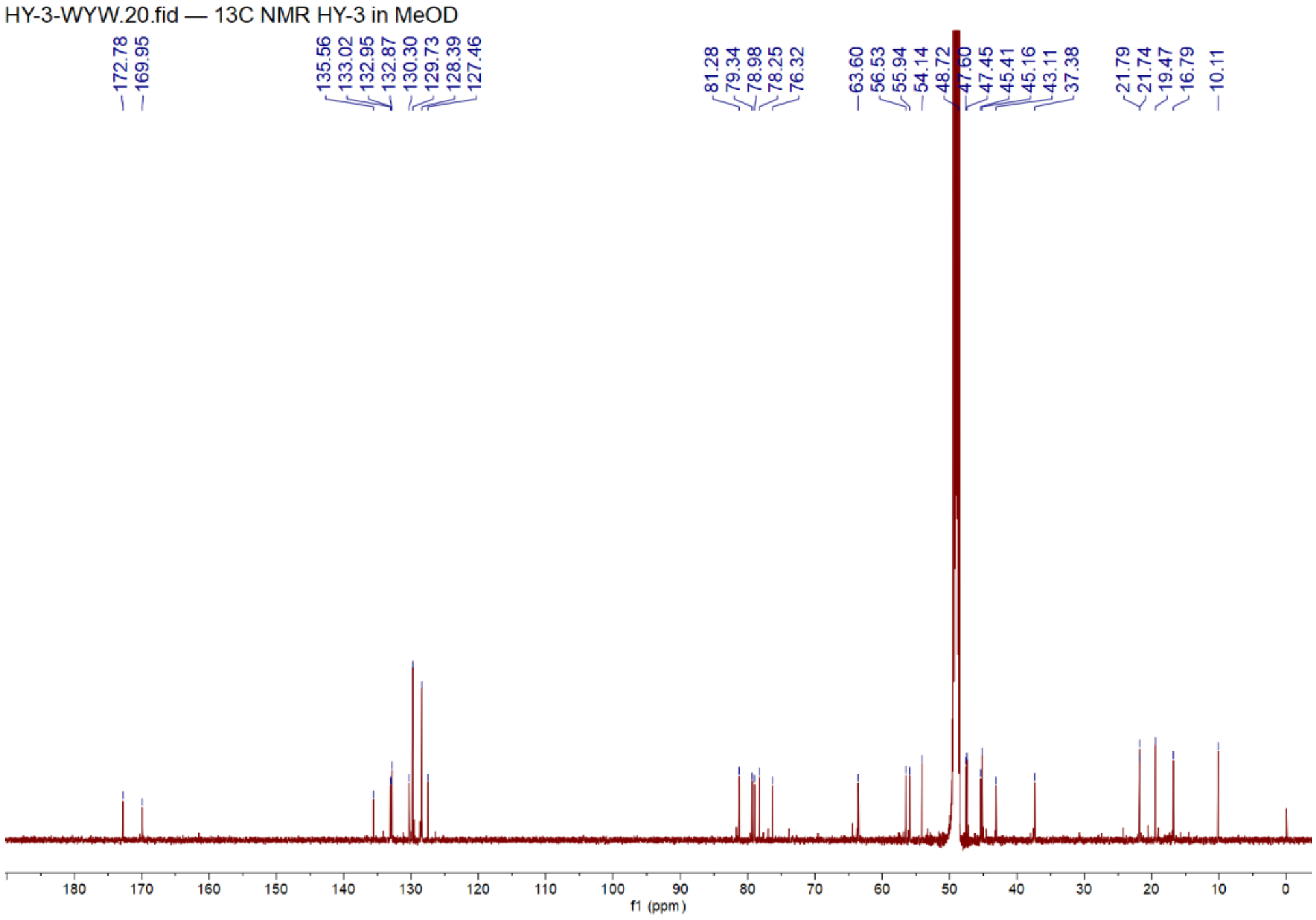

Figure S9.4 ^{13}C NMR spectrum of compound **9** (150 MHz, MeOD)

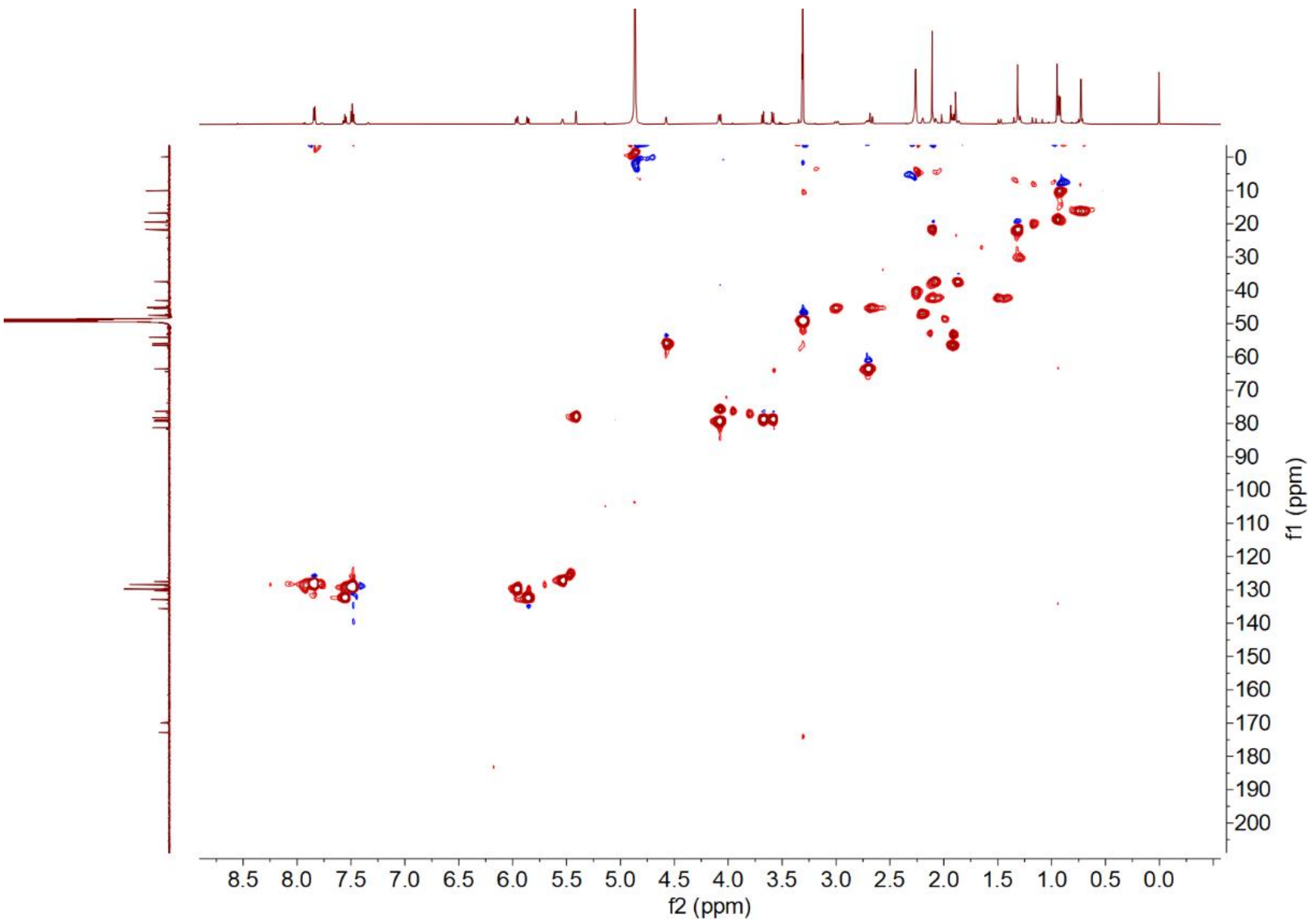

Figure S9.5 HSQC spectrum of compound **9**

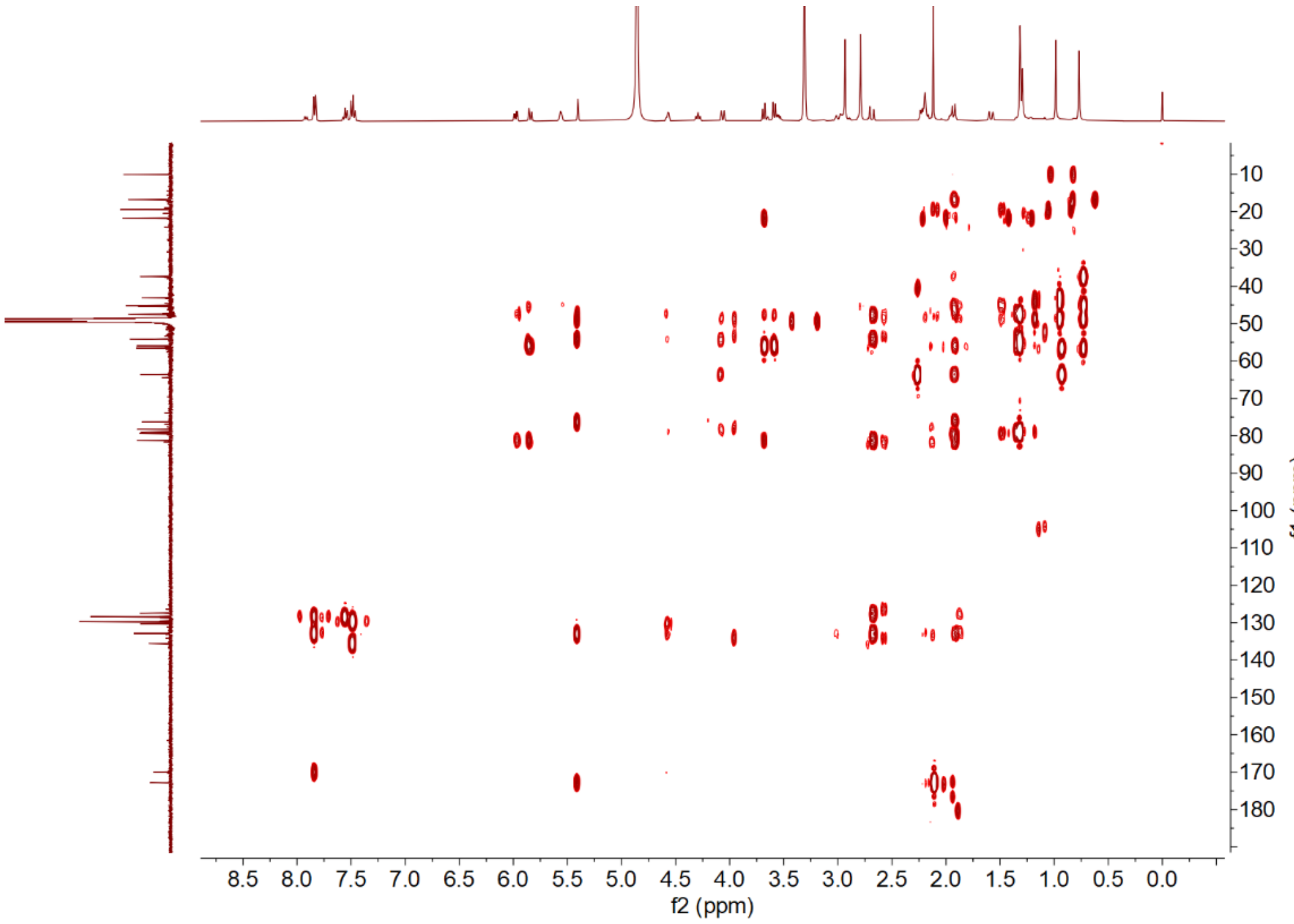

Figure S9.6 HMBC spectrum of compound **9**

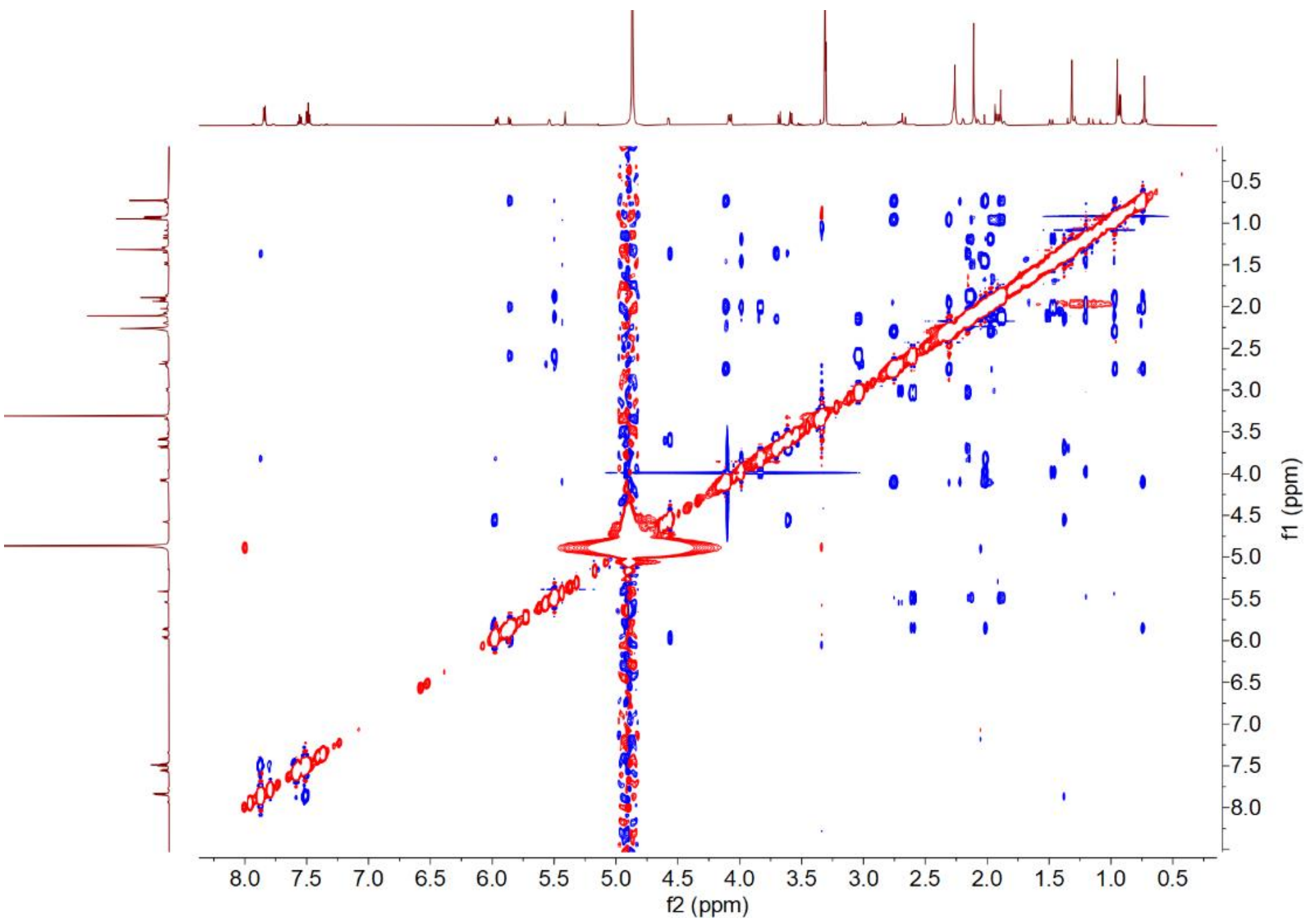

Figure S9.7 NOESY spectrum of compound **9**

Table S7 Experimental/calculated NMR shifts and statistical parameters for **9**

Position / Parameter	Experimental	Isomer 9a	Isomer 9b
C	132.9	141.4275	143.3326
C	130.3	142.7415	139.5166
C	55.9	64.64766	62.8617
C	47.5	54.32722	52.56163

C	54.1	56.56747	52.65463
C	76.3	81.55073	83.04445
C	79	89.70063	92.30312
C	47.6	53.74194	58.93107
C	135.6	143.4762	142.3591
C	81.3	86.0	86.3
C	127.5	137.6	136.4
C	37.4	42.51	41.12
C	45.4	50.40	51.04
C	48.7	54.73	54.16
C	43.1	45.81	45.42
C	79.3	82.98	83.57
C	56.5	61.60	61.05
C	16.8	18.57	18.13
C	45.2	48.97	43.54
C	63.6	66.42	67.79
C	10.1	12.14	12.55
C	19.5	21.80	22.95
C	78.3	82.13	82.86
C	21.8	24.06	24.18
C	133	142.18	142.70
C	128.4	136.9672	136.9336
C	128.4	135.2126	136.7729
C	129.7	136.2527	135.3928
C	129.7	135.1231	135.2874
C	132.9	139.1878	139.4065
C	172.8	182.0352	182.0989
C	169.9	186.1927	185.3907
C	21.7	25.81501	25.0989636544
C	40.1	46.59912	46.3393
C	40.1	36.6151	37.38326
**^{13}C MAE (unscaled)**		**6.074**	**6.076**
**^{13}C CMAE**		**1.822**	**1.992**
**^{13}C R^2**		**0.9974**	**0.9964**
**^{13}C sDP4$^+$ Probability**		**99.07%**	**0.93%**
**^{13}C uDP4$^+$ Probability**		**99.99%**	**0.01%**
**^{13}C DP4$^+$ Probability**		**100%**	**0%**
H	5.86	6.334361	6.088986
H	5.96	6.595472	6.241238
H	4.58	3.892266	3.981857
H	1.93	1.673178	2.162454
H	4.08	4.060196	3.867565
H	5.41	4.874358	4.760352

H	2.2	2.278327	2.934817
H	5.54	6.053718	5.944272
H	2.1	2.4428	2.534892
H	1.86	2.057291	2.11863
H	2.07	2.260223	2.062695
H	1.48	1.57474	1.464092
H	4.08	4.370288	4.455434
H	1.93	1.859914	2.15103
H	0.73	0.826125	0.876068
H	2.99	3.053291	2.762608
H	2.67	2.8487	2.354942
H	2.71	2.678372	2.805074
H	0.93	1.022021	1.073353
H	0.95	1.295627	1.393111
H	3.68	3.695673	3.721276
H	3.59	3.30228	3.456231
H	1.32	1.311416	1.476591
H	7.84	8.190366	8.122303
H	7.84	8.123409	8.113779
H	7.49	7.937075	7.958661
H	7.49	7.923558	7.957916
H	7.55	8.106266	8.128937
H	2.11	2.418593	2.31507
H	2.26	2.577961	2.498152
H	2.26	2.140712	2.267976
**^{1}H MAE (unscaled)**		**0.268**	**0.286**
**^{1}H sDP4^{+} Probability**		**98.20%**	**1.80%**
**^{1}H uDP4^{+} Probability**		**99.42%**	**0.58%**
**^{1}H DP4^{+} Probability**		**99.99%**	**0.01%**
**sDP4^{+} Probability (All Data)**		**99.98%**	**0.02%**
**uDP4^{+} Probability (All Data)**		**100%**	**0.00%**
**DP4^{+} Probability (All Data)**		**100%**	**0%**

Table S8 Relative energies and Boltzmann populations for **9a**

Conformer	Energy (Hartree)	Δ*G* (kcal/mol)	Population (%)
9a-1	−1922.703071	0.00	72.73
9a-2	−1922.702132	0.59	26.90
9a-3	−1922.697345	3.59	0.17
9a-4	−1922.696430	4.17	0.06

Conformer	Energy (Hartree)	ΔG (kcal/mol)	Population (%)
9a-5	−1922.696433	4.17	0.06
9a-6	−1922.696440	4.16	0.06
9a-7	−1922.691120	7.50	0.00

Cartesian coordinates of **9a-1**

Center Number	Atomic Number	Atomic Type	Coordinates (Angstroms) X	Y	Z
1	6	0	-2.566617	-1.086836	1.239485
2	6	0	-3.605282	-0.304724	0.451572
3	6	0	-3.363701	1.232783	0.612820
4	6	0	-1.845453	1.564082	0.506110
5	6	0	-1.373214	1.009157	1.868106
6	6	0	-1.552875	-0.502641	1.887835
7	6	0	-1.052889	1.043952	-0.700533
8	6	0	0.437871	1.474712	-0.681308
9	6	0	1.313286	0.569830	0.219563
10	6	0	1.233562	0.847193	1.728320
11	6	0	2.795878	0.443889	-0.231791
12	6	0	3.505860	-0.638189	0.683145
13	6	0	3.582369	-0.075760	2.112472
14	6	0	2.258390	0.518964	2.534998
15	6	0	3.023528	-0.128516	-1.652863
16	6	0	4.378089	-0.878589	-1.588024
17	6	0	4.836700	-0.853801	-0.096693
18	6	0	0.001907	1.487697	2.347875
19	6	0	2.716001	-1.971582	0.742551
20	6	0	3.499157	1.823584	-0.168265
21	6	0	-4.282793	2.051939	-0.299005
22	7	0	-3.678737	-0.743129	-0.952194
23	6	0	-4.811874	-1.153087	-1.626792
24	6	0	5.756084	-2.055223	0.218076
25	7	0	6.893507	-2.044839	-0.753747
26	8	0	5.327724	-0.277476	-2.455638
27	6	0	-3.598184	1.607664	2.110579
28	8	0	-4.907791	-1.000699	-2.840852
29	6	0	-5.916333	-1.835636	-0.863917
30	6	0	-7.232539	-1.593864	-1.282650
31	6	0	-8.301091	-2.233522	-0.659101

32	6	0	−8.066027	−3.146311	0.372869
33	6	0	−6.757424	−3.416600	0.775389
34	6	0	−5.687795	−2.761193	0.163272
35	8	0	−1.675784	1.319390	−1.947632
36	6	0	6.206394	−2.150639	1.686420
37	6	0	7.977068	−1.117564	−0.419840
38	6	0	7.422334	−3.380564	−1.019319
39	8	0	−2.331068	1.600717	2.775899
40	1	0	5.442001	0.049639	0.034216
41	1	0	−1.746880	2.656402	0.551420
42	1	0	0.873653	−0.424582	0.060428
43	8	0	0.648995	2.862264	−0.261058
44	6	0	0.276251	3.874936	−1.060860
45	8	0	−0.389343	3.747680	−2.074037
46	6	0	0.794766	5.194782	−0.546166
47	1	0	−2.690417	−2.167929	1.268345
48	1	0	−4.587932	−0.519893	0.885213
49	1	0	−0.854618	−1.094193	2.476981
50	1	0	−1.029879	−0.047699	−0.653990
51	1	0	0.787106	1.393530	−1.715596
52	1	0	3.861769	−0.857509	2.830780
53	1	0	4.371315	0.687595	2.191445
54	1	0	2.146992	0.704668	3.603486
55	1	0	3.075384	0.649302	−2.420640
56	1	0	2.219669	−0.815281	−1.943351
57	1	0	4.229642	−1.927845	−1.890919
58	1	0	0.033413	1.327558	3.431647
59	1	0	0.027434	2.573361	2.194785
60	1	0	3.307683	−2.732635	1.262472
61	1	0	1.784331	−1.866246	1.304930
62	1	0	2.471736	−2.371140	−0.246767
63	1	0	3.437936	2.284829	0.820639
64	1	0	4.547844	1.758831	−0.466679
65	1	0	3.021851	2.506268	−0.876405
66	1	0	−4.111418	3.123718	−0.142981
67	1	0	−4.115246	1.833067	−1.354432
68	1	0	−5.336789	1.846842	−0.070085
69	1	0	−3.046205	−0.264733	−1.592743
70	1	0	5.201153	−2.970699	−0.020568
71	1	0	6.123416	−0.829754	−2.317686
72	1	0	−4.017878	2.616758	2.202093
73	1	0	−4.287031	0.907627	2.603255
74	1	0	−7.396641	−0.906175	−2.106085
75	1	0	−9.317811	−2.028928	−0.983827

76	1	0	-8.899224	-3.651741	0.854046
77	1	0	-6.566990	-4.141693	1.562218
78	1	0	-4.671174	-2.993841	0.464659
79	1	0	-1.442656	2.234932	-2.202536
80	1	0	6.966744	-2.929577	1.815876
81	1	0	5.366122	-2.407842	2.337560
82	1	0	6.627312	-1.205834	2.047359
83	1	0	8.553840	-1.411714	0.473887
84	1	0	8.674229	-1.069703	-1.263752
85	1	0	7.581259	-0.111084	-0.259165
86	1	0	7.903778	-3.850865	-0.142398
87	1	0	8.172381	-3.321061	-1.815764
88	1	0	6.616557	-4.037798	-1.361611
89	1	0	0.550205	5.312784	0.513784
90	1	0	1.886528	5.219903	-0.633854
91	1	0	0.361820	6.009218	-1.127536

Cartesian coordinates of **9a-2**

Center Number	Atomic Number	Atomic Type	Coordinates (Angstroms) X	Y	Z
1	6	0	-2.816828	-0.724845	1.158081
2	6	0	-3.782962	0.082316	0.301370
3	6	0	-3.470249	1.604109	0.413765
4	6	0	-1.933374	1.858228	0.345902
5	6	0	-1.530887	1.329076	1.740207
6	6	0	-1.790850	-0.170017	1.812265
7	6	0	-1.130654	1.264510	-0.819056
8	6	0	0.378355	1.619874	-0.764838
9	6	0	1.177410	0.700281	0.189371
10	6	0	1.063543	1.022894	1.686877
11	6	0	2.665149	0.486436	-0.206608
12	6	0	3.281914	-0.612863	0.754704
13	6	0	3.340383	-0.023884	2.174481
14	6	0	2.040179	0.657771	2.536359
15	6	0	2.909637	-0.127558	-1.607101
16	6	0	4.219085	-0.945666	-1.479867
17	6	0	4.624803	-0.917698	0.026628
18	6	0	-0.147213	1.753178	2.244864
19	6	0	2.418176	-1.899698	0.813706
20	6	0	3.440156	1.827067	-0.145309
21	6	0	-4.318752	2.438910	-0.551440

22	7	0	−3.817463	−0.374060	−1.101366
23	6	0	−4.274553	−1.586828	−1.563935
24	6	0	5.463527	−2.162174	0.393979
25	7	0	6.637676	−2.226734	−0.531648
26	8	0	5.229960	−0.410043	−2.320919
27	6	0	−3.729172	2.043787	1.890807
28	8	0	−3.851198	−2.070305	−2.609784
29	6	0	−5.348301	−2.299577	−0.783274
30	6	0	−6.498692	−1.650497	−0.314297
31	6	0	−7.505597	−2.374395	0.325949
32	6	0	−7.366895	−3.750782	0.513188
33	6	0	−6.228613	−4.405966	0.036381
34	6	0	−5.233101	−3.686821	−0.622384
35	8	0	−1.702509	1.540582	−2.091212
36	6	0	5.850189	−2.259442	1.880170
37	6	0	7.760340	−1.363741	−0.157352
38	6	0	7.096299	−3.593511	−0.769408
39	8	0	−2.483984	2.002698	2.594611
40	1	0	5.276360	−0.047940	0.164515
41	1	0	−1.784359	2.945766	0.359566
42	1	0	0.694252	−0.275023	0.040938
43	8	0	0.648270	3.008418	−0.381097
44	6	0	0.358653	4.011529	−1.225634
45	8	0	−0.278261	3.885865	−2.257583
46	6	0	0.929973	5.318892	−0.735575
47	1	0	−3.016868	−1.792503	1.232531
48	1	0	−4.788277	−0.070252	0.706566
49	1	0	−1.145173	−0.771574	2.449425
50	1	0	−1.165159	0.174917	−0.744122
51	1	0	0.754425	1.488010	−1.784333
52	1	0	3.546562	−0.804734	2.918191
53	1	0	4.170023	0.693388	2.268270
54	1	0	1.905249	0.878958	3.595339
55	1	0	3.029229	0.629803	−2.387784
56	1	0	2.080660	−0.776997	−1.912247
57	1	0	4.026598	−1.991034	−1.771212
58	1	0	−0.155510	1.633821	3.334260
59	1	0	−0.057694	2.828939	2.051000
60	1	0	2.949346	−2.680404	1.368483
61	1	0	1.475799	−1.731865	1.342133
62	1	0	2.184451	−2.306399	−0.175239
63	1	0	3.023448	2.519275	−0.881891
64	1	0	3.371689	2.311614	0.831938
65	1	0	4.492934	1.699527	−0.407293

66	1	0	-4.096016	3.504958	-0.423447
67	1	0	-4.132615	2.175720	-1.592817
68	1	0	-5.388055	2.295489	-0.348150
69	1	0	-3.131636	0.041544	-1.729300
70	1	0	4.869962	-3.050428	0.145723
71	1	0	5.991600	-0.997154	-2.140850
72	1	0	-4.102531	3.074083	1.933302
73	1	0	-4.464743	1.394944	2.385779
74	1	0	-6.620881	-0.583801	-0.480956
75	1	0	-8.400332	-1.864222	0.672830
76	1	0	-8.147832	-4.312851	1.018519
77	1	0	-6.123394	-5.479675	0.167372
78	1	0	-4.360909	-4.189149	-1.029512
79	1	0	-1.403005	2.431086	-2.365583
80	1	0	6.310656	-1.334565	2.244401
81	1	0	6.558740	-3.078387	2.050788
82	1	0	4.972108	-2.458213	2.501393
83	1	0	7.419538	-0.334333	-0.016952
84	1	0	8.279744	-1.686813	0.761021
85	1	0	8.493364	-1.363227	-0.971656
86	1	0	7.511141	-4.086367	0.128969
87	1	0	7.881521	-3.583663	-1.533441
88	1	0	6.267800	-4.203572	-1.143298
89	1	0	0.655720	5.485681	0.310470
90	1	0	2.023864	5.283320	-0.784342
91	1	0	0.562019	6.134787	-1.358195

Cartesian coordinates of **9a-3**

Center Number	Atomic Number	Atomic Type	Coordinates (Angstroms) X	Y	Z
1	6	0	-2.541513	-1.079074	1.237351
2	6	0	-3.585119	-0.304109	0.448624
3	6	0	-3.357850	1.235126	0.615386
4	6	0	-1.842288	1.579835	0.512878
5	6	0	-1.367841	1.025512	1.874747
6	6	0	-1.534640	-0.487787	1.890020
7	6	0	-1.045144	1.069756	-0.694856
8	6	0	0.443853	1.502384	-0.668578
9	6	0	1.316599	0.598370	0.235503
10	6	0	1.241354	0.889083	1.741529
11	6	0	2.797716	0.469617	-0.218436

12	6	0	3.522998	−0.591756	0.705130
13	6	0	3.607413	0.007518	2.120041
14	6	0	2.276421	0.586067	2.544959
15	6	0	3.027297	−0.139193	−1.617090
16	6	0	4.478914	−0.690875	−1.572220
17	6	0	4.866527	−0.826748	−0.070920
18	6	0	0.001529	1.515286	2.360036
19	6	0	2.732425	−1.921460	0.810957
20	6	0	3.492849	1.855303	−0.177149
21	6	0	−4.282482	2.048899	−0.295681
22	7	0	−3.650236	−0.738455	−0.957028
23	6	0	−4.774472	−1.164637	−1.636278
24	6	0	5.665229	−2.125341	0.225034
25	7	0	6.698392	−2.352054	−0.806466
26	8	0	5.417563	0.209285	−2.177569
27	6	0	−3.599073	1.602533	2.113895
28	8	0	−4.867542	−1.011540	−2.850599
29	6	0	−5.871343	−1.865272	−0.879173
30	6	0	−5.633062	−2.787744	0.148581
31	6	0	−6.694981	−3.460986	0.754723
32	6	0	−8.005713	−3.211774	0.345640
33	6	0	−8.250328	−2.302121	−0.686880
34	6	0	−7.189179	−1.644656	−1.304498
35	8	0	−1.665634	1.354963	−1.941475
36	6	0	6.210398	−2.162578	1.667939
37	6	0	7.835274	−1.438926	−0.777518
38	6	0	7.134007	−3.737693	−0.888440
39	8	0	−2.333382	1.606078	2.781595
40	1	0	5.530005	0.016574	0.151269
41	1	0	−1.752151	2.672864	0.560741
42	1	0	0.873133	−0.395434	0.084101
43	8	0	0.648959	2.889727	−0.246283
44	6	0	0.297507	3.899936	−1.058158
45	8	0	−0.344664	3.769204	−2.086263
46	6	0	0.805724	5.220782	−0.536127
47	1	0	−2.655844	−2.161254	1.263684
48	1	0	−4.567171	−0.529681	0.878368
49	1	0	−0.833482	−1.074871	2.480215
50	1	0	−1.020108	−0.022219	−0.656580
51	1	0	0.797076	1.423839	−1.701554
52	1	0	3.912044	−0.749018	2.854384
53	1	0	4.381603	0.788001	2.167507
54	1	0	2.168454	0.781211	3.612137
55	1	0	2.922960	0.589190	−2.429116

56	1	0	2.314968	−0.948977	−1.818298
57	1	0	4.549317	−1.665609	−2.068581
58	1	0	0.031416	1.351871	3.443376
59	1	0	0.016555	2.601756	2.211012
60	1	0	2.542549	−2.388636	−0.160715
61	1	0	3.294297	−2.642877	1.412448
62	1	0	1.773005	−1.784599	1.315731
63	1	0	4.558574	1.786770	−0.400046
64	1	0	3.060082	2.502373	−0.946219
65	1	0	3.358893	2.357616	0.783915
66	1	0	−4.111661	1.834415	−1.351478
67	1	0	−5.335018	1.834423	−0.068624
68	1	0	−4.120272	3.121655	−0.136566
69	1	0	−3.023137	−0.250252	−1.595176
70	1	0	4.980219	−2.973042	0.105193
71	1	0	5.350156	0.103679	−3.138788
72	1	0	−4.029160	2.606998	2.208041
73	1	0	−4.281849	0.893877	2.602702
74	1	0	−4.614450	−3.004364	0.454969
75	1	0	−6.496762	−4.183488	1.541997
76	1	0	−8.833011	−3.731131	0.822130
77	1	0	−9.268562	−2.113908	−1.016711
78	1	0	−7.360268	−0.959096	−2.128276
79	1	0	−1.420888	2.267286	−2.196762
80	1	0	6.783654	−1.259082	1.905056
81	1	0	6.869244	−3.025531	1.815812
82	1	0	5.403174	−2.243479	2.402150
83	1	0	8.449231	−1.615574	−1.668394
84	1	0	7.488659	−0.404868	−0.821695
85	1	0	8.489643	−1.565315	0.105473
86	1	0	7.758800	−3.866805	−1.780153
87	1	0	6.264818	−4.397945	−0.987819
88	1	0	7.727032	−4.085272	−0.019833
89	1	0	0.404081	6.032663	−1.142942
90	1	0	0.516309	5.351532	0.511097
91	1	0	1.900330	5.235894	−0.575797

Table S9 Relative energies and Boltzmann populations for **9b**

Conformer	Energy (Hartree)	Δ*G* (kcal/mol)	Population (%)
9b-1	−1922.696300	0.21	40.89

Conformer	Energy (Hartree)	ΔG (kcal/mol)	Population (%)
9b-2	−1922.691568	3.18	0.27
9b-3	−1922.690859	3.63	0.13
9b-4	−1922.696641	0.00	58.67
9b-5	−1922.686320	6.48	0.00
9b-6	−1922.688976	4.81	0.02
9b-7	−1922.688977	4.81	0.02
9b-8	−1922.687833	5.53	0.01

Cartesian coordinates of **9b-1**

Center Number	Atomic Number	Atomic Type	Coordinates (Angstroms) X	Y	Z
1	6	0	3.663179	0.259597	1.790711
2	6	0	3.773821	-0.452629	0.455496
3	6	0	2.456347	-1.235752	0.150331
4	6	0	1.195898	-0.389404	0.491389
5	6	0	1.278330	-0.434269	2.027912
6	6	0	2.536727	0.274047	2.509534
7	6	0	0.995486	1.027155	-0.043056
8	6	0	-0.461453	1.597562	0.124389
9	6	0	-1.617301	0.854821	0.870451
10	6	0	-1.248801	-0.175467	1.942004
11	6	0	-2.672563	0.270449	-0.107735
12	6	0	-3.868887	-0.338156	0.733563
13	6	0	-3.342549	-1.554980	1.518258
14	6	0	-2.036026	-1.229660	2.207976
15	6	0	-3.393974	1.300443	-1.012371
16	6	0	-4.801177	0.705845	-1.270214
17	6	0	-4.916421	-0.580842	-0.394313
18	6	0	0.012808	0.070231	2.736570
19	6	0	-4.435272	0.673834	1.762671
20	6	0	-2.018671	-0.776877	-1.047489
21	6	0	2.472238	-1.846078	-1.254869
22	7	0	4.157441	0.474649	-0.618892
23	6	0	5.208436	0.337393	-1.500163

24	6	0	−6.392851	−0.886579	−0.057548
25	7	0	−7.156092	−0.984929	−1.341608
26	8	0	−4.985645	0.439636	−2.652401
27	6	0	2.285084	−2.350447	1.238526
28	8	0	5.155414	0.834601	−2.622089
29	6	0	6.450900	−0.389402	−1.057367
30	6	0	6.993303	−0.278932	0.230127
31	6	0	8.192035	−0.918827	0.549692
32	6	0	8.855226	−1.682653	−0.411681
33	6	0	8.326525	−1.788025	−1.701186
34	6	0	7.140281	−1.133998	−2.025048
35	8	0	1.361429	1.073085	−1.422533
36	6	0	−6.604169	−2.093935	0.872473
37	6	0	−7.042201	−2.277374	−2.021511
38	6	0	−8.558357	−0.601139	−1.197514
39	8	0	1.421341	−1.848773	2.268987
40	1	0	−4.550672	−1.414905	−1.003475
41	1	0	0.335240	−0.999106	0.204928
42	1	0	−2.109759	1.680409	1.402339
43	8	0	−0.369868	2.912904	0.778002
44	6	0	0.161030	3.916866	0.056424
45	6	0	0.092076	5.227756	0.799892
46	8	0	0.627976	3.781344	−1.061636
47	1	0	4.556433	0.767097	2.150727
48	1	0	4.577903	−1.193355	0.527627
49	1	0	2.503709	0.766350	3.480213
50	1	0	1.649183	1.701759	0.524389
51	1	0	−0.811110	1.795267	−0.890614
52	1	0	−4.067599	−1.872369	2.278998
53	1	0	−3.215242	−2.426987	0.859657
54	1	0	−1.728151	−1.909309	3.001687
55	1	0	−2.886592	1.454686	−1.969579
56	1	0	−3.470322	2.279440	−0.524969
57	1	0	−5.568522	1.424067	−0.937067
58	1	0	0.125831	1.147575	2.919077
59	1	0	−0.038583	−0.415975	3.717372
60	1	0	−3.724393	0.867679	2.570231
61	1	0	−4.710398	1.633221	1.313680
62	1	0	−5.336628	0.264416	2.231882
63	1	0	−2.704866	−1.088739	−1.838582
64	1	0	−1.153319	−0.335049	−1.554318
65	1	0	−1.670807	−1.668743	−0.519799
66	1	0	3.325521	−2.527088	−1.371892
67	1	0	1.557233	−2.424883	−1.426117

68	1	0	2.535661	-1.081968	-2.030920
69	1	0	3.367178	0.938470	-1.069648
70	1	0	-6.810732	-0.004489	0.443142
71	1	0	-5.884188	0.053108	-2.679411
72	1	0	1.811841	-3.243863	0.814880
73	1	0	3.251037	-2.646451	1.669920
74	1	0	6.495776	0.333754	0.975518
75	1	0	8.609647	-0.815319	1.547744
76	1	0	9.785266	-2.185927	-0.160675
77	1	0	8.845525	-2.372361	-2.456341
78	1	0	6.733085	-1.180091	-3.029994
79	1	0	1.226105	2.004381	-1.692605
80	1	0	-6.073582	-2.983617	0.516456
81	1	0	-7.667726	-2.346315	0.955207
82	1	0	-6.244410	-1.877230	1.882215
83	1	0	-7.493551	-2.195714	-3.016566
84	1	0	-5.990865	-2.546015	-2.154738
85	1	0	-7.547495	-3.102489	-1.490977
86	1	0	-9.142075	-1.282639	-0.551895
87	1	0	-9.033827	-0.593046	-2.184559
88	1	0	-8.626477	0.408674	-0.780187
89	1	0	0.609909	5.998357	0.228327
90	1	0	-0.954127	5.516878	0.946797
91	1	0	0.544989	5.124577	1.790826

Cartesian coordinates of **9b-2**

Center Number	Atomic Number	Atomic Type	Coordinates (Angstroms) X	Y	Z
1	6	0	3.784746	-0.287787	1.232911
2	6	0	3.723301	-1.102651	-0.050766
3	6	0	2.352128	-1.828522	-0.165069
4	6	0	1.183595	-0.868488	0.211937
5	6	0	1.396283	-0.803478	1.736985
6	6	0	2.734193	-0.145663	2.046679
7	6	0	1.047848	0.499823	-0.454602
8	6	0	-0.194244	1.341879	-0.015381
9	6	0	-1.418788	0.727400	0.731799
10	6	0	-1.106363	-0.310963	1.815330
11	6	0	-2.572986	0.240103	-0.185131
12	6	0	-3.786156	-0.195848	0.739509

13	6	0	-3.366022	-1.442730	1.541584
14	6	0	-1.995501	-1.257641	2.155209
15	6	0	-3.216445	1.318563	-1.092541
16	6	0	-4.696659	0.890956	-1.251079
17	6	0	-4.914671	-0.345199	-0.323828
18	6	0	0.223421	-0.198595	2.529653
19	6	0	-4.175048	0.906925	1.759286
20	6	0	-2.103157	-0.907177	-1.116729
21	6	0	2.198106	-2.572058	-1.497325
22	7	0	3.984806	-0.290944	-1.258568
23	6	0	5.085690	0.488372	-1.528071
24	6	0	-6.395989	-0.463919	0.098999
25	7	0	-7.236265	-0.511677	-1.138307
26	8	0	-4.995703	0.604897	-2.610269
27	6	0	2.232703	-2.835043	1.028404
28	8	0	4.993094	1.483658	-2.243405
29	6	0	6.419368	0.070775	-0.971827
30	6	0	7.288764	1.075890	-0.526323
31	6	0	8.556578	0.750797	-0.048747
32	6	0	8.981747	-0.580359	-0.041038
33	6	0	8.134913	-1.583391	-0.515812
34	6	0	6.857125	-1.260799	-0.974792
35	8	0	1.045693	0.282774	-1.876894
36	6	0	-6.694296	-1.607640	1.084258
37	6	0	-7.304004	-1.826028	-1.781004
38	6	0	-8.577475	0.029887	-0.933707
39	8	0	1.484808	-2.205317	2.073165
40	1	0	-4.682328	-1.234765	-0.919471
41	1	0	0.255527	-1.421540	0.047677
42	1	0	-1.813634	1.608459	1.256400
43	8	0	0.361942	2.397642	0.835588
44	6	0	-0.074075	3.666698	0.632083
45	6	0	0.599541	4.619845	1.590835
46	8	0	-0.892618	3.977791	-0.205469
47	1	0	4.743101	0.169148	1.472228
48	1	0	4.497855	-1.875526	0.002546
49	1	0	2.823709	0.415456	2.975269
50	1	0	1.914100	1.116738	-0.193004
51	1	0	-0.583250	1.841418	-0.906311
52	1	0	-4.083271	-1.648660	2.346719
53	1	0	-3.375338	-2.343213	0.909425
54	1	0	-1.725920	-1.952257	2.950344
55	1	0	-2.747829	1.366420	-2.080246
56	1	0	-3.137779	2.318811	-0.654732

57	1	0	-5.351473	1.707230	-0.904055
58	1	0	0.444727	0.856054	2.727069
59	1	0	0.188321	-0.710702	3.497431
60	1	0	-3.416481	1.024792	2.537958
61	1	0	-4.337061	1.883493	1.292850
62	1	0	-5.104236	0.632282	2.270534
63	1	0	-1.225399	-0.585783	-1.686035
64	1	0	-1.833833	-1.820078	-0.578365
65	1	0	-2.870854	-1.157281	-1.853125
66	1	0	2.989858	-3.324310	-1.609634
67	1	0	1.234450	-3.093155	-1.527425
68	1	0	2.241437	-1.902840	-2.356698
69	1	0	3.150920	0.070848	-1.716435
70	1	0	-6.679518	0.476910	0.586642
71	1	0	-5.934591	0.331538	-2.571391
72	1	0	1.688952	-3.738257	0.728320
73	1	0	3.221336	-3.138885	1.399497
74	1	0	6.956953	2.108596	-0.569647
75	1	0	9.218543	1.535867	0.306832
76	1	0	9.973955	-0.833158	0.323270
77	1	0	8.470779	-2.616743	-0.535193
78	1	0	6.211539	-2.039894	-1.369820
79	1	0	1.007551	1.149020	-2.315759
80	1	0	-7.773807	-1.732013	1.229364
81	1	0	-6.258708	-1.400677	2.065879
82	1	0	-6.288522	-2.564208	0.737471
83	1	0	-7.863658	-2.576322	-1.196387
84	1	0	-7.802565	-1.721008	-2.751043
85	1	0	-6.298129	-2.212655	-1.965607
86	1	0	-8.510725	1.049829	-0.541802
87	1	0	-9.197872	-0.567285	-0.240292
88	1	0	-9.101965	0.070051	-1.894880
89	1	0	0.312869	4.377476	2.619970
90	1	0	1.687403	4.522407	1.522330
91	1	0	0.298316	5.641549	1.357570

Cartesian coordinates of **9b-3**

Center Number	Atomic Number	Atomic Type	Coordinates (Angstroms) X	Y	Z
1	6	0	3.684132	1.210108	1.181174
2	6	0	3.840614	-0.121466	0.470569

3	6	0	2.584675	-1.017507	0.719876
4	6	0	1.272551	-0.186650	0.593897
5	6	0	1.366888	0.627562	1.900062
6	6	0	2.572201	1.554875	1.837487
7	6	0	0.999854	0.654393	-0.655176
8	6	0	-0.358469	1.435247	-0.666119
9	6	0	-1.548334	1.097741	0.288903
10	6	0	-1.184168	0.733359	1.732600
11	6	0	-2.593407	0.101015	-0.283730
12	6	0	-3.798461	-0.002338	0.743019
13	6	0	-3.281052	-0.672729	2.030915
14	6	0	-1.976592	-0.051647	2.480401
15	6	0	-3.304380	0.533519	-1.590088
16	6	0	-4.708370	-0.117121	-1.526712
17	6	0	-4.844763	-0.772427	-0.115692
18	6	0	0.070741	1.347488	2.315685
19	6	0	-4.362075	1.390139	1.125732
20	6	0	-1.950038	-1.280071	-0.561282
21	6	0	2.640668	-2.309587	-0.102398
22	7	0	4.160846	0.049122	-0.956750
23	6	0	5.205793	-0.544925	-1.638837
24	6	0	-6.326473	-0.848575	0.314675
25	7	0	-7.086327	-1.578339	-0.747914
26	8	0	-4.870499	-1.064472	-2.573902
27	6	0	2.512275	-1.350097	2.247220
28	8	0	5.100810	-0.815575	-2.831411
29	6	0	6.495653	-0.811507	-0.911400
30	6	0	7.037549	0.063320	0.040321
31	6	0	8.276892	-0.206176	0.622727
32	6	0	8.981844	-1.356707	0.266058
33	6	0	8.453355	-2.227379	-0.691023
34	6	0	7.225217	-1.948876	-1.285947
35	8	0	1.165556	-0.097147	-1.865316
36	6	0	-6.559465	-1.411107	1.727953
37	6	0	-6.992643	-3.037801	-0.674396
38	6	0	-8.481603	-1.155734	-0.839268
39	8	0	1.627041	-0.413551	2.868515
40	1	0	-4.491277	-1.805322	-0.209786
41	1	0	0.440958	-0.886587	0.731717
42	1	0	-2.068733	2.063108	0.338488
43	8	0	0.035361	2.827280	-0.419376
44	6	0	-0.620279	3.798100	-1.101536
45	6	0	-0.045421	5.157191	-0.775220
46	8	0	-1.536484	3.592659	-1.867303

47	1	0	4.537219	1.885202	1.145287
48	1	0	4.697899	-0.643121	0.910135
49	1	0	2.513604	2.503876	2.366972
50	1	0	1.755755	1.437790	-0.734314
51	1	0	-0.727964	1.394932	-1.692302
52	1	0	-4.011761	-0.574941	2.844395
53	1	0	-3.152425	-1.756372	1.886790
54	1	0	-1.676559	-0.252060	3.508654
55	1	0	-2.784176	0.184272	-2.487245
56	1	0	-3.373130	1.622299	-1.668893
57	1	0	-5.478956	0.664447	-1.627593
58	1	0	0.139648	2.395813	2.005282
59	1	0	0.032846	1.334603	3.410289
60	1	0	-3.665111	1.949490	1.755282
61	1	0	-4.602210	2.006329	0.254181
62	1	0	-5.281881	1.273480	1.708854
63	1	0	-1.543151	-1.758440	0.333244
64	1	0	-2.654750	-1.966517	-1.035586
65	1	0	-1.136788	-1.149654	-1.282698
66	1	0	2.722189	-2.119155	-1.174624
67	1	0	3.509849	-2.914028	0.187332
68	1	0	1.744777	-2.916170	0.077932
69	1	0	3.355264	0.146673	-1.572768
70	1	0	-6.732529	0.170059	0.292498
71	1	0	-5.776001	-1.401372	-2.421530
72	1	0	2.104828	-2.354719	2.413107
73	1	0	3.504454	-1.304356	2.717339
74	1	0	6.505611	0.972453	0.303511
75	1	0	8.693319	0.485697	1.349985
76	1	0	9.944051	-1.569591	0.724295
77	1	0	9.004666	-3.118118	-0.980110
78	1	0	6.815793	-2.598599	-2.052869
79	1	0	0.755300	-0.969124	-1.757361
80	1	0	-6.200391	-0.714699	2.491009
81	1	0	-6.042118	-2.364571	1.880912
82	1	0	-7.627232	-1.573544	1.915799
83	1	0	-7.439183	-3.468015	-1.577804
84	1	0	-5.945614	-3.351020	-0.640579
85	1	0	-7.514145	-3.471831	0.195929
86	1	0	-8.950426	-1.640116	-1.703072
87	1	0	-8.532586	-0.072596	-0.989658
88	1	0	-9.083751	-1.409989	0.052509
89	1	0	1.009575	5.196970	-1.065822
90	1	0	-0.605328	5.925148	-1.309820

91	1	0	-0.094800	5.341162	0.302997

Cartesian coordinates of **9b-4**

Center Number	Atomic Number	Atomic Type	Coordinates (Angstroms) X	Y	Z
1	6	0	3.730007	0.510742	-1.350011
2	6	0	3.705613	0.917104	0.114064
3	6	0	2.322113	1.526090	0.486312
4	6	0	1.148145	0.696797	-0.113821
5	6	0	1.313186	1.074976	-1.596882
6	6	0	2.647491	0.572133	-2.130611
7	6	0	1.019219	-0.809759	0.097065
8	6	0	-0.383252	-1.414378	-0.279809
9	6	0	-1.543773	-0.601833	-0.943464
10	6	0	-1.188308	0.652078	-1.747007
11	6	0	-2.698589	-0.301506	0.051746
12	6	0	-3.880950	0.401856	-0.732880
13	6	0	-3.397722	1.786428	-1.203880
14	6	0	-2.032370	1.691108	-1.846422
15	6	0	-3.399812	-1.538970	0.666674
16	6	0	-4.857920	-1.091102	0.938893
17	6	0	-5.010226	0.341346	0.337790
18	6	0	0.131425	0.656889	-2.483041
19	6	0	-4.307515	-0.401101	-1.989134
20	6	0	-2.180040	0.558306	1.235089
21	6	0	2.211591	1.824059	1.985436
22	7	0	4.065851	-0.198292	1.006575
23	6	0	5.282475	-0.830462	1.097527
24	6	0	-6.481523	0.625244	-0.038057
25	7	0	-7.325215	0.412255	1.180289
26	8	0	-5.143420	-1.126261	2.328995
27	6	0	2.125055	2.835859	-0.352476
28	8	0	5.366723	-2.001407	1.459709
29	6	0	6.530396	-0.043186	0.793197
30	6	0	7.552432	-0.680264	0.076884
31	6	0	8.752773	-0.019986	-0.178697
32	6	0	8.957455	1.273953	0.307367
33	6	0	7.957568	1.902661	1.051440
34	6	0	6.747157	1.249741	1.289100
35	8	0	1.312185	-1.126968	1.459728
36	6	0	-6.721459	1.983206	-0.719879

37	6	0	-7.351100	1.543432	2.110627
38	6	0	-8.683243	-0.021478	0.860618
39	8	0	1.359850	2.514631	-1.522792
40	1	0	-4.743302	1.048816	1.130920
41	1	0	0.230886	1.180324	0.231971
42	1	0	-1.937676	-1.325046	-1.670581
43	8	0	-0.164605	-2.564300	-1.170879
44	6	0	0.376757	-3.666804	-0.619719
45	6	0	0.447443	-4.802459	-1.609841
46	8	0	0.752289	-3.735675	0.537939
47	1	0	4.686659	0.169273	-1.741873
48	1	0	4.454821	1.702641	0.256666
49	1	0	2.704942	0.303259	-3.184310
50	1	0	1.749114	-1.308935	-0.553045
51	1	0	-0.774716	-1.830913	0.649917
52	1	0	-4.095702	2.217134	-1.933453
53	1	0	-3.373961	2.502812	-0.369045
54	1	0	-1.726974	2.539884	-2.457284
55	1	0	-2.940197	-1.864369	1.604754
56	1	0	-3.379297	-2.394601	-0.018412
57	1	0	-5.551528	-1.766959	0.411649
58	1	0	0.335032	-0.347245	-2.879700
59	1	0	0.099223	1.337399	-3.341354
60	1	0	-5.212862	0.036119	-2.424744
61	1	0	-3.540582	-0.369372	-2.767623
62	1	0	-4.526776	-1.450790	-1.770877
63	1	0	-2.932753	0.653243	2.021534
64	1	0	-1.315783	0.070697	1.700829
65	1	0	-1.867529	1.561245	0.932467
66	1	0	2.991212	2.531713	2.296911
67	1	0	1.239499	2.278288	2.210021
68	1	0	2.310199	0.921201	2.588653
69	1	0	3.287371	-0.803904	1.270181
70	1	0	-6.804443	-0.157262	-0.735698
71	1	0	-6.064075	-0.795742	2.366754
72	1	0	1.562447	3.584843	0.217003
73	1	0	3.087590	3.278466	-0.642822
74	1	0	7.393633	-1.698862	-0.263689
75	1	0	9.534150	-0.518357	-0.746337
76	1	0	9.897122	1.785597	0.116419
77	1	0	8.121681	2.899285	1.452734
78	1	0	5.980910	1.730879	1.890586
79	1	0	1.225501	-2.100835	1.519681
80	1	0	-6.275709	2.810131	-0.156630

81	1	0	−7.793248	2.186691	−0.826896
82	1	0	−6.288184	1.996441	−1.723934
83	1	0	−7.886767	2.425424	1.719586
84	1	0	−7.852345	1.231968	3.033818
85	1	0	−6.333257	1.845948	2.371165
86	1	0	−8.649145	−0.922741	0.240107
87	1	0	−9.280801	0.742187	0.329799
88	1	0	−9.211662	−0.268063	1.788199
89	1	0	0.988459	−4.486148	−2.507195
90	1	0	0.949809	−5.654226	−1.150890
91	1	0	−0.562605	−5.089772	−1.920804

Myribuxine J (**10**)

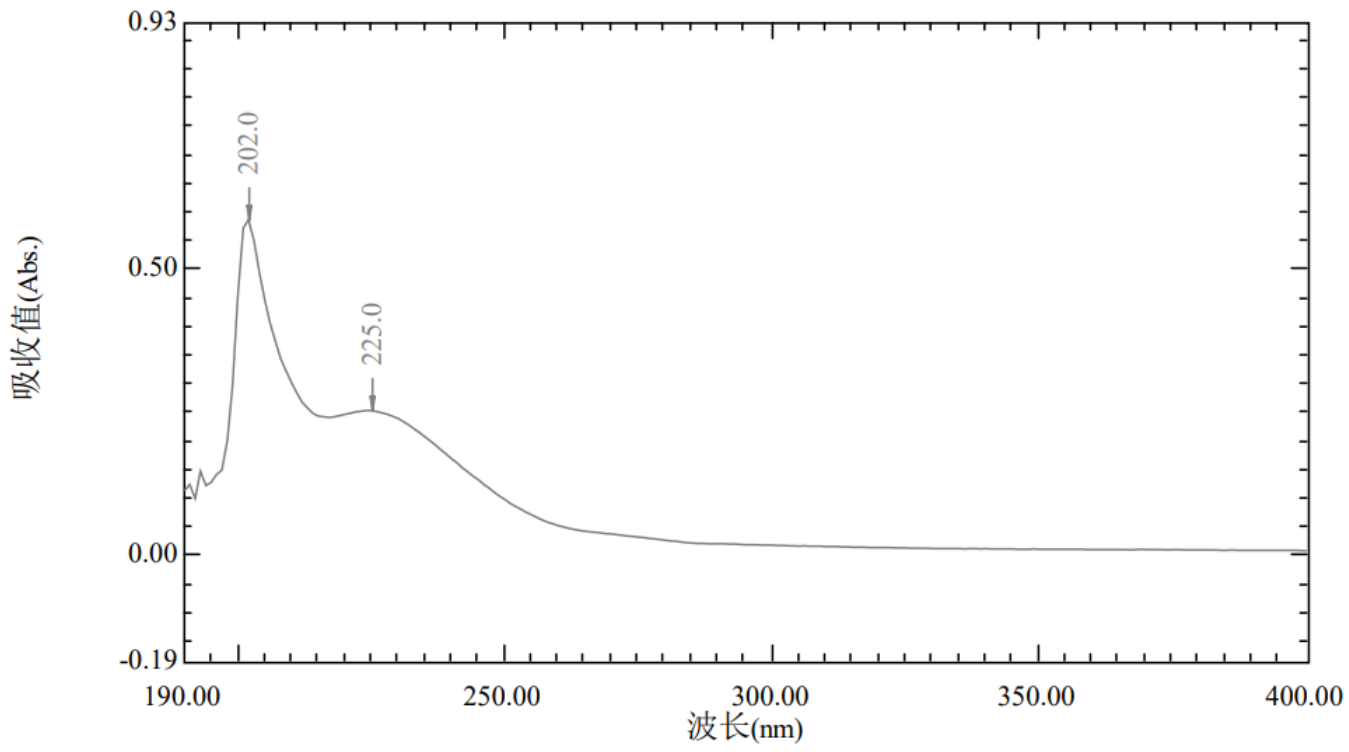

Figure S10.1 UV spectrum of compound **10**

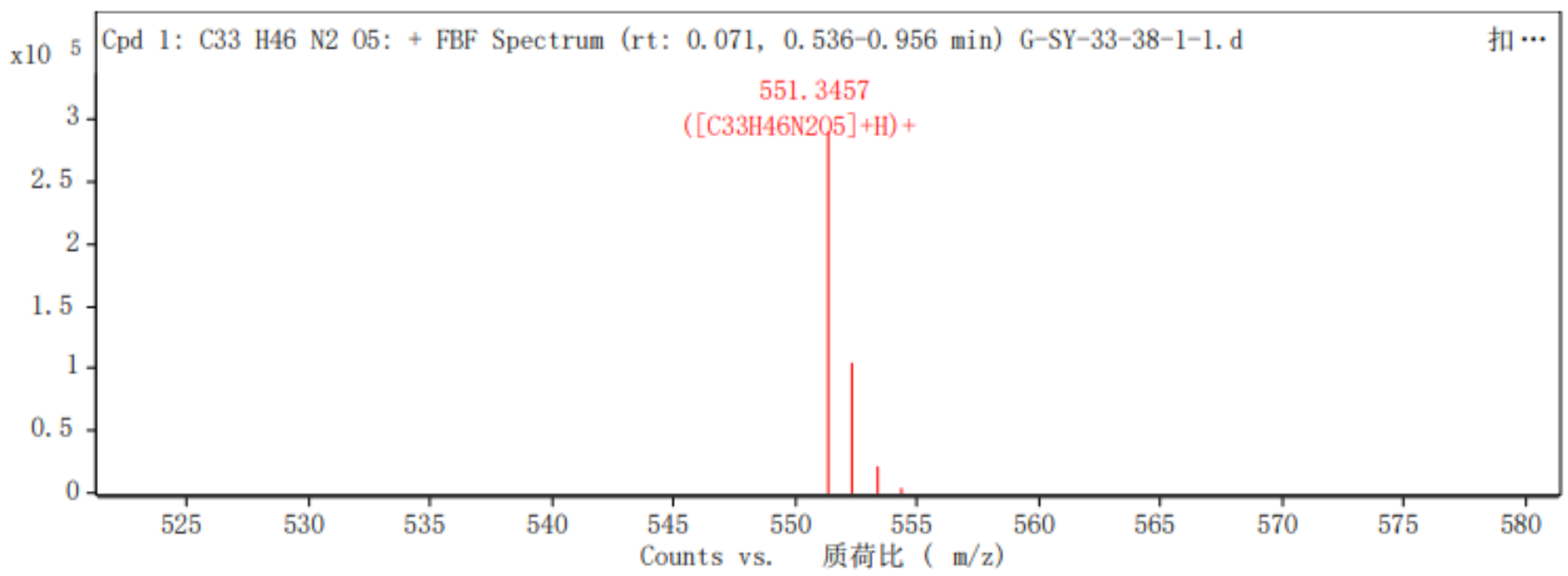

Figure S10.2 HR-ESI-MS spectrum of compound **10**

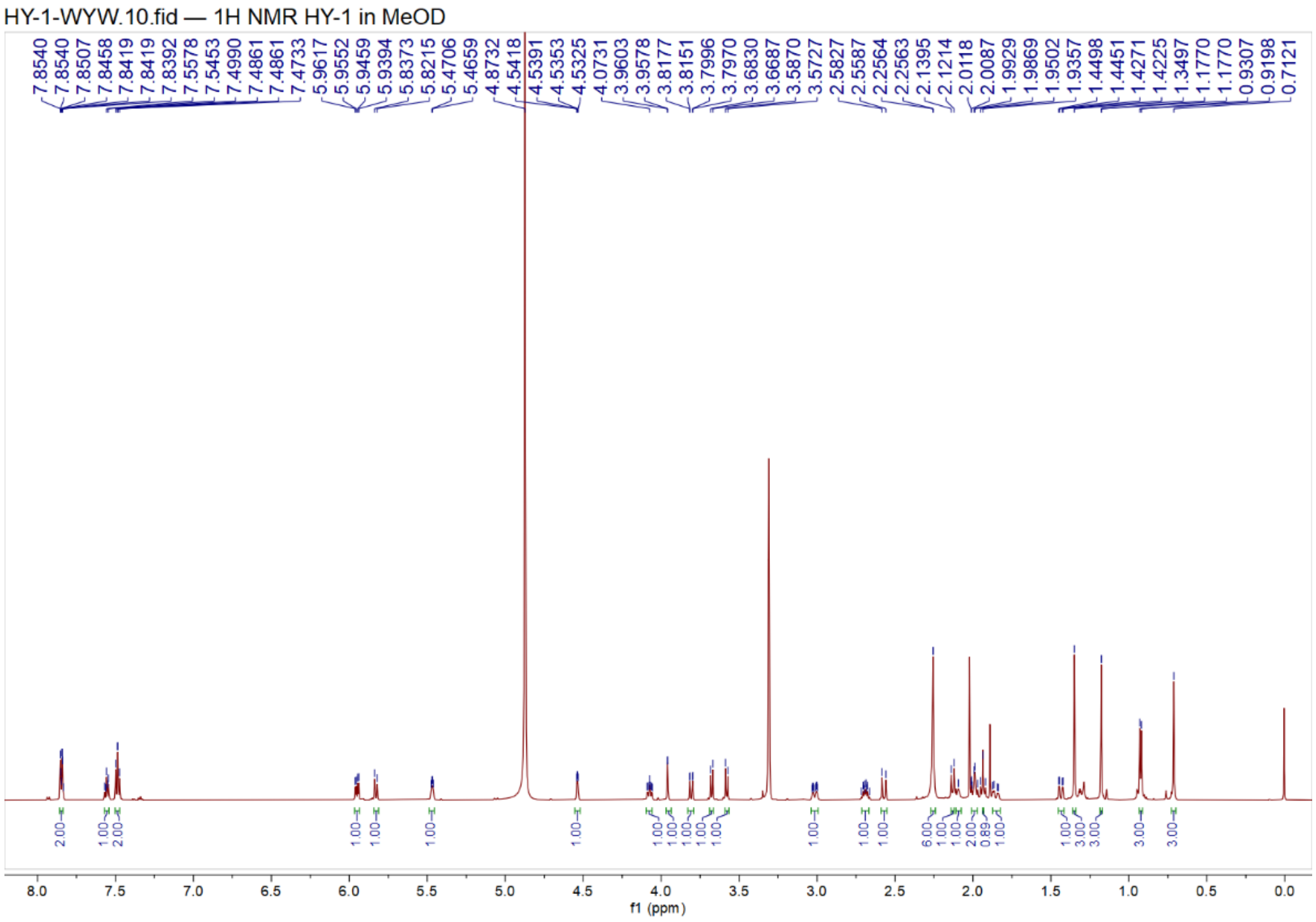

Figure S10.3 ^{1}H NMR spectrum of compound **10** (600 MHz, MeOD)

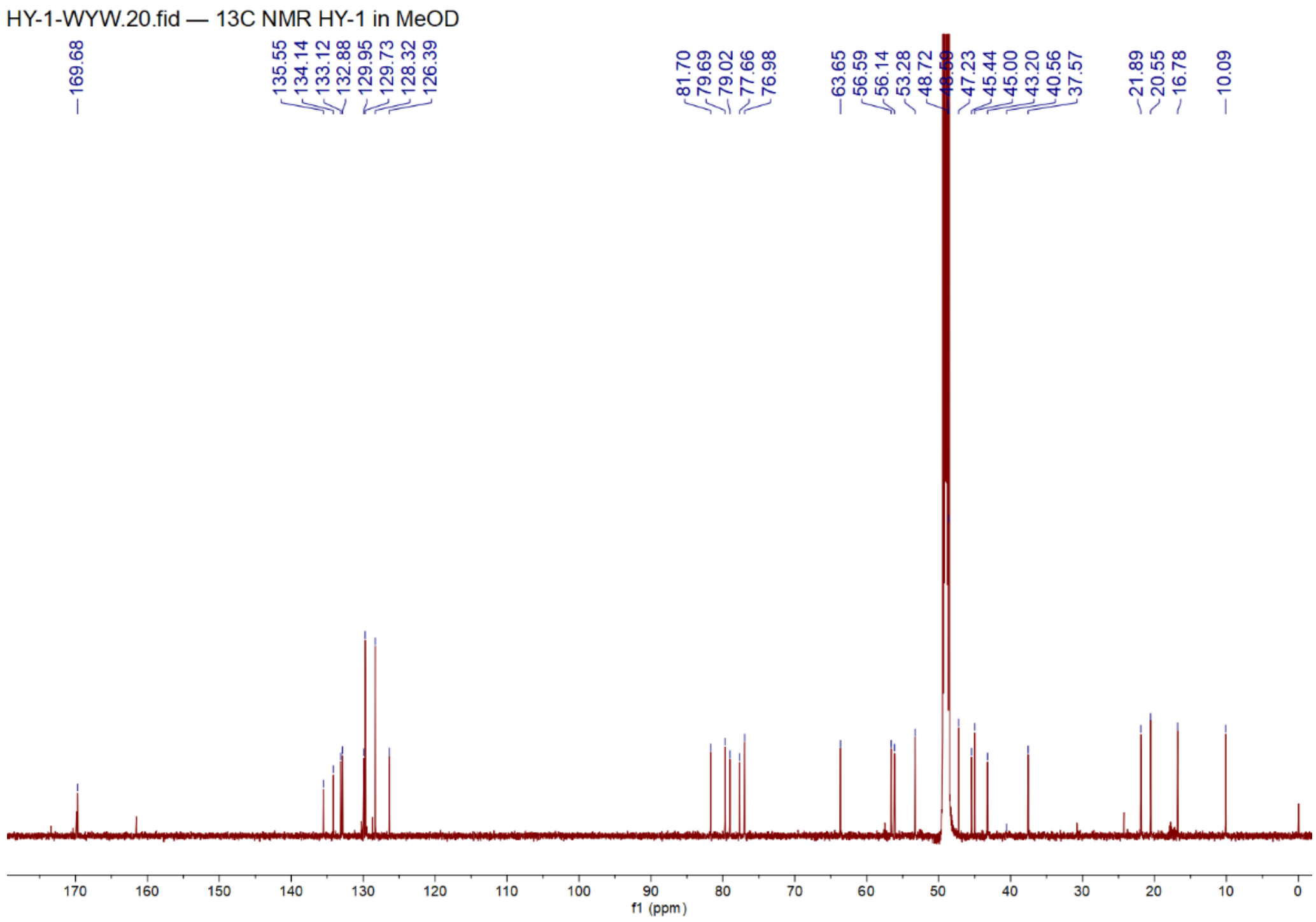

Figure S10.4 ^{13}C NMR spectrum of compound **10** (150 MHz, MeOD)

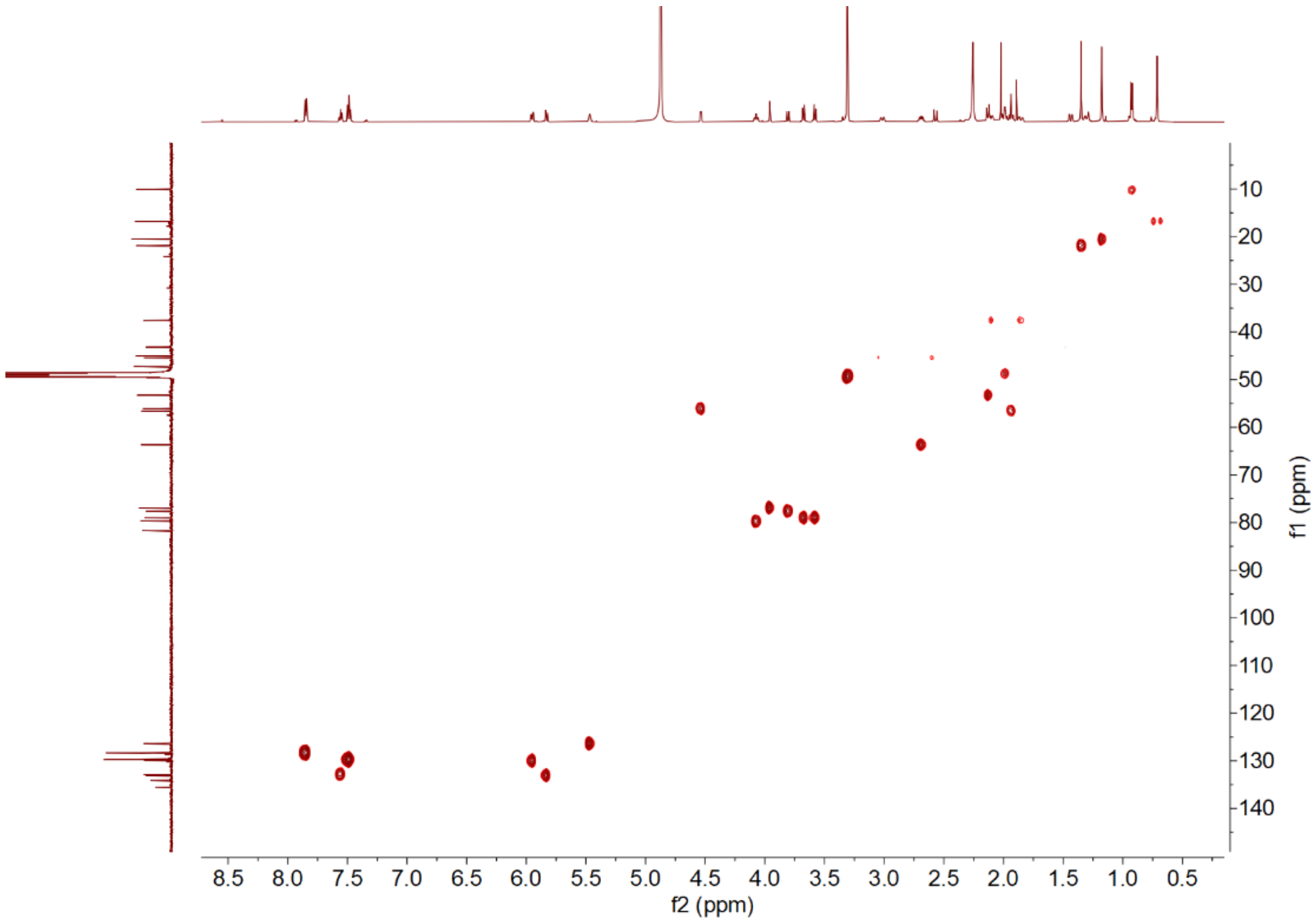

Figure S10.5 HSQC spectrum of compound **10**

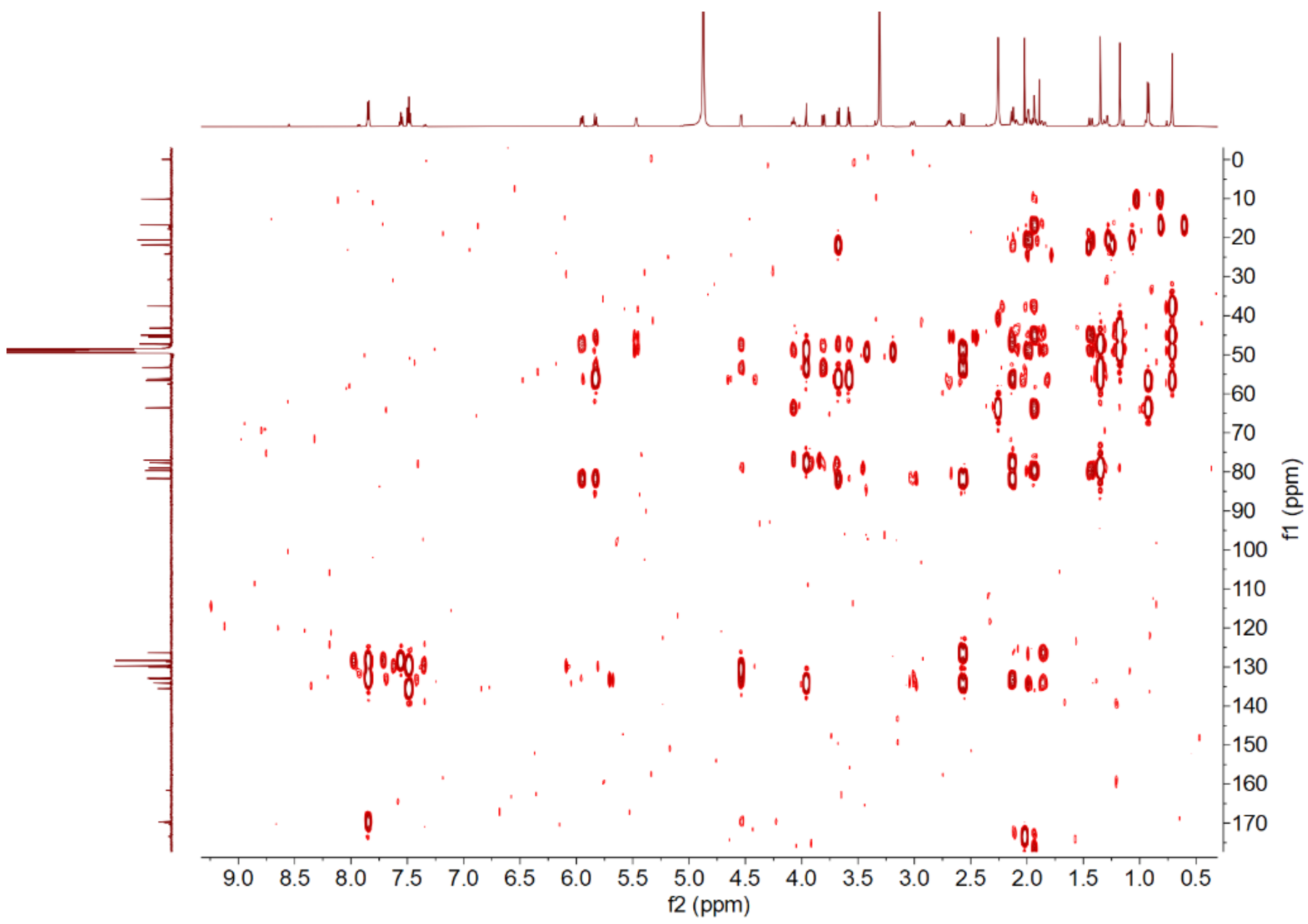

Figure S10.6 HMBC spectrum of compound **10**

Myribuxine K (**11**)

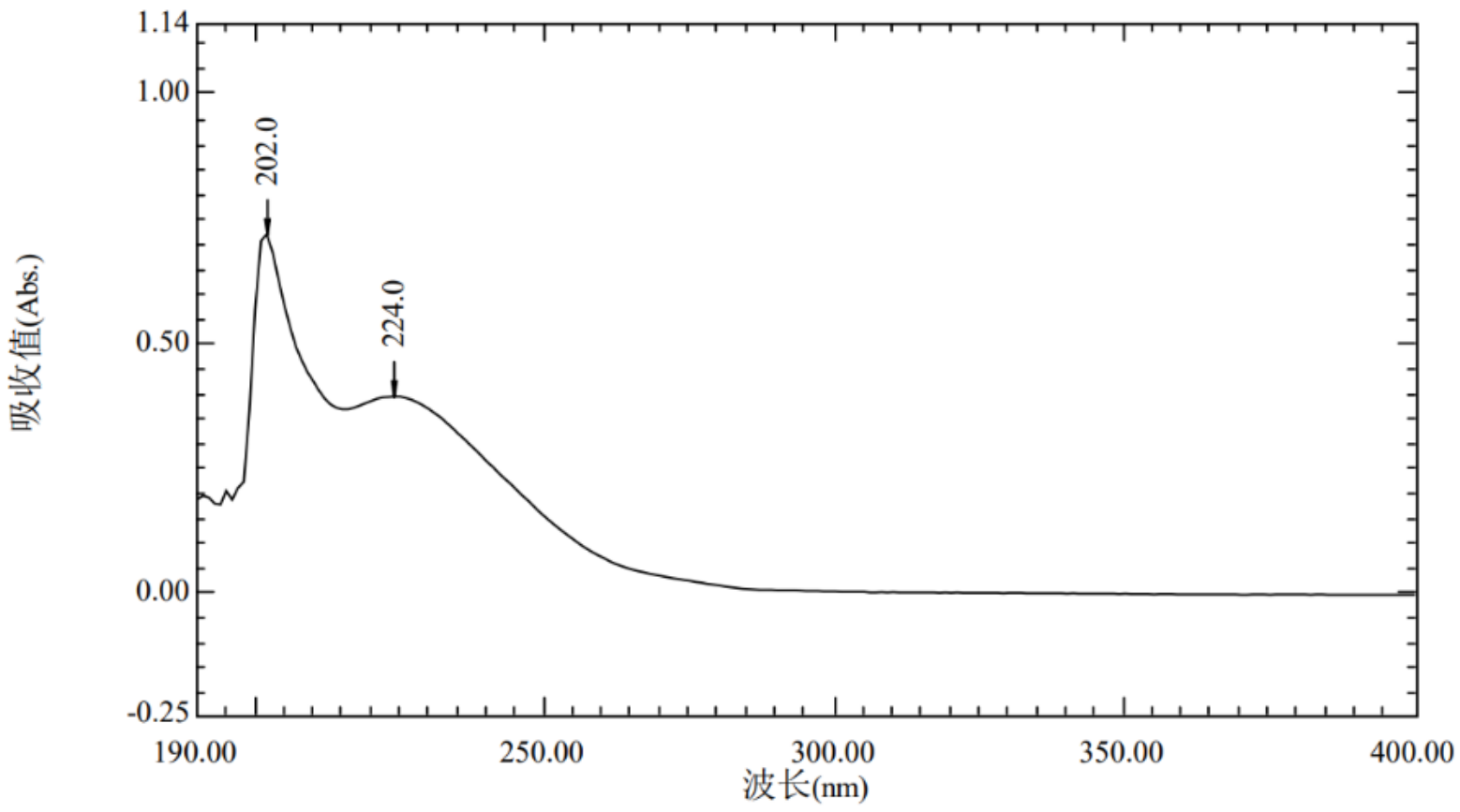

Figure S11.1 UV spectrum of compound **11**

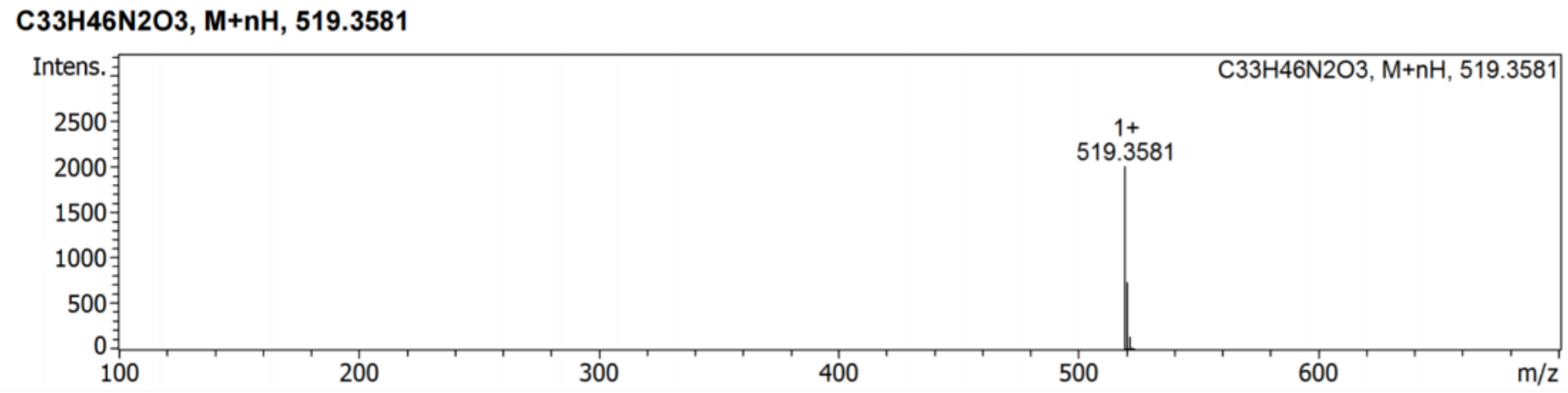

Figure S11.2 HR-ESI-MS spectrum of compound **11**

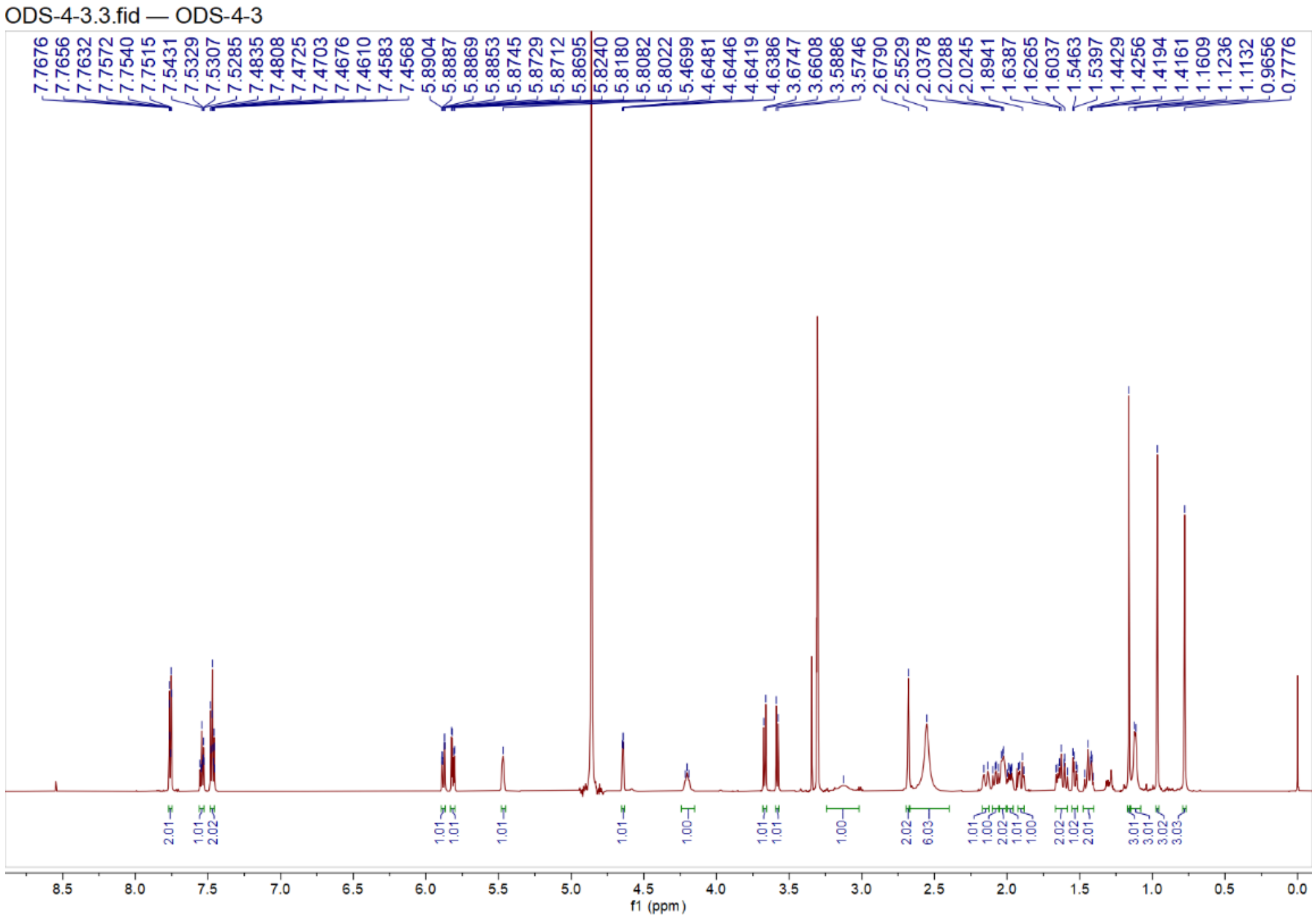

Figure S11.3 ^{1}H NMR spectrum of compound **11** (600 MHz, MeOD)

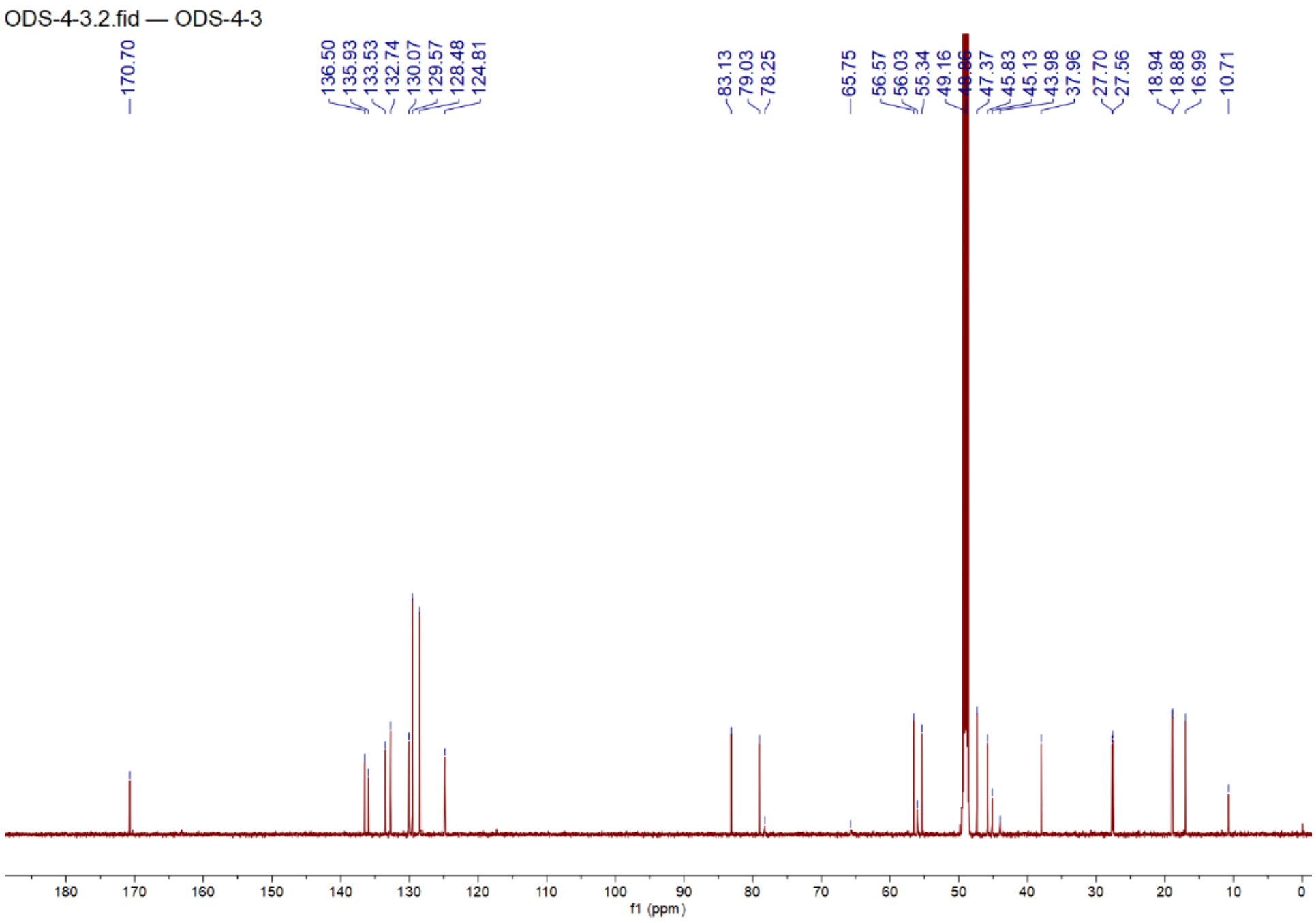

Figure S11.4 ^{13}C NMR spectrum of compound **11** (150 MHz, MeOD)

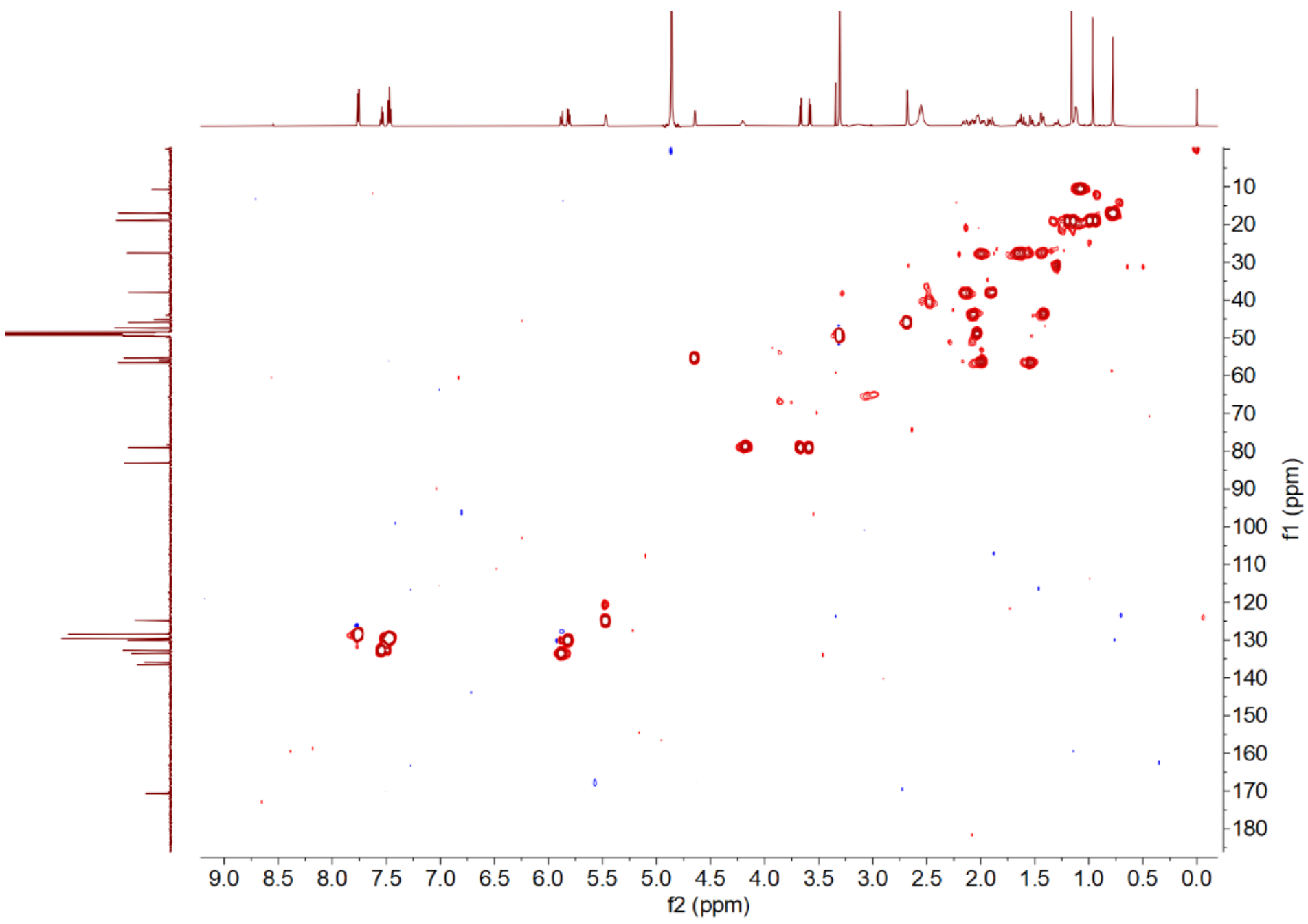

Figure S11.5 HSQC spectrum of compound **11**

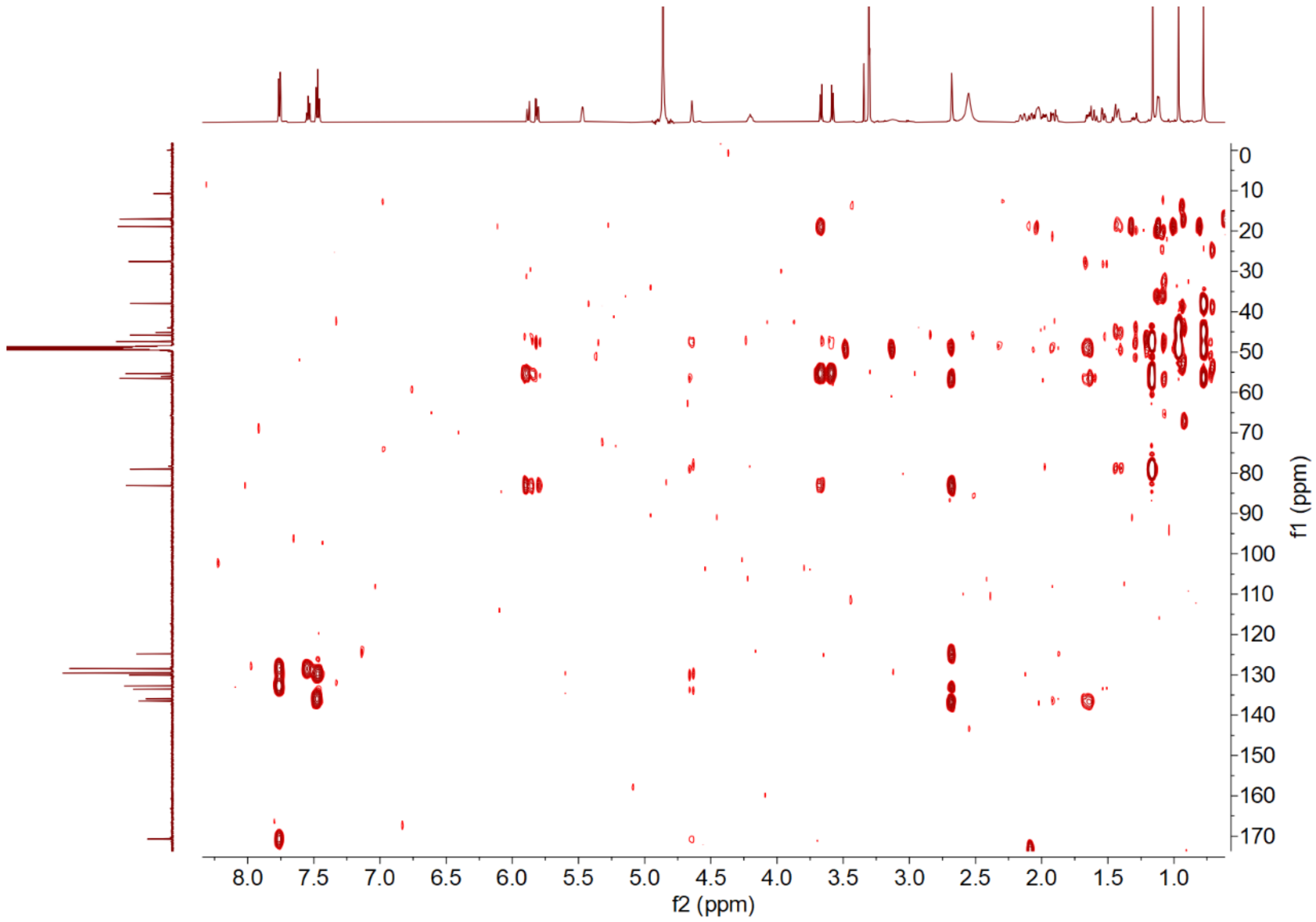

Figure S11.6 HMBC spectrum of compound **11**

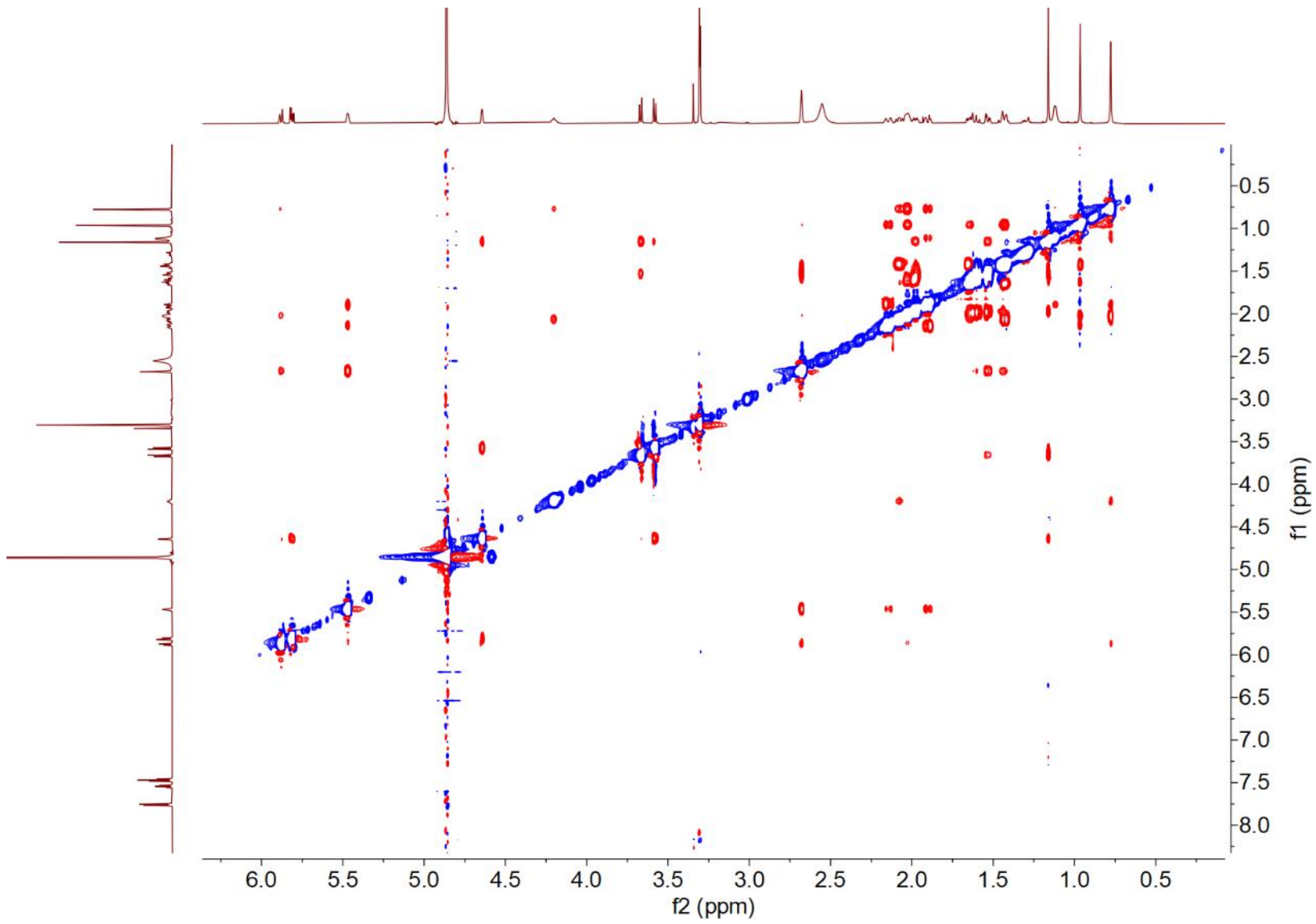

Figure S11.7 NOESY spectrum of compound **11**

Myribuxine L (**12**)

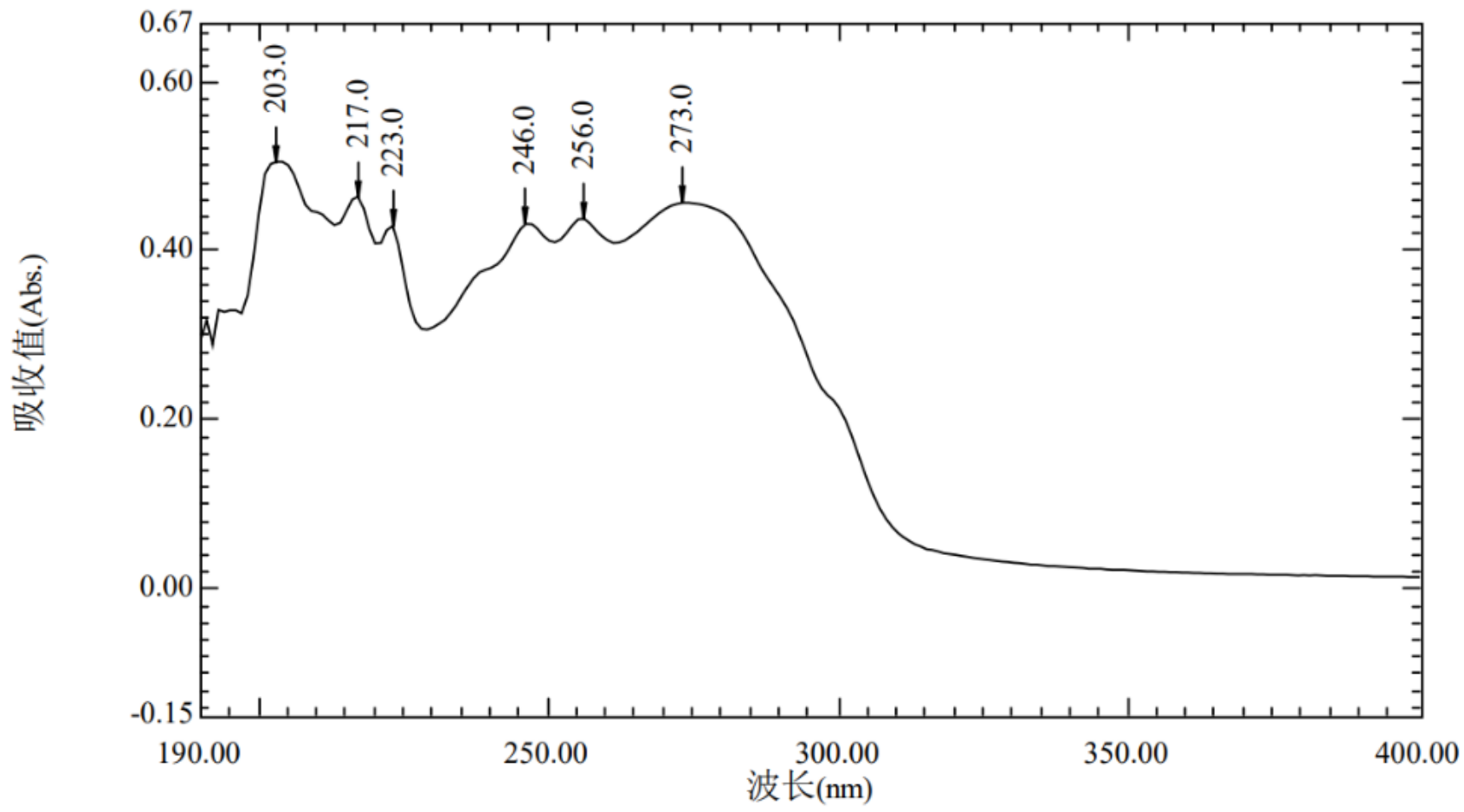

Figure S12.1 UV spectrum of compound **12**

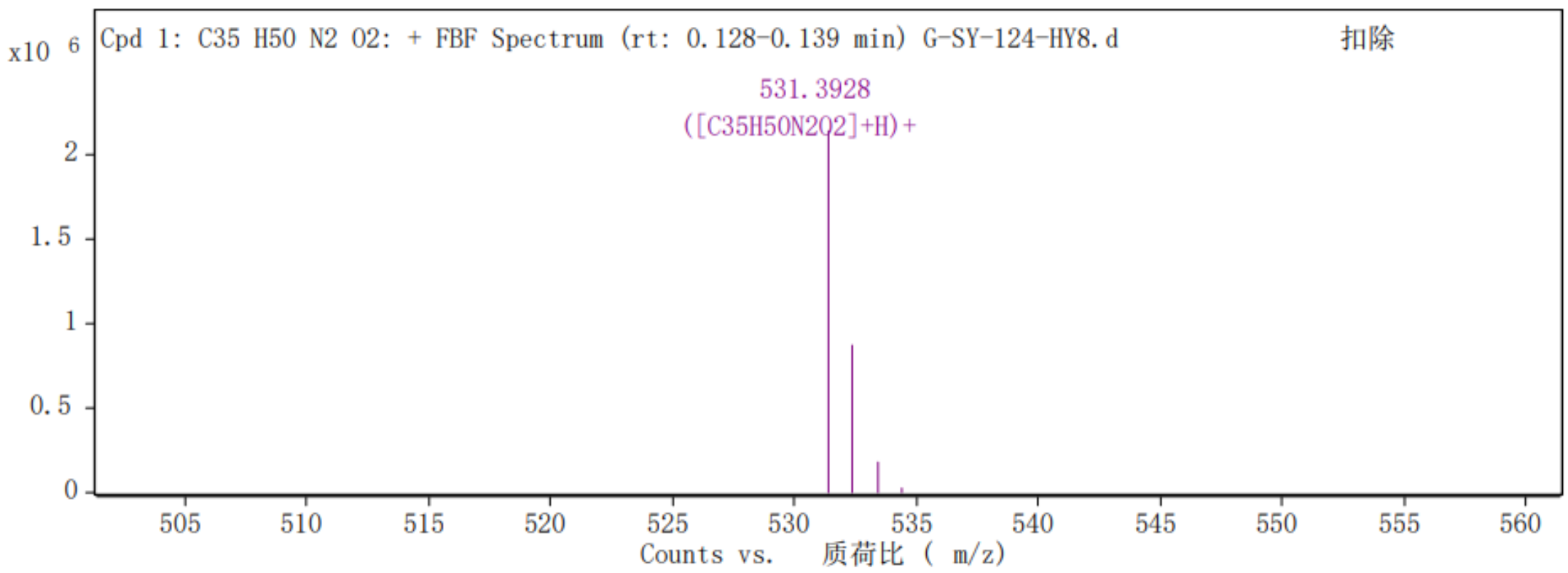

Figure S12.2 HR-ESI-MS spectrum of compound **12**

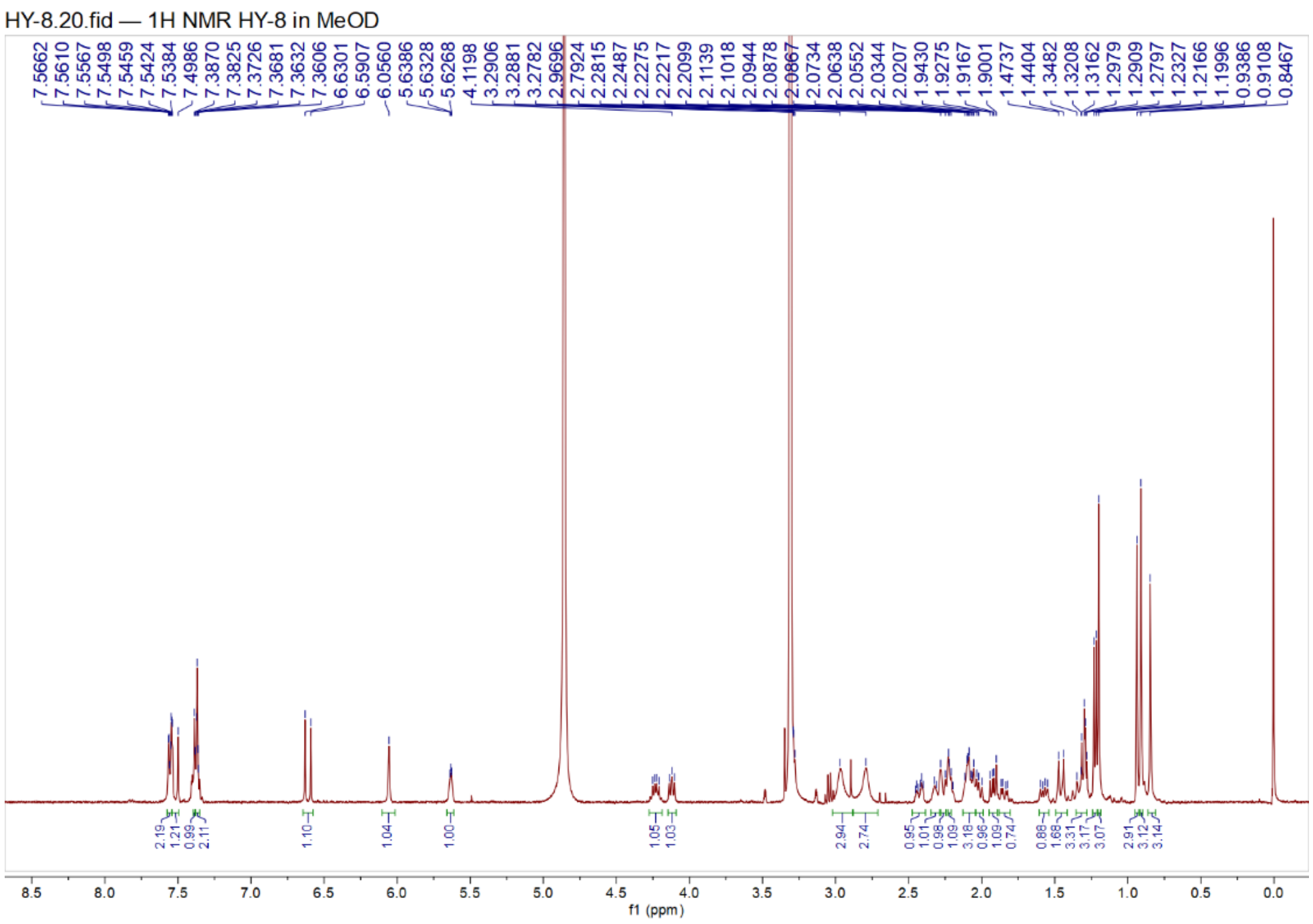

Figure S12.3 ^{1}H NMR spectrum of compound **12** (400 MHz, MeOD)

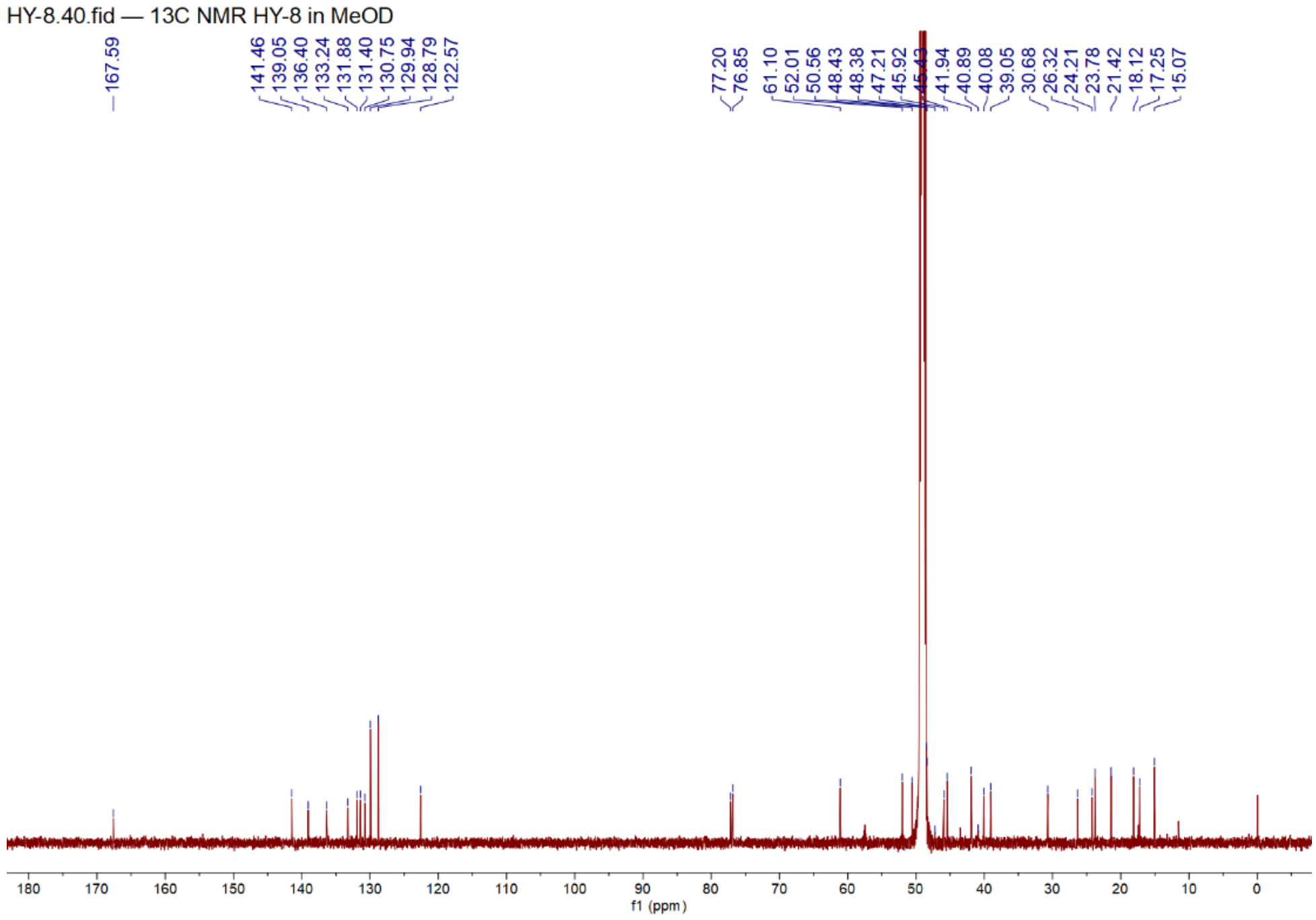

Figure S12.4 ^{13}C NMR spectrum of compound **12** (100 MHz, MeOD)

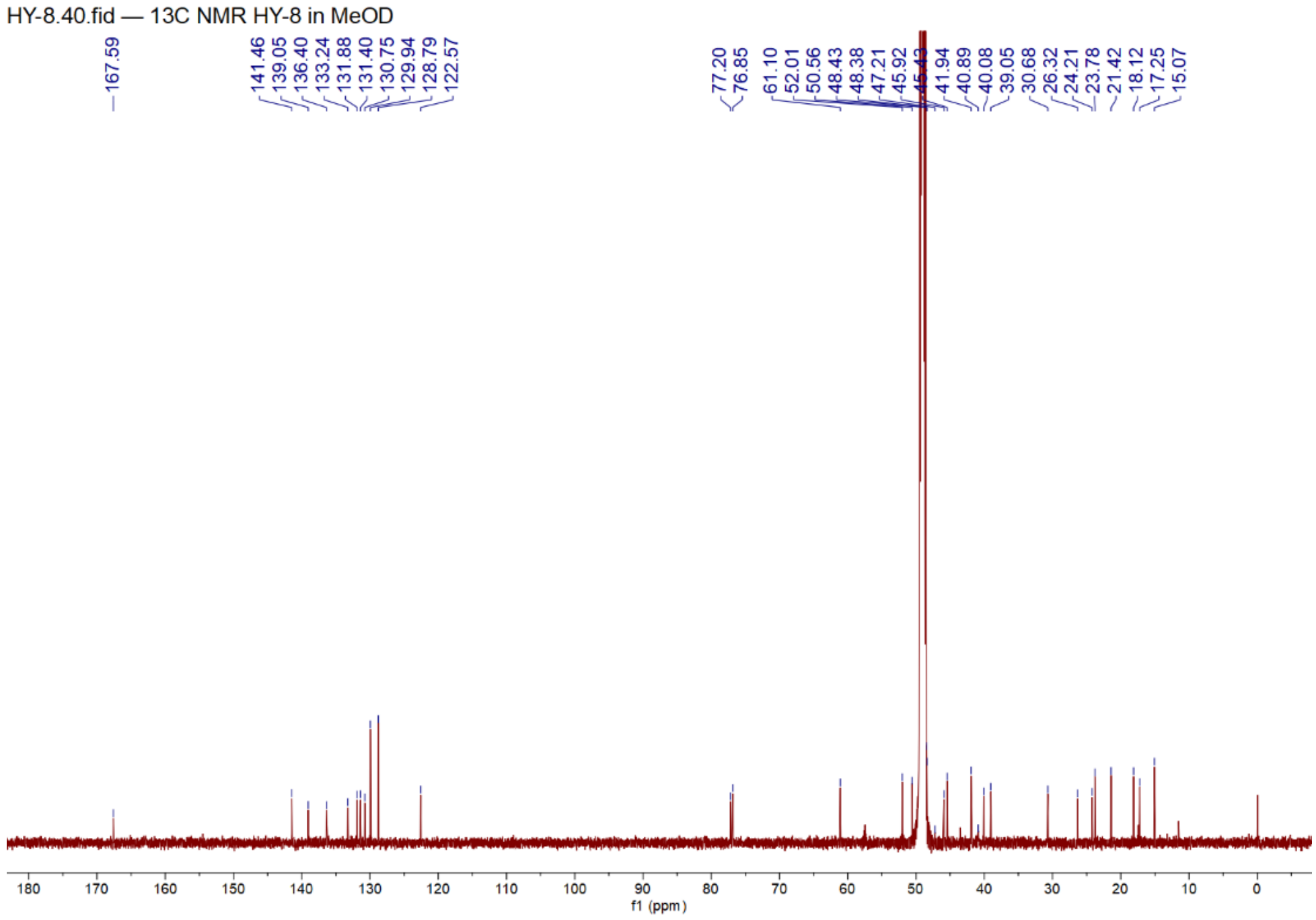

Figure S12.5 HSQC spectrum of compound **12**

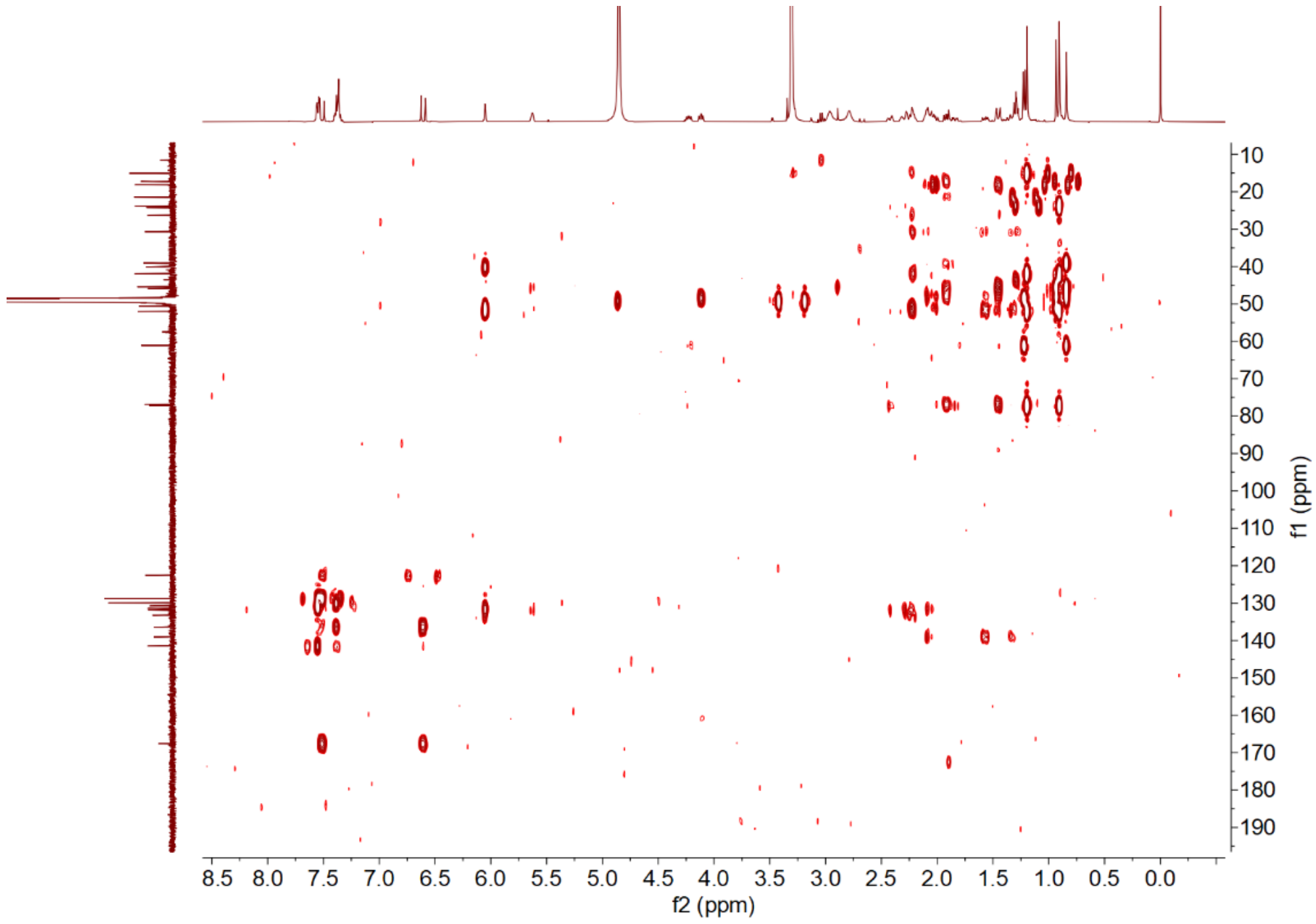

Figure S12.6 HMBC spectrum of compound **12**

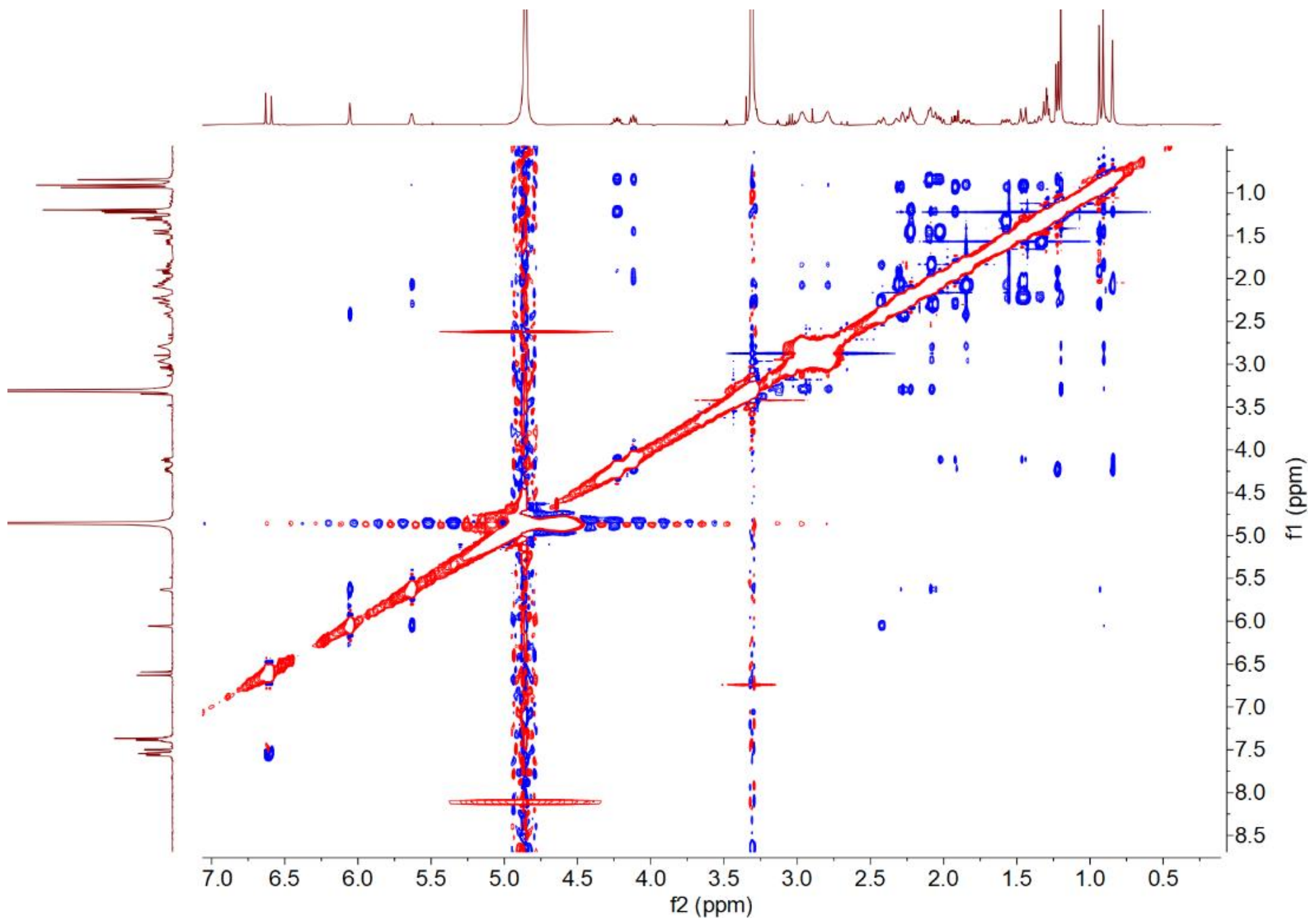

Figure S12.7 NOESY spectrum of compound **12**
